# Lactone Enolates of Isochroman-3-ones and 2-Coumaranones: Quantification of Their Nucleophilicity in DMSO and Conjugate Additions to Chalcones

**DOI:** 10.1021/acs.joc.4c00277

**Published:** 2024-04-30

**Authors:** Mohammad Sadeq Mousavi, Antonia Di Mola, Giovanni Pierri, Consiglia Tedesco, Magenta J. Hensinger, Aijia Sun, Yilan Wang, Peter Mayer, Armin R. Ofial, Antonio Massa

**Affiliations:** † Dipartimento di Chimica e Biologia “A. Zambelli”, Università degli Studi di Salerno, Via Giovanni Paolo II, 84084 Fisciano, SA, Italy; ‡ Department Chemie, Ludwig-Maximilians-Universität München, Butenandtstr. 5-13, 81377 München, Germany

## Abstract

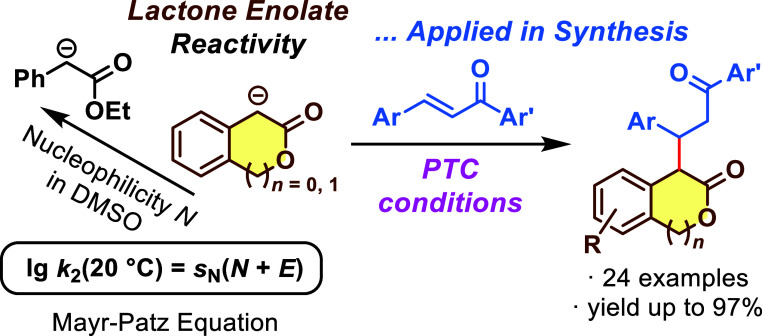

Owing to stereoelectronic effects, lactones often deviate in reactivity from their open-chain ester analogues as demonstrated by the CH acidity (in DMSO) of 3-isochromanone (p*K*_a_ = 18.8) and 2-coumaranone (p*K*_a_ = 13.5), which is higher than that of ethyl phenylacetate (p*K*_a_ = 22.6). We have now characterized the reactivity of the lactone enolates derived from 3-isochromanone and 2-coumaranone by following the kinetics of their Michael reactions with *p*-quinone methides and arylidenemalonates (reference electrophiles) in DMSO at 20 °C. Evaluation of the experimentally determined second-order rate constants *k*_2_ by the Mayr–Patz equation, lg *k*_2_ = *s*_N_(*N* + *E*), furnished the nucleophilicity parameters *N* (and *s*_N_) of the lactone enolates. By localizing their position on the Mayr nucleophilicity scale, the scope of their electrophilic reaction partners becomes predictable, and we demonstrate a novel catalytic methodology for a series of carbon–carbon bond-forming reactions of lactone enolates with chalcones under phase transfer conditions in toluene.

## Introduction

**Figure 1:**
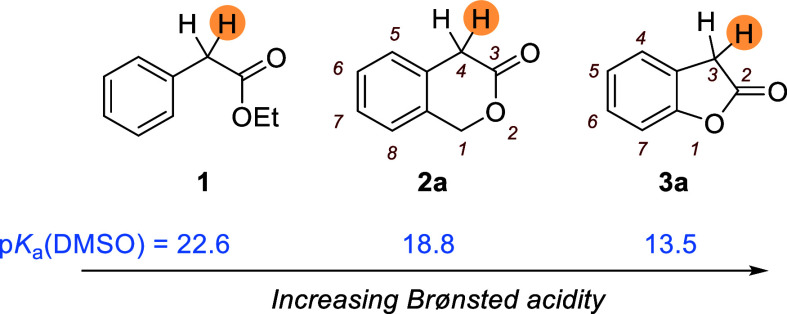
C–H acidities (p*K*_a_ in DMSO, data from ref (4)) of the ester **1** and the lactones **2a** and **3a**.

The development of new applications of well-known reaction modes is of paramount importance in organic chemistry, which usually stems from the identification of new nucleophiles or electrophiles. When there is the necessity to develop catalytic reactions, a pronucleophile should have suitable Brønsted acidity to be deprotonated under relatively mild conditions to guarantee turnover during the reaction. At the same time, the deprotonated species should be sufficiently nucleophilic to react at a reasonable rate with the electrophilic reaction partner. In this context, monoesters, such as ethyl phenylacetate **1**, have rarely been applied as pronucleophiles and required mostly the use of stoichiometric amounts of strong bases to undergo reactions.^1^ If additional strong electron-withdrawing groups (EWGs) are present on the aromatic ring in the ortho or para position to stabilize the formed ester enolate ion, 2-arylacetate esters become suitable for organocatalytic reactions.^2^ Another viable strategy includes the addition of isothiourea to activate the 2-phenylacetates as C1 ammonium enolates.^3^ The observed reactivity of arylacetates in organic syntheses can be ascribed to the relatively low Brønsted acidity of alkyl phenylacetates. For example, a p*K*_a_ of 22.6 (in DMSO) has been estimated for ethyl phenylacetate **1** (Figure 1).^4^

Differently, the structurally related lactones 3-isochromanone (**2a**) and 2-coumaranone [**3a**, also known as benzofuran-2(3*H*)-one] have p*K*_a_(DMSO) values of 18.8 and 13.5, respectively, close to the C–H acidity level of methyl *p*-nitrophenylacetate **1′** (p*K*_a_ = 15.1 in DMSO^2b^). The surprisingly high CH acidities of **2a** and **3a**, which are by 4 and 9 orders of magnitude stronger CH acids than **1**, were rationalized as being a consequence of the locked s-(*E*) conformation of the alkoxy group, which is linked to the carbonyl carbon of the ester (or lactone) moiety. The s-(*E*) conformation causes ineffective *n*_O_ → σ*_CO_ interactions that can operate in the better-stabilized s-(*Z*) conformation of open chain esters.^5^

A limited number of catalytic reactions of 3-substituted 2-coumaranones **3** have been reported.^6^ However, to our knowledge, the use of 3-isochromanones (**2**) as pronucleophiles has so far almost been neglected. While S_N_2 alkylations at C-4 of **2a** as well as Knoevenagel-type condensations with aldehydes have occasionally been studied,^7,8^ only one example for a conjugate addition to a Michael acceptor has been reported to date. Flintoft and co-workers generated the lithium enolate of lactone **2a**, which reacted with nitroethene in THF (−78 °C to r.t., 30 min, 75% yield).^9^ This void of synthetic application is surprising since applications of benzolactones **2** and **3** can be correlated to natural products modification^10,11^ and are, thus, synthetically valuable.

As part of our research interest in the development of new methodologies for the synthesis of heterocyclic compounds,^12^ the aim of this work is the determination of nucleophilicity parameters of the lactone enolates of **2a** and **3a** in the framework of the Mayr reactivity scales^13,14^ and the investigation of their synthetic utility in Michael reactions. In particular, chalcones attracted our attention as Michael acceptors because they are an often-met motif in natural products and relevant in medicinal chemistry.^15^

## Results and Discussion

### Nucleophilicity of Lactone Enolates

**Figure 2:**
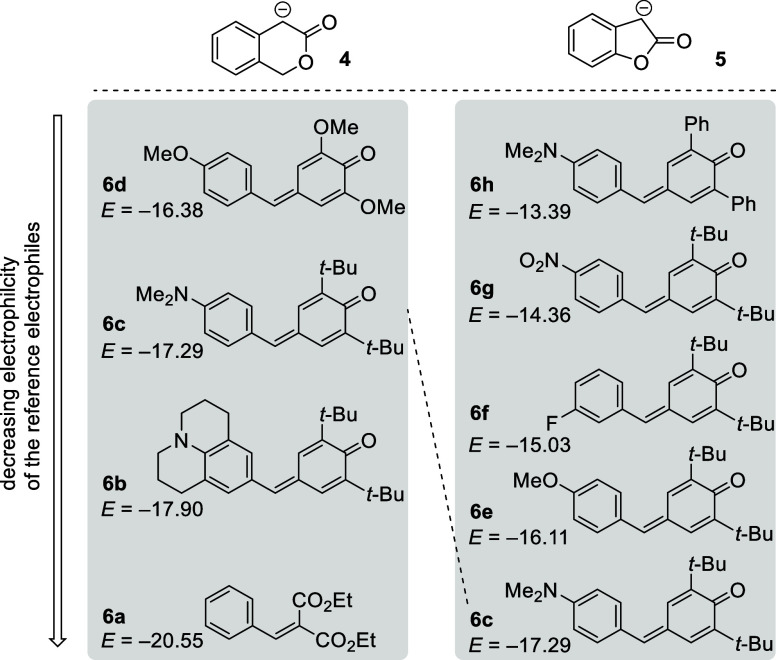
Reference electrophiles used for the characterization of the nucleophilic lactone enolates **4** and **5**. Electrophilicity parameters *E* are from refs (13a,^14^,^16^).

The more than 5 orders of magnitude different p*K*_a_(DMSO) values of the lactones **2a** and **3a** already indicate that significantly different nucleophilic reactivities have to be expected for the corresponding lactone enolate species **4** and **5**. Consequently, different sets of reference electrophiles **6** were chosen as the reaction partners for **4** and **5** in the kinetic measurements (Figure 2), which were carried out to quantify the nucleophilic reactivities of the lactone enolates in DMSO solution. The selected quinone methides and arylidenemalonates qualify as reference electrophiles because of their reliably determined electrophilic reactivity *E* on the Mayr scale^16^ and their favorable UV–vis absorbance ranges, which enabled us to follow the kinetics of their reactions with the lactone enolates **4** and **5** by photometric methods.

**Scheme 1:**
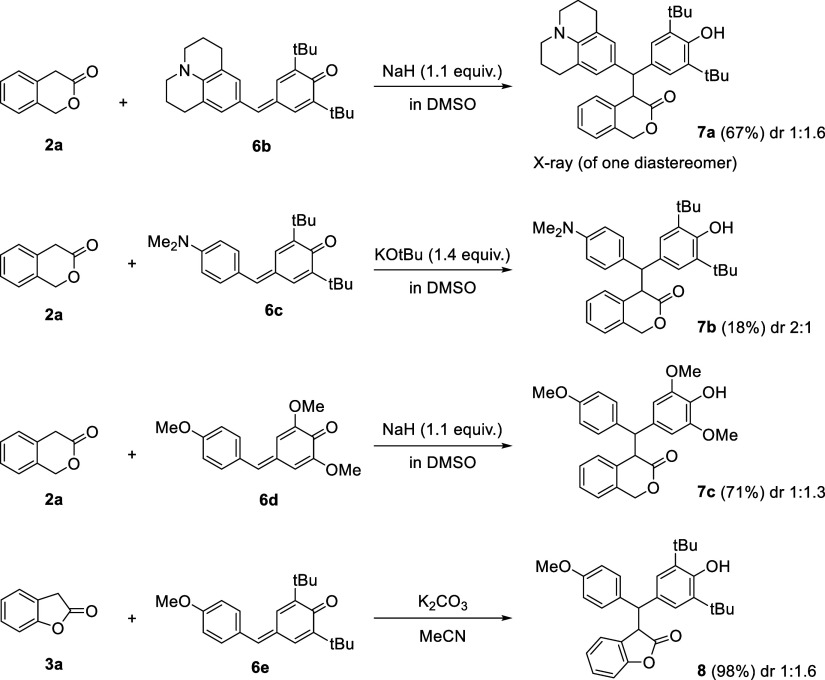
Michael Adducts of Reactions of Lactones **2a** and **3a** with Quinone Methides **6** under Basic Conditions

The lactone enolate **4** (counterion: Na^+^) was quantitatively generated in DMSO solution by deprotonation of **2a** with sodium hydride (1.1 equiv).^17^ The more acidic lactone **3a** was quantitatively deprotonated by the milder Brønsted base DBU (2.2 equiv) to generate DMSO solutions of the lactone enolate **5** (counterion: DBU-H^+^). In a first step, we investigated the products, which were formed in exemplary reactions of the lactone enolates with selected reference electrophiles. As shown in Scheme 1, all selected combinations of lactones and electrophiles reacted in the presence of different bases and solvents to the expected Michael adducts. Diastereomeric mixtures of the Michael adducts **7a**–**7c** and **8**, isolated with low diastereoselectivity, were obtained after aqueous workup. As a consequence of the selective and uniform product formations, we assumed the occurrence of analogous Michael additions for all further combinations of lactone enolates with other electrophiles, which were studied in the kinetic experiments.

The kinetics of adduct formation between **4** or **5** and the reference electrophiles **6** in DMSO (20 °C) were followed spectrophotometrically by utilizing stopped-flow techniques. The nucleophiles were used in at least 10-fold excess to achieve pseudo-first-order conditions, which enabled us to derive the first-order rate constants *k*_obs_ (s^–1^) by least-squares fitting of the function *A*_t_ = *A*_0_ exp(−*k*_obs_*t*) + *C* to the experimentally observed decay of the time-dependent absorbances of **6**. Only for the reaction of **4** with **6a**, the kinetic experiments were carried out by using the electrophile **6a** in excess (>10 equiv).

For each nucleophile–electrophile combination, *k*_obs_ was determined at four or five different concentrations of the excess reaction partner, which made it possible to calculate the second-order rate constants *k*_2_ (M^–1^ s^–1^) from the slope of the linear relationships of *k*_obs_ with the nucleophile concentration (or electrophile concentration for the reaction of **4** with **6a**). Details of all kinetics experiments are given in the Supporting Information, and the determined second-order rate constants *k*_2_ are listed in Table 1.

**Table 1:** Second-Order Rate Constants *k*_2_ (M^–1^ s^–1^) for the Reactions of Lactone Enolate Ions **4** and **5** with Reference Electrophiles **6** (in DMSO at 20 °C)

lactone enolates	electrophiles	*E*	*k*_2_ (M^–1^ s^–1^)	*N* (*s*_N_)^a^
**4** (enolate of **2a**)	**6a**	–20.55	3.79 × 10^2^	25.39 (0.54)
	**6b**	–17.90	1.40 × 10^4^	
	**6c**	–17.29	2.04 × 10^4^	
	**6d**	–16.38	6.68 × 10^4^	
**5** (enolate of **3a**)	**6c**	–17.29	5.19 × 10^1^	19.60 (0.75)
	**6e**	–16.11	3.60 × 10^2^	
	**6f**	–15.03	3.06 × 10^3^	
	**6g**	–14.36	1.31 × 10^4^	
	**6h**	–13.39	3.28 × 10^4^	
enolate of **1**	**6c**	–17.29	5.51 × 10^5^^b^	27.54 (0.57)^b^
enolate of **1′**	**6c**	–17.29	7.21 × 10^1^^b^	20.00 (0.71)^b^

a Calculated by using eq 1.

b With data from ref (18).

The second-order rate constants *k*_2_ were then evaluated by the Mayr–Patz eq 1.
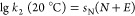
1

**Figure 3:**
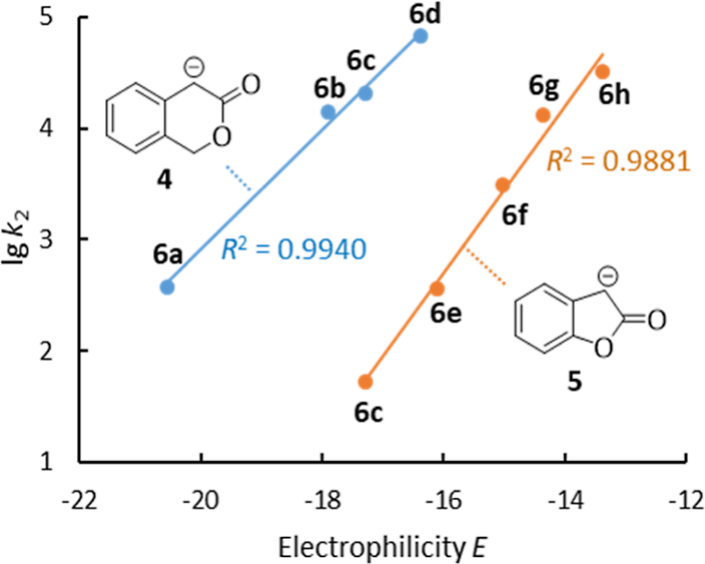
Determination of *N* and *s*_N_ for the lactone enolates **4** and **5** from the linear correlations of lg *k*_2_ with the electrophilicity parameters *E* of the reference electrophiles **6**.

In eq 1, the decadic logarithm of a second-order rate constants *k*_2_ for a nucleophile–electrophile reaction is expressed by the three parameters *E*, *N*, and *s*_N_. Given that the electrophilicities *E* of the reference electrophiles **6** were reported before, and the second-order rate constants *k*_2_ were experimentally determined in this work, the remaining parameters *N* and *s*_N_, which are characteristic for the reactivity of a nucleophile in a specific solvent, can be calculated. Thus, the quantitative descriptors *N* (and *s*_N_) for the nucleophilicities of **4** (*N* = 25.39, *s*_N_ = 0.54) and **5** (*N* = 19.60, *s*_N_ = 0.75) were derived from the linear correlations of lg *k*_2_ with the *E* parameters of the electrophilic reaction partners (Figure 3).

**Figure 4:**
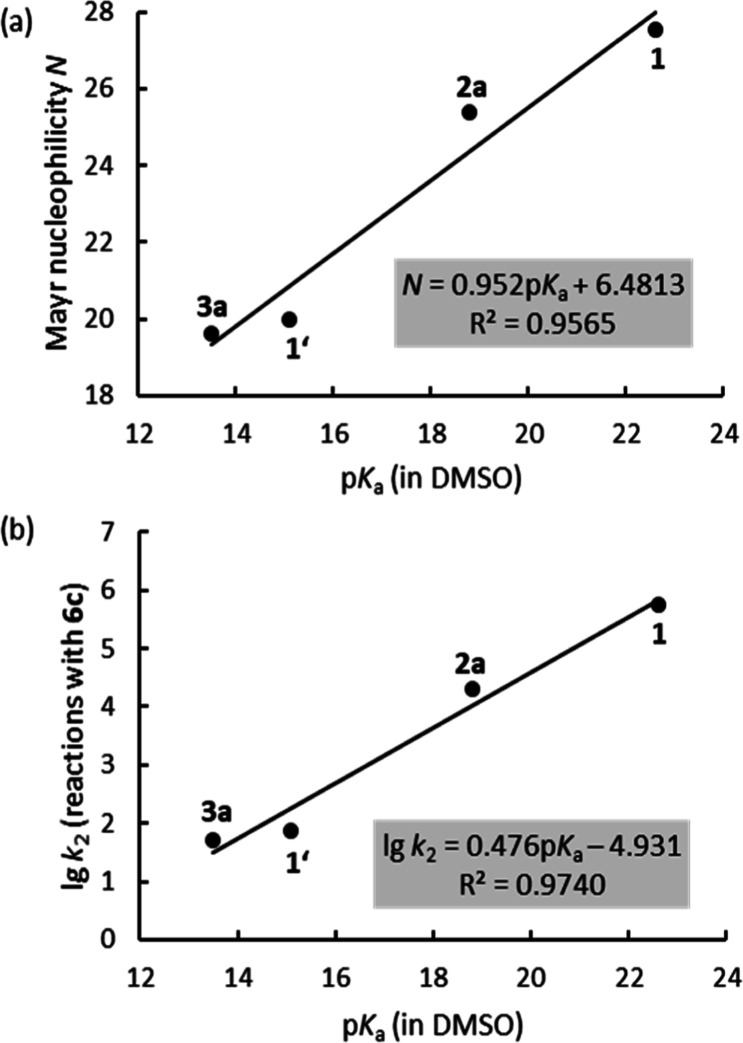
(a) Correlation of enolate nucleophilicities *N* with p*K*_a_ of the corresponding CH acids **1**, **1′**, **2a**, and **3a**. (b) The Brønsted plot shows a linear relationship between the enolate reactivities (lg *k*_2_) toward **6c** and the acidity constants p*K*_a_ of the corresponding CH acids **1**, **1′**, **2a**, and **3a**.

Interestingly, the determined *N* parameters for the carbanions derived from **1**, **1′**, **2a**, and **3a** correlate linearly with the Brønsted acidities p*K*_a_ of these CH acids. The scatter in Figure 4a (*R*^2^ = 0.9565) is rationalized by the fact that the slope parameters *s*_N_, which vary between 0.54 and 0.75 for this set of nucleophiles (see Table 1), were neglected when constructing the graph. Linear correlations of higher precision can be expected if rate constants for reactions of the enolates with a common electrophile are plotted against the p*K*_a_ values of **1**, **1′**, **2a**, and **3a**.

Accordingly, the Brønsted correlation in Figure 4b illustrates that the nucleophilic reactivities (lg *k*_2_) of the ester and lactone enolates in reactions with quinone methide **6c** correlate excellently with the Brønsted basicities of the CH acids **1**, **1′**, **2a**, and **3a** in DMSO (*R*^2^ = 0.9740). The slope of 0.476 in Figure 4b is similar to the one reported for an analogous correlation for a series of benzyl anions stabilized by ethoxycarbonyl, nitro, cyano, and sulfonyl groups (slope = 0.438, *n* = 11),^18^ which covered 14 orders of magnitude on the p*K*_a_ scale but showed much higher uncertainties (*R*^2^ = 0.74) owing to the structurally more diverse set of carbanions that undergo reactions via variable intrinsic barriers.^18^

**Scheme 2:**
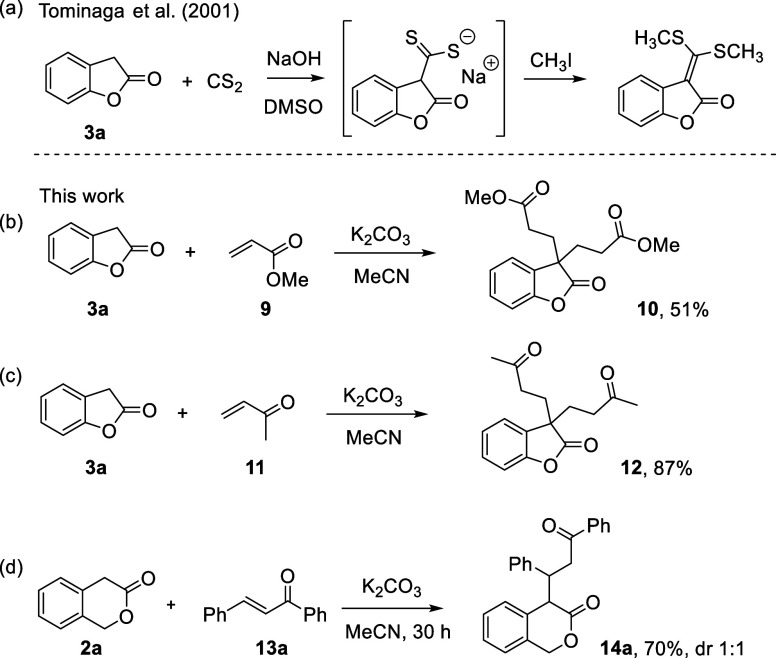
Michael Adducts of Reactions of Lactones **2a** and **3a** with (a) Carbon Disulfide, (b) Methyl Acrylate (**9**), (c) But-3-enone (**11**), and (d) Chalcone (**13a**) Yields of isolated product after chromatographic purification.

By using the Mayr nucleophilicity parameters *N* and *s*_N_, it is now possible to rationalize published synthetic procedures that involve the investigated lactone enolates. For example, Tominaga and co-workers reported that a mixture of 2-coumaranone **3a**, sodium hydroxide, and carbon disulfide in DMSO reacted at 10–15 °C to form an anionic adduct, which was trapped by methylation with iodomethane to form 3-bis(methylthio)methylene-2-coumaranone (Scheme 2a).^19^ This procedure is in good agreement with a moderately rapid reaction that can be expected from a second-order rate constant of *k*_2_ = 27 M^–1^ s^–1^ (at 20 °C), which is calculated by using eq 1, the *N* (and *s*_N_) parameter of **5** in DMSO, and the electrophilicity *E* = −17.70 for CS_2_.^20^

On the fundament of their nucleophilicity parameters, we attempted to further explore the synthetic scope of **2a** and **3a** and investigated their reactivity toward prototypical Michael acceptors, such as methyl acrylate (**9**) or the α,β-unsaturated ketones **11** and **13a**. The reactions of **3a** with the β-unsubstituted electrophiles **9** (*E* = −18.84)^21^ and **11** (*E* = −16.76),^21^ both gave the 1:2 adducts **10** and **12**, respectively (Scheme 2b,c).

Interestingly, the parent chalcone **13a** (*E* = −19.39)^21^ and lactone **2a** under basic conditions in MeCN formed product **14a** in good yield. The 4-monofunctionalized lactone **14a** corresponds to the 1:1-Michael adduct and was isolated as a 1:1 mixture of diastereomers (Scheme 2d).

### Base-Catalyzed Michael Additions of Lactones to Chalcones

**Scheme 3:**
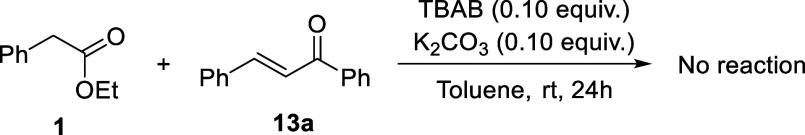
Open-Chain Ester **1** Did Not React with Chalcone **13a** under the Optimal Conditions of Table 2, Entry 6

Aiming to develop a catalytic process, we optimized the adduct formation of **2a** with the parent chalcone **13a** under phase transfer conditions. In entry 1 of Table 2, lactone **2a** and chalcone **13a** were mixed in toluene in the presence of K_2_CO_3_ (1 equiv.). Not unexpectedly, the starting materials were recovered completely after 24 h at ambient temperature (Table 1, entry 1) because of the insufficient solubility of potassium carbonate in toluene. The use of the phase transfer catalyst tetra-*n*-butyl ammonium bromide (TBAB) led to **14a** in 90% yield within 2 h (entry 2), which was both a significantly shorter reaction time and a higher yield of **14a** than from the analogous reaction performed in the more polar solvent acetonitrile but without the phase transfer catalyst (Scheme 2d). Comparable yields of **14a** were obtained even when decreasing the amounts of both TBAB and K_2_CO_3_ to 0.05 and 0.1 equiv, respectively (compare with entries 3 and 4). The catalysts amount was further decreased to 0.01 equiv with a concomitant scale up at 1 mmol, leading to high yield as well but requiring a longer reaction time (entry 5). However, the conditions were adjusted for practical reasons using 0.1 equiv of both TBAB and K_2_CO_3_, providing product formation in almost quantitative yield over 5 h (entry 6). These conditions can also be scaled up to 1 mmol with minimal impact on product yield (89% yield). The use of Li_2_CO_3_ proved to be less effective, probably because of the lower counteranion exchange rate with TBAB (entry 7). On the other hand, when the significantly less Brønsted acidic ethyl 2-phenylacetate **1** was subjected to the same conditions, even after mixing for 24 h, we recovered the starting materials unreacted. This demonstrates the importance of the p*K*_a_ of the pronucleophiles’ C–H bonds for the development of effective catalytic reactions (Scheme 3).

**Table 2:**
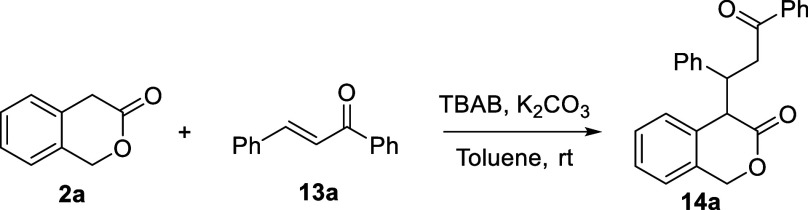
Optimization of the Michael Adduct Formation between Chalcone (**13a**) and Lactone **2a**

entry	NBu_4_Br (equiv)	base (equiv)	molarity [**2a**]	time (h)	yield (%)^a^
1		K_2_CO_3_ (1.0)	0.337	24	-b
2	0.20	K_2_CO_3_ (1.0)	0.337	2	90
3	0.05	K_2_CO_3_ (1.0)	0.337	2	88
4	0.05	K_2_CO_3_ (0.10)	0.337	2.5	86
5	0.01	K_2_CO_3_ (0.01)	0.675	48	88
**6**	**0.10**	**K**_**2**_**CO**_**3**_**(0.10)**	**0.675**	**5**	**97**
7	0.10	Li_2_CO_3_ (0.10)	0.675	36	80

a Isolated yield.

b Starting materials recovered.

The scope of the lactone–chalcone adduct formation was further explored under the optimized conditions of entry 6 of Table 2. To assess the full extent of this reaction, we synthesized several substituted 3-isochromanones with a range of electronic properties incorporated on the aromatic ring [**2b**–**2f**, Supporting Information]. Several chalcones **13** reacted with differently substituted 3-isochromanones **2a**–**2f** with both reactants bearing halogens, electron-donating groups and EWGs in different positions on the aromatic rings, showing variable reaction time as detected by thin-layer chromatography (TLC) (used to monitor the disappearance of **2**), obtaining **14** in moderate to excellent yields (Table 3). In the case of (*E*)-3-(2-chlorophenyl)-1-phenylprop-2-en-1-one **13d**, a high reaction temperature was required, probably because of the increased steric hindrance (entry 4), while at room temperature, we did not observe any reaction. 7-Nitroisochroman-3-one **2d** was not reactive at room temperature, and a higher reaction temperature (110 °C) was necessary to achieve a moderate yield in the Michael addition with the parent chalcone **13a** (entry 13). In this case, the nitro group leads to a decrease of the p*K*_a_, stabilizing the carbanion by resonance, and its nucleophilicity is lower than that of **4**, explaining the observed reactivity. On the chalcone side, even (*E*)-3-(furan-2-yl)-1-phenylprop-2-en-1-one (**13j**), the presumably least electrophilic Michael acceptor in Table 3, gave a good yield in the reaction with **2a** to give product **14j** (entry 10).

**Table 3:**
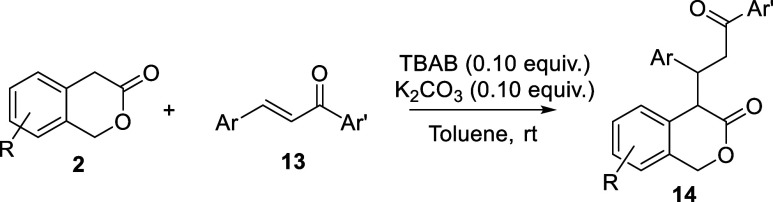
Scope Analysis for the Base-Catalyzed Michael Addition of Lactones **2** and Chalcones **13**

entry	lactones	chalcones	Ar	Ar′	time (h)	yield (%)^a^	dr^b^
1	**2a**, R = H	**13a**	Ph	Ph	5	**14a**, 97	50/50
2	**2a**, H	**13b**	4-ClC_6_H_4_	Ph	5	**14b**, 97	50/50
3	**2a**, H	**13c**	2-NO_2_C_6_H_4_	Ph	7	**14c**, 68	56/44
4^c^	**2a**, H	**13d**	2-ClC_6_H_4_	Ph	18	**14d**, 62	60/40
5	**2a**, H	**13e**	4-FC_6_H_4_	Ph	7	**14e**, 91	53/47
6	**2a**, H	**13f**	4-MeOC_6_H_4_	Ph	9	**14f**, 82	55/45
7	**2a**, H	**13g**	Ph	3-CF_3_C_6_H_4_	6	**14g**, 76	53/47
8	**2a**, H	**13h**	Ph	4-NO_2_C_6_H_4_	8	**14h**, 77	52/48
9	**2a**, H	**13i**	Ph	4-MeOC_6_H_4_	10	**14i**, 73	53/47
10	**2a**, H	**13j**	2-furyl	Ph	10	**14j**, 65	58/42
11	**2b**, 7-Br	**13a**	Ph	Ph	6	**14k**, 81	54/46
12	**2c**, 6-OMe	**13a**	Ph	Ph	3	**14l**, 96	50/50
13^d^	**2d**, 7-NO_2_	**13a**	Ph	Ph	12	**14m**, 58	53/47
14	**2e**, 6-Cl	**13a**	Ph	Ph	9	**14n**, 94	52/48
15	**2c**, 6-OMe	**13f**	4-MeOC_6_H_4_	Ph	3	**14o**, 93	51/49
16	**2f**, 8-F	**13a**	Ph	Ph	7	**14p**, 90	59/41

a Isolated yield.

b Determined by ^1^H NMR spectroscopic analysis of the crude material.

c Reaction temperature at 60 °C.

d Reaction temperature at 110 °C.

**Figure 5:**
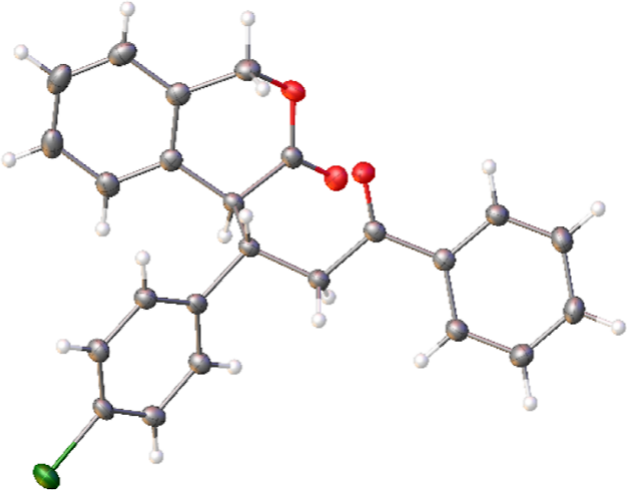
ORTEP diagram of **14b-1**.^22^ Crystals of **14b-1** were obtained by slow evaporation of a solution of **14b-1** (5 mg) in a dichloromethane/hexane mixture (1 mL, 1:5).

Low diastereoselectivity was usually observed, but the majority of diastereomeric mixtures were easily separated by chromatography or crystallization. In the case of the chloro-substituted **14b**, crystals suitable for single-crystal X-ray analysis were obtained by slow evaporation of a solution of **14b** (5 mg) in a dichloromethane/hexane mixture (1 mL, v/v = 1:5) allowing for determination of the relative configuration as (S*,S*) (Figure 5). This was extended to all the series, considering the similarity of the ^1^H NMR spectra and of the retention factors observed in chromatography.

**Figure 6:**
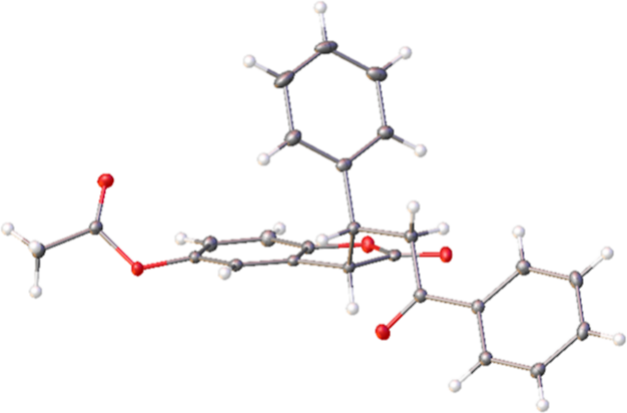
ORTEP diagram of **15d-1**.^22^ Crystals of **15d-1** were obtained by slow evaporation of a solution of **15d** (10 mg) in a diethyl ether/isopropanol mixture (1 mL, v/v = 1:4).

Although the enolate of 2-coumaranone **3a** is less nucleophilic than that of 3-isochromanone **2a**, it reacted successfully with chalcones **13** under the conditions of entry 6 from Table 2. Several substituted 2-coumaranones were reacted with chalcones bearing different substituents on both the aromatic rings as well, leading from good to high yields and from moderate to low diastereoselectivity (Table 4). Of note, 5-chloro-coumaranone **3c** required a higher temperature and longer reaction time (Table 4, entry 8). Only 5-nitro-coumaranone **3d** did not react at all, even at 80 °C, because of the decrease of its nucleophilicity (entries 9 and 10) by the electron-withdrawing and, therefore, anion-stabilizing properties of the nitro group (Hammett substituent constant σ_m_ = 0.71^23^). In this series, the separation of the diastereomers was more problematic and only in the case of **15d** did attempts at crystallization yield crystals suitable for X-ray analysis, depicted in Figure 6, allowing for the determination of the relative configuration as (S*,R*).

**Table 4:**
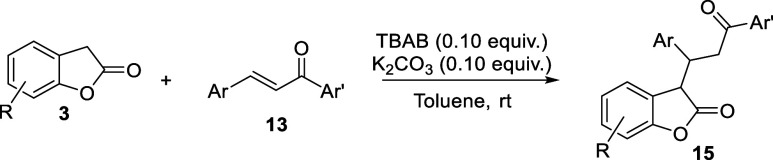
Analysis of the Scope with 2-Coumaranones **3** and Chalcones **13**

entry	lactones	chalcones	Ar	Ar′	time (h)	yield (%)^a^	d.r^b^
1	**3a**, R = H	**13a**	Ph	Ph	4	**15a**, 81	64/36
2	**3a**, H	**13b**	4-ClC_6_H_4_	Ph	5.5	**15b**, 83	53/47
3	**3a**, H	**13f**	4-MeOC_6_H_4_	Ph	5	**15c**, 87	54/46
4	**3b**, 5-OAc	**13a**	Ph	Ph	2.5	**15d**, 89	59/41
5	**3a**, H	**13i**	Ph	4-MeOC_6_H_4_	5	**15e**, 83	62/38
6	**3a**, H	**13d**	2-ClC_6_H_4_	Ph	8.5	**15f**, 81	54/46
7	**3a**, H	**13e**	4-FC_6_H_4_	Ph	6.5	**15g**, 79	64/36
8^c^	**3c**, 5-Cl	**13a**	Ph	Ph	36	**15h**, 75	63/37
9^d^	**3d**, 5-NO_2_	**13a**	Ph	Ph	24	-e	-
10^d^	**3d**, 5-NO_2_	**13f**	4-MeOC_6_H_4_	Ph	40	-e	-

a Isolated yield.

b Determined by ^1^H NMR on the crude material.

c Reaction temperature at 50 °C.

d Reaction temperature at 80 °C.

e Starting materials recovered.

## Conclusions

In this article, we explored the reactivity of isochroman-3-ones and 2-coumaranones as nucleophiles in Michael reactions. Based on the Mayr–Patz eq 1 and by following the kinetics of the reactions of the respective lactone enolates with a series of reference electrophiles (quinone methides and arylidenemalonates) with known electrophilicity *E*, the nucleophilicity parameters *N* (*s*_N_) of the corresponding lactone enolates **4** and **5** in DMSO were determined to be 25.39 (0.54) and 19.60 (0.75), respectively. Considering the higher Brønsted acidity of isochroman-3-one and 2-coumaranone in comparison to their noncyclic structurally analogous alkyl phenylacetates, along with their high level of nucleophilic reactivity, an efficient catalytic method for Michael additions was developed. In particular, series of substituted isochroman-3-ones and 2-coumaranones were synthesized and then shown to react with chalcones. In combination with the phase transfer catalyst tetra-*n*-butylammonium bromide (0.1 equiv), the low-cost Brønsted base K_2_CO_3_ (0.1 equiv) could be used in catalytic amounts to activate the lactones in toluene and, thus, widen the scope of this carbon–carbon bond-forming reaction. Based on these findings, further studies about the development of asymmetric versions of the investigated Michael additions and other applications in reactions with carbon-carbon and carbon-heteroatom bond formation are ongoing.

## Experimental Section

### General Information

Unless otherwise noted, all chemicals, reagents, and solvents for the performed reactions are commercially available. Isochroman-3-one was purchased from fluorochem, and substituted isochroman-3-ones were prepared according to literature procedures (see the Supporting Information for further details). All the reactions were monitored by TLC on precoated silica gel plates (0.25 mm) and visualized by fluorescence quenching at 254 nm. Flash chromatography was carried out using silica gel 60 (70–230 mesh, Merck, Darmstadt, Germany). Preparative TLC (PrepTLC) was carried out on silica gel plates (200 × 200 × 0.5 mm). The products were detected under UV light, extracted with ethyl acetate, and obtained after rotary evaporation. Yields are given for isolated products showing one spot on a TLC plate. The NMR spectra were recorded on Bruker DRX 600, 400, and 300 MHz spectrometers (600 MHz, ^1^H, 150 MHz, ^13^C; 400 MHz, ^1^H, 100.6 MHz; ^13^C, 300 MHz, ^1^H, 75.5 MHz, ^13^C, 250 MHz, ^1^H, 62.5 MHz, ^13^C). Internal reference was set to the residual solvent signals (δ_H_ 7.26 ppm, δ_C_ 77.16 ppm for CDCl_3_).^24^ The ^13^C{^1^H} NMR spectra were recorded under broad-band proton-decoupling and reported *C*_q_, CH, CH_2_, or CH_3_ assignments were based on additional heteronuclear single quantum coherence (HSQC) and heteronuclear multiple bond correlation (HMBC) experiments. ^1^H NMR and high-resolution mass spectrometry (HRMS) data are reported for all compounds. IR and ^13^C NMR data are given only for new compounds. The following abbreviations are used to indicate the multiplicity in NMR spectra: s—singlet, d—doublet, t—triplet, q—quartet, dd—doublet of doublets, m—multiplet, brs—broad signal. High-resolution mass spectra were acquired by the Salerno team using a Bruker SolariX XR Fourier transform ion cyclotron resonance mass spectrometer (Bruker Daltonik GmbH, Bremen, Germany) equipped with a 7T refrigerated actively shielded superconducting magnet. For ionization of the samples electrospray ionization (ESI) or matrix-assisted laser desorption/ionization (MALDI) was applied. In Munich, HRMS was performed by using a Thermo Finnigan LTQ FT (ESI) or a Thermo Finnigan MAT 95 instrument (EI). IR spectra were recorded on a IR Bruker Vertex 70v spectrometer.

### Kinetics

The kinetics of the reactions of the lactone enolates with the reference electrophiles **6** were followed by UV/vis spectroscopy (Applied Photophysics SX20 stopped-flow spectrophotometer). A constant temperature (20.0 ± 0.2 °C) was maintained through the use of a circulating bath cryostat. All solutions were freshly prepared under an atmosphere of dry argon by using dry DMSO (over molecular sieves, Acros Organics). Solutions of sodium 3-oxoisochroman-4-ide (**4**) in DMSO were prepared by deprotonation of 3-isochromanone (**2a**) with sodium hydride. Solutions of 2-oxo-2,3-dihydrobenzofuran-3-ide (**5**) in DMSO were prepared by deprotonation of 2-coumaranone (**3a**) with DBU (2.2 equiv.).

The kinetic measurements were initiated by mixing equal volumes of DMSO solutions of the nucleophiles and electrophiles. Selected reactions of **4** with the electrophiles **6** were measured with and without added crown ether (15-crown-5, 1.1 equiv relative to the concentration of **4**). In general, nucleophile concentrations were at least ten times higher than electrophile concentrations to achieve pseudo-first-order kinetics. Only for the reaction of **4** with **6a** (Table S1), the kinetic experiments were carried out by using the electrophile **6a** in excess (>10 equiv). The first-order rate constants *k*_obs_ (s^–1^) could be obtained from the decay of the absorbance at or close to the absorption maximum of the reaction partner used in lower concentration by least-squares fitting of the equation *A*_*t*_ = *A*_0_ exp(−*k*_obs_*t*) + *C* to the exponential absorption decay curve. Plots of *k*_obs_ (s^–1^) versus the nucleophile concentration (or electrophile concentration for the reaction of **4** with **6a**) gave the second-order rate constants *k*_2_ (M^–1^ s^–1^) as slopes of the linear correlations.

### 4-((3,5-Di-*tert*-butyl-4-hydroxyphenyl)(julolidin-9-yl)-λ^3^-methyl)-4λ^3^-isochroman-3-one (**7a**)

#### Procedure A

3-Isochromanone **2a** (31.2 mg, 0.211 mmol), *p*QM **6b** (77.5 mg, 0.199 mmol), and potassium *tert*-butoxide (30.3 mg, 0.270 mmol) were mixed in DMSO (13 mL) and stirred for 10 min. Then, the reaction mixture was poured on 0.5% aq. acetic acid (50 mL). The phases were separated, and the aqueous phase was extracted with ethyl acetate (3 × 40 mL). The combined organic phases were washed with brine (2 × 40 mL), dried over sodium sulfate, and filtered. Partial crystallization by slow diffusion of pentane into a dichloromethane solution of the diastereomeric mixture of **7a** at 4 °C (fridge) delivered a small amount of diastereomerically pure cubic crystals of **7a** (13.9 mg, yield: 13%), which were characterized by X-ray crystallography (bv020).^22^ A solution of the diastereomerically pure crystals in CDCl_3_ was used for the NMR spectroscopic characterization of **7a**-major.

#### Procedure B

3-Isochromanone **2a** (42.5 mg, 0.287 mmol) and sodium hydride (7.1 mg, 95% purity, 0.281 mmol) were dissolved in DMSO (5 mL) under an argon atmosphere at room temperature and stirred for 45 min. Then, a solution of *p*QM **6b** (101 mg, 0.259 mmol) in a DMSO/dichloromethane mixture (4.5 mL/2.5 mL) was added. The reaction mixture was stirred for 1 h and then poured on 0.5% aq. acetic acid (50 mL). The layers were separated, and the aq. phase was extracted with ethyl acetate (3 × 20 mL). The combined organic phases were washed with brine (2 × 20 mL) and dried over MgSO_4_. After filtration, volatiles were evaporated to obtain the crude product (146 mg). Purification by column chromatography (silica gel, *n*-pentane/ethyl acetate = 7:1) furnished **7a** (92.7 mg, yield: 67%); a mixture of diastereomers, d.r. 1:1.6. NMR spectra of this sample were used to assign ^1^H and ^13^C resonances of **7a**-minor.

**7a**-major: ^1^H NMR (400 MHz, CDCl_3_, δ) 7.23–7.19 (m, 1H), 7.10–7.07 (m, 1H), 7.04–7.02 (m, 1H), 6.84 (s, 2H), 6.72 (s, 2H), 6.61–6.59 (m, 1H), 5.05 (s, 1H), 4.93 (d, *J* = 14.3 Hz, 1H), 4.54 (d, *J* = 14.1 Hz, 1H), 4.37 (d, *J* = 6.8 Hz, 1H), 4.30 (d, *J* = 7.0 Hz, 1H), 3.10 (dd, *J* = 6.3 Hz, 5.0 Hz, 4H), 2.79–2.67 (m, 4H), 2.00–1.93 (m, 4H), 1.28 (s, 18H). ^13^C{^1^H} NMR (101 MHz, CDCl_3_, δ) 172.4 (C_q_), 152.8 (C_q_), 142.0 (C_q_), 135.4 (C_q_), 133.6 (C_q_), 131.9 (C_q_), 130.5 (C_q_), 129.0 (CH), 127.7 (CH), 127.6 (C_q_), 127.3 (CH), 127.2 (CH), 126.0 (CH), 124.0 (C_q_), 121.5 (C_q_), 69.9 (CH_2_), 54.4 (CH, Ar_2_CH), 53.4 (CH), 50.2 (CH_2_), 34.3 (C_q_), 30.3 (CH_3_), 27.8 (CH_2_), 22.3 (CH_2_). HRMS (ESI): *m*/*z* calcd for C_36_H_44_NO_3_^+^ (M + H^+^) 538.3316; found: 538.3305.

**7a**-minor: ^1^H NMR (400 MHz, CDCl_3_, δ) 6.57 (s, 2H), 5.08 (s, 1H), 4.87 (d, *J* = 14.2 Hz, 1H), 4.23 (d, *J* = 5.6 Hz, 1H), 2.64–2.61 (m, 4H), 1.37 (s, 18H); further resonances could not unequivocally be assigned because of superimposition with resonances of **7a**-major. ^13^C{^1^H} NMR (101 MHz, CDCl_3_, δ) 172.0, 152.8, 142.0, 135.6, 134.1, 132.2, 131.4, 128.9, 127.8, 127.7, 127.6, 127.2, 125.7, 123.7, 121.3, 69.9, 56.2, 52.9, 50.2, 34.5, 30.4, 27.8, 22.3.

### 4-((3,5-Di-*tert*-butyl-4-hydroxyphenyl)(4-(dimethylamino)phenyl)-λ^3^-methyl)-4λ^3^-isochroman-3-one (**7b**)

The product was obtained from 3-isochromanone **2a** (29.6 mg, 0.200 mmol), *p*QM **6c** (65.3 mg, 0.193 mmol), and potassium *tert*-butoxide (31.4 mg, 0.280 mmol) by analogously following *Procedure A* (cf. synthesis of **7a**). Purification of the crude product by column chromatography (silica gel, pentane/ethyl acetate = 7:1) furnished **7b** (17.1 mg, yield: 18%); a mixture of diastereomers, d.r. 1:2.

^1^H NMR (400 MHz, CDCl_3_, δ) 7.30–7.28 (m, 4H), 7.25–7.17 (m, 4H), 7.11–7.02 (m, 9H), 6.78–6.76 (m, 5H), 6.72–6.70 (m, 4H), 6.62–6.58 (m, 4H), 5.10 (s, 1 H^minor^), 5.07 (s, 2 H^major^), 4.94 (d, *J* = 14.2 Hz, 2 H^major^), 4.89 (d, *J* = 14.3 Hz, 1 H^minor^), 4.57 (d, *J* = 14.2 Hz, 1 H^major^), 4.46 (d, *J* = 6.2 Hz, 1 H^minor^), 4.43 (br s, 4 H^major^), 4.34 (d, *J* = 6.1 Hz, 1 H^minor^), 4.39 (d, *J* = 14.0 Hz, 1 H^minor^), 2.94 (s, 12 H^major^), 2.91 (s, 6 H^minor^), 1.37 (s, 18 H^minor^), 1.29 (s, 38 H^major^); reported integrals refer to 1H (=1.0) of the minor diastereomer. ^13^C{^1^H} NMR (101 MHz, CDCl_3_, δ) 172.2 (C_q_^major^), 171.8 (C_q_^minor^), 152.9 (C_q_^major+minor^), 149.65 (C_q_^minor^), 149.61 (C_q_^major^), 135.7 (C_q_^minor^), 135.5 (C_q_^major^), 134.1 (C_q_^minor^), 133.6 (C_q_^major^), 132.0 (C_q_^minor^), 131.8 (C_q_^major^), 131.2 (C_q_^minor^), 130.5 (C_q_^major^), 129.7 (CH^minor^), 129.4 (CH^major^), 128.91 (CH^major^), 128.88 (CH^minor^), 128.84 (C_q_^minor^), 128.4 (C_q_^major^), 128.0 (CH^minor^), 127.9 (CH^major^), 127.29 (CH^minor^), 127.27 (CH^major^), 126.0 (CH^major^), 125.7 (CH^minor^), 124.1 (CH^major^), 123.9 (CH^minor^), 112.7 (CH^major^), 112.5 (CH^minor^), 69.90 (CH_2_^major^), 69.88 (CH_2_^minor^), 55.9 (CH, lactone-CH^minor^ or Ar_2_CH^minor^), 54.4 (CH, lactone-CH^major^ or Ar_2_CH^major^), 53.2 (CH, lactone-CH^major^ or Ar_2_CH^major^), 52.6 (CH, (CH, lactone-CH^minor^ or Ar_2_CH^minor^)), 40.75 (CH_3_^minor^), 40.74 (CH_3_^major^), 34.5 (C_q_^minor^), 34.3 (C_q_^major^), 30.4 (CH_3_^minor^), 30.3 (CH_3_^major^). HRMS (ESI): *m*/*z* calcd for C_32_H_40_NO_3_^+^ (M + H^+^): 486.3003; found: 486.2995.

### 4-((4-Hydroxy-3,5-dimethoxyphenyl)(4-methoxyphenyl)-λ^3^-methyl)-4λ^3^-isochroman-3-one (**7c**)

The product was obtained from 3-isochromanone **2a** (38.4 mg, 0.259 mmol), sodium hydride (7.1 mg, 95% purity, 0.281 mmol), and *p*QM **6d** (61.0 mg, 0.224 mmol) by analogously following *Procedure B* (cf. synthesis of **7a**). Purification of the crude product by column chromatography (silica gel, *n*-pentane/ethyl acetate = 1:1) furnished a yellow oil (74.8 mg). The content of residual ethyl acetate in the sample was determined from the integrals in the ^1^H NMR spectrum (7.9 mg) and the yield of **7c** calculated (66.8 mg, yield: 71%); a mixture of diastereomers, d.r. 1:1.3.

^1^H NMR (599 MHz, CDCl_3_, δ) 7.29–7.24 (m, 6.6H), 7.16–7.13 (m, 2.4H), 7.11–7.08 (m, 2.4H), 7.04–7.03 (m, 2 H^minor^), 6.88–6.85 (m, 2.7 H^major^), 6.77–6.74 (m, 2 H^minor^), 6.73–6.71 (m, 2.3H), 6.52 (s, 2 H^minor^), 6.27 (s, 2.6 H^major^), 5.04 (d, *J* = 14.2 Hz, 1.3 H^major^), 5.02 (d, *J* = 14.1 Hz, 1 H^minor^), 4.84 (d, *J* = 14.3 Hz, 1.3 H^major^), 4.77 (d, *J* = 14.3 Hz, 1 H^minor^), 4.51–4.49 (m, 2.3 H^major+minor^), 4.39–4.37 (m, 2.3 H^major+minor^), 3.81 (s, 6 H^minor^), 3.80 (s, 4.0 H^major^), 3.77 (s, 3 H^minor^), 3.69 (s, 7.9 H^major^); reported integrals refer to 1H (=1.0) of the minor diastereomer. ^13^C{^1^H} NMR (151 MHz, CDCl_3_, δ) 171.5 (C_q_^major^), 171.3 (C_q_^minor^), 158.8 (C_q_^major^), 158.7 (C_q_^minor^), 147.0 (C_q_^minor^), 146.8 (C_q_^major^), 134.0 (C_q_^minor^), 133.8 (C_q_^major^), 133.50 (C_q_^major^), 133.48 (C_q_^minor^), 132.5 (C_q_^minor^), 132.3 (C_q_^major^), 131.65 (C_q_^minor^), 131.61 (C_q_^major^), 131.5 (C_q_^minor^), 131.3 (C_q_^major^), 129.9 (CH^minor^), 129.6 (CH^major^), 128.73 (CH^minor^), 128.71 (CH^major^), 128.3 (CH^major^), 128.2 (CH^minor^), 127.61 (CH^minor^), 127.57 (CH^major^), 124.33 (CH^major^), 124.26 (CH^minor^), 114.0 (CH^major^), 113.8 (CH^minor^), 105.8 (CH^major^), 105.7 (CH^minor^), 70.1 (CH_2_^minor^), 70.0 (CH_2_^major^), 56.5 (CH_3_, 2 × OMe^minor^), 56.3 (CH_3_, 2 × OMe^major^), 55.36 (CH_3_, OMe^major^), 55.34 (CH_3_, OMe^minor^), 54.7 (CH^minor^), 54.4 (CH^major^), 52.3 (CH^major^), 52.1 (CH^minor^); additional resonances at δ 171.6, 60.6, 21.2, and 14.3 are caused by residual ethyl acetate, which could not be removed from the sample. HRMS (EI): *m*/*z* calcd for C_25_H_22_O_6_^•+^ [M – H_2_]^•+^: 418.1411; found: 418.1415.

### 3-((3,5-Di-*tert*-butyl-4-hydroxyphenyl)(4-methoxyphenyl)methyl)benzofuran-2(3H)-one (**8**)

Under an atmosphere of dry N_2_, 2-coumaranone **3a** (100 mg, 0.746 mmol) and potassium carbonate (206 mg, 1.49 mmol) were mixed in acetonitrile (2 mL). Then, *p*QM **6e** (242 mg, 0.746 mmol, solution in 8 mL MeCN) was added dropwise. The reaction mixture was stirred for 1.5 h at room temperature. The resulting purple solution was filtered and neutralized with sat. aq NH_4_Cl solution (ca. 25 mL). The aq phase was extracted with ethyl acetate (3 × 25 mL), and the combined organic phases were dried (MgSO_4_) and filtered. Evaporation of volatiles and drying under vacuum yielded a foamy, orange solid (360 mg). The content of residual ethyl acetate in the sample was determined from the integrals in the ^1^H NMR spectrum (25 mg) and the yield of **8** calculated (335 mg, yield: 98%); a mixture of diastereomers, d.r. 1:1.6.

^1^H NMR (400 MHz, CDCl_3_, δ) 7.24–7.19 (m, 5.2 H^major+minor^), 7.02–6.96 (m, 9.7 H^major+minor^), 6.90–6.87 (m, 3.3 H^major^), 6.77–6.72 (m, 6.3 H^major+minor^), 6.68–6.63 (m, 2.0 H^minor^), 5.15 (s, 1 H^minor^), 5.06 (s, 1.6 H^major^), 4.81 (d, *J* = 4.8 Hz, 1 H^minor^), 4.78 (d, *J* = 5.3 Hz, 1.6 H^major^), 4.52–4.49 (m, 2.6 H^major+minor^), 3.82 (s, 4.7 H^major^), 3.76 (s, 3.0 H^minor^), 1.36 (s, 18.0 H^minor^), 1.26 (s, 29.4 H^major^); reported integrals refer to 1H (=1.0) of the minor diastereomer. ^13^C{^1^H} NMR (101 MHz, CDCl_3_, δ) 175.7 (C_q_^minor^), 175.6 (C_q_^major^), 158.7 (C_q_^minor^), 158.5 (C_q_^major^), 154.18 (C_q_^minor^), 154.16 (C_q_^major^), 153.0 (C_q_^major^), 152.8 (C_q_^minor^), 135.8 (C_q_^minor^), 135.6 (C_q_^major^), 133.1 (C_q_^major^), 131.6 (C_q_^minor^), 130.7 (CH^minor^), 130.4 (CH^major^), 129.6 (CH^major^), 129.2 (CH^minor^ or C_q_^minor^), 129.1 (CH^minor^ or C_q_^minor^), 129.0 (C_q_^major^), 126.50 (C_q_^major^), 126.47 (C_q_^minor^), 126.1 (CH^major^), 125.5 (CH^major^), 125.4 (CH^minor^), 125.2 (CH^minor^), 123.81 (CH^major^), 123.75 (CH^minor^), 113.9 (CH^major^), 113.8 (CH^minor^), 110.71 (CH^minor^), 110.69 (CH^major^), 55.4 (CH_3_^major^), 55.3 (CH_3_^minor^), 51.6 (CH, C–H^minor^), 51.4 (CH, C–H^major^), 49.54 (CH, C–H^minor^), 49.52 (CH, C–H^major^), 34.6 (C_q_^minor^), 34.3 (C_q_^major^), 30.4 (CH_3_^minor^), 30.3 (CH_3_^major^). HRMS (EI): *m*/*z* calcd for C_30_H_32_O_4_^•+^ [M – H_2_]^•+^: 456.2295; found: 456.2292.

### Dimethyl 3,3′-(2-oxo-2,3-dihydrobenzofuran-3,3-diyl)dipropionate (**10**)

#### Procedure C

Under an atmosphere of dry N_2_, 2-coumaranone **3a** (100 mg, 0.746 mmol) was added to a solution of potassium carbonate (206 mg, 1.49 mmol) in acetonitrile (5 mL). Subsequently, methyl acrylate (**9**) (67 μL, 0.739 mmol) was added dropwise, and the reaction mixture was stirred at room temperature for 30 h (TLC showed complete consumption of **3a**). Then, the mixture was washed with sat. aq NH_4_Cl solution, and the aqueous phase was extracted with ethyl acetate (3 × 30 mL). The combined organic phases were dried over MgSO_4_ and filtered. Volatiles were removed under vacuum, which left a colorful oily crude product. Purification by column chromatography (silica gel, hexane/CH_2_Cl_2_ = 5:1) gave **10** (58.2 mg, yield: 51%) as a gray-green oil.

^1^H NMR (599 MHz, CDCl_3_, δ) 7.33–7.31 (m, 1H), 7.20–7.12 (m, 3H), 3.55 (s, 6H), 2.32–2.15 (m, 6H), 1.97 (ddd, *J* = 16.0, 10.7, 5.0 Hz, 2H). ^13^C{^1^H} NMR (151 MHz, CDCl_3_, δ) 178.7 (C_q_), 172.6 (C_q_), 153.4 (C_q_), 129.6 (CH), 128.1 (C_q_), 124.8 (CH), 123.8 (CH), 111.2 (CH), 51.9 (CH_3_), 50.9 (C_q_), 33.0 (CH_2_), 29.4 (CH_2_). IR (ATR, neat): 2953, 1798, 1733, 1477, 1461, 1435, 1226, 1196, 1170, 1128, 1089, 1032, 868, 753 cm^–1^. HRMS (EI): *m*/*z* calcd for C_16_H_18_O_6_^•+^ [M^•+^]: 306.1098; found: 306.1097.

### 4,4′-(2-Oxo-2,3-dihydrobenzofuran-3,3-diyl)bis(butan-2-one) (**12**)

The product was obtained from 2-coumaranone **3a** (100 mg, 0.746 mmol) and methyl vinyl ketone (**11**) (52 mg, 0.742 mmol) by analogously following *Procedure C* (cf. synthesis of **10**). Purification by column chromatography (silica gel, hexane/CH_2_Cl_2_ = 5:1) gave **12** (88.7 mg, yield: 87%) as a yellow-brown oil.

^1^H NMR (599 MHz, CDCl_3_, δ) 7.34–7.31 (m, 1H), 7.18–7.10 (m, 3H), 2.32–2.28 (m, 2H), 2.26–2.13 (m, 4H), 2.10–2.00 (m, 2H), 1.98 (s, 6H). ^13^C{^1^H} NMR (151 MHz, CDCl_3_, δ) 206.9 (C_q_, ketone C=O), 179.1 (C_q_, lactone C=O), 153.1 (C_q_), 129.4 (CH), 128.8 (C_q_), 124.9 (CH), 123.8 (CH), 111.0 (CH), 50.4 (C_q_), 38.4 (CH_2_), 31.6 (CH_2_), 30.1 (CH_3_). IR (ATR, neat): 2927, 1798, 1712, 1478, 1461, 1362, 1225, 1164, 1129, 1038, 880, 754, 733 cm^–1^. HRMS (EI): *m*/*z* calcd for C_16_H_18_O_4_^•+^ [M^•+^]: 274.1200; found: 274.1199.

### K_2_CO_3_-Promoted Michael Reaction of **2a** with Chalcone **13a** (Scheme 2d)

Isochroman-3-one **2** (30 mg, 0.20 mmol), potassium carbonate (28 mg, 0.20 mmol), and chalcone **13a** (46 mg, 0.22 mmol) were stirred in MeCN (0.4 mL) for 30 h at room temperature. Purification was carried out directly by chromatography on a silica gel column with eluent hexane/ethyl acetate (95/5 → 70/30) to elute **14a** (50 mg, 70% yield) as a mixture of diastereomers.

### Catalytic Michael Reactions of **2** or **3** with Chalcones

#### Procedure D

Isochroman-3-one **2** or 3-coumaranone **3** (0.135 mmol), potassium carbonate (10 mol %), tetrabutyl ammonium bromide (TBAB, 10 mol %), and chalcone (0.148 mmol, 1.1 equiv) were stirred in toluene (0.2 mL) for 5 h at room temperature. Purification was carried out by chromatography on a short pad of silica gel column with eluent hexane/ethyl acetate (95/5 → 70/30) to elute the mixture of diastereomers. Where possible, the diastereomers were separated by PrepTLC using hexane/ethyl acetate (90/10) as the eluent.

*4-(3-Oxo-1,3-diphenylpropyl)isochroman-3-one* (**14a**) was purified by flash chromatography (silica gel, hexane/ethyl acetate = 8:2), isolated in a total yield of 97%, and analyzed by HRMS. Subsequently, PrepTLC (silica gel, hexane/ethyl acetate = 85:15) was used to separate the diastereomers of **14b** (dr = 50/50). **HRMS** (MALDI-FT ICR): *m*/*z* calcd. for C_24_H_21_O_3_ [M + H]^+^: 357.1485; found: 357.1475.

The reaction was also scaled up to 1 mmol scale of isochroman-3-one **2a**. Product **14a** was isolated as a mixture of diastereomers after flash chromatographic purification on silica gel (hexane/ethyl acetate = 8:2) in a total yield of 89% (317 mg).

(R*)-4-((R*)-3-Oxo-1,3-diphenylpropyl)isochroman-3-one (**14a-1**): white solid (23 mg); mp 143–145 °C. ^1^H NMR (400 MHz, CDCl_3_, δ) 8.01 (dd, *J* = 8.4, 1.3 Hz, 2H), 7.65 (d, *J* = 6.1 Hz, 2H), 7.56 (t, *J* = 7.5 Hz, 2H), 7.49 (t, *J* = 7.6 Hz, 1H), 7.37–7.28 (m, 2H), 7.24 (t, *J* = 7.3 Hz, 2H), 6.99 (d, *J* = 6.9 Hz, 2H), 6.95 (d, *J* = 7.7 Hz, 1H), 4.76 (d, *J* = 14.4 Hz, 1H), 4.40 (dd, *J* = 18.3, 9.4 Hz, 1H), 4.22 (d, *J* = 3.8 Hz, 1H), 4.03–3.94 (m, 1H), 3.60 (d, *J* = 15.3 Hz, 1H), 3.54 (dd, *J* = 18.3, 4.4 Hz, 1H). ^13^C{^1^H} NMR (151 MHz, CDCl_3_, δ) 200.0, 172.5, 140.6, 138.5, 135.2, 134.7, 132.4, 131.1, 130.09, 130.06, 129.9, 129.7, 129.5, 129.1, 128.8, 124.7, 71.1, 51.0, 48.4, 42.9. IR (neat): 2926, 1727, 1679, 1595 cm^–1^.

(R*)-4-((S*)-3-Oxo-1,3-diphenylpropyl)isochroman-3-one (**14a-2**): white solid (23 mg); mp 137–139 °C. ^1^H NMR (400 MHz, CDCl_3_, δ) 8.03 (dd, *J* = 7.1, 1.3 Hz, 2H), 7.50 (tt, *J* = 7.4, 1.3 Hz, 1H), 7.39 (t, *J* = 7.8 Hz, 1H), 7.24–7.09 (m, 4H), 7.08 (d, *J* = 7.3 Hz, 1H), 7.00 (t, *J* = 7.6 Hz, 1H), 6.90 (dd, *J* = 7.4, 2.1 Hz, 2H), 6.42 (d, *J* = 7.7 Hz, 1H), 5.18 (d, *J* = 14.1 Hz, 1H), 5.01 (d, *J* = 14.1 Hz, 1H), 3.91 (ddd, *J* = 9.1, 7.2, 5.2 Hz, 1H), 3.87 (d, *J* = 9.1 Hz, 1H), 3.79 (dd, *J* = 17.8, 7.3 Hz, 1H), 3.42 (dd, *J* = 17.7, 5.3 Hz, 1H). ^13^C{^1^H} NMR (101 MHz, CDCl_3_, δ) 197.8, 172.4, 140.7, 136.8, 133.3, 132.6, 131.7, 128.7, 128.6, 128.5, 128.3, 128.1, 127.7, 127.48, 127.43, 124.5, 69.6, 52.7, 42.6, 42.1. IR (neat): 2994, 1731, 1685, 1597 cm^–1^.

*4-(1-(4-Chlorophenyl)-3-oxo-3-phenylpropyl)isochroman-3-one* (**14b**) was purified by flash chromatography (silica gel, hexane/ethyl acetate = 8:2), isolated in a total yield of 97%, and analyzed by HRMS. Subsequently, PrepTLC (silica gel, hexane/ethyl acetate = 85:15) was used to separate the diastereomers of **14b** (dr = 50/50).

HRMS (MALDI-FT ICR): *m*/*z* calcd. for C_24_H_20_ClO_3_ [M + H]^+^: 391.1095; found: 391.1091.

(R*)-4-((R*)-1-(4-Chlorophenyl)-2-oxo-2-phenylethyl)isochroman-3-one (**14b-1**): white solid (26 mg); mp 174–176 °C. ^1^H NMR (400 MHz, CDCl_3_, δ) 8.11 (dd, *J* = 7.1, 1.5 Hz, 2H), 7.65 (d, *J* = 6.1 Hz, 2H), 7.56 (t, *J* = 7.5 Hz, 2H), 7.49 (t, *J* = 7.6 Hz, 1H), 7.36–7.29 (m, 2H), 7.24 (t, *J* = 7.3 Hz, 2H), 6.99 (d, *J* = 6.9 Hz, 2H), 6.95 (d, *J* = 7.7 Hz, 1H), 4.76 (d, *J* = 14.4 Hz, 1H), 4.40 (dd, *J* = 18.3, 9.4 Hz, 1H), 4.22 (d, *J* = 3.8 Hz, 1H), 4.04–3.94 (m, 1H), 3.60 (d, *J* = 15.3 Hz, 1H), 3.54 (dd, *J* = 18.3, 4.4 Hz, 1H). ^13^C{^1^H} NMR (63 MHz, CDCl_3_, δ) 198.1, 170.7, 137.6, 136.7, 133.5, 133.3, 133.2, 130.6, 129.4, 128.6, 128.0, 127.4, 123.4, 69.7, 49.3, 46.2, 41.3. IR (neat): 2925, 1744, 1685, 1590 cm^–1^.

(R*)-4-((S*)-1-(4-Chlorophenyl)-2-oxo-2-phenylethyl)isochroman-3-one (**14b-2**): white solid (26 mg); mp 164–166 °C. ^1^H NMR (400 MHz, CDCl_3_, δ) 7.95 (dd, *J* = 7.2, 1.5 Hz, 2H), 7.57 (t, *J* = 7.3 Hz, 1H), 7.46 (t, *J* = 7.4 Hz, 2H), 7.25 (t, *J* = 7.5 Hz, 1H), 7.18 (d, *J* = 6.1 Hz, 2H), 7.10 (t, *J* = 7.4 Hz, 1H), 6.91 (d, *J* = 8.4 Hz, 2H), 6.48 (d, *J* = 7.6 Hz, 2H), 5.36 (d, *J* = 14.1 Hz, 1H), 5.13 (d, *J* = 14.3 Hz, 1H), 3.97–3.79 (m, 3H), 3.47 (dd, *J* = 17.6, 5.6 Hz, 1H). ^13^C{^1^H} NMR (101 MHz, CDCl_3_, δ) 197.4, 171.9, 139.3, 136.7, 133.4, 133.2, 132.4, 131.6, 129.7, 128.7, 128.5, 128.0, 127.9, 127.6, 127.1, 124.7, 69.6, 52.6, 41.9, 36.2. IR (neat): 2924, 1725, 1685, 1582 cm^–1^.

*4-(1-(2-Nitrophenyl)-3-oxo-3-phenylpropyl)isochroman-3-one* (**14c**) was purified by flash chromatography (silica gel, hexane/ethyl acetate = 7:3) and isolated as a white solid (36 mg, yield: 68%), a mixture of diastereomers (dr = 56/44). ^1^H NMR (400 MHz, CDCl_3_, δ) 7.99^major^ (dd, *J* = 7.1, 1.4 Hz, 2H), 7.94^major^ (dd, *J* = 7.1 Hz, 1.4 Hz, 2H), 7.77–7.70 (m, 3H), 7.68 (d, *J* = 3.9 Hz, 3H), 7.60 (d, *J* = 7.4 Hz, 2H), 7.48 (t, *J* = 9.3 Hz, 5H), 7.45–7.40 (m, 4H), 7.29 (d, *J* = 5.0 Hz, 2H), 7.15 (d, *J* = 7.3 Hz, 1H), 7.04 (dt, *J* = 8.4, 4.4 Hz, 1H), 6.37^minor^ (d, *J* = 7.6 Hz, 1H), 6.02^minor^ (d, *J* = 14.2 Hz, 1H), 5.35^major^ (d, *J* = 14.2 Hz, 1H), 5.08^minor^ (d, *J* = 14.2 Hz, 1H), 5.01–4.83 (m, 2H), 4.26^minor^ (d, *J* = 7.6 Hz, 1H), 4.05–3.90 (m, 3H), 3.72^major^ (dd, *J* = 18.4, 7.3 Hz, 1H), 3.54^minor^ (dd, *J* = 18.0, 6.7 Hz, 1H), 3.45 (d, *J* = 2.6 Hz, 1H), ^13^C{^1^H} NMR (63 MHz, CDCl_3_, δ) 196.6, 196.5, 171.3, 170.3, 150.4, 150.2, 136.3, 136.2, 136.0, 134.4, 133.3, 132.7, 132.7, 131.9, 131.8, 131.0, 129.4, 129.2, 128.7, 128.6, 128.5, 128.1, 128.0, 127.9, 127.8, 127.7, 127.2, 124.8, 124.5, 124.4, 69.9, 69.6, 52.6, 50.3, 42.2, 42.0, 37.2, 33.8. HRMS (MALDI-FT ICR): *m*/*z* calcd. for C_26_H_24_NaO_5_ [M + Na]^+^: 439.1516; found: 439.1525.

*4-(1-(2-Chlorophenyl)-3-oxo-3-phenylpropyl)isochroman-3-one* (**14d**). The reaction mixture was heated at 60 °C (oil bath). The crude product was purified by flash chromatography (silica gel, hexane/ethyl acetate = 85:15) and isolated as a white solid (32 mg, yield: 62%), a mixture of diastereomers (dr = 60/40). ^1^H NMR (400 MHz, CDCl_3_, δ) 8.03^minor^ (d, *J* = 4.4 Hz, 2H), 8.02^major^ (d, *J* = 4.2 Hz, 2H), 7.65–7.56 (m, 2H), 7.55–7.48 (m, 4H), 7.39 (d, *J* = 7.3 Hz, 2H), 7.27 (td, *J* = 16.1, 8.1 Hz, 6H), 7.19 (d, *J* = 6.8 Hz, 1H), 7.09–7.01 (m, 2H), 6.36^minor^ (d, *J* = 7.6 Hz, 1H), 6.03^minor^ (d, *J* = 14.1 Hz, 1H), 5.34^minor^ (d, *J* = 14.2 Hz, 1H), 4.95^minor^ (d, *J* = 14.4 Hz, 1H), 4.75–4.66^major^ (m, 1H), 4.40 (d, *J* = 14.6 Hz, 1H), 4.25 (d, *J* = 5.6 Hz, 1H), 4.15 (dd, *J* = 18.1, 8.2 Hz, 1H), 4.03 (dd, *J* = 18.3, 6.7 Hz, 1H), 3.96 (d, *J* = 10.9 Hz, 1H), 3.63^major^ (dd, *J* = 18.3, 6.0 Hz, 1H), 3.50^minor^ (dd, *J* = 18.1, 5.5 Hz, 1H). ^13^C{^1^H} NMR (101 MHz, CDCl_3_, δ) 197.6, 197.4, 171.9, 171.1, 138.8, 137.6, 136.8, 136.7, 135.1, 134.9, 133.37, 133.32, 132.9, 132.5, 132.0, 130.8, 129.9, 129.7, 129.1, 128.9, 128.7, 128.6, 128.6, 128.3, 128.1, 128.0, 127.8, 127.8, 127.7, 127.5, 127.4, 127.1, 124.5, 124.0, 69.9, 52.6, 50.3, 42.3, 42.0, 40.4, 36.8. HRMS (MALDI-FT ICR): *m*/*z* calcd. for C_24_H_20_ClO_3_ [M + H]^+^: 391.1095; found: 391.1072.

*4-(1-(4-Fluorophenyl)-3-oxo-3-phenylpropyl)isochroman-3-one* (**14e**) was purified by flash chromatography (silica gel, hexane/ethyl acetate = 9:1), isolated in a total yield of 91%, and analyzed by HRMS. Subsequently, PrepTLC (silica gel, hexane/ethyl acetate = 92:8) was used to separate the diastereomers (dr = 53/47).

HRMS (MALDI-FT ICR): *m*/*z* calcd. for C_24_H_19_FO_3_K [M + K]^+^: 413.0950; found: 413.0982.

(R*)-4-((R*)-1-(4-fluorophenyl)-3-oxo-3-phenylpropyl)isochroman-3-one (**14e-1**): white solid (24 mg); mp 137–139 °C. ^1^H NMR (400 MHz, CDCl_3_, δ) 8.02 (dd, *J* = 7.2, 1.4 Hz, 2H), 7.63 (tt, *J* = 7.4, 1.4 Hz, 1H), 7.52 (t, *J* = 7.6 Hz, 2H), 7.30 (dd, *J* = 7.5, 1.2 Hz, 1H), 7.23 (d, *J* = 6.5 Hz, 1H), 7.15 (t, *J* = 7.5 Hz, 1H), 7.01–6.93 (m, 4H), 6.54 (d, *J* = 6.4 Hz, 1H), 5.37 (d, *J* = 14.2 Hz, 1H), 5.17 (d, *J* = 14.3 Hz, 1H), 4.07–4.00 (m, 1H), 3.93 (dd, *J* = 7.1, 6.5 Hz, 1H), 3.87 (d, *J* = 7.1 Hz, 1H), 3.54 (dd, *J* = 17.8, 5.9 Hz, 1H). ^13^C{^1^H} NMR (101 MHz, CDCl_3_, δ) 197.5, 172.0, 161.8 (d, *J*_C,F_ = 246.4 Hz), 136.5 (d, *J*_C,F_ = 13.2 Hz), 133.2, 132.3, 131.4, 129.7 (d, *J*_C,F_ = 7.9 Hz), 128.5, 128.4, 128.0, 127.7, 127.4, 124.5, 115.5, 115.2, 69.5, 52.6, 42.1, 41.6. ^19^F{^1^H} NMR (376 MHz, CDCl_3_, δ) −114.21. IR (neat): 2924, 1725, 1686, 1597 cm^–1^.

(R*)-4-((S*)-1-(4-Fluorophenyl)-3-oxo-3-phenylpropyl)isochroman-3-one (**14e-2**): white solid (22 mg); mp 134–136 °C. ^1^H NMR (400 MHz, CDCl_3_, δ) 8.12 (dd, *J* = 7.1, 1.3 Hz, 2H), 7.64 (t, *J* = 7.6 Hz, 2H), 7.56 (t, *J* = 7.6 Hz, 2H), 7.49 (t, *J* = 7.5 Hz, 1H), 7.33 (d, *J* = 7.3 Hz, 1H), 7.01–6.92 (m, 5H), 4.84 (d, *J* = 14.5 Hz, 1H), 4.34 (dd, *J* = 18.2, 9.1 Hz, 1H), 4.19 (d, *J* = 3.9 Hz, 1H), 3.98 (dt, *J* = 8.9, 4.4 Hz, 1H), 3.79 (d, *J* = 14.5 Hz, 1H), 3.52 (dd, *J* = 18.2, 4.8 Hz, 1H). ^13^C{^1^H} NMR (101 MHz, CDCl_3_, δ) 198.3, 170.9, 162.3 (d, *J*_C,F_ = 246.7 Hz), 137.0, 135.0, 133.5, 133.4, 130.8, 129.8 (d, *J*_C,F_ = 7.7 Hz), 128.8, 128.7, 128.1, 127.5, 123.5, 115.6, 115.4, 69.8, 49.5, 46.3, 41.7. ^19^F{^1^H} NMR (376 MHz, CDCl_3_, δ) −114.21. IR (neat): 2925, 1744, 1681, 1511 cm^–1^.

*4-(1-(4-Methoxyphenyl)-3-oxo-3-phenylpropyl)isochroman-3-one* (**14f**) was purified by flash chromatography (silica gel, hexane/ethyl acetate = 8:2), isolated in a total yield of 82%, and analyzed by HRMS. Subsequently, PrepTLC (silica gel, hexane/ethyl acetate = 85:15) was used to separate the diastereomers (dr = 55/45).

HRMS (MALDI-FT ICR): *m*/*z* calcd. for C_25_H_21_KO_4_ [M + K]^+^: 425.1149; found: 425.1155.

(R*)-4-((R*)-1-(4-Methoxyphenyl)-3-oxo-3-phenylpropyl)isochroman-3-one (**14f-1**): white solid (23 mg); mp 138–141 °C. ^1^H NMR (400 MHz, CDCl_3_, δ) 8.13 (dd, *J* = 7.1, 1.5 Hz, 2H), 7.68–7.61 (m, 2H), 7.55 (t, *J* = 7.5 Hz, 2H), 7.48 (t, *J* = 7.5 Hz, 1H), 7.36–7.29 (m, 1H), 6.96 (d, *J* = 7.6 Hz, 1H), 6.90 (d, *J* = 8.8 Hz, 2H), 6.77 (d, *J* = 8.8 Hz, 2H), 4.79 (d, *J* = 14.4 Hz, 1H), 4.34 (dd, *J* = 18.2, 9.3 Hz, 1H), 4.18 (d, *J* = 3.8 Hz, 1H), 3.93 (dt, *J* = 8.8, 4.2 Hz, 1H), 3.82 (s, 3H), 3.74 (d, *J* = 14.3 Hz, 1H), 3.50 (dd, *J* = 18.2, 4.6 Hz, 1H). ^13^C{^1^H} NMR (101 MHz, CDCl_3_, δ) 198.7, 171.2, 159.2, 137.2, 134.0, 133.3, 131.2, 131.0, 129.3, 128.7, 128.6, 128.1, 127.3, 123.4, 113.9, 69.9, 55.2, 49.7, 46.3, 41.8. IR (neat): 3065, 1744, 1679, 1513 cm^–1^.

(R*)-4-((S*)-1-(4-Methoxyphenyl)-3-oxo-3-phenylpropyl)isochroman-3-one (**14f-2**): white solid (19 mg); mp 121–124 °C. ^1^H NMR (400 MHz, CDCl_3_, δ) 8.02 (dd, *J* = 7.1, 1.5 Hz, 2H), 7.62 (t, *J* = 7.3 Hz, 1H), 7.51 (t, *J* = 7.6 Hz, 2H), 7.37–7.26 (m, 1H), 7.22–7.16 (m, 2H), 6.92 (d, *J* = 8.8 Hz, 2H), 6.79 (d, *J* = 8.8 Hz, 2H), 6.62 (d, *J* = 7.9 Hz, 1H), 5.20 (d, *J* = 14.2 Hz, 1H), 5.11 (d, *J* = 14.3 Hz, 1H), 4.06–3.95 (m, 1H), 3.87 (d, *J* = 7.3 Hz, 1H), 3.82 (s, 3H), 3.78 (d, *J* = 7.3 Hz, 1H), 3.51 (dd, *J* = 17.7, 5.5 Hz, 1H). ^13^C{^1^H} NMR (101 MHz, CDCl_3_, δ) 197.9, 172.4, 158.9, 136.9, 133.2, 132.6, 132.6, 131.7, 129.3, 128.7, 128.6, 128.1, 127.7, 127.4, 124.5, 113.9, 69.7, 55.2, 52.7, 42.3, 42.1. IR (neat): 3061, 1741, 1669, 1523 cm^–1^.

*4-(3-Oxo-1-phenyl-3-(3-(trifluoromethyl)phenyl)propyl)isochroman-3-one* (**14g**) was purified by flash chromatography (silica gel, hexane/ethyl acetate = 85:15), isolated in a total yield of 76%, and analyzed by HRMS. Subsequently, PrepTLC (silica gel, hexane/ethyl acetate = 9:1) was used to separate the diastereomers (dr = 53/47).

HRMS (MALDI-FT ICR): *m*/*z* calcd. for C_25_H_19_F_3_KO_3_ [M + K]^+^: 463.0918; found: 463.0945.

(R*)-4-((R*)-2-Oxo-1-phenyl-2-(3-(trifluoromethyl)phenyl)ethyl)isochroman-3-one (**14g-1**): white solid (22 mg); mp 133–135 °C. ^1^H NMR (400 MHz, CDCl_3_, δ) 8.35 (dd, *J* = 18.0, 7.9 Hz, 2H), 7.91 (d, *J* = 8.1 Hz, 1H), 7.91 (d, *J* = 8.1 Hz, 1H), 7.64 (d, *J* = 7.6 Hz, 1H), 7.50 (t, *J* = 7.6 Hz, 1H), 7.33 (q, *J* = 7.0 Hz, 2H), 7.25 (t, *J* = 7.4 Hz, 2H), 6.99 (d, *J* = 7.3 Hz, 2H), 6.95 (d, *J* = 7.5 Hz, 1H), 4.77 (d, *J* = 14.4 Hz, 1H), 4.43 (dd, *J* = 18.4, 9.5 Hz, 1H), 4.21 (d, *J* = 3.8 Hz, 1H), 3.99 (dt, *J* = 9.4, 4.2 Hz, 1H), 3.62 (d, *J* = 14.4 Hz, 1H), 3.54 (dd, *J* = 18.4, 4.3 Hz, 1H), ^13^C{^1^H} NMR (101 MHz, CDCl_3_, δ) 197.4, 171.2, 138.9, 137.6, 133.6, 131.4 (q, *J*_C,F_ = 32.5 Hz), 131.4, 131.1, 129.8, 129.8, 129.4, 128.7, 128.3, 128.1, 127.9, 127.5, 125.0 (q, *J*_C,F_ = 3.7 Hz), 123.5, 69.8, 49.4, 46.9, 41.7. ^19^F{^1^H} NMR (376 MHz, CDCl_3_, δ) −62.76. IR (neat): 2924, 1725, 1685, 1582 cm^–1^.

(R*)-4-((S*)-2-Oxo-1-phenyl-2-(3-(trifluoromethyl)phenyl)ethyl)isochroman-3-one (**14g-2**): white solid (20 mg); mp 114–116 °C. ^1^H NMR (400 MHz, CDCl_3_, δ) 8.21 (s, 1H), 8.16 (d, *J* = 8.0 Hz, 1H), 7.82 (d, *J* = 7.8 Hz, 1H), 7.61 (t, *J* = 7.7 Hz, 1H), 7.28–7.08 (m, 5H), 7.04 (t, *J* = 7.4 Hz, 1H), 6.98 (dd, *J* = 6.5, 3.1 Hz, 2H), 6.39 (d, *J* = 7.5 Hz, 1H), 5.45 (d, *J* = 14.2 Hz, 1H), 5.14 (d, *J* = 14.2 Hz, 1H), 3.97–3.90 (m, 2H), 3.46 (dd, *J* = 13.5, 6.5 Hz, 1H). ^13^C{^1^H} NMR (101 MHz, CDCl_3_, δ) 196.6, 172.4, 140.7, 137.4, 132.7, 131.6, 131.4 (q, *J*_C,F_ = 32.8 Hz), 131.3, 129.7 (q, *J*_C,F_ = 3.9 Hz), 129.3, 128.6, 128.6, 128.3, 127.8, 127.6, 127.5, 125.0, 124.9, 124.6, 69.6, 52.8, 42.5. ^19^F{^1^H} NMR (376 MHz, CDCl_3_, δ) −62.64. IR (neat): 2935, 1727, 1689, 1612 cm^–1^.

(R*)-4-((R*)-3-(4-Nitrophenyl)-3-oxo-1-phenylpropyl)isochroman-3-one (**14h**) was purified by flash chromatography (silica gel, hexane/ethyl acetate = 7:3) and isolated as a white solid (21 mg, yield: 77%); mp 183–186 °C. ^1^H NMR (400 MHz, CDCl_3_, δ) 8.35 (d, *J* = 8.9 Hz, 2H), 8.23 (d, *J* = 9.0 Hz, 2H), 7.56 (d, *J* = 7.7 Hz, 1H), 7.44 (t, *J* = 7.5 Hz, 1H), 7.26 (t, *J* = 5.8 Hz, 2H), 7.18 (t, *J* = 7.2 Hz, 2H), 6.90 (t, *J* = 6.6 Hz, 3H), 4.71 (d, *J* = 14.5 Hz, 1H), 4.39 (dd, *J* = 18.5, 9.5 Hz, 1H), 4.13 (d, *J* = 3.8 Hz, 1H), 3.91 (dt, *J* = 8.7, 4.0 Hz, 1H), 3.57–3.43 (m, 2H). ^13^C{^1^H} NMR (101 MHz, CDCl_3_, δ) 197.3, 171.1, 150.6, 141.5, 138.6, 133.4, 131.1, 129.3, 128.8, 128.7, 128.2, 128.1, 128.0, 127.6, 124.0, 123.5, 69.8, 49.3, 46.9, 42.1. IR (neat): 2944, 1729, 1682, 1597 cm^–1^. HRMS (MALDI-FT ICR): *m*/*z* calcd. for C_24_H_19_NNaO_5_ [M + Na]^+^: 424.1155; found: 424.1177. The other diastereomer was isolated with an unsuitable purity. Therefore, we were not able to perform a full characterization. The overall yield and the dr were determined on the integration of crude NMR.

*4-(3-(4-Methoxyphenyl)-3-oxo-1-phenylpropyl)isochroman-3-one* (**14i**) was purified by flash chromatography (silica gel, hexane/ethyl acetate = 8:2), isolated in a total yield of 73%, and analyzed by HRMS. Subsequently, PrepTLC (silica gel, hexane/ethyl acetate = 85:15) was used to separate the diastereomers (dr = 53/47).

HRMS (MALDI-FT ICR): *m*/*z* calcd. for C_25_H_22_NaO_4_ [M + Na]^+^: 409.1410; found: 409.1417.

(R*)-4-((R*)-3-(4-Methoxyphenyl)-3-oxo-1-phenylpropyl)isochroman-3-one (**14i-1**): white solid (20 mg); mp 146–148 °C. ^1^H NMR (400 MHz, CDCl_3_, δ) 8.13 (d, *J* = 9.0 Hz, 2H), 7.64 (dd, *J* = 13.2, 7.2 Hz, 2H), 7.55 (t, *J* = 7.5 Hz, 2H), 7.48 (t, *J* = 7.5 Hz, 1H), 7.32 (t, *J* = 7.5 Hz, 1H), 6.96 (d, *J* = 7.6 Hz, 1H), 6.90 (d, *J* = 8.8 Hz, 2H), 6.77 (d, *J* = 8.8 Hz, 2H), 4.79 (d, *J* = 14.4 Hz, 1H), 4.34 (dd, *J* = 18.2, 9.3 Hz, 1H), 4.18 (d, *J* = 3.8 Hz, 1H), 3.93 (dt, *J* = 8.8, 4.2 Hz, 1H), 3.82 (s, 3H), 3.74 (d, *J* = 14.3 Hz, 1H), 3.50 (dd, *J* = 18.2, 4.6 Hz, 1H). ^13^C{^1^H} NMR (101 MHz, CDCl_3_, δ) 198.7, 171.3, 159.2, 137.2, 134.0, 133.3, 131.2, 131.1, 129.3, 128.7, 128.7, 128.2, 127.4, 123.4, 113.9, 69.9, 55.3, 49.7, 46.4, 41.8. IR (neat): 2994, 1744, 1679, 1513 cm^–1^.

(R*)-4-((S*)-3-(4-Methoxyphenyl)-3-oxo-1-phenylpropyl)isochroman-3-one (**14i-2**): white solid (18 mg); mp 141–143 °C, ^1^H NMR (400 MHz, CDCl_3_, δ) 8.02 (d, *J* = 8.9 Hz, 2H),7.62 (t, *J* = 7.3 Hz, 1H), 7.51 (t, *J* = 7.6 Hz, 2H), 7.30 (t, *J* = 8.3 Hz, 1H), 7.18 (t, *J* = 7.7 Hz, 2H), 6.92 (d, *J* = 8.8 Hz, 2H), 6.79 (d, *J* = 8.8 Hz, 2H), 6.62 (d, *J* = 7.9 Hz, 1H), 5.20 (d, *J* = 14.2 Hz, 1H), 5.11 (d, *J* = 14.2 Hz, 1H), 4.09–3.95 (m, 1H), 3.87 (d, *J* = 7.3 Hz, 1H), 3.82 (s, 3H), 3.78 (t, *J* = 6.7 Hz, 1H). ^13^C{^1^H} NMR (101 MHz, CDCl_3_, δ) 197.9, 172.5, 158.9, 136.9, 133.3, 132.65, 132.62, 131.8, 129.3, 128.7, 128.6, 128.1, 127.8, 127.4, 124.5, 113.9, 69.7, 55.3, 52.7, 42.3, 42.1. IR (neat): 2991, 1740, 1681, 1599 cm^–1^.

*((Furan-2-yl)-3-oxo-3-phenylpropyl)isochroman-3-one* (**14j**) was purified by flash chromatography (silica gel, hexane/ethyl acetate = 80:20) and isolated as a white solid (30 mg, yield: 65%), a mixture of diastereomers (dr = 58/42). ^1^H NMR (400 MHz, CDCl_3_, δ) 8.10^major^ (dd, *J* = 7.1, 1.4 Hz, 2H), 8.04^minor^ (dd, *J* = 7.1, 1.4 Hz, 2H), 7.64 (d, *J* = 7.3 Hz, 2H), 7.58–7.51 (m, 5H), 7.45 (t, *J* = 7.4 Hz, 2H), 7.40–7.33 (m, 3H), 7.31–7.16 (m, 3H), 7.08^major^ (d, *J* = 7.7 Hz, 1H), 6.70^minor^ (d, *J* = 7.7 Hz, 1H), 6.30^major^ (dd, *J* = 3.2, 1.9 Hz, 1H), 6.26^minor^ (dd, *J* = 3.2, 1.9 Hz, 1H), 5.89^minor^ (d, *J* = 3.2 Hz, 1H), 5.84^major^ (d, *J* = 3.2 Hz, 1H), 5.54^minor^ (d, *J* = 14.3 Hz, 1H), 5.38^major^ (s, 1H), 5.24^minor^ (d, *J* = 14.3 Hz, 1H), 5.02^major^ (d, *J* = 14.5 Hz, 1H), 4.31^major^ (d, *J* = 13.4 Hz, 1H), 4.19–4.07 (m, 3H), 3.86–3.77^major^ (m, 1H), 3.63–3.51 (m, 3H), ^13^C{^1^H} NMR (75 MHz, CDCl_3_, δ) 198.6, 197.8, 171.7, 170.5, 140.6, 138.9, 137.0, 136.7, 133.8, 133.5, 133.4, 133.1, 133.0, 131.9, 130.9, 130.2, 129.9, 128.8, 128.8, 128.7, 128.6, 128.3, 128.2, 128.1, 128.0, 127.8, 127.7, 127.7, 126.5, 121.4, 121.2, 68.9, 68.8, 52.3, 49.0, 47.1, 42.6, 42.1, 41.4. HRMS (MALDI-FT ICR): *m*/*z* calcd. for C_22_H_18_NaO_4_ [M + Na]^+^: 369.1097; found: 369.1093.

*7-Bromo-4-(3-oxo-1,3-diphenylpropyl)isochroman-3-one* (**14k**) was purified by flash chromatography (silica gel, hexane/ethyl acetate = 80:20) and isolated as a white solid (47 mg, yield: 81%), a mixture of diastereomers (dr = 54/46). ^1^H NMR (400 MHz, CDCl_3_, δ) 8.14^major^ (dd, *J* = 7.1, 1.4 Hz, 2H), 8.03^minor^ (d, *J* = 7.1, 1.4, 2H), 7.71–7.48 (m, 8H), 7.41–7.21 (m, 9H), 7.12–6.97 (m, 5H), 6.36^minor^ (d, *J* = 7.9 Hz, 1H), 5.35^minor^ (d, *J* = 14.3 Hz, 1H), 5.10 ^minor^ (d, *J* = 14.3 Hz, 1H), 4.69^major^ (d, *J* = 14.5 Hz, 1H), 4.43^major^ (dd, *J* = 18.4, 9.8 Hz, 1H), 4.19^major^ (d, *J* = 3.5 Hz, 1H), 4.02–3.88 (m, 3H), 3.55–3.45 (m, 4H). ^13^C{^1^H} NMR (75 MHz, CDCl_3_, δ) 198.6, 197.8, 171.7, 170.5, 140.6, 138.9, 137.0, 136.7, 133.8, 133.5, 133.4, 133.1, 133.0, 131.9, 130.9, 130.2, 129.9, 128.8, 128.8, 128.7, 128.6, 128.3, 128.2, 128.1, 128.0, 127.8, 127.74, 127.7, 126.5, 121.4, 121.2, 68.9, 68.8, 52.3, 49.0, 47.1, 42.6, 42.1, 41.4. HRMS (MALDI-FT ICR): *m*/*z* calcd. for C_24_H_19_BrNaO_3_ [M + Na]^+^: 457.0410; found: 457.0419.

*6-Methoxy-4-(3-oxo-1,3-diphenylpropyl)isochroman-3-one* (**14l**) was purified by flash chromatography (silica gel, hexane/ethyl acetate = 75:25), isolated in a total yield of 96%, and analyzed by HRMS. Subsequently, PrepTLC (silica gel, hexane/ethyl acetate = 80:20) was used to separate the diastereomers (dr = 50/50).

HRMS (MALDI-FT ICR): *m*/*z* calcd. For C_25_H_21_KO_4_ [M + K]^+^: 425.1149; found: 425.1189.

(R*)-6-Methoxy-4-((S*)-3-oxo-1,3-diphenylpropyl)isochroman-3-one (**14l-1**): white solid (25 mg); mp 157–159 °C. ^1^H NMR (400 MHz, CDCl_3_, δ) 8.13 (dd, *J* = 7.0, 1.5 Hz, 2H), 7.65 (t, *J* = 7.4 Hz, 2H), 7.33–7.29 (m, 1H), 7.24 (t, *J* = 7.3 Hz, 2H), 7.14 (s, 1H), 7.04 (d, *J* = 6.9 Hz, 2H), 6.86 (s, 2H), 4.38 (dd, *J* = 18.3, 9.4 Hz, 1H), 4.16 (d, *J* = 3.9 Hz, 1H), 4.01 (dt, *J* = 9.1, 4.2 Hz, 1H), 3.95 (s, 3H), 3.59 (d, *J* = 13.9 Hz, 1H), 3.53 (dd, *J* = 18.3, 4.4 Hz, 1H). ^13^C{^1^H} NMR (101 MHz, CDCl_3_, δ) 198.6, 171.2, 159.9, 139.3, 137.1, 135.3, 133.3, 128.7, 128.6, 128.4, 128.2, 127.8, 124.7, 123.4, 114.1, 112.3, 69.5, 55.5, 50.0, 46.8, 41.5. IR (neat): 3061, 1734, 1685, 1598 cm^–1^.

(R*)-6-Methoxy-4-((S*)-3-oxo-1,3-diphenylpropyl)isochroman-3-one (**14l-2**): white solid (25 mg); mp 146–148 °C. ^1^H NMR (400 MHz, CDCl_3_, δ) 8.03 (dd, *J* = 7.1, 1.5 Hz, 2H), 7.51 (t, *J* = 7.6 Hz, 2H), 7.30–7.26 (m, 3H), 7.11 (d, *J* = 8.4 Hz, 1H), 7.07 (d, *J* = 3.6 Hz, 2H), 6.81 (dd, *J* = 8.4, 2.6 Hz, 1H), 5.95 (d, *J* = 2.6 Hz, 1H), 5.39–5.29 (m, 1H), 5.11 (d, *J* = 13.8 Hz, 1H), 3.99 (d, *J* = 7.9 Hz, 1H), 3.93 (d, *J* = 7.9 Hz, 1H), 3.88 (d, *J* = 12.4 Hz, 1H), 3.58 (d, *J* = 4.7 Hz, 1H), 3.53 (s, 3H). ^13^C{^1^H} NMR (101 MHz, CDCl_3_, δ) 197.9, 172.4, 159.0, 141.0, 136.9, 134.2, 133.3, 128.7, 128.6, 128.5, 128.1, 127.4, 125.7, 123.7, 114.0, 113.1, 69.4, 55.2, 53.1, 42.5, 42.2. IR (neat): 3065, 1724, 1681, 1612 cm^–1^.

*7-Nitro-4-(3-oxo-1,3-diphenylpropyl)isochroman-3-one* (**14m**). The reaction mixture was heated at 110 °C (oil bath). The crude product was purified by flash chromatography (silica gel, hexane/ethyl acetate = 7:3) to afford a white solid (31 mg, yield: 58%), a mixture of diastereomers (dr = 53/47). ^1^H NMR (400 MHz, CDCl_3_, δ) 8.15^major^ (d, *J* = 6.5 Hz, 2H), 8.04^minor^ (d, *J* = 7.7 Hz, 2H), 7.94^major^ (t, *J* = 10.2 Hz, 2H), 7.87^minor^ (s, 1H), 7.67^major^ (dt, *J* = 14.7, 7.2 Hz, 2H), 7.59–7.51 (m, 3H), 7.26–7.38 (m, 7H), 7.05 (d, *J* = 3.6 Hz, 2H), 6.98 (d, *J* = 7.2 Hz, 2H), 6.58^minor^ (d, *J* = 8.4 Hz, 1H), 5.66^major^ (d, *J* = 14.3 Hz, 1H), 5.32^major^ (d, *J* = 14.6 Hz, 1H), 4.84 (d, *J* = 14.9 Hz, 1H), 4.51 (dd, *J* = 18.9, 10.3 Hz, 1H), 4.37 (d, *J* = 3.4 Hz, 1H), 4.11–3.95 (m, 4H), 3.53–3.46 (m, 3H). ^13^C{^1^H} NMR (101 MHz, CDCl_3_, δ) 199.9, 199.1, 172.2, 171.0, 148.6, 142.9, 139.9, 138.2, 135.0, 135.0, 134.0, 131.1, 131.0, 130.4, 130.4, 130.2, 130.1, 129.7, 129.6, 129.5, 129.4, 125.2, 124.1, 121.4, 120.3, 70.2, 70.0, 54.4, 50.6, 48.8, 44.0, 43.5, 42.7. HRMS (MALDI-FT ICR): *m*/*z* calcd for C_24_H_19_NNaO_5_ [M + Na]^+^: 424.1155; found: 424.1180.

*6-Chloro-4-(3-oxo-1,3-diphenylpropyl)isochroman-3-one* (**14n**) was purified by flash chromatography (silica gel, hexane/ethyl acetate = 85:15), isolated in a total yield of 94%, and analyzed by HRMS. Subsequently, PrepTLC (silica gel, hexane/ethyl acetate = 90:10) was used to separate the diastereomers (dr = 52/48).

HRMS (MALDI-FT ICR): *m*/*z* calcd. For C_24_H_19_ClNaO_3_ [M + Na]^+^: 413.0914; found: 413.0919.

(R*)-6-Chloro-4-((R*)-3-oxo-1,3-diphenylpropyl)isochroman-3-one (**14n-1**): white solid (25 mg); mp 146–148 °C. ^1^H NMR (400 MHz, CDCl_3_, δ) 8.14 (dd, *J* = 7.0, 1.5 Hz, 2H), 7.69 (d, *J* = 2.1 Hz, 1H), 7.65 (dt, *J* = 7.4, 1.4 Hz, 1H), 7.56 (t, *J* = 7.5 Hz, 2H), 7.33–7.28 (m, 2H), 7.25 (t, *J* = 7.3 Hz, 2H), 7.02 (s, 1H), 7.00 (d, *J* = 1.6 Hz, 1H), 6.88 (d, *J* = 8.1 Hz, 1H), 4.71 (d, *J* = 14.5 Hz, 1H), 4.42 (dd, *J* = 18.4, 9.8 Hz, 1H), 4.19 (d, *J* = 3.7 Hz, 1H), 3.96 (dt, *J* = 9.8, 3.8 Hz, 1H), 3.54–3.50 (m, 1H), 3.48 (d, *J* = 4.0 Hz, 1H). ^13^C{^1^H} NMR (101 MHz, CDCl_3_, δ) 198.6, 171.2, 159.9, 139.3, 137.1, 135.3, 133.4, 128.7, 128.6, 128.4, 128.2, 127.8, 124.7, 123.4, 114.1, 112.3, 69.5, 55.5, 50.0, 46.8, 41.5. IR (neat): 3029, 1766, 1680, 1600 cm^–1^.

(R*)-6-Chloro-4-((S*)-3-oxo-1,3-diphenylpropyl)isochroman-3-one (**14n-2**): white solid (23 mg); mp 129–132 °C. ^1^H NMR (400 MHz, CDCl_3_, δ) 8.03 (dd, *J* = 7.1, 1.5 Hz, 2H), 7.63 (t, *J* = 7.4 Hz, 1H), 7.52 (t, *J* = 7.6 Hz, 2H), 7.33–7.29 (m, 3H), 7.26 (dd, *J* = 8.1, 2.0 Hz, 1H), 7.15 (d, *J* = 8.1 Hz, 1H), 7.04 (d, *J* = 2.2 Hz, 1H), 7.02 (d, *J* = 3.7 Hz, 1H), 6.44 (d, *J* = 2.1 Hz, 1H), 5.35 (d, *J* = 14.3 Hz, 1H), 5.13 (d, *J* = 14.3 Hz, 1H), 4.02–3.97 (m, 1H), 3.94–3.89 (m, 2H), 3.53 (dd, *J* = 12.8, 4.4 Hz, 1H). ^13^C{^1^H} NMR (75 MHz, CDCl_3_, δ) 197.7, 171.5, 140.3, 136.6, 134.6, 133.5, 133.3, 130.0, 128.6, 128.1, 128.0, 127.6, 127.4, 125.7, 68.8, 52.5, 42.4, 41.8. IR (neat): 3029, 1734, 1685, 1598 cm^–1^.

*6-Methoxy-4-(1-(4-methoxyphenyl)-3-oxo-3-phenylpropyl)isochroman-3-one* (**14o**) was purified by flash chromatography (silica gel, hexane/ethyl acetate = 8:2) and isolated as a white solid (52 mg, yield: 93%), a mixture of diastereomers (dr = 51/49). ^1^H NMR (400 MHz, CDCl_3_, δ) 8.11 (dd, *J* = 7.1 Hz, 1.5 Hz, 2H), 8.02 (dd, *J* = 7.1 Hz, 1.5 Hz, 2H), 7.68–7.57 (m, 2H), 7.52 (dt, *J* = 14.5, 7.5 Hz, 4H), 7.12 (s, 1H), 7.09 (d, *J* = 8.4 Hz, 1H), 6.96–6.92 (m, 5H), 6.87 (m, 2H), 6.85–6.69 (m, 5H), 5.18 (d, *J* = 13.8 Hz, 1H), 5.06 (d, *J* = 13.9 Hz, 1H), 4.73 (d, *J* = 13.9 Hz, 1H), 4.32 (dd, *J* = 18.3, 9.3 Hz, 1H), 4.12 (d, *J* = 3.8 Hz, 1H), 4.05–3.95 (m, 1H), 3.94 (s, 3H), 3.90 (dd, *J* = 8.2, 2.7 Hz, 1H), 3.85 (d, *J* = 7.9 Hz, 1H), 3.80 (s, 6H), 3.69 (d, *J* = 14.0 Hz, 1H), 3.58 (s, 3H), 3.53 (dd, *J* = 7.0, 5.1 Hz, 1H), 3.48 (dd, *J* = 7.7, 5.2 Hz, 1H). ^13^C{^1^H} NMR (75 MHz, CDCl_3_, δ) 198.7, 198.0, 172.5, 171.3, 159.9, 159.2, 159.0, 158.9, 137.2, 136.9, 135.4, 134.1, 133.3, 133.3, 132.7, 131.2, 129.4, 129.3, 128.7, 128.7, 128.2, 128.1, 125.6, 124.8, 123.8, 123.3, 114.0, 113.9, 113.3, 112.3, 69.7, 69.4, 55.5, 55.3, 55.3, 55.2, 53.0, 50.1, 46.0, 42.3, 42.0, 41.8. HRMS (MALDI-FT ICR): *m*/*z* calcd. for C_26_H_24_NaO_5_ [M + Na]^+^: 439.1516; found: 439.1525.

*8-Fluoro-4-(-3-oxo-1,3-diphenylpropyl)isochroman-3-one* (**14p**) was purified by flash chromatography (silica gel, hexane/ethyl acetate = 80:20), isolated in a total yield of 90%, and analyzed by HRMS. Subsequently, PrepTLC (silica gel, hexane/ethyl acetate = 85:15) was used to separate the diastereomers (dr = 59/41).

HRMS (MALDI-FT ICR): *m*/*z* calcd. For C_24_H_20_FO_3_ [M + H]^+^: 375.1391; found: 375.1386.

(R*)-8-Fluoro-4-((R*)-3-oxo-1,3-diphenylpropyl)isochroman-3-one (**14p-1**): white solid (30 mg); mp 119–121 °C. ^1^H NMR (400 MHz, CDCl_3_, δ) 8.07 (dd, *J* = 7.1, 1.5 Hz, 2H), 7.59 (t, *J* = 7.4 Hz, 1H), 7.54–7.41 (m, 2H), 7.43–7.38 (m, 2H), 7.25 (d, *J* = 7.3 Hz, 1H), 7.19 (t, *J* = 7.9 Hz, 2H), 6.99–6.92 (m, 1H), 6.91 (d, *J* = 7.0 Hz, 2H), 4.95 (d, *J* = 15.0 Hz, 1H), 4.35 (dd, *J* = 18.4, 9.7 Hz, 1H), 4.18 (d, *J* = 3.6 Hz, 1H), 3.89 (dt, *J* = 9.7, 3.8 Hz, 1H), 3.44 (dd, *J* = 18.4, 4.1 Hz, 1H), 3.36 (d, *J* = 15.0 Hz, 1H). ^13^C NMR (63 MHz, CDCl_3_, δ) 198.4, 170.3, 157.2 (d, *J*_C,F_ = 247.2 Hz), 138.7, 136.8, 136.5, 136.4, 133.3, 130.0 (d, *J*_C,F_ = 8.1 Hz), 128.6, 128.1, 128.0, 127.9, 123.5 (d, *J*_C,F_ = 3.3 Hz), 118.7 (d, *J*_C,F_ = 16.6 Hz), 113.7 (d, *J*_C,F_ = 20.2 Hz), 64.2 (d, *J*_C,F_ = 3.7 Hz), 48.8, 47.1, 41.2. ^19^F{^1^H} NMR (376 MHz, CDCl_3_, δ) −120.74. IR (neat): 2947, 1755, 1656, 929 cm^–1^.

(R*)-8-Fluoro-4-((S*)-3-oxo-1,3-diphenylpropyl)isochroman-3-one (**14p-2**): white solid (21 mg); mp 113–115 °C. ^1^H NMR (300 MHz, CDCl_3_, δ) 7.97 (dd, *J* = 7.0, 1.5 Hz, 2H), 7.57 (tt, *J* = 7.4, 1.5 Hz, 1H), 7.46 (t, *J* = 7.4 Hz, 2H), 7.24–7.19 (m, 3H), 7.09–7.01 (m, 1H), 6.99–6.89 (m, 3H), 6.29 (d, *J* = 7.6 Hz, 1H), 5.40 (d, *J* = 13.8 Hz, 1H), 5.03 (d, *J* = 14.8 Hz, 1H), 4.00–3.95 (m, 2H), 3.85 (ddd, *J* = 17.6, 5.0, 2.7 Hz, 1H), 3.45 (ddd, *J* = 17.5, 3.4, 1.3 Hz, 1H). ^13^C NMR (63 MHz, CDCl_3_, δ) 197.6, 171.6, 157.8 (d, *J*_C,F_ = 247.4 Hz), 140.3, 136.6, 135.2, 133.2, 129.1 (d, *J*_C,F_ = 8.2 Hz), 128.55, 128.50, 128.1, 127.9, 127.5, 124.1, 119.1 (d, *J*_C,F_ = 16.3 Hz), 114.0 (d, *J*_C,F_ = 20.4 Hz), 63.4 (d, *J*_C,F_ = 3.7 Hz), 52.0, 42.8, 41.8. ^19^F{^1^H} NMR (376 MHz, CDCl_3_, δ) −119.96. IR (neat): 2942, 1749, 1627, 925 cm^–1^.

*3-(3-Oxo-1,3-diphenylpropyl)benzofuran-2(3H)-one* (**15a**) was purified by flash chromatography (silica gel, hexane/ethyl acetate = 75:25) and isolated as a white solid (37 mg, yield: 81%), a mixture of diastereomers (dr = 64/36).

^1^H NMR (400 MHz, CDCl_3_, δ) 8.11^major^ (dd, *J* = 7.0, 1.5 Hz, 2H), 8.02^minor^ (dd, *J* = 7.0, 1.5 Hz, 2H) 7.64 (q, *J* = 8.1 Hz 2H), 7.52–7.56 (m, 4H), 7.44^major^ (d, *J* = 7.2 Hz, 1H), 7.31 (d, *J* = 7.7 Hz, 4H), 7.17–7.25 (m, 9H), 7.06 (d, *J* = 7.9 Hz, 1H), 6.92^major^ (d, *J* = 7.9 Hz, 1H), 6.76^minor^ (d, *J* = 7.9 Hz, 1H), 4.24–4.28 (m, 4H), 4.20^major^ (d, *J* = 7.3 Hz, 1H), 4.04–4.07 (m, 1H), 4.00^minor^ (d, *J* = 6.6 Hz, 1H), 3.71^minor^ (d, *J* = 6.7 Hz, 1H), 3.57–3.68 (m, 2H). ^13^C{^1^H} NMR (75 MHz, CDCl_3_, δ) 198.3, 197.5, 176.1, 175.5, 153.7, 153.4, 139.9, 138.7, 136.7, 136.6, 133.4, 133.2, 129.0, 128.7, 128.6, 128.5, 128.3, 128.05, 128.00, 127.7, 127.5, 127.3, 126.0, 125.7, 125.1, 124.5, 123.9, 123.7, 110.6, 110.3, 47.9, 47.5, 42.8, 41.9, 41.4, 39.5. HRMS (MALDI-FT ICR) *m*/*z*: calcd for C_23_H_18_NaO_3_ [M + Na]^+^: 365.1148, found: 365.1140.

*3-(1-(4-Chlorophenyl)-2-oxo-2-phenylethyl)benzofuran-2(3H)-one* (**15b**) was purified by flash chromatography (silica gel, hexane/ethyl acetate = 75:25) and isolated as a white solid (42 mg, yield: 83%), a mixture of diastereomers (dr = 53/47). ^1^H NMR (400 MHz, CDCl_3_, δ) 8.10^major^ (d, *J* = 7.1 Hz, 2H), 8.00^minor^ (d, *J* = 7.1 Hz, 1H), 7.66^major^ (q, *J* = 7.6 Hz, 2H), 7.58–7.51 (m, 4H), 7.45 (d, *J* = 7.5 Hz, 1H), 7.39–7.30^minor^ (m, 2H), 7.27^major^ (d, *J* = 8.4 Hz, 2H), 7.22 (d, *J* = 8.6 Hz, 1H), 7.19–7.12^major^ (m, 4H), 7.12–7.07^minor^ (m, 3H), 6.96 (d, *J* = 8.1 Hz, 1H), 6.89 (d, *J* = 7.6 Hz, 1H), 4.31–4.15 (m, 4H), 4.08^minor^ (q, *J* = 7.4 Hz, 1H), 3.93^major^ (dd, *J* = 17.3, 6.3 Hz, 1H), 3.72–3.56 (m, 2H). ^13^C{^1^H} NMR (75 MHz, CDCl_3_, δ) 197.9, 197.1, 175.8, 175.2, 153.7, 153.3, 138.2, 137.1, 136.5, 136.4, 133.5, 133.4, 133.1, 129.7, 129.3, 129.2, 128.9, 128.6, 128.5, 128.0, 127.9, 125.7, 125.2, 124.9, 124.3, 124.0, 123.8, 110.8, 110.5, 47.9, 47.5, 42.2, 41.3, 41.1, 39.5. HRMS (MALDI-FT ICR) *m*/*z*: calcd for C_23_H_17_ClNaO_3_ [M + Na]^+^: 399.0758, found: 399.0762.

*3-(1-(4-Methoxyphenyl)-2-oxo-2-phenylethyl)benzofuran-2(3H)-one* (**15c**) was purified by flash chromatography (silica gel, hexane/ethyl acetate = 70:30) and isolated as a white solid (43 mg, yield: 87%), a mixture of diastereomers (dr = 54/46). ^1^H NMR (400 MHz, CDCl_3_, δ) 8.11^major^ (dd, *J* = 7.0, 1.4 Hz, 2H), 8.01^minor^ (dd, *J* = 7.0, 1.4 Hz, 2H), 7.64 ^major^ (q, *J* = 8.4 Hz, 2H), 7.54 (q, *J* = 8.4 Hz, 4H), 7.45 (d, *J* = 7.3 Hz, 1H), 7.32 (t, *J* = 7.3 Hz, 1H), 7.26 (t, *J* = 7.4 Hz, 1H), 7.20 (td, *J* = 7.6, 1.2 Hz, 1H), 7.09 (dd, *J* = 15.9, 8.8 Hz, 6H), 6.94 ^minor^ (d, *J* = 9.2 Hz, 1H), 6.87–6.80 (m, 3H), 6.73 (d, *J* = 8.8 Hz, 2H), 4.30–4.20 (m, 3H), 4.17 (d, *J* = 7.4 Hz, 1H), 4.06–3.91 (m, 2H), 3.82^minor^ (s, 3H), 3.76 ^major^ (s, 3H), 3.65^major^ (dd, *J* = 16.9, 7.3 Hz, 1H), 3.58^minor^ (d, *J* = 12.6 Hz, 1H). ^13^C{^1^H} NMR (75 MHz, CDCl_3_, δ) 198.5, 197.8, 176.2, 175.7, 158.9, 158.7, 153.9, 153.6, 136.9, 136.8, 133.4, 133.3, 131.9, 130.8, 129.5, 129.1, 128.8, 128.7, 128.2, 128.1, 126.4, 125.9, 125.3, 124.6, 124.0, 123.8, 113.9, 113.8, 110.8, 110.5, 55.2, 55.1, 48.3, 47.9, 42.3, 41.8, 41.4, 39.9. HRMS (MALDI-FT ICR) *m*/*z*: calcd for C_24_H_20_NaO_4_ [M + Na]^+^: 395.1234, found: 395.1253.

*2-Oxo-3-(2-oxo-1,2-diphenylethyl)-2,3-dihydrobenzofuran-5-yl acetate* (**15d**) was purified by flash chromatography (silica gel, hexane/ethyl acetate = 75:25) and isolated as a white solid (48 mg, yield: 89%), a mixture of diastereomers (dr = 59/41). ^1^H NMR (400 MHz, CDCl_3_, δ) 8.10^major^ (dd, *J* = 7.1, 1.5 Hz, 2H), 8.02 (dd, *J* = 7.1, 1.5 Hz, 2H), 7.64^major^ (q, *J* = 7.5 Hz, 2H), 7.59–7.50 (m, 3H), 7.42^minor^ (d, *J* = 7.0 Hz, 1H), 7.38–7.28 (m, 2H), 7.26–7.20 (m, 5H), 7.18 (d, *J* = 7.9 Hz, 2H), 7.04–6.99 (m, 1H), 6.96 (dd, *J* = 8.6, 2.4 Hz, 1H), 6.90 (d, *J* = 8.6, 1H), 6.55 (m, 1H), 4.36–4.23^major^ (m, 3H), 4.21^minor^ (d, *J* = 8.8 Hz, 1H), 4.07^minor^ (q, *J* = 7.1 Hz, 1H), 3.97^minor^ (dd, *J* = 17.1, 6.9 Hz, 1H), 3.68^minor^ (dd, *J* = 17.2, 7.0 Hz, 1H), 3.59^major^ (dd, *J* = 17.1, 3.8 Hz, 1H), 2.38^major^ (s, 3H), 2.31^minor^ (s, 3H). ^13^C{^1^H} NMR (75 MHz, CDCl_3_, δ) 198.3, 197.6, 175.9, 175.6, 169.6, 169.5, 151.3, 150.9, 146.9, 146.6, 139.6, 138.5, 136.8, 136.7, 133.5, 133.4, 130.0, 129.4, 128.77, 128.74, 128.6, 128.5, 128.2, 128.2, 127.8, 127.6, 127.2, 126.7, 122.3, 121.9, 119.2, 118.6, 111.2, 111.0, 48.5, 48.2, 43.0, 42.1, 41.4, 39.5, 21.1, 21.0. HRMS (MALDI-FT ICR) *m*/*z*: calcd for C_25_H_20_KO_5_ [M + K]^+^: 439.0942, found: 439.0967.

One diastereomer was separated by crystallization from a mixture of isopropanol/ether = 1:5 (1 mL):

(R*)-2-Oxo-3-((S*)-3-oxo-1,3-diphenylpropyl)-2,3-dihydrobenzofuran-5-yl acetate: white solid (28 mg); mp 116.5–117.7 °C. ^1^H NMR (400 MHz, CDCl_3_, δ) 8.10 (dd, *J* = 7.1, 1.5 Hz, 2H), 7.66 (q, *J* = 7.4, 1H), 7.55 (t, *J* = 7.6 Hz, 2H), 7.26–7.16 (m, 6H), 6.96 (dd, *J* = 8.6, 2.4 Hz, 1H), 4.34–4.21 (m, 3H), 3.59 (dd, *J* = 17.2, 4.0 Hz, 1H), 2.38 (s, 3H). ^13^C{^1^H} NMR (75 MHz, CDCl_3_, δ) 197.3, 171.2, 150.6, 141.5, 138.6, 133.4, 131.0, 129.3, 128.8, 128.7, 128.2, 128.1, 128.0, 127.6, 124.0, 123.5, 69.8, 49.3, 46.9, 42.1. IR (KBr): 2923, 1786, 1769, 1688, 1643, 1487 cm^–1^. HRMS (MALDI-FT ICR) *m*/*z*: calcd for C_25_H_20_KO_5_ [M + K]^+^: 439.0942, found: 439.0967.

*3-(3-(4-Methoxyphenyl)-3-oxo-1-phenylpropyl)benzofuran-2(3H)-one* (**15e**) was purified by flash chromatography (silica gel, hexane/ethyl acetate = 70:30) and isolated as a white solid (41 mg, yield: 83% yield), a mixture of diastereomers (dr = 62/38). ^1^H NMR (400 MHz, CDCl_3_, δ) 8.09^major^ (d, *J* = 9.0 Hz, 2H), 8.00^minor^ (d, *J* = 8.9 Hz, 2H), 7.44^major^ (d, *J* = 7.3 Hz, 1H), 7.35–7.25 (m, 3H), 7.24–7.14 (m, 8H), 7.10–7.04^major^ (m, 1H), 7.00 (t, *J* = 9.5 Hz, 3H), 6.91^major^ (d, *J* = 8.7 Hz, 1H), 6.84^minor^ (d, *J* = 7.4 Hz, 1H), 4.32–4.23 (m, 3H), 4.21^major^ (d, *J* = 8.6 Hz, 1 H), 4.07^minor^ (q, *J* = 7.1 Hz, 1H), 3.93^major^ (s, 3H), 3.92^minor^ (s, 3H), 3.63^minor^ (dd, *J* = 17.0, 7.3 Hz, 1H), 3.54^major^ (dd, *J* = 16.6, 4.1 Hz, 1H), ^13^C{^1^H} NMR (75 MHz, CDCl_3_, δ) 196.9, 196.1, 176.2, 175.7, 163.8, 163.7, 153.9, 153.6, 140.1, 139.0, 130.5, 130.4, 130.0, 129.9, 129.1, 128.8, 128.6, 128.5, 128.4, 128.1, 127.6, 127.4, 126.3, 125.9, 125.3, 124.7, 124.0, 123.8, 113.9, 110.7, 110.4, 55.6, 48.1, 47.8, 43.2, 42.3, 41.1, 39.2. HRMS (MALDI-FT ICR) *m*/*z*: calcd for C_24_H_20_KO_4_ [M + K]^+^: 411.0993, found: 411.0973.

*3-(1-(2-Chlorophenyl)-3-oxo-3-phenylpropyl)benzofuran-2(3H)-one* (**15f**) was purified by flash chromatography (silica gel, hexane/ethyl acetate = 75:25) and isolated as a white solid (41 mg, yield: 81% yield), a mixture of diastereomers (dr = 54/46). ^1^H NMR (400 MHz, CDCl_3_, δ) 8.02^major^ (dd, *J* = 7.1, 1.4 Hz, 2H), 7.96^minor^ (d, *J* = 7.1, 1.4 Hz, 2H), 7.62 (q, *J* = 8.0 Hz, 2H), 7.54–7.46 (m, 4H), 7.40 (dd, *J* = 5.8, 3.7 Hz, 1H), 7.37–7.31 (m, 3H), 7.29 (dd, *J* = 8.5, 5.0 Hz, 3H), 7.24–7.06 (m, 5H), 7.03 (d, *J* = 8.2 Hz, 1H), 6.84^minor^ (d, *J* = 7.0 Hz, 1H), 5.01^major^ (td, *J* = 7.4, 4.8 Hz, 1H), 4.59^minor^ (q, *J* = 7.4 Hz, 1H), 4.45^major^ (d, *J* = 4.8 Hz, 1H), 3.97^major^ (dd, *J* = 18.0, 7.7 Hz, 1H), 3.81^minor^ (dd, *J* = 17.6, 8.8 Hz, 1H), 3.69^minor^ (dd, *J* = 17.7, 5.0 Hz, 1H), 3.48^major^ (dd, *J* = 18.0, 6.9 Hz, 1H). ^13^C{^1^H} NMR (75 MHz, CDCl_3_, δ) 197.5, 197.0, 176.2, 175.2, 153.6, 153.4, 137.8, 137.3, 136.4, 134.3, 134.1, 133.3, 133.2, 130.1, 130.0, 129.2, 128.9, 128.6, 128.5, 128.4, 128.0, 127.9, 127.6, 127.03, 127.00, 125.9, 125.2, 125.0, 124.7, 124.1, 123.7, 110.6, 110.3, 46.4, 46.2, 39.1, 39.0, 37.1. HRMS (MALDI-FT ICR) *m*/*z*: calcd for C_23_H_17_ClKO_3_ [M + K]^+^: 415.0497, found: 415.0499.

*3-(1-(4-Fluorophenyl)-3-oxo-3-phenylpropyl)benzofuran-2(3H)-one* (**15g**). The reaction mixture was heated at 50 °C (oil bath). The crude product was purified by flash chromatography (silica gel, hexane/ethyl acetate = 75:25) and isolated as a white solid (38 mg, yield: 79%), a mixture of diastereomers (dr = 64/36). ^1^H NMR (400 MHz, CDCl_3_, δ) 8.11^major^ (dd, *J* = 7.2, 1.3 Hz, 2H), 8.01^minor^ (dd, *J* = 7.2, 1.3 Hz, 2H), 7.65^major^ (q, *J* = 7.8 Hz, 2H), 7.58–7.51 (m, 3H), 7.46^major^ (d, *J* = 6.5 Hz, 1H), 7.34^minor^ (t, *J* = 7.9 Hz, 1H), 7.26 (d, *J* = 8.6 Hz, 1H), 7.22 (dd, *J* = 7.5, 1.2 Hz, 1H), 7.17^minor^ (dd, *J* = 8.9, 5.4 Hz, 1H), 7.12^major^ (dd, *J* = 9.0, 5.4 Hz, 3H), 7.07^minor^ (d, *J* = 8.0 Hz, 1H), 6.98 (t, *J* = 8.7 Hz, 1H), 6.94 (d, *J* = 7.9 Hz, 1H), 6.88 (t, *J* = 8.7 Hz, 3H), 4.33–4.21 (m, 4H), 4.18 (d, *J* = 7.2 Hz, 1H), 4.09^minor^ (q, *J* = 7.3 Hz, 1H), 3.94^major^ (dd, *J* = 17.3, 6.4 Hz, 1H), 3.73–3.55 (m, 2H). ^13^C{^1^H} NMR (101 MHz, CDCl_3_, δ) 198.2, 197.4, 176.0, 175.5, 163.2, 160.7, 153.9, 153.5, 136.8, 136.7, 135.6, 134.5, 133.6, 133.5, 130.1 (d, *J*_C,F_ = 7.7 Hz), 129.7 (d, *J*_C,F_ = 7.7 Hz), 129.3, 129.0, 128.8 (2C), 128.2, 128.1, 126.1, 125.5, 125.1, 124.5, 124.2, 123.9, 115.5 (d, *J*_C,F_ = 21.2 Hz), 115.3 (d, *J*_C,F_ = 21.2 Hz), 110.9, 110.6, 48.2, 47.9, 42.3, 41.5, 41.5, 39.8. ^19^F{^1^H} NMR (376 MHz, CDCl_3_, δ) −114.53, −114.84. HRMS (MALDI-FT ICR) *m*/*z*: calcd for C_23_H_17_FNaO_3_ [M + Na]^+^: 383.1053, found: 383.1055.

*5-Chloro-3-(3-oxo-1,3-diphenylpropyl)benzofuran-2(3H)-one* (**15h**). The reaction mixture was heated at 50 °C (oil bath). The crude product was purified by flash chromatography (silica gel, hexane/ethyl acetate = 80:20) and isolated as a white solid (38 mg, yield: 75%), a mixture of diastereomers (dr = 63/37). ^1^H NMR (300 MHz, CDCl_3_, δ) 8.04^major^ (d, *J* = 8.1 Hz, 2H), 7.95^minor^ (d, *J* = 8.2 Hz, 2H), 7.73^minor^ (d, *J* = 8.1 Hz, 1H), 7.58^major^ (d, *J* = 6.7 Hz, 2H), 7.49^major^ (t, *J* = 7.8 Hz, 3H), 7.38^major^ (d, *J* = 6.6 Hz, 3H), 7.25 (d, *J* = 7.6 Hz, 4H), 7.19–7.07 (m, 8H), 7.03 (d, *J* = 7.3 Hz, 2H), 6.87^minor^ (d, *J* = 8.0 Hz, 2H), 6.79–6.67^major^ (m, 1H), 4.29–4.11 (m, 4H), 3.98^major^ (d, *J* = 3.9 Hz, 1H), 3.92^minor^ (d, *J* = 6.5 Hz, 1H), 3.67–3.49 (m, 2H). ^13^C{^1^H} NMR (75 MHz, CDCl_3_, δ) 198.3, 197.5, 176.0, 175.5, 153.7, 153.4, 139.9, 138.7, 136.7, 136.6, 133.3, 133.2, 129.2, 129.0, 128.6, 128.5, 128.3, 127.5, 127.3, 126.0, 125.7, 125.1, 124.5, 123.9, 123.7, 110.6, 110.3, 47.9, 47.6, 42.9, 41.9, 41.5, 39.5. HRMS (MALDI-FT ICR) *m*/*z*: calcd for C_23_H_17_ClNaO_3_ [M + Na]^+^: 399.0758, found: 399.0771.

## Data Availability

The data underlying this study are available in the published article and its Supporting Information.

## Supporting Information Available

Evaluated kinetic data, procedures for the preparation of starting materials, crystallographic data, and copies of NMR spectra of all new compounds (PDF)
Primary kinetic data (ZIP)
