# Supplementary material for: Lactone Enolates of Isochroman-3-ones and 2-Coumaranones: Quantification of Their Nucleophilicity in DMSO and Conjugate Additions to Chalcones
Source: J Org Chem. 2024 Apr 30;89(10):6915–28. doi: 10.1021/acs.joc.4c00277 (PMC11110064; doi:10.1021/acs.joc.4c00277)
Supplement: Supplementary file 1 — jo4c00277_si_001.pdf [file jo4c00277_si_001.pdf]

Supporting Information

Lactone Enolates of Isochroman-3-one and 2-Coumaranones: Quantification of Their Nucleophilicity in DMSO and Conjugate Additions to Chalcones

Mohammad Sadeq Mousavi,[a] Antonia Di Mola,[a] Giovanni Pierri,[a] Consiglia Tedesco,[a] Magenta J. Hensinger,[b] Aijia Sun,[b] Yilan Wang,[b] Peter Mayer,[b] Armin R. Ofial*,[b] and Antonio Massa*,[a]

[a] Dipartimento di Chimica e Biologia "A. Zambelli" Università degli Studi di Salerno, Via Giovanni Paolo II, 84084-Fisciano (SA) (Italy)

E-mail: amassa@unisa.it

[b] Department Chemie, Ludwig-Maximilians-Universität München, Butenandtstr. 5-13, 81377 München (Germany)

E-Mail: ofial@lmu.de

Table of Contents

1. General S2
2. Kinetics S3
   2.1 Kinetics of the reactions of the 3-isochromanone anion **4** with reference electrophiles S3
   2.2 Kinetics of the reactions of the 2-coumaranone anion **5** with reference electrophiles S9
3. Synthesis of Starting Materials S15
4. X-ray Crystallography S18
5. Copies of NMR Spectra S22
6. References S79

1. General

Unless otherwise noted, all chemicals, reagents and solvents for the performed reactions are commercially available. Isochroman-3-one was purchased from Fluorochem, substituted isochroman-3-one were prepared according to literature procedures. All the reactions were monitored by thin layer chromatography (TLC) on precoated silica gel plates (0.25 mm) and visualized by fluorescence quenching at 254 nm. Flash chromatography was carried out using silica gel 60 (70–230 mesh, Merck, Darmstadt, Germany). Yields are given for isolated products showing one spot on a TLC plate. The NMR spectra were recorded on Bruker DRX 600, 400, and 300 MHz spectrometers (600 MHz, ^{1}H, 150 MHz, ^{13}C; 400 MHz, ^{1}H, 100.6 MHz; ^{13}C, 300 MHz, ^{1}H, 75.5 MHz, ^{13}C, 250 MHz, ^{1}H, 62.5 MHz, ^{13}C). Internal reference was set to the residual solvent signals (δ_H 7.26 ppm, δ_C 77.16 ppm for $CDCl_3$). The ^{13}C NMR spectra were recorded under broad-band proton-decoupling. ^{1}HNMR data and HRMS are reported for all compounds. IR and ^{13}CNMR data are given only for unknown compounds. The following abbreviations are used to indicate the multiplicity in NMR spectra: s-singlet, d-doublet, t-triplet, q-quartet, dd-doublet of doublets, m-multiplet, brs-broad signal. High resolution mass spectra (HRMS) were acquired using a Bruker SolariX XR Fourier transform ion cyclotron resonance mass spectrometer (Bruker Daltonik GmbH, Bremen, Germany) equipped with a 7T refrigerated actively-shielded superconducting magnet. For ionization of the samples electrospray ionization (ESI) or MALDI was applied. IR spectra were recorded on a IR Bruker Vertex 70v spectrometer.

Kinetics. The kinetics of the reactions of the lactone enolates with the reference electrophiles **6** were followed by UV/Vis spectroscopy (Applied Photophysics SX.20 stopped-flow spectrophotometer). A constant temperature (20.0 ± 0.2 °C) was maintained through the use of a circulating bath cryostat. All solutions were freshly prepared under an atmosphere of dry argon by using dry DMSO (over molecular sieves, Acros Organics). Solutions of sodium 3-oxoisochroman-4-ide (**4**) in DMSO were prepared by deprotonation of 3-isochromanone (**2a**) with sodium hydride. Solutions of 2-oxo-2,3-dihydrobenzofuran-3-ide (**5**) in DMSO were prepared by deprotonation of 2-coumaranone (**3a**) with DBU (2.2 equiv.).

The kinetic measurements were initiated by mixing equal volumes of DMSO solutions of the nucleophiles and electrophiles. Selected reactions of **4** with the electrophiles **6** were measured with and without added crown ether (15-crown-5, 1.1 equiv. relative to the concentration of **4**). In general, nucleophile concentrations were at least ten times higher than electrophile concentrations to achieve pseudo-first order kinetics. Only for the reaction of **4** with **6a** (Table S2), the kinetic experiments were carried out by using the electrophile **6a** in excess (> 10 equiv.). The first-order rate constants k_{obs} (s^{-1}) could be obtained from the decay of the absorbance at or close to the absorption maximum of the reaction partner used in lower concentration by least squares fitting of the equation $A_t = A_0 \exp(-k_{obs}t) + C$ to the exponential absorption decay curve. Plots of k_{obs} (s^{-1}) versus the nucleophile concentration gave the second-order rate constants k_2 (M^{-1} s^{-1}) as slopes of the linear correlations. Error bars in these diagrams represent 5% uncertainty of concentrations and 10% uncertainty of k_{obs} values.

2. Kinetics

2.1 Kinetics of the reactions of the 3-isochromanone anion **4** with reference electrophiles

For kinetic measurements, 3-isochromanone (**2a**) was dissolved in DMSO and deprotonated by addition of 1.1 equiv. of sodium hydride (95% purity) to yield DMSO solutions of the nucleophilic **4** (counterion: Na^+). To characterize **4** in DMSO solution, the analogous experiment was performed in d_6-DMSO and the solution investigated by NMR spectroscopy (Figure S1), which showed quantitative deprotonation of 3-isochromanone.

To study the influence of the counterion (Na^+) on the reactivity of the lactone enolate **4**, the sodium cation was complexed by addition of 15-crown-5 in individual kinetic measurements of the reactions of **4** with **6c** and **6d**. The observed rate constants of these kinetic measurements did not deviate detectably from correlations of k_{obs} with [**4**] of measurements without addition of 15-crown-5. Thus, data from measurements with and without added crown ether were used undiscriminatingly to determine k_2 from linear k_{obs} vs [**4**] plots.

Spectroscopic characterization data of **4** (as depicted in Figures S1 and S2):

^{1}H NMR (400 MHz, d_6-DMSO): δ 6.73–6.65 (m, 2 H), 6.24–6.19 (m, 2 H), 4.61 (s, 2 H), 3.80 (s, 1 H). **^{13}C NMR** (101 MHz, d_6-DMSO): δ 166.6, 143.5, 126.9, 122.4, 122.3, 115.2, 113.6, 67.8, 67.6. **UV-Vis** (DMSO): λ_{max} = 336 nm.

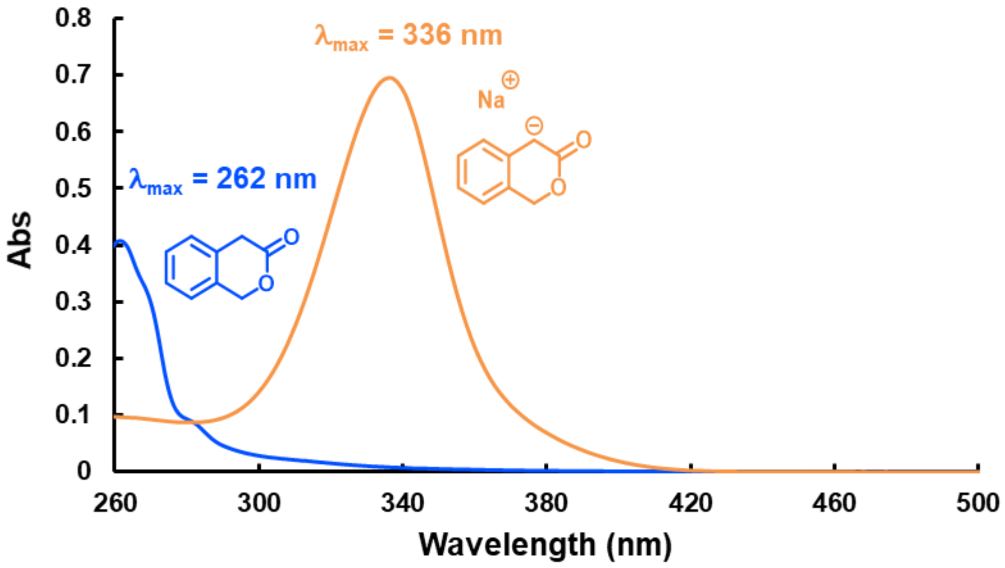

Figure S1. UV-Vis spectra of 3-isochromanone (**2**) and the corresponding lactone enolate **4** (counterion: Na^+) in DMSO.

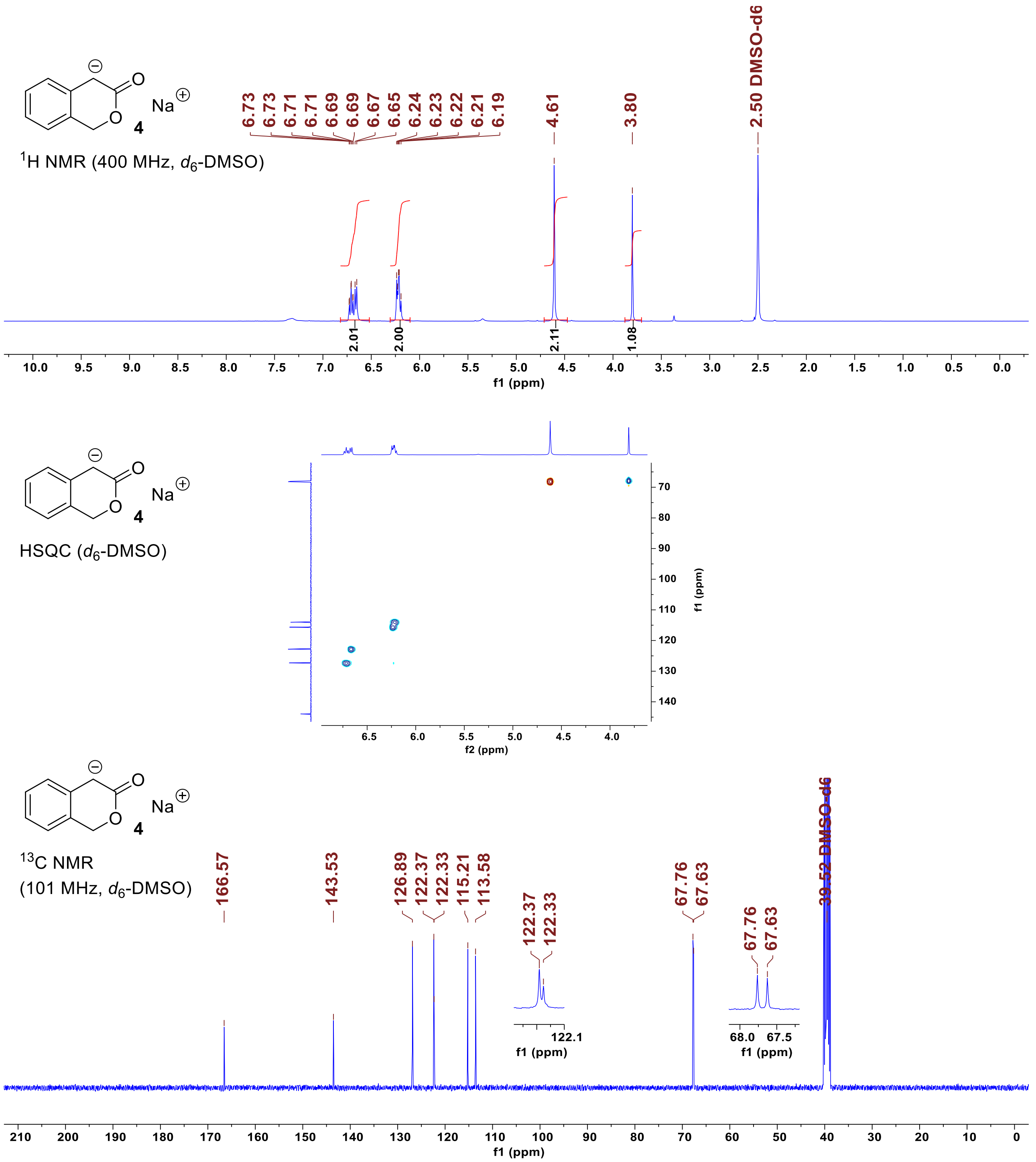

Figure S2. NMR spectroscopic characterization of **4** in d_6-DMSO (generated by adding 1.1 equiv sodium hydride to 3-isochromanone).

Table S1. Kinetics of the reaction of **4** with **6a** in DMSO at 20 °C (stopped-flow method, kinetics evaluated at λ = 370 nm).

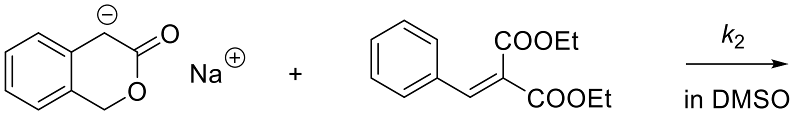

4 **6a**

[**4**] (M)	[**6a**] (M)	k_{obs} (s^{-1})
1.00×10^{-4}	1.00×10^{-3}	2.96×10^{-1}
1.00×10^{-4}	2.00×10^{-3}	7.50×10^{-1}
1.00×10^{-4}	3.00×10^{-3}	1.08
1.00×10^{-4}	4.00×10^{-3}	1.45

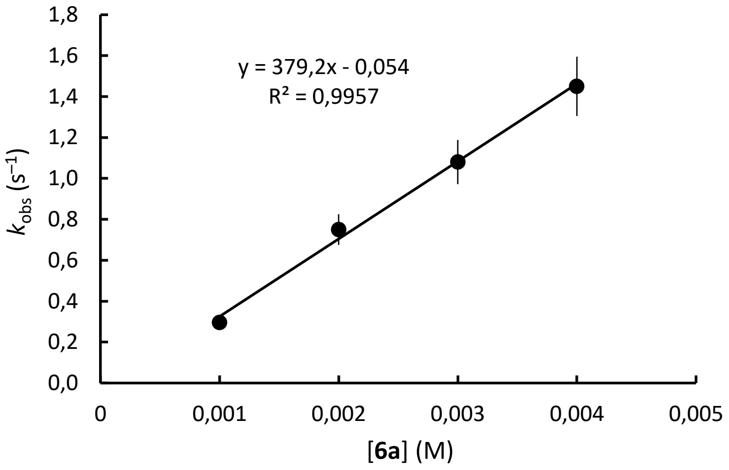

k_2= (3.79 ± 0.18) × 10^2 M^{-1} s^{-1}

Table S2. Kinetics of the reaction of **4** with **6b** in DMSO at 20 °C (stopped-flow method, kinetics evaluated at λ = 521 nm).

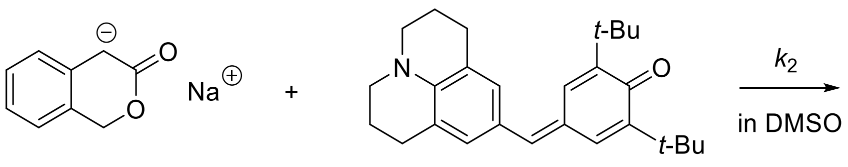

4 **6b**

[**4**] (M)	[**6b**] (M)	k_{obs} (s^{-1})
3.00×10^{-4}	3.00×10^{-5}	1.23
6.00×10^{-4}	3.00×10^{-5}	4.96
9.00×10^{-4}	3.00×10^{-5}	9.72
1.20×10^{-3}	3.00×10^{-5}	1.48×10^{1}
1.50×10^{-3}	3.00×10^{-5}	1.73×10^{1}

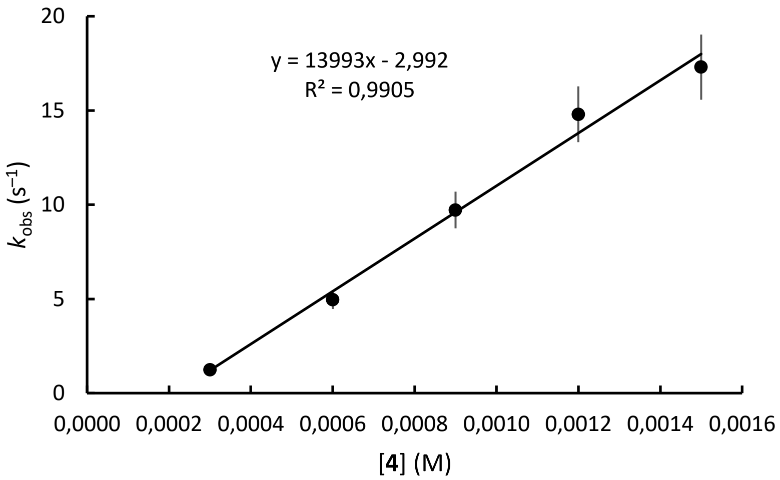

$\boldsymbol{k_2 = (1.40 \pm 0.08) \times 10^{4}\ M^{-1}\ s^{-1}}$

Table S3. Kinetics of the reaction of **4** with **6c** in DMSO at 20 °C (stopped-flow method, kinetics evaluated at λ = 486 nm).

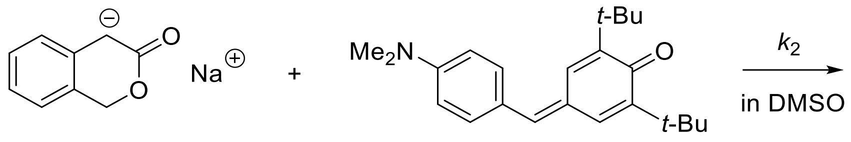

4 **6c**

[**4**] (M)	[15-crown-5] (M)	[**6c**] (M)	k_{obs} (s^{-1})
3.00×10^{-4}	3.30×10^{-4}	3.00×10^{-5}	1.58
6.00×10^{-4}	–	3.00×10^{-5}	8.09
9.00×10^{-4}	9.90×10^{-4}	3.00×10^{-5}	1.34×10^{1}
1.20×10^{-3}	–	3.00×10^{-5}	2.03×10^{1}
1.50×10^{-3}	1.65×10^{-3}	3.00×10^{-5}	2.60×10^{1}

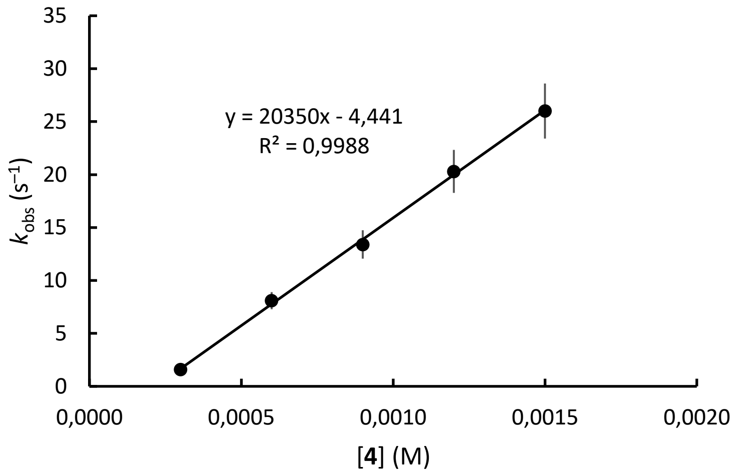

k_2**= (2.04 ± 0.04) × 10⁴ M⁻¹ s⁻¹**

Table S4. Kinetics of the reaction of **4** with **6d** in DMSO at 20 °C (stopped-flow method, kinetics evaluated at λ = 407 nm).

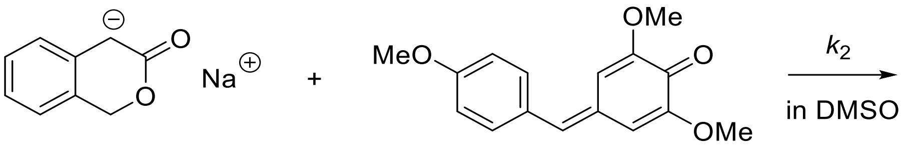

[**4**] (M)	[15-crown-5] (M)	[**6d**] (M)	k_{obs} (s^{-1})
3.00×10^{-4}	3.30×10^{-4}	3.00×10^{-5}	7.86
6.00×10^{-4}	–	3.00×10^{-5}	3.44×10^{1}
9.00×10^{-4}	9.90×10^{-4}	3.00×10^{-5}	4.66×10^{1}
1.20×10^{-3}	–	3.00×10^{-5}	7.06×10^{1}

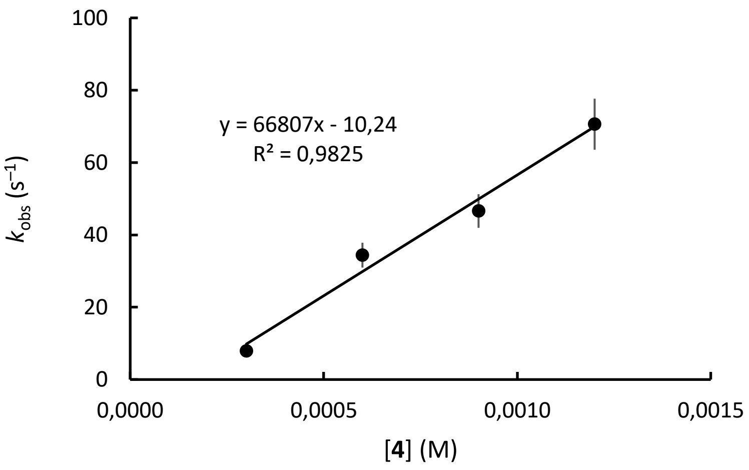

k_2 **= (6.68 ± 0.63) × 10⁴ M⁻¹ s⁻¹**

2.2 Kinetics of the reactions of the 2-coumaranone anion **5** with reference electrophiles

The successive deprotonation of 2-coumaranone **3a** (n = 3.57 × 10^{-6} mol) by a step-by-step addition of DBU (n_{end} = 8.0 × 10^{-6} mol, 2.24 equiv.) in DMSO (V_0 = 17.15 mL, V_{end} = 17.65 mL) was monitored at 20 °C by UV-Vis spectroscopy at 286 nm (Figure S3).

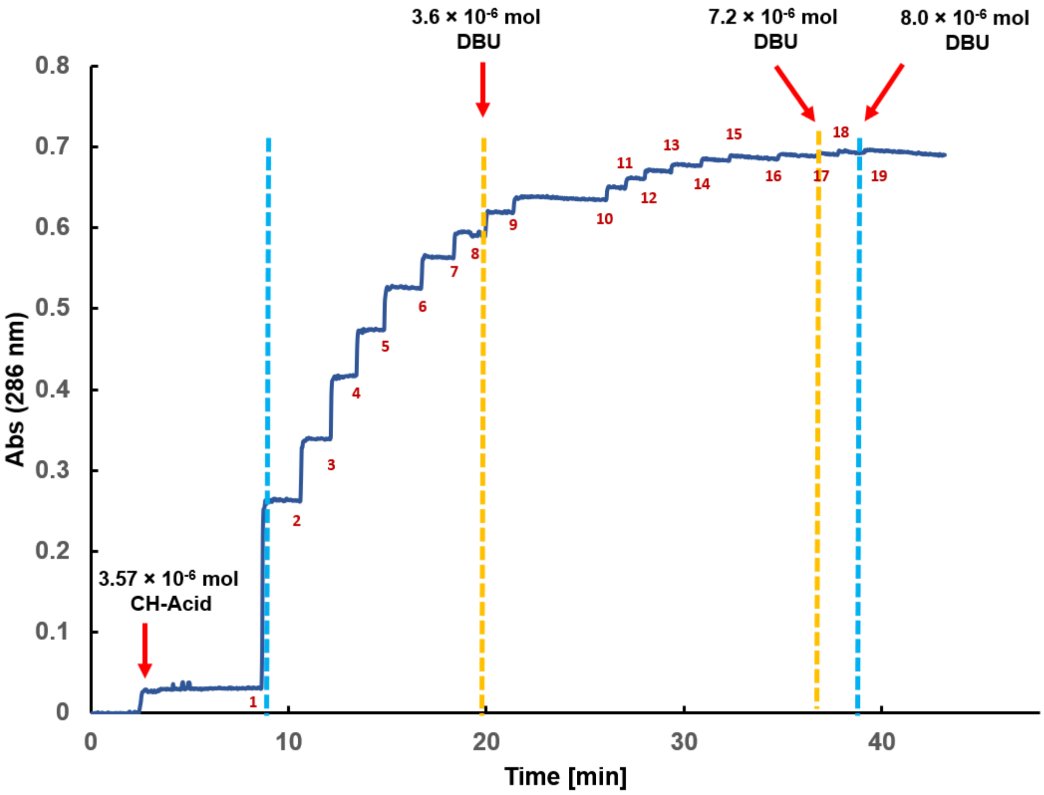

Figure S3. Formation of the anion **4** through deprotonation of 2-coumaranone **3a** (3.57 × 10^{-6} mol) by DBU in DMSO at 20 °C. Yellow dashed lines mark the addition of n equivs of DBU. Two blue dashed lines represent the start and the end of the DBU addition process, respectively.

Table S5. Kinetics of the reaction of **5** with **6c** in DMSO at 20 °C (stopped-flow method, kinetics evaluated at λ = 486 nm).

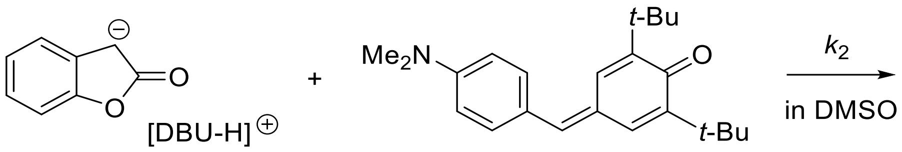

5 **6c**

[**5**] (M)	[**6c**] (M)	k_{obs} (s^{-1})
5.13×10^{-4}	5.18×10^{-5}	1.99×10^{-2}
1.04×10^{-3}	5.18×10^{-5}	4.77×10^{-2}
1.55×10^{-3}	5.18×10^{-5}	7.51×10^{-2}
2.08×10^{-3}	5.18×10^{-5}	1.01×10^{-1}

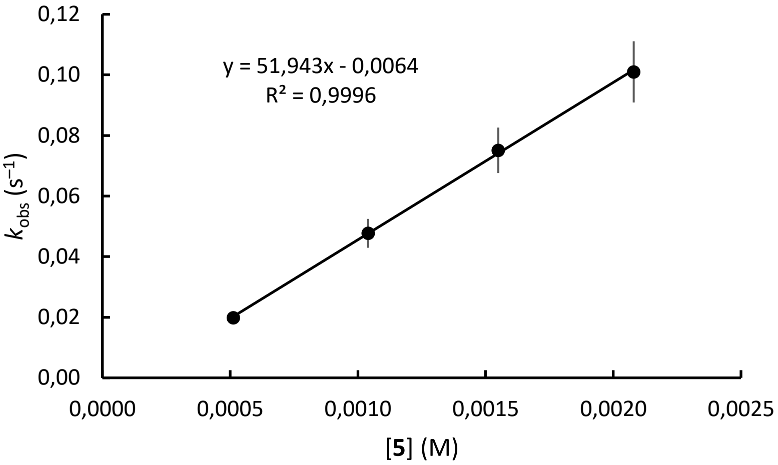

$\mathbf{k_2 = (5.19 \pm 0.07) \times 10^1\ M^{-1}\ s^{-1}}$

Table S6. Kinetics of the reaction of **5** with **6e** in DMSO at 20 °C (stopped-flow method, kinetics evaluated at λ = 393 nm).

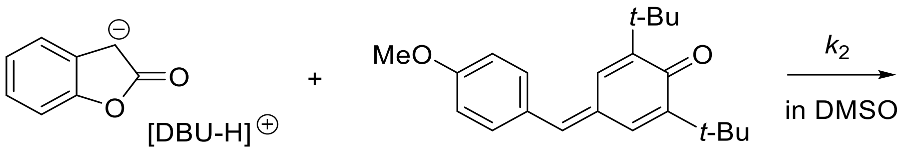

5 **6e**

[**5**] (M)	[**6e**] (M)	k_{obs} (s^{-1})
5.02×10^{-4}	4.97×10^{-5}	1.36×10^{-1}
1.00×10^{-3}	4.97×10^{-5}	2.33×10^{-1}
1.50×10^{-3}	4.97×10^{-5}	4.83×10^{-1}
2.00×10^{-3}	4.97×10^{-5}	6.52×10^{-1}

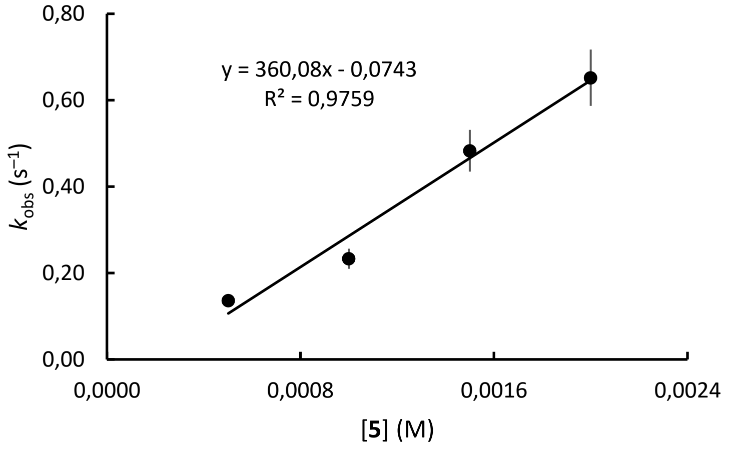

$$k_2 = (3.60 \pm 0.40) \times 10^2 \ M^{-1} \ s^{-1}$$

Table S7. Kinetics of the reaction of **5** with **6f** in DMSO at 20 °C (stopped-flow method, kinetics evaluated at λ = 370 nm).

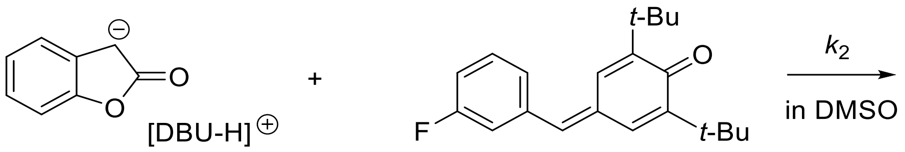

5 **6f**

[**5**] (M)	[**6f**] (M)	k_{obs} (s^{-1})
4.98×10^{-4}	5.00×10^{-5}	6.88×10^{-1}
1.01×10^{-3}	5.00×10^{-5}	2.34
1.50×10^{-3}	5.00×10^{-5}	4.11
2.00×10^{-3}	5.00×10^{-5}	5.20

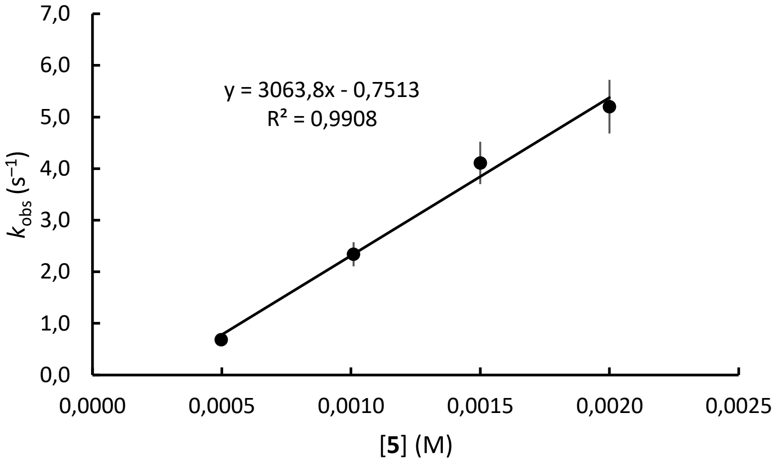

$\boldsymbol{k_2 = (3.06 \pm 0.21) \times 10^3\ M^{-1}\ s^{-1}}$

Table S8. Kinetics of the reaction of **5** with **6g** in DMSO at 20 °C (stopped-flow method, kinetics evaluated at λ = 374 nm).

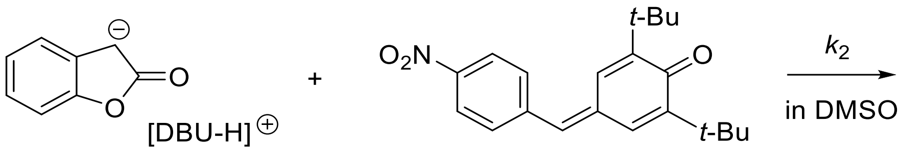

5 **6g**

[**5**] (M)	[**6g**] (M)	k_{obs} (s^{-1})
5.02×10^{-4}	5.01×10^{-5}	8.42
1.00×10^{-3}	5.01×10^{-5}	1.29×10^{1}
1.50×10^{-3}	5.01×10^{-5}	2.06×10^{1}
2.00×10^{-3}	5.01×10^{-5}	2.77×10^{1}

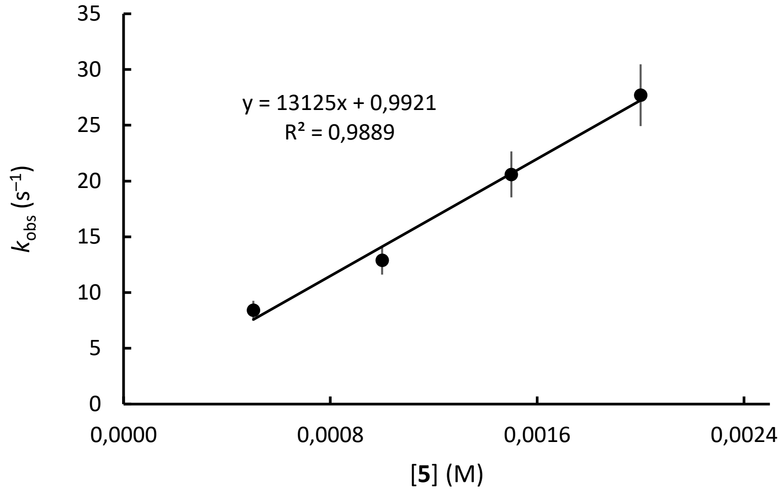

$$k_2 = (1.31 \pm 0.10) \times 10^4\ M^{-1}\ s^{-1}$$

Table S9. Kinetics of the reaction of **5** with **6h** in DMSO at 20 °C (stopped-flow method, kinetics evaluated at λ = 533 nm).

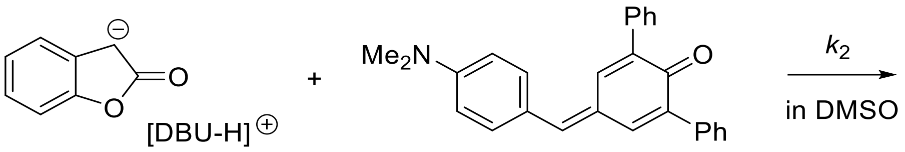

5 **6h**

[**5**] (M)	[**6h**] (M)	k_{obs} (s^{-1})
5.04×10^{-4}	5.01×10^{-5}	9.24
1.01×10^{-3}	5.01×10^{-5}	2.95×10^{1}
1.51×10^{-3}	5.01×10^{-5}	4.35×10^{1}
2.02×10^{-3}	5.01×10^{-5}	5.98×10^{1}

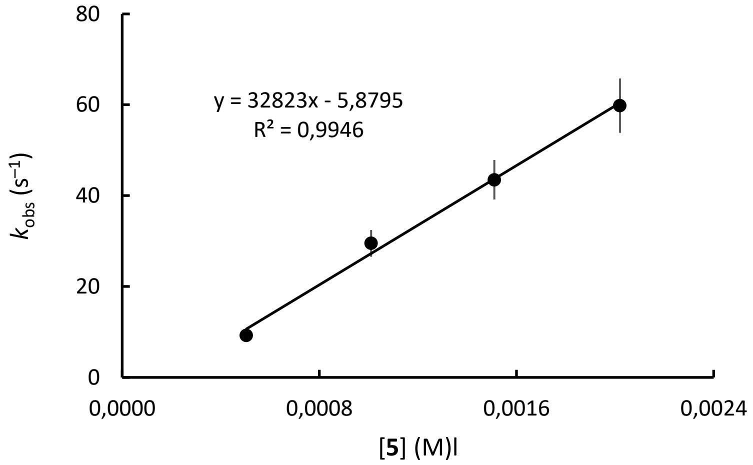

$\boldsymbol{k_2 = (3.28 \pm 0.17) \times 10^4\ M^{-1}\ s^{-1}}$

3. Synthesis of Starting Materials

6-Chloroisochroman-3-one (2e).

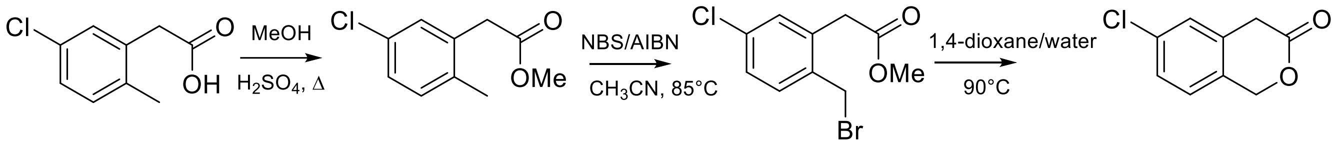

Methyl 2-(2-(bromomethyl)-5-chlorophenyl) acetate was prepared as reported in reference.[S1] A solution of 277.5 mg of this compound (1 mmol, 1 eq.) in 1,4 dioxane/water (2 mL, 1/1) was refluxed in an oil bath at 90°C for overnight. Then solvent was removed under reduced pressure and the crude was taken up ethyl acetate. Purification by on silica gel (petroleum ether/ethyl acetate, 10:1) afforded the product as white solid. Yield 91 % (166 mg). Mp. 102–103 °C, **^{1}H NMR** (400 MHz, $CDCl_3$) δ 7.26 (d, *J* = 8.4 Hz, 1 H), 7.19 (d, *J* = 6.8 Hz, 1 H), 7.15 (s, 1 H), 5.25 (s, 2 H), 3.66 (s, 2 H). **^{13}C NMR** (100 MHz, $CDCl_3$) δ 169.8, 134.5, 132.2, 129.9, 127.5, 127.1, 126.0, 69.4, 35.8. IR (neat): 2918, 1748, 1607, 743 cm^{-1}. **HRMS** (MALDI-FT ICR): *m/z* calcd. for $C_9H_7ClNaO_2^+$ $[M+Na]^+$: 205.0026; found: 205.0024.

6-Methoxyisochroman-3-one (2c).

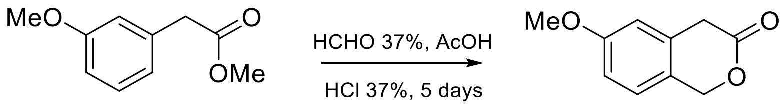

Prepared as reported in literature.[S2] White solid, yield: 37% (150mg). M.p. and spectroscopic data were found in agreement with those reported in literature.[S2] ^{1}H NMR (400 MHz, $CDCl_3$) δ 7.17 (d, *J* = 8.3 Hz, 1 H), 6.83 (dd, *J* = 8.3, 2.5 Hz, 1 H), 6.76 (d, *J* = 2.5 Hz, 1 H), 5.26 (s, 2 H), 3.83 (s, 3 H), 3.68 (s, 2 H).

Synthesis of 7-substituted isochroman-3-ones

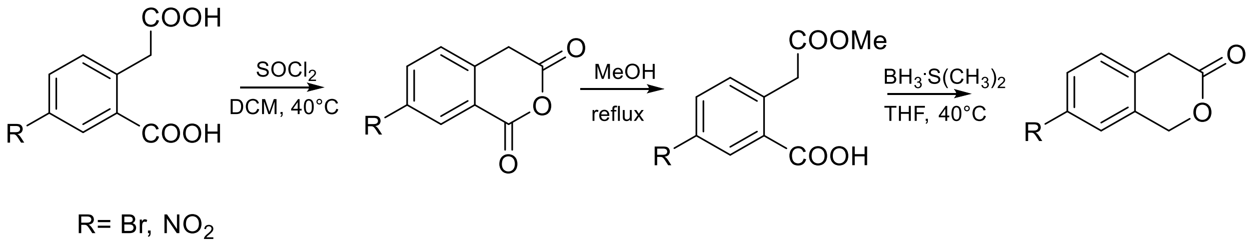

Prepared as reported in literature.[S3]

7-Bromoisochroman-3-ones (2b). White solid. Yield: 81% (100 mg). M.p. and spectroscopic data were found in agreement with those reported in literature. **^{1}H NMR** (400 MHz, $CDCl_3$) δ 7.54 (d, *J* = 8.3 Hz, 1 H), 7.47 (s, 1 H), 7.17 (d, *J* = 8.0 Hz, 1 H), 5.34 (s, 2 H), 3.73 (s, 2 H).

7-Nitroisochroman-3-ones (2d). White solid. Yield: 72% (130 mg). M.p. and spectroscopic data were found in agreement with those reported in literature.[S4] **^{1}H NMR** (400 MHz, $CDCl_3$) δ 8.31 (dd, *J* = 8.3, 2.3 Hz, 1 H), 8.23 (d, *J* = 2.2 Hz, 1 H), 7.50 (d, *J* = 8.3 Hz, 1 H), 5.48 (s, 2 H), 3.91 (s, 2 H).

Synthesis of 8-fluoroisochroman-3-one (2f).

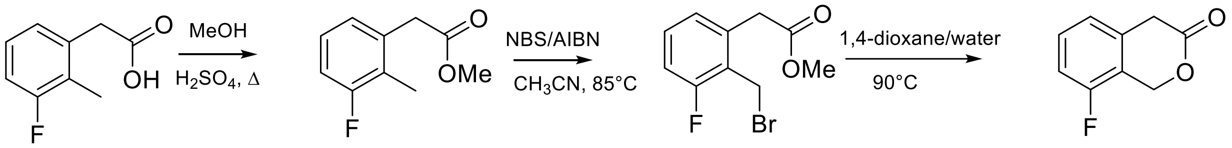

Concentrated H_2SO_4 (0.5 mL) was added to a solution of 2-(3-fluoro-2-methylphenyl)acetic acid (900 mg, 5.35 mmol) in MeOH (15 mL). The mixture was refluxed by heating with an oil bath overnight. The solvent was removed under reduced pressure, the crude rinsed up with ethyl acetate and washed twice with $NaHCO_3$ saturated solution and brine. Pale oil (898 mg, yield: 98%) **^{1}H NMR** (300 MHz, $CDCl_3$) δ 7.18–7.07 (m, 1 H), 7.02–6.92 (m, 2 H), 3.71 (s, 3 H), 3.68 (s, 2 H), 2.23 (s, 3 H). **^{13}C NMR** (100 MHz, $CDCl_3$) δ 171.5, 161.4 (d, $J_{C,F}$ = 243.6 Hz), 135.2, 126.8 (d, $J_{C,F}$ = 9.0 Hz), 125.8 (d, $J_{C,F}$ = 3.2 Hz), 124.2 (d, $J_{C,F}$ = 16.7 Hz), 114.1 (dd, $J_{C,F}$ = 23.1, 2.3 Hz), 52.1, 38.8, 10.8. ^{19}F{^{1}H} NMR (376 MHz, $CDCl_3$): δ –116.15. IR (neat): 2976, 1739, 1635, 928 cm^{-1}. HRMS (MALDI-FT ICR): *m/z* calcd. for $C_{10}H_{11}FNaO_2^+$ $[M+Na]^+$: 205.0536; found: 205.0554.

The obtained methyl ester (900 mg, 4.95mmol, 1.0 equiv.) was dissolved in CH_3CN (10 mL) and treated with *N*-bromosuccinimide (NBS, 968 mg, 1.2 equiv.) and *α,α'*-azoisobutyronitrile (AIBN, 81 mg, 0.1 equiv.) at 85°C (oil bath) overnight. Then, the mixture was extracted by diethyl ether/water to remove succinimide and, without any other purification, the crude product (1.0 g, 3.84 mmol, 1 eq.) was refluxed at 90 °C (oil bath) overnight in 1,4 dioxane/water (12 mL, 1/1). Then solvent was removed under reduced pressure and the crude was taken up with ethyl acetate. Purification by on silica gel (petroleum ether/ethyl acetate, 10:1) afforded the product as a white solid. Yield 64% (overall of two steps, 3.5 mmol, 584 mg). Mp. 111-112 °C. **^{1}H NMR** (400 MHz, $CDCl_3$) δ 7.32 (td, *J* = 8.0, 5.4 Hz, 1 H), 7.00–7.04 (m, 2 H), 5.42 (s, 2 H), 3.72 (s, 2 H). **^{13}C NMR** (100 MHz, $CDCl_3$) δ 169.9, 158.2 (d, $J_{C,F}$ = 248.7 Hz), 133.6 (d, $J_{C,F}$ = 3.7 Hz), 130.4 (d, $J_{C,F}$ = 8.1 Hz), 122.8 (d, $J_{C,F}$ = 3.7 Hz), 118.8 (d, $J_{C,F}$ = 16.8 Hz), 114.3 (d, $J_{C,F}$ = 20.5 Hz), 64.0, 35.7. **^{19}F{^{1}H} NMR** (376 MHz, $CDCl_3$): δ –120.20. **IR** (neat): 2947, 1745, 1632, 932 cm^{-1}. **HRMS** (MALDI-FT ICR): *m/z* calcd. for $C_9H_7FNaO_2^+$ $[M+Na]^+$: 189.0323; found: 183.0355.

Synthesis of 2-oxo-2,3-dihydrobenzofuran-5-yl acetate (3b).

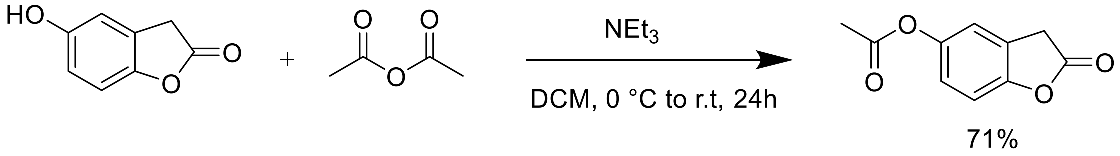

Acetic anhydride (110 µL, 1.1 mmol, 1.1 eq.), and triethylamine (209 µL, 1.5 mmol, 1.5 eq.) were added to a solution of 5-hydroxybenzofuran-2(3*H*)-one (150 mg, 1 mmol, 1 eq.) in DCM (4 mL). The reaction mixture was stirred for 12 h. The solution was washed with 1N HCl, dried over Na_2SO_4, and purified by silica gel column chromatography using a linear gradient of 0-20% ethyl acetate in hexane to give the product in 91% yield (172mg). **^{1}H NMR** (400 MHz, $CDCl_3$) δ 7.15 (dd, *J* = 8.6, 1.6 Hz, 1 H), 7.11 (s, 1 H), 7.07 (d, *J* = 8.6 Hz, 1 H), 3.82 (s, 2 H), 2.36 (s, 3 H). **^{13}C NMR** (75 MHz, $CDCl_3$) δ 173.8, 169.6, 151.9, 146.7, 124.1, 121.9, 118.5, 111.1, 33.1, 20.9. **IR** (neat): 2940, 1780, 1761, 756 cm^{-1}. **HRMS** (MALDI-FT ICR): *m/z* calcd. for $C_{10}H_8NaO_4^+$ $[M+Na]^+$: 215.0315; found: 215.0321.

Synthesis of 5-chlorobenzofuran-2(3H)-one (3c).

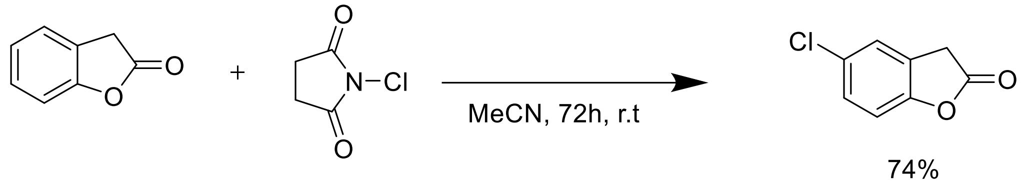

Prepared as reported in literature.[S5] 2-Benzofuranone **3a** (2-coumaranone, 192 mg, 1.43 mmol), *N*-chlorosuccinimide (572 mg, 4.26 mmol), and anhydrous CH_3CN (5 mL) were mixed and the solution was stirred for 72 hours. CH_3CN was then evaporated and the residue was diluted with ether. The organic solution was washed with one portion of $Na_2S_2O_3$ (aq) and 1 ml of H_2O, then dried over Na_2SO_4. After removal of the Na_2SO_4, the solvent was evaporated and the residue was purified by silica gel column chromatography using a linear gradient of 0-50% ethyl acetate in hexane to give the product in 70% yield (168mg). **^{1}H NMR** (400 MHz, $CDCl_3$) δ 7.22 (td, *J* = 5.5, 4.6, 2.8 Hz, 2 H), 6.97 (d, *J* = 9.2 Hz, 1 H), 3.68 (s, 2 H).

4. X-ray Crystallography

Slow diffusion of pentane into a dichloromethane solution of the diasteromeric mixture of **7a** at 4 °C (fridge) delivered a small amount of diastereomerically pure cubic crystals of **7a**, which were characterized by X-ray crystallography (bv020). The X-ray intensity data of **7a**-major (bv020) were measured on a Bruker D8 Venture TXS system equipped with a multilayer mirror monochromator and a Mo Kα rotating anode X-ray tube (λ = 0.71073 Å). The frames were integrated with the Bruker SAINT software package.[S6] Data were corrected for absorption effects using the Multi-Scan method (SADABS).[S7] The structure was solved and refined using the Bruker SHELXTL Software Package.[S8] All hydrogen atoms have been calculated in ideal geometry riding on their parent atoms. The ring disorders have been described by split models. The ratios of site occupation factors refined to 0.62/0.38 and 0.56/0.44. The figures have been drawn at the 25% ellipsoid probability level.[S9] In the case of disorder the less-occupied parts have been neglected for the figures.

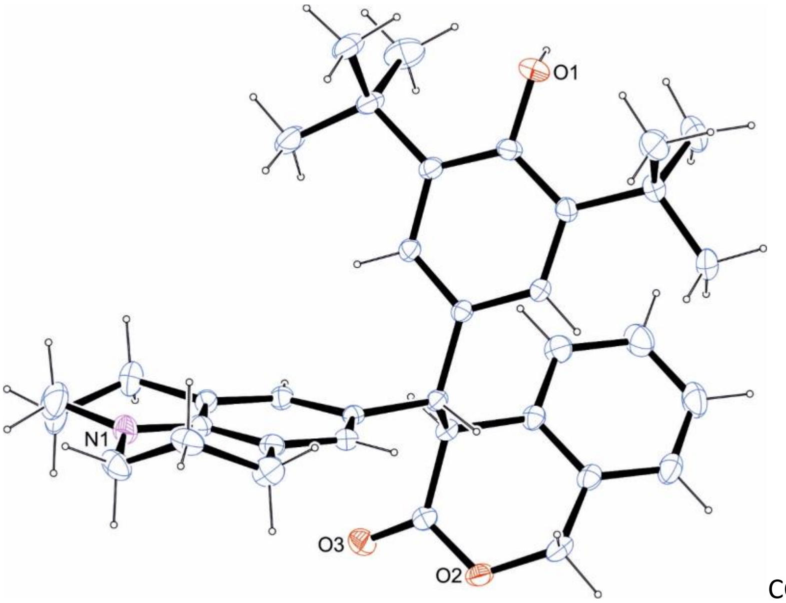

CCDC 2320508 (bv020)

Table S10. Crystallographic data for **7a**-major.

	**7a** (bv020)
net formula	$C_{36}H_{43}NO_3$
M_r/g mol^{-1}	537.71
crystal size/mm	0.140 × 0.110 × 0.090
T/K	173.(2)
radiation	MoKα
diffractometer	'Bruker D8 Venture TXS'
crystal system	triclinic
space group	'P -1'
a/Å	10.8226(4)
b/Å	11.4692(5)
c/Å	13.4846(5)
α/°	109.2660(10)
β/°	103.3470(10)
γ/°	90.3050(10)
V/Å^3	1531.32(10)
Z	2
calc. density/g cm^{-3}	1.166
μ/mm^{-1}	0.073
absorption correction	Multi-Scan
transmission factor range	0.93–0.99
refls. measured	26526
R_{int}	0.0389
mean σ(I)/I	0.0346
θ range	3.156–26.372
observed refls.	5044
x, y (weighting scheme)	0.0537, 0.7416
hydrogen refinement	constr
refls in refinement	6236
parameters	407
restraints	7
$R(F_{obs})$	0.0517
$R_w(F^2)$	0.1398
S	1.050
shift/error$_{max}$	0.001
max electron density/e Å^{-3}	0.393
min electron density/e Å^{-3}	−0.334

Crystals of the compound **14b-1** suitable for single crystal X-ray diffraction analysis were obtained by slow evaporation, dissolving 5 mg of the compound in dichloromethane/hexane (1 ml, 1:5). A colorless needle-like single crystal with dimensions of 0.13 mm × 0.11 mm × 0.04 mm was selected and mounted on a cryoloop with paratone oil and measured at 100 K.

Crystals of the compound **15d-1** suitable for single crystal X-ray diffraction analysis were obtained by slow evaporation, dissolving 10 mg of the compound in ether/isopropyl alcohol (1 ml 1:4). A colorless needle-like single crystal with dimensions of 0.25 mm × 0.11 mm × 0.03 mm was selected and mounted on a cryoloop with paratone oil and measured at 100 K.

Both crystals were measured using Cu-Kα radiation (λ= 1.54178 Å) on a Bruker D8 QUEST diffractometer equipped with a PHOTON detector. Data indexing was performed using APEX3.1 Data integration and

reduction were performed using SAINT.2 Absorption correction was performed by multi-scan method in SADABS.3 The structures were solved by direct methods using SHELXS4 and refined by means of full matrix least-squares based on F2 using the program SHELXL5 and OLEX26 as GUI. Non-hydrogen atoms were refined anisotropically, hydrogen atoms were positioned geometrically and included in structure factors calculations but not refined. ORTEP diagrams (Figure S4) were drawn using OLEX2. Relevant crystallographic data are reported in Table S11.

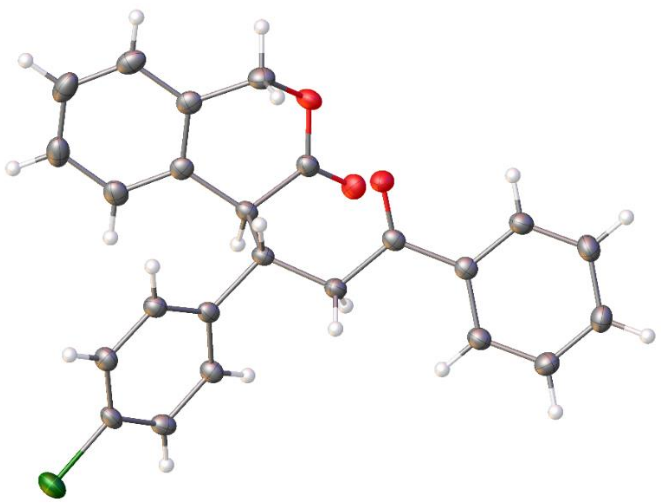

(a) **14b-1** (CCDC 2314553)

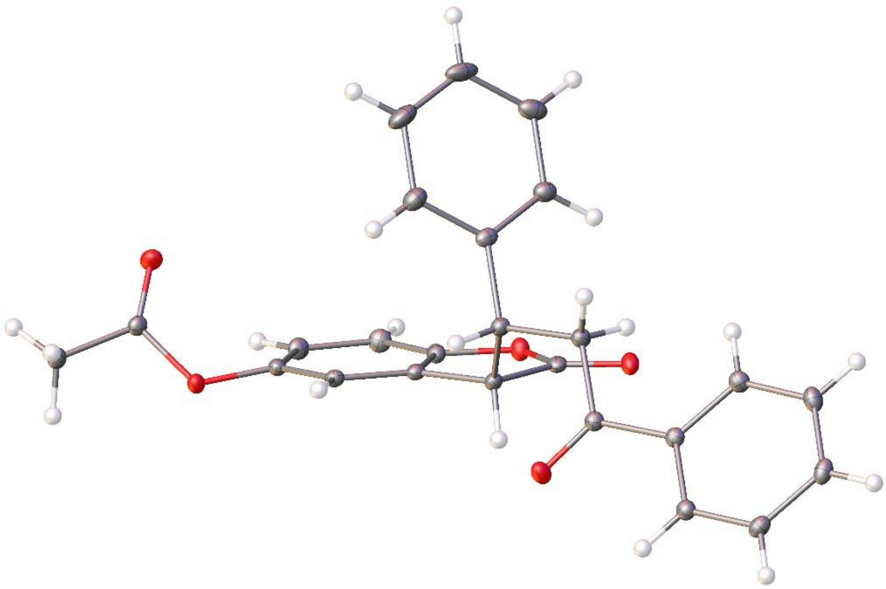

(b) **15d-1** (CCDC 2314554)

Figure S4. ORTEP drawings of (a) **14b-1** and (b) **15d-1**. Atom types: C grey, H white, O red and Cl green. Ellipsoids are drawn at 20% probability level.[S10-S15]

Table S11. Crystallographic data for **14b-1 and 15d-1**.

	**14b-1**	**15d-1**
T (K)	100	100
Formula	$C_{24}H_{19}ClO_3$	$C_{25}H_{20}O_5$
Formula weight	390.84	400.41
System	monoclinic	monoclinic
Space group	$P\ 2_1/c$	$P\ 2_1/n$
a (Å)	13.6338(5)	9.3036(2)
b (Å)	11.7590(4)	20.4381(5)
c (Å)	13.1680(4)	10.6038(3)
α (°)	90	90
β (°)	114.7100(11)	99.6619(9)
γ (°)	90	90
V (Å^3)	1917.79(11)	1987.69(9)
Z	4	4
Dx (g cm^{-3})	1.354	1.338
λ (Å)	1.54178	1.54178
μ (mm^{-1})	1.944	0.761
F_{000}	816.0	840.0
R1 (I > 2σI)	0.0500(3142)	0.0323(3392)
$_{w}R_2$	0.1406(3772)	0.0786(3805)
N. of param.	253	273
GooF	1.022	1.034
ρ_{min}, ρ_{max} (eÅ^{-3})	-0.48, 0.35	-0.17, 0.28

5. Copies of NMR Spectra

Methyl 2-(3-fluoro-2-methylphenyl)acetate

^{1}H NMR (400 MHz, $CDCl_3$)

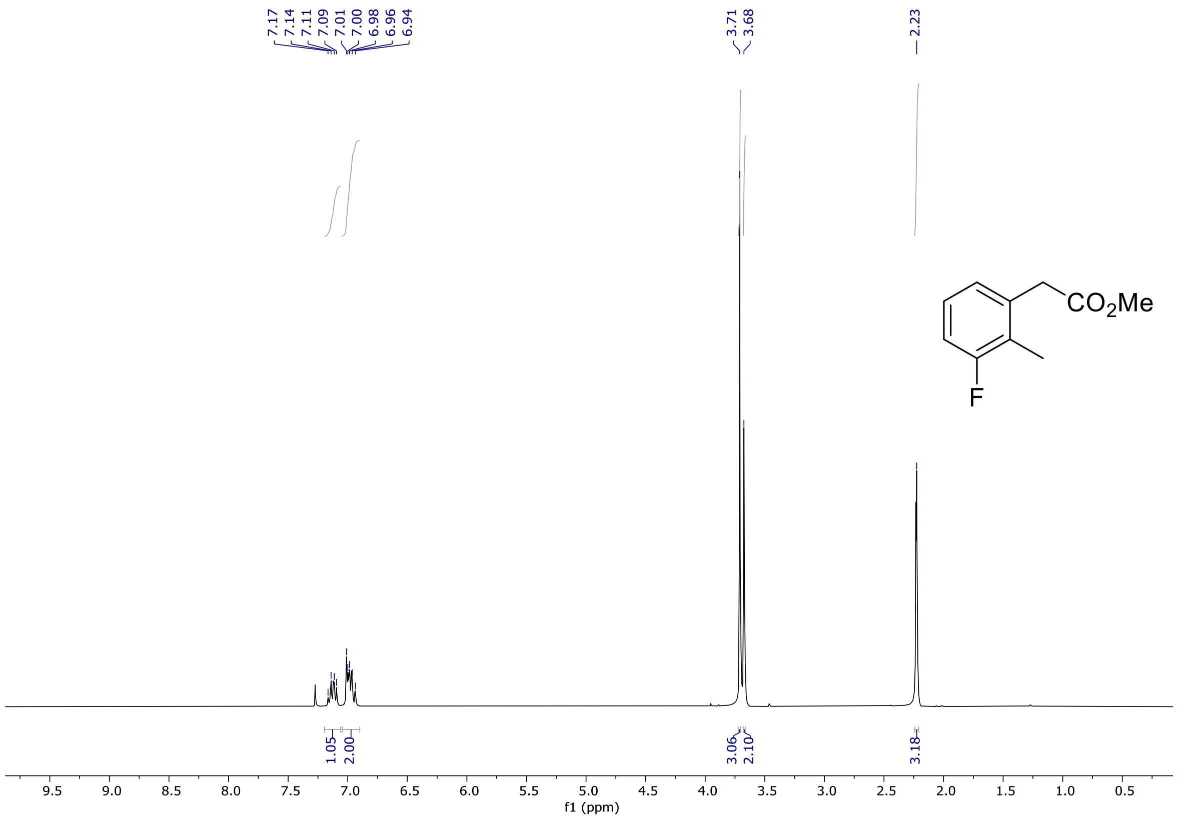

$^{13}C\{^{1}H\}$ NMR (100 MHz, $CDCl_3$)

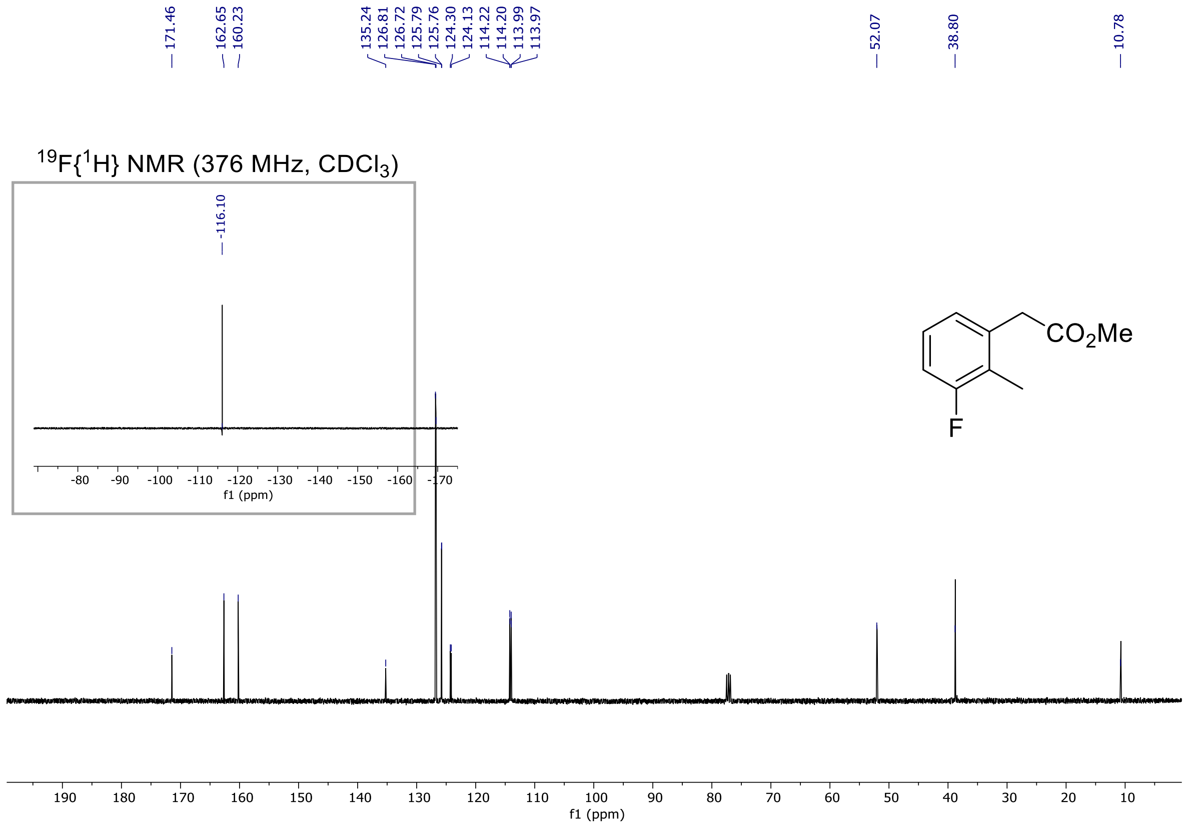

6-Chloroisochroman-3-one

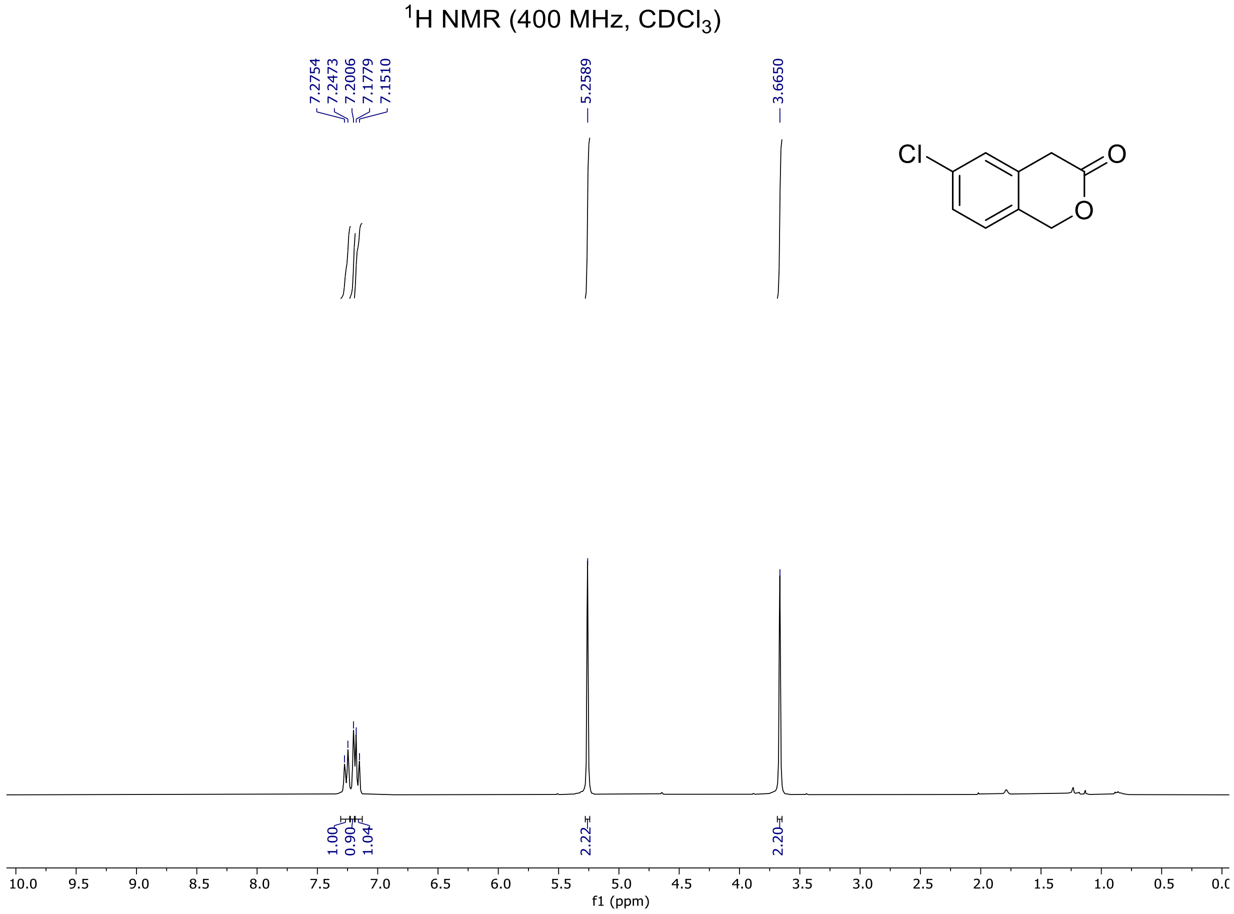

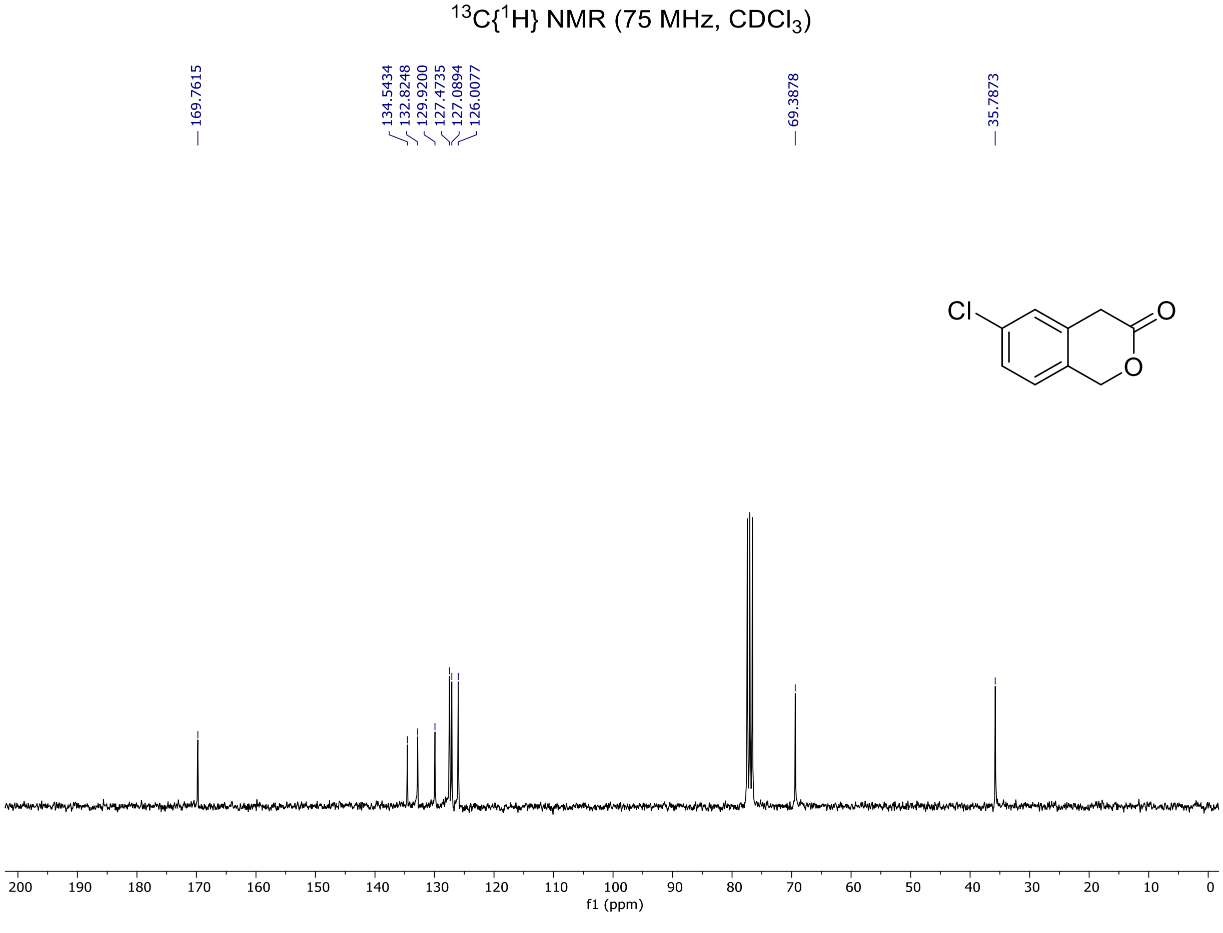

8-Fluoroisochroman-3-one

^{1}H NMR (400 MHz, $CDCl_3$)

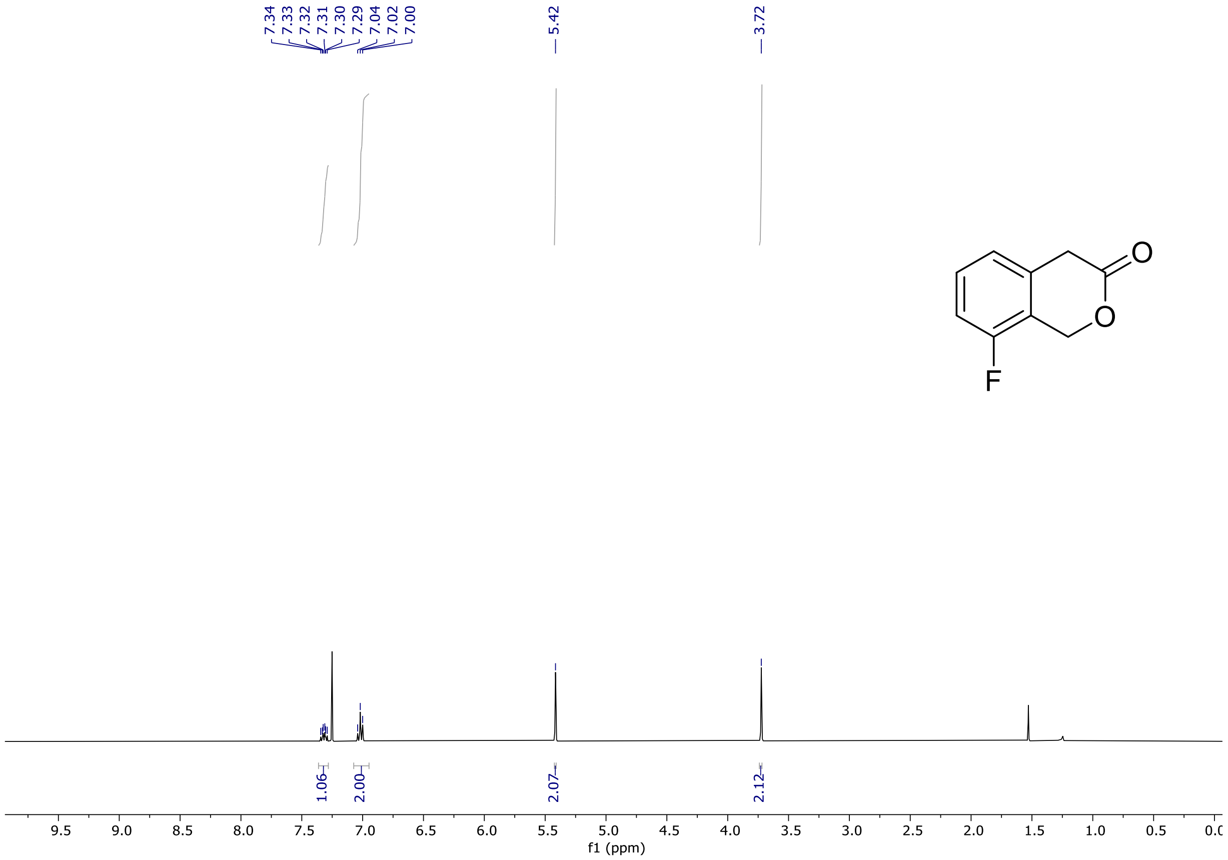

$^{13}C\{^{1}H\}$ NMR (100 MHz, $CDCl_3$)

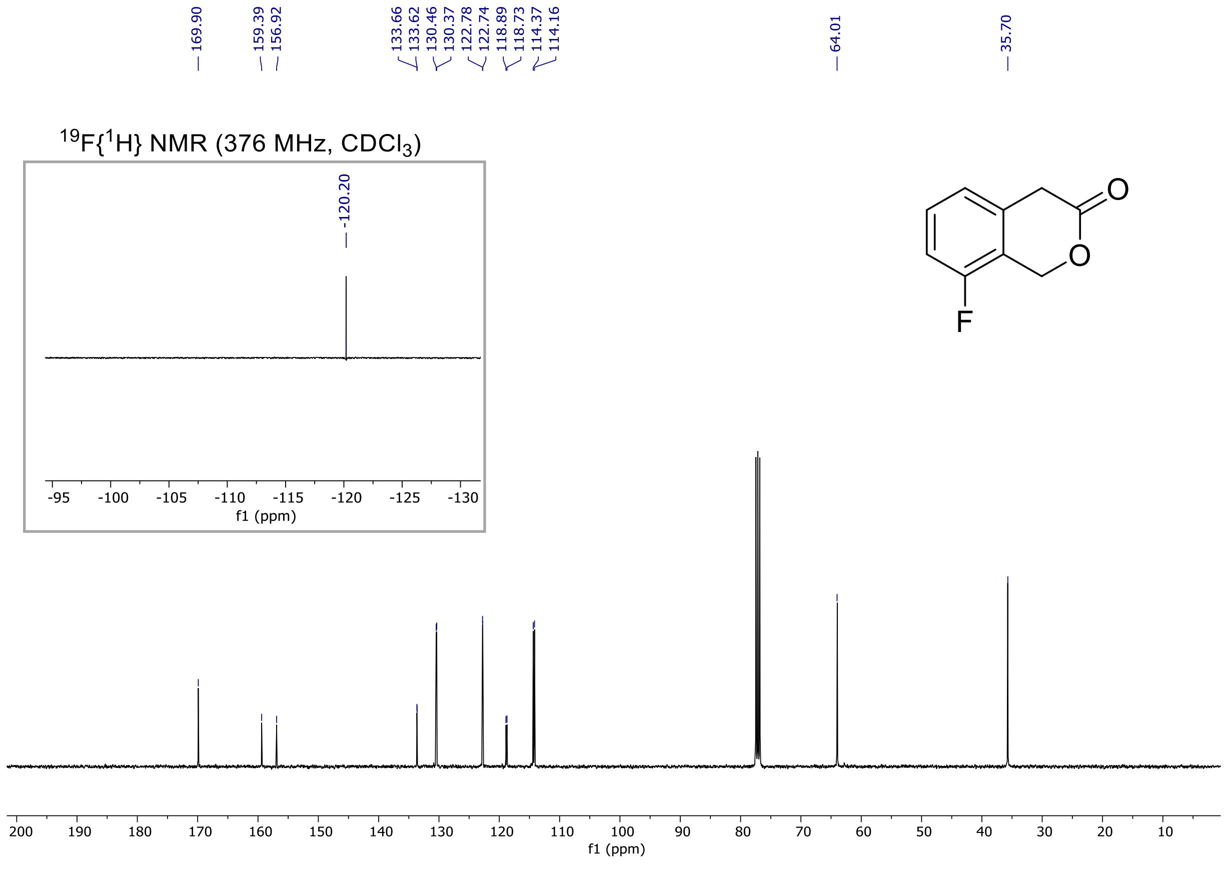

4-((3,5-Di-*tert*-butyl-4-hydroxyphenyl)(julolidin-9-yl)-λ³-methyl)-4λ³-isochroman-3-one (7a)

^{1}H NMR (400 MHz, $CDCl_3$) of **7a**-major:

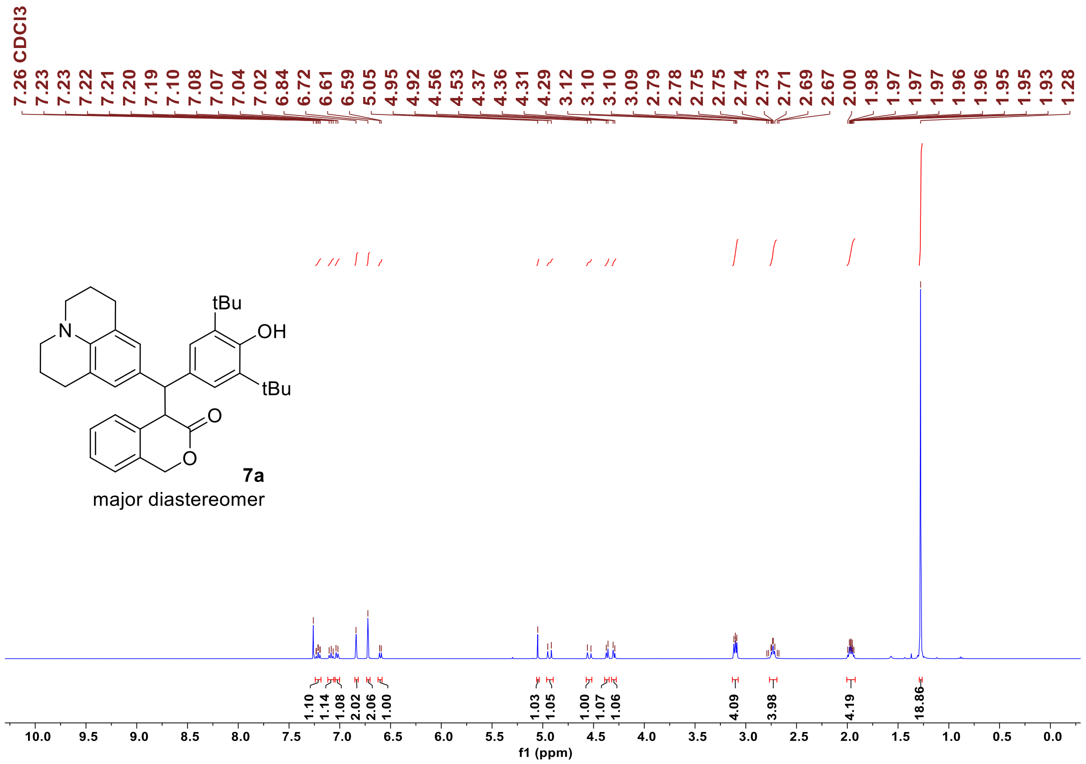

^{13}C{^{1}H} NMR (101 MHz, $CDCl_3$) of **7a**-major:

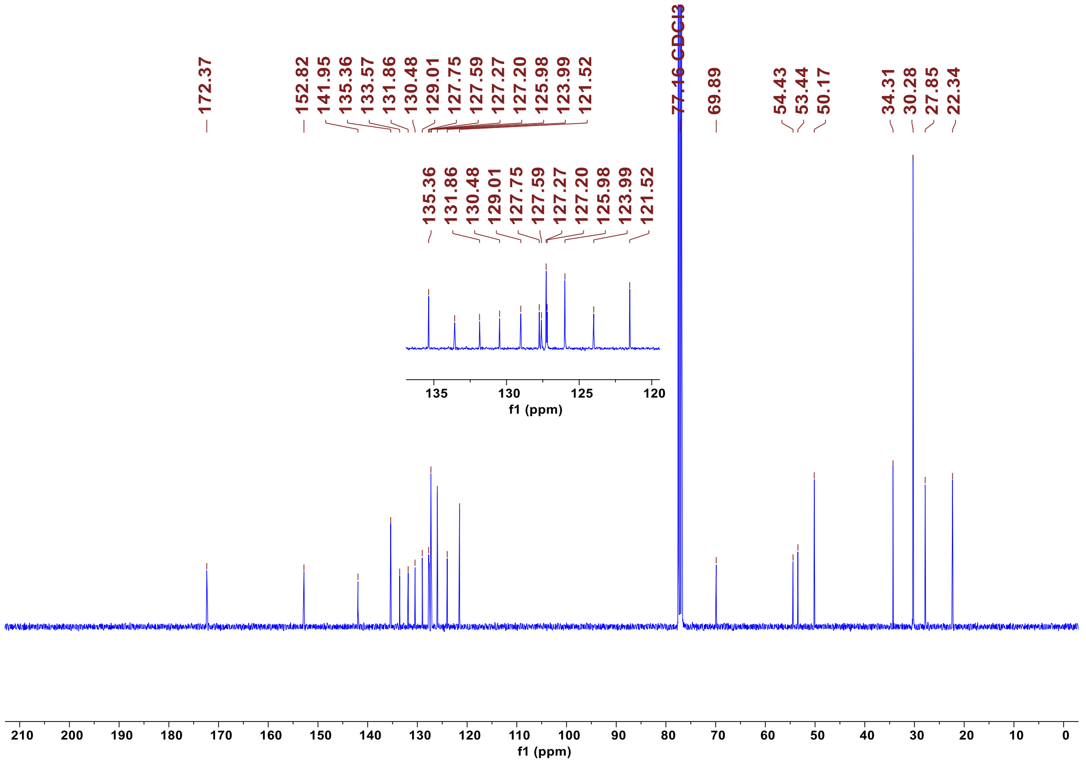

HSQC spectrum of **7a**-major (in $CDCl_3$):

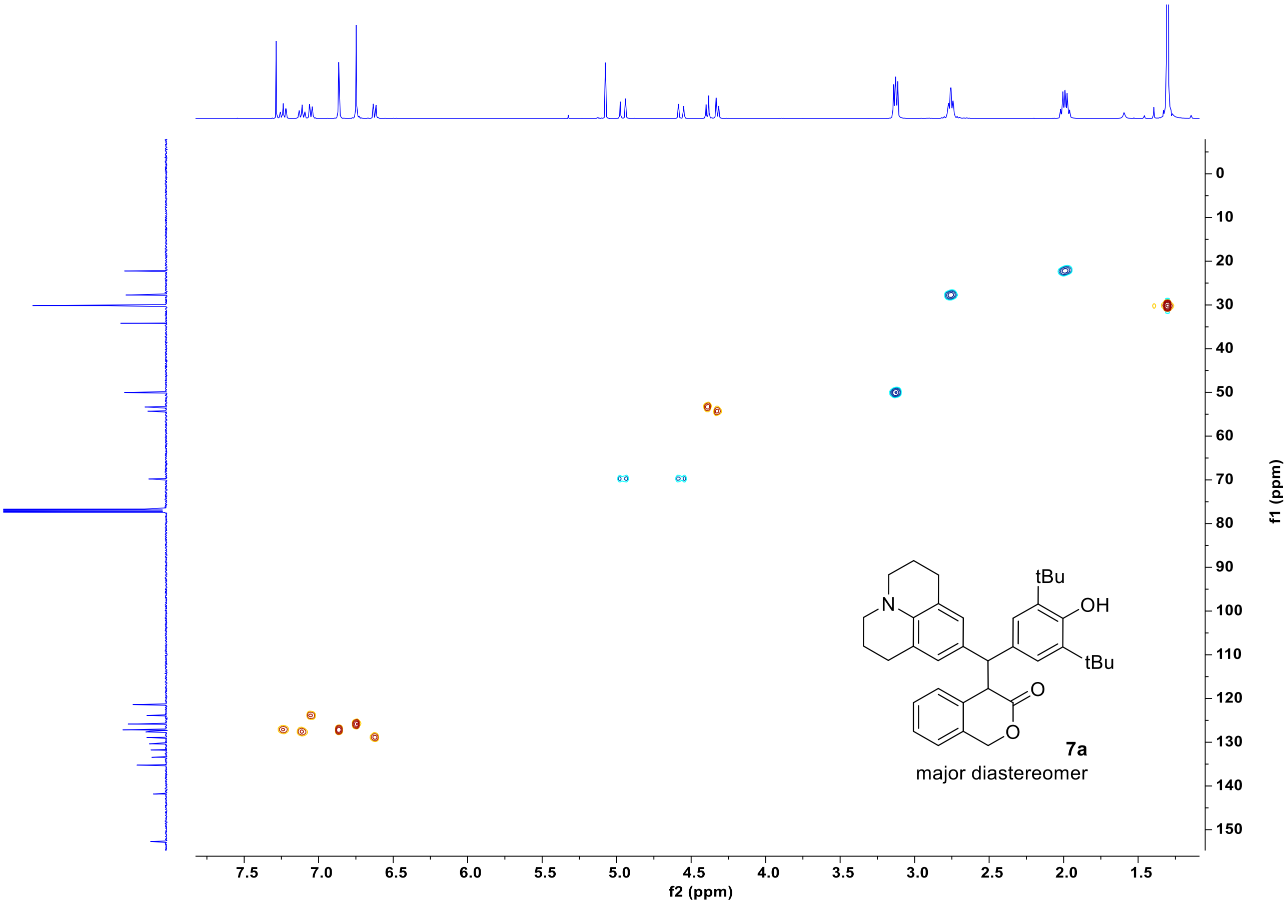

HMBC spectrum of **7a**-major (in $CDCl_3$):

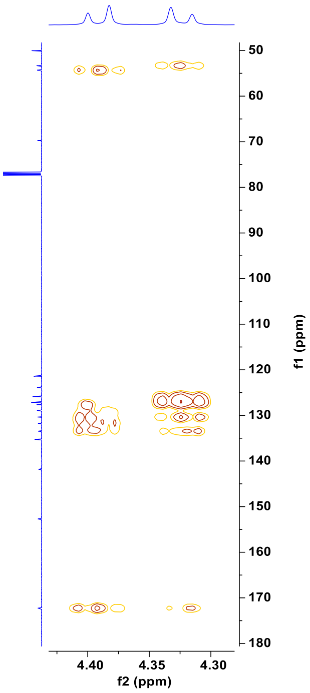

^{1}**H NMR** (400 MHz, $CDCl_3$) of **7a** (mixture of diastereomers):

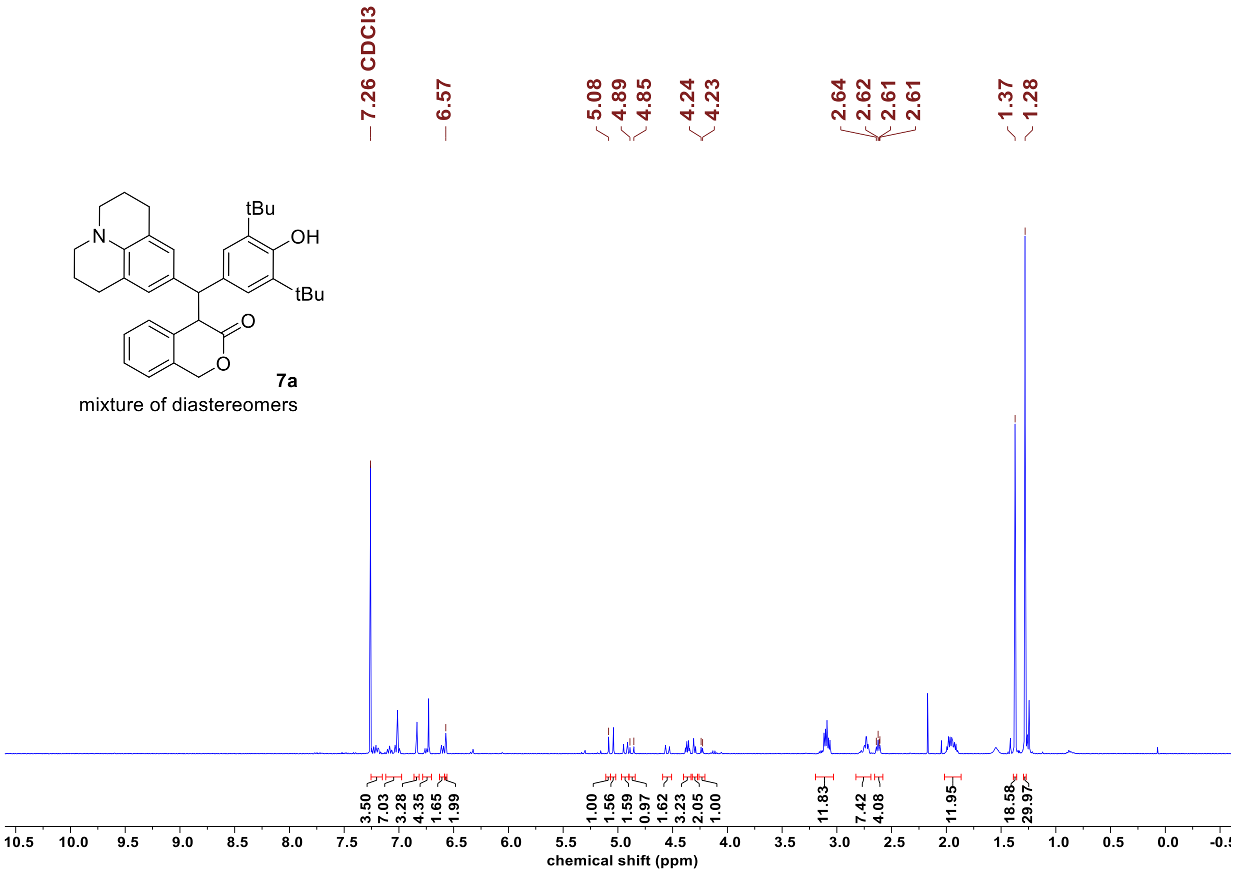

^{13}**C{**^{1}**H} NMR** (101 MHz, $CDCl_3$) of **7a** (mixture of diastereomers):

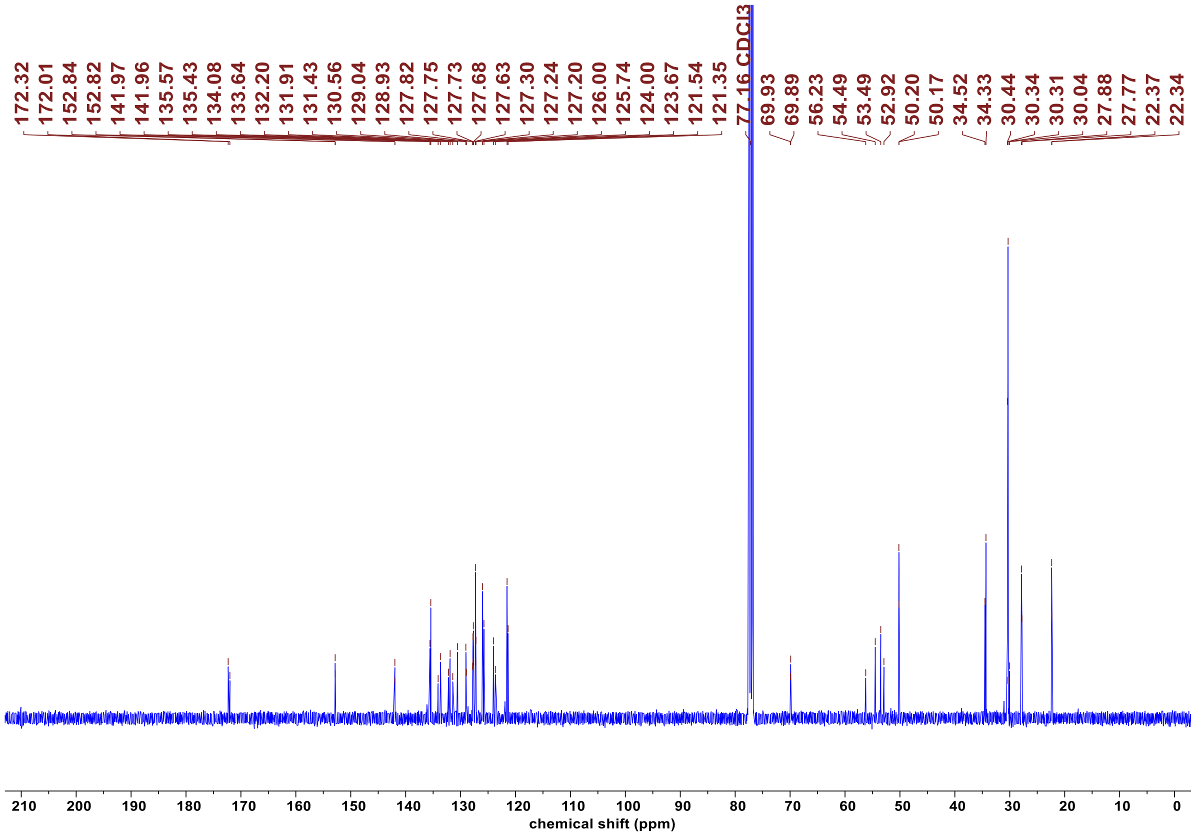

^{13}C NMR signals assigned to the minor diastereomer of **7a**:

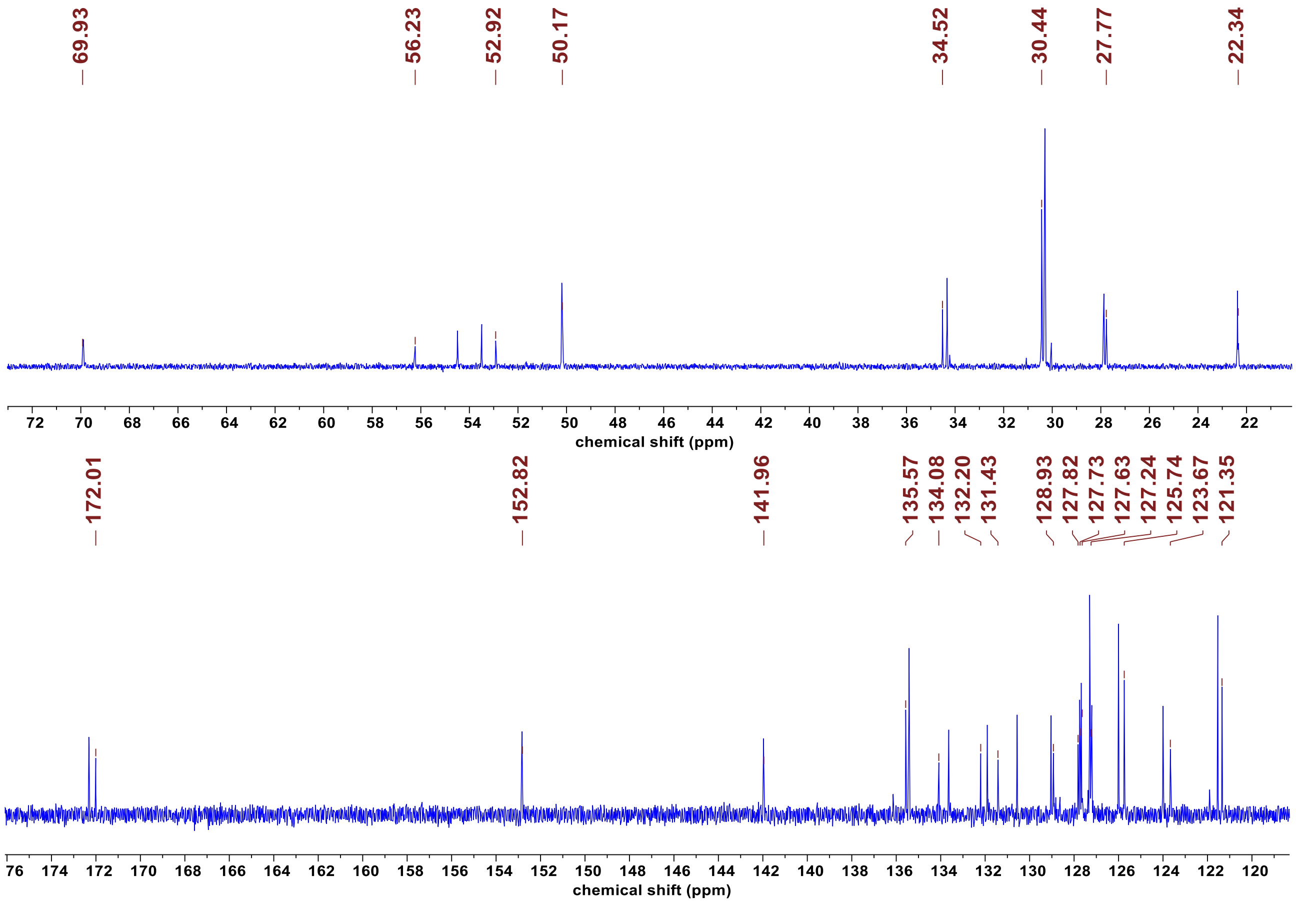

4-((3,5-Di-tert-butyl-4-hydroxyphenyl)(4-(dimethylamino)phenyl)-λ^3-methyl)-4λ^3-isochroman-3-one (7b)

^{1}**H NMR** (400 MHz, CDCl$_3$) of **7b** (mixture of diastereomers):

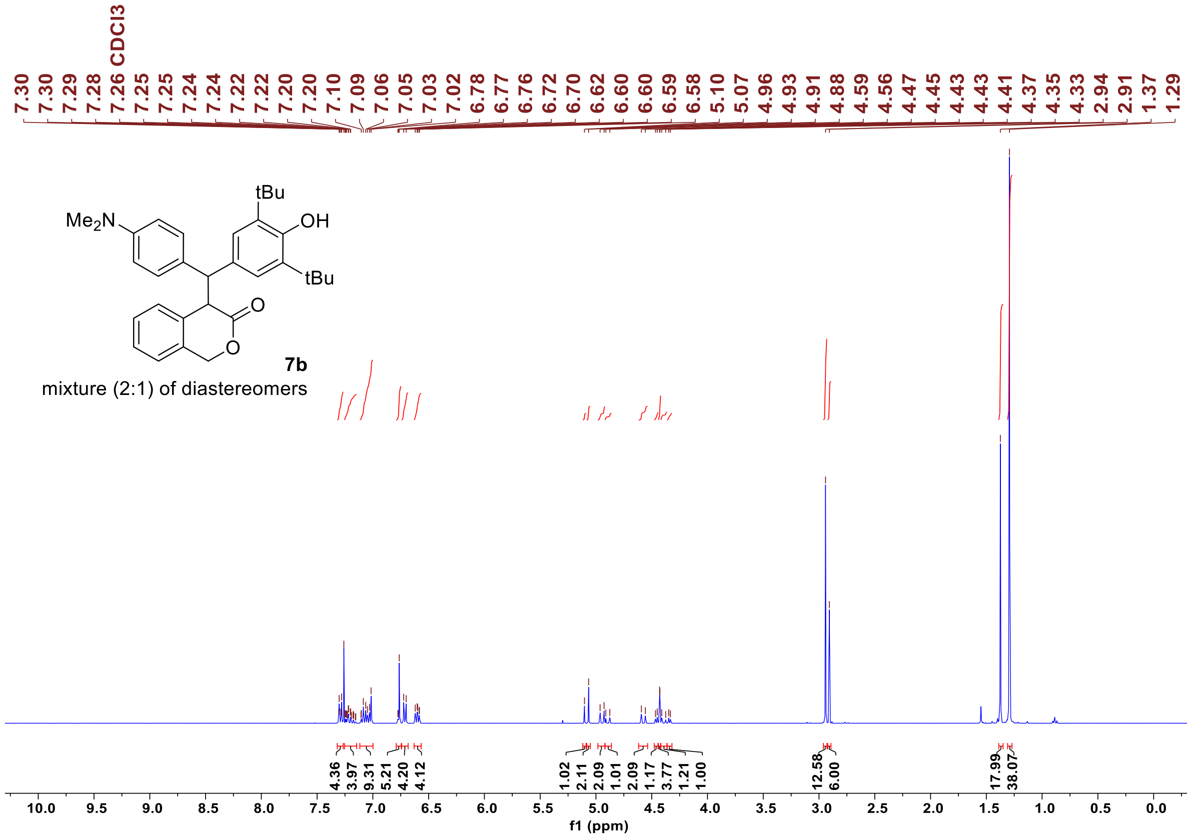

^{13}**C{^{1}H} NMR** (101 MHz, CDCl$_3$) of **7b** (mixture of diastereomers):

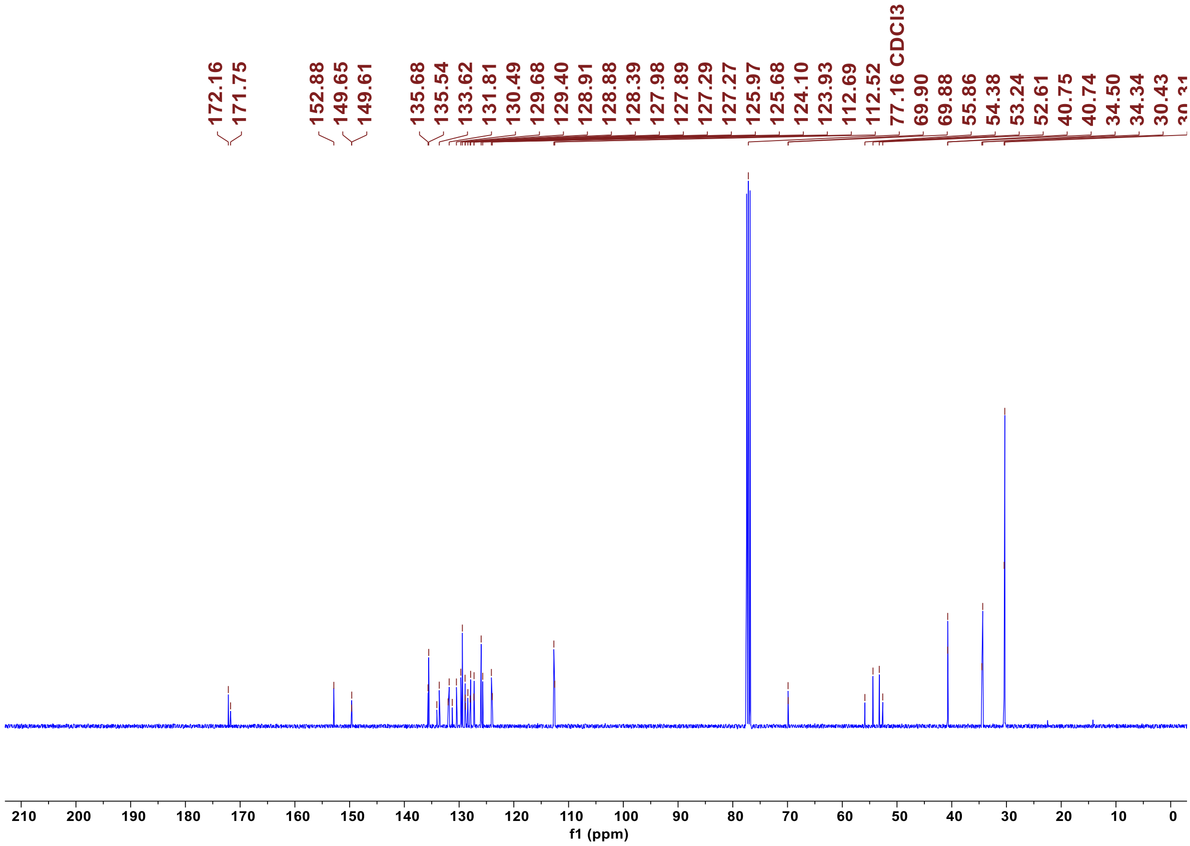

HSQC spectrum of **7b** (mixture of diastereomers, in $CDCl_3$):

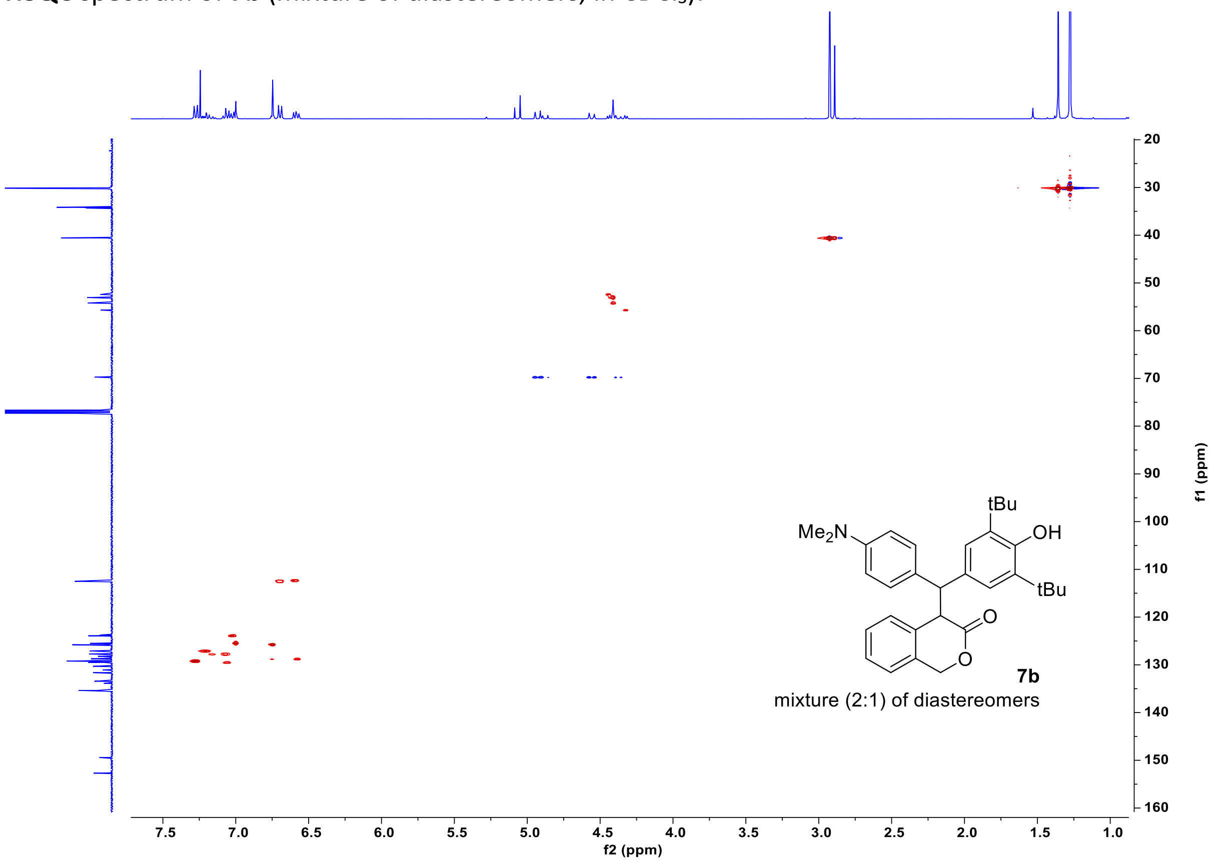

^{13}C NMR signals assigned to the major diastereomer of **7b**:

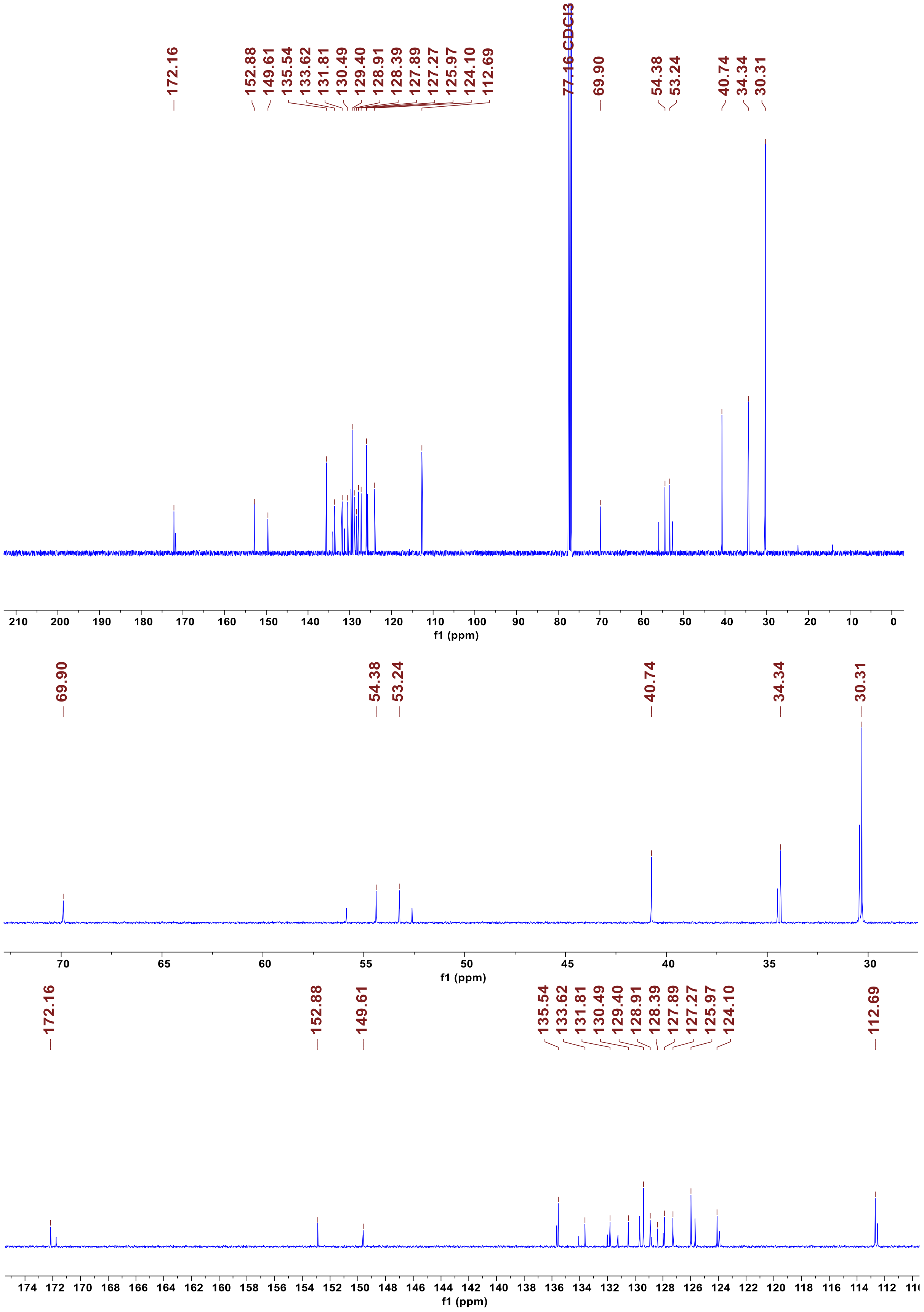

^{13}C NMR signals of the minor diastereomer of **7b**:

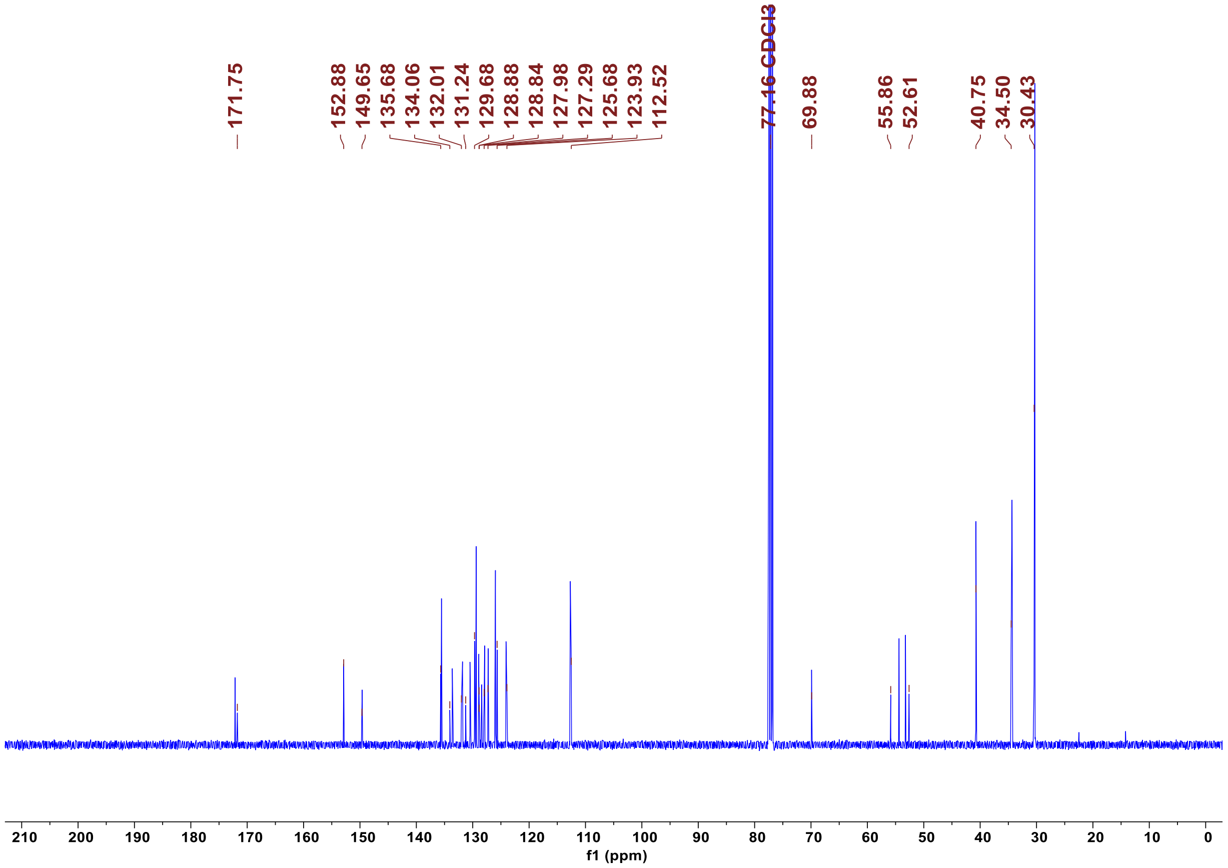

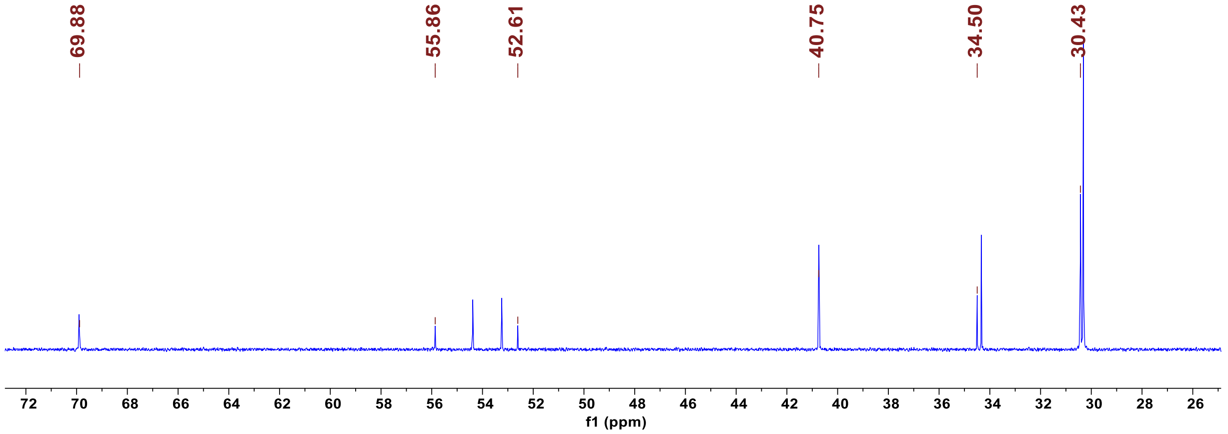

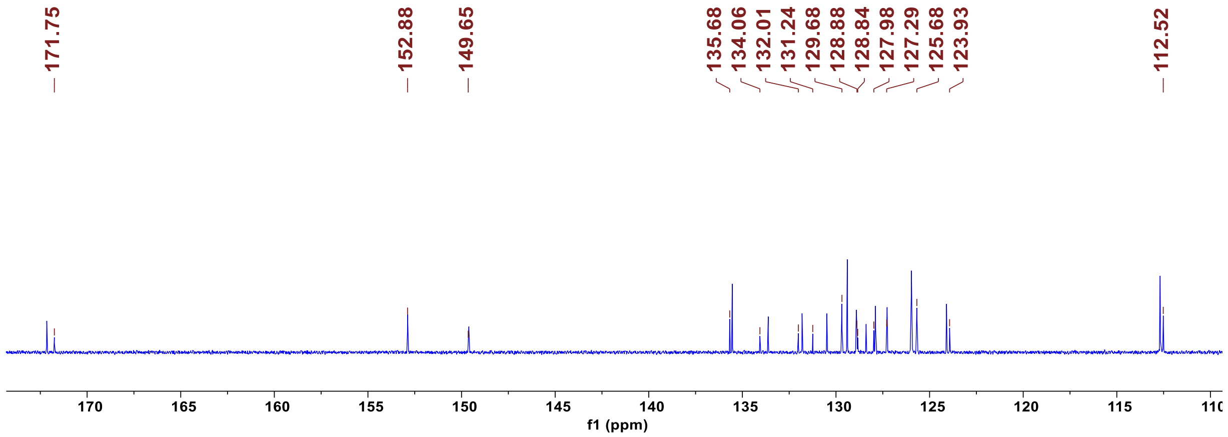

4-((4-Hydroxy-3,5-dimethoxyphenyl)(4-methoxyphenyl)-λ³-methyl)- 4λ³-isochroman-3-one (7c)

^{1}H NMR (599 MHz, $CDCl_3$) of **7c** (mixture of diastereomers):

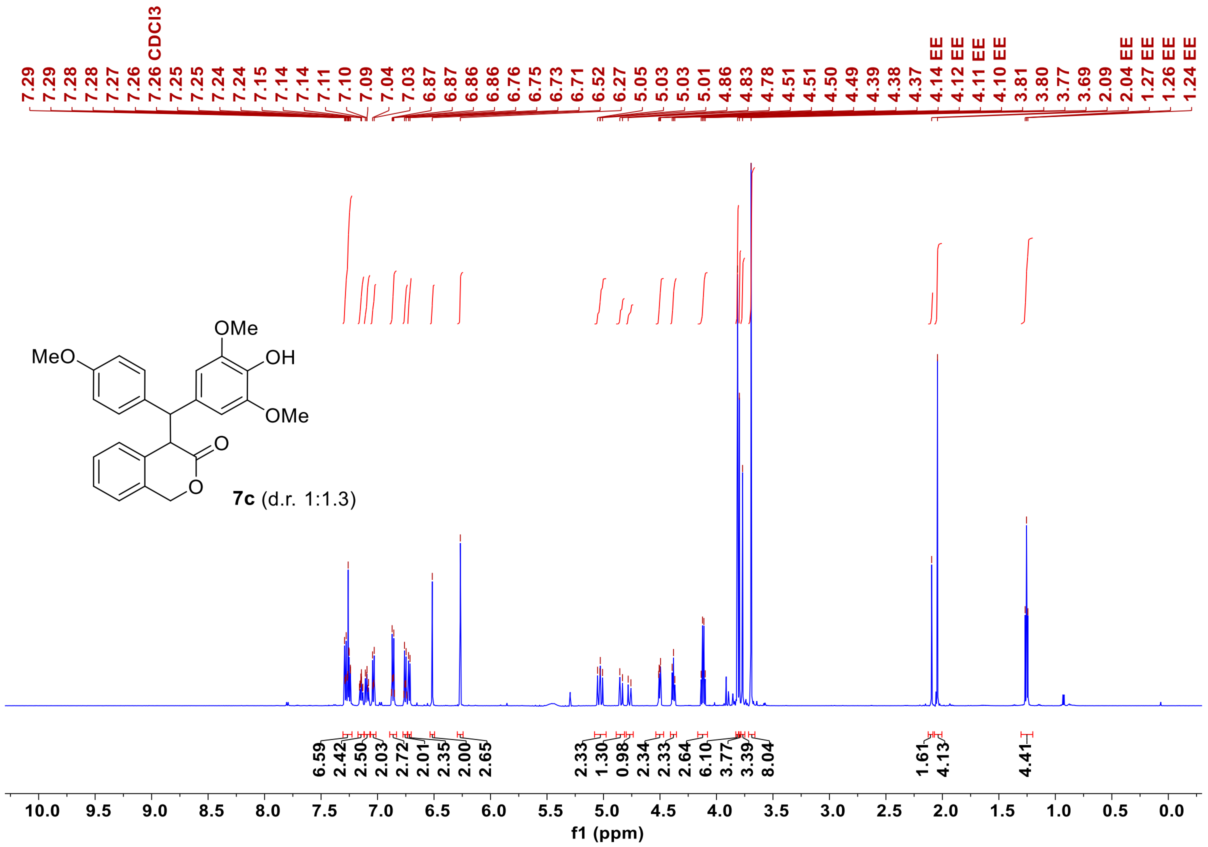

^{13}C{^{1}H} NMR (151 MHz, $CDCl_3$) of **7c** (mixture of diastereomers):

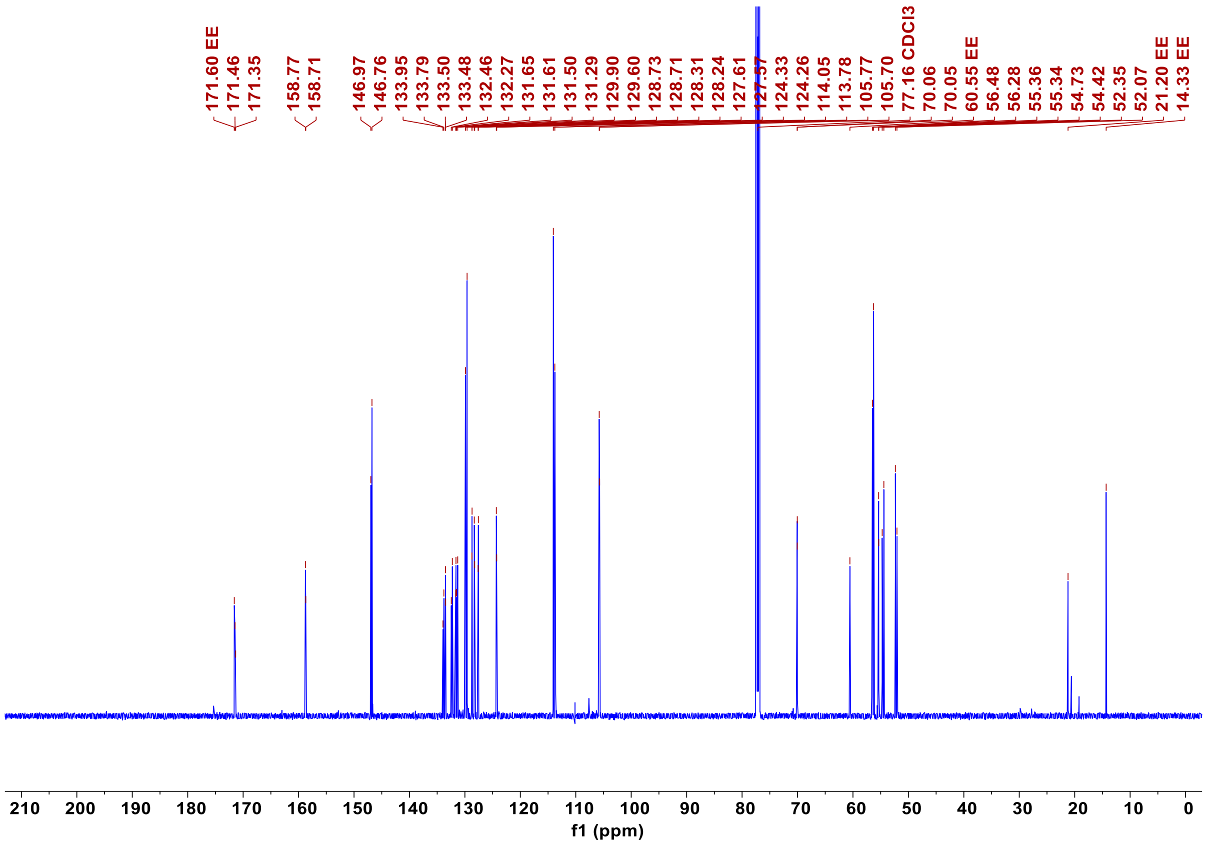

HSQC spectrum of **7c** (mixture of diastereomers, in $CDCl_3$):

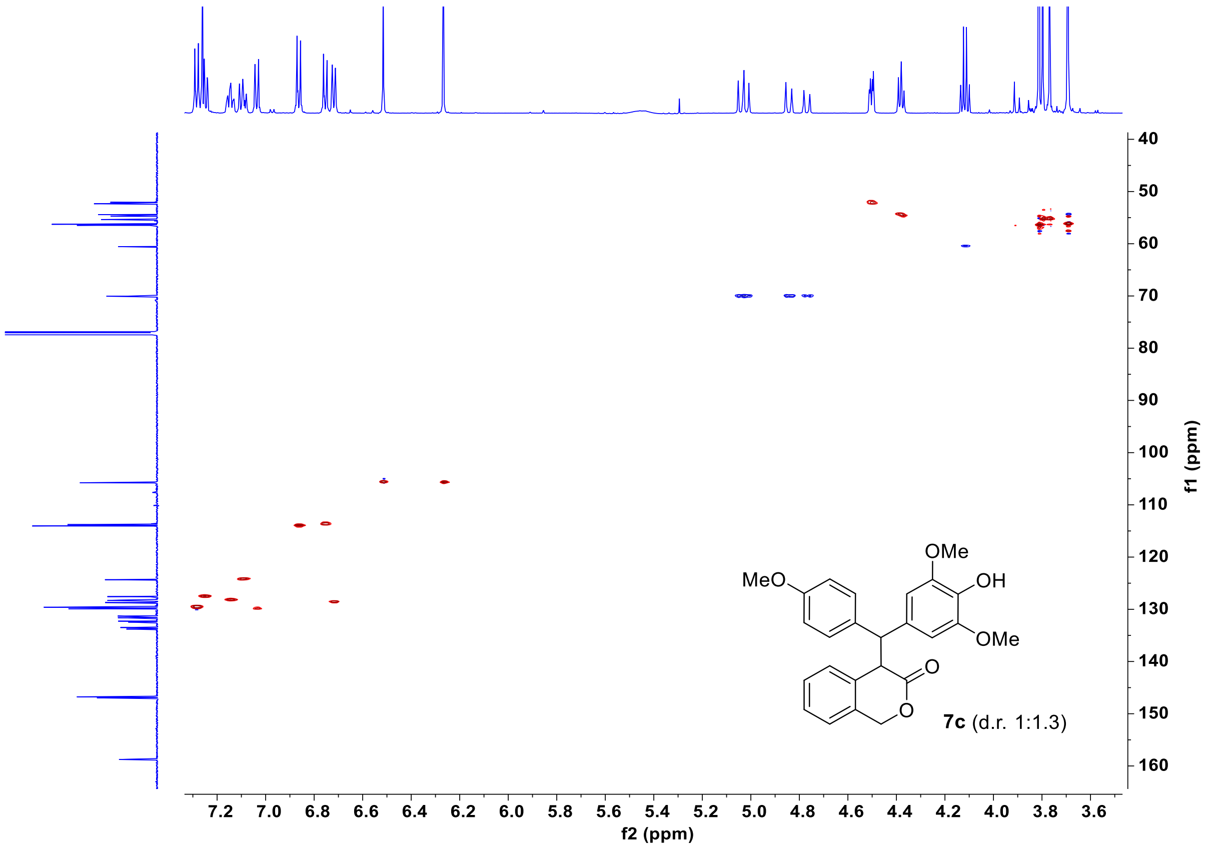

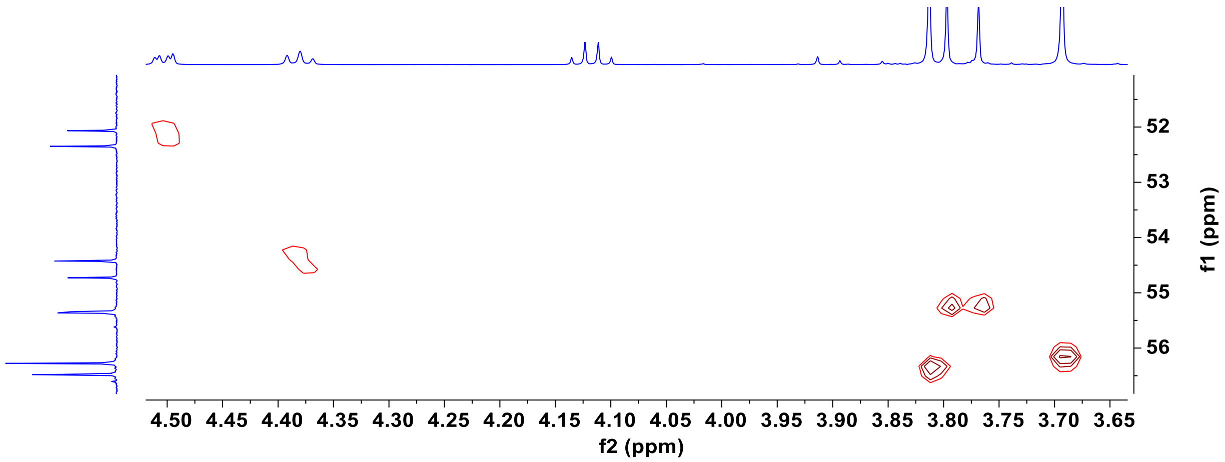

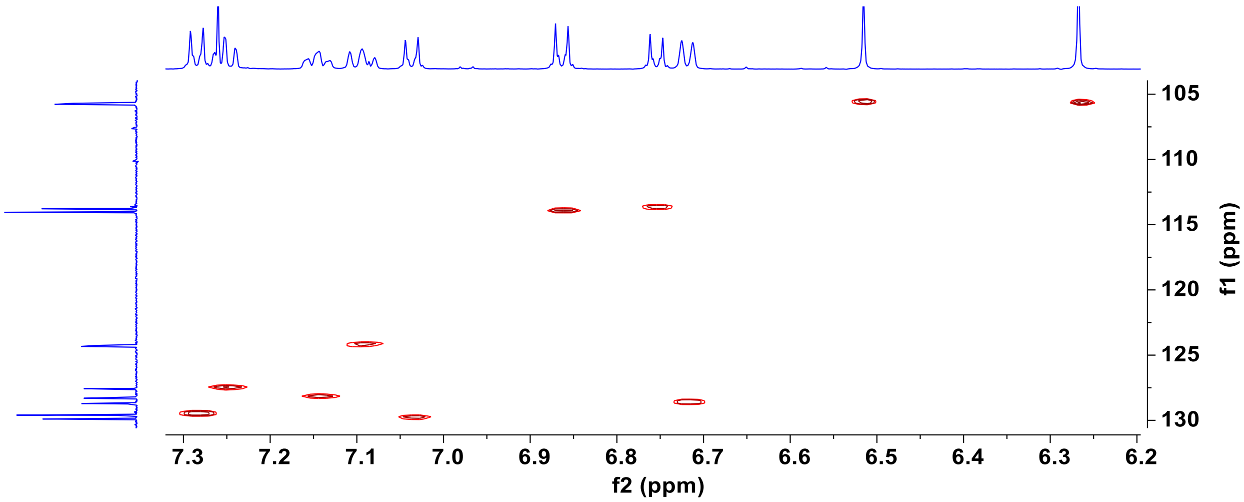

^{13}C NMR signals assigned to major diastereomer of **7c**:

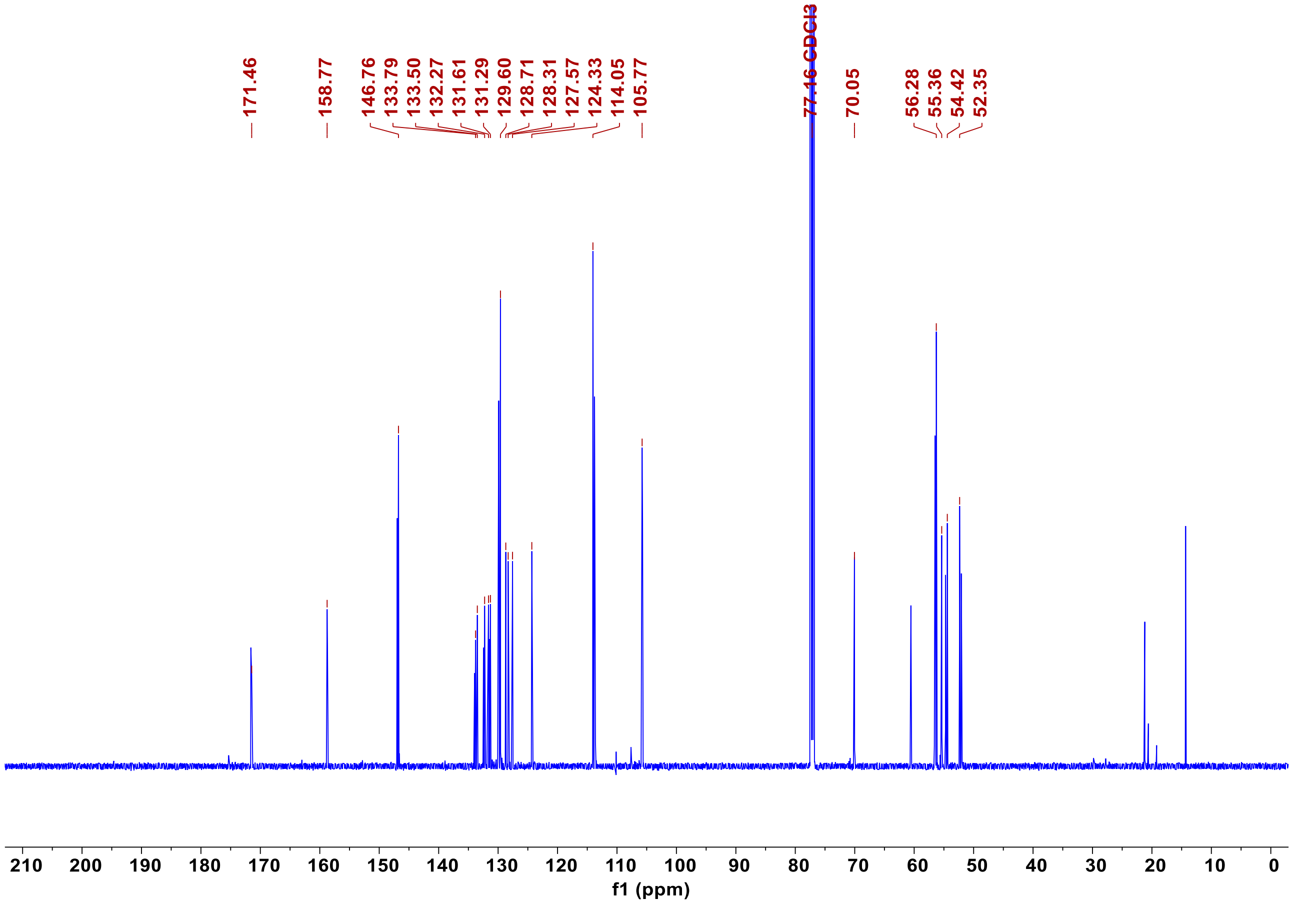

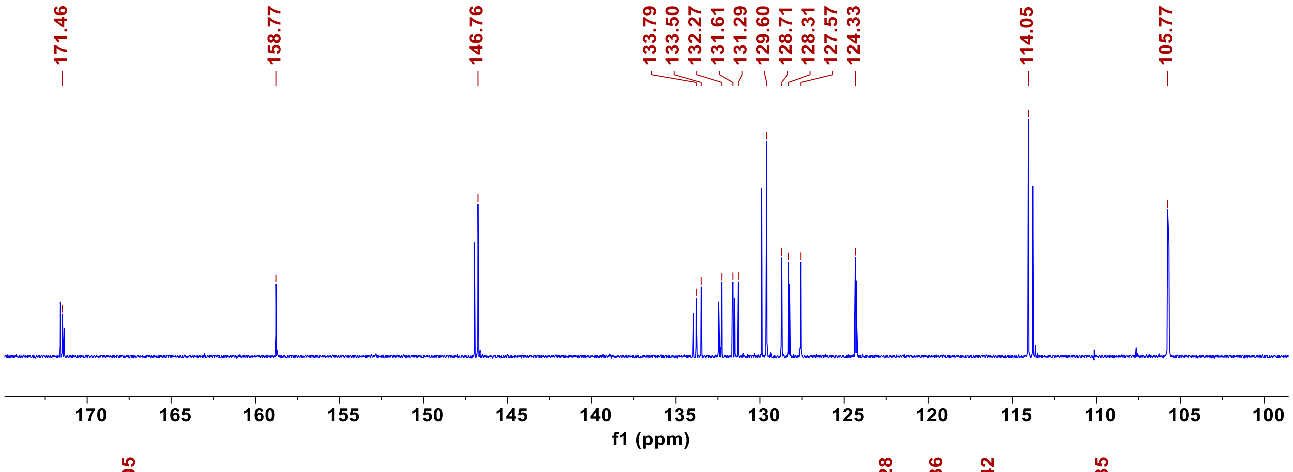

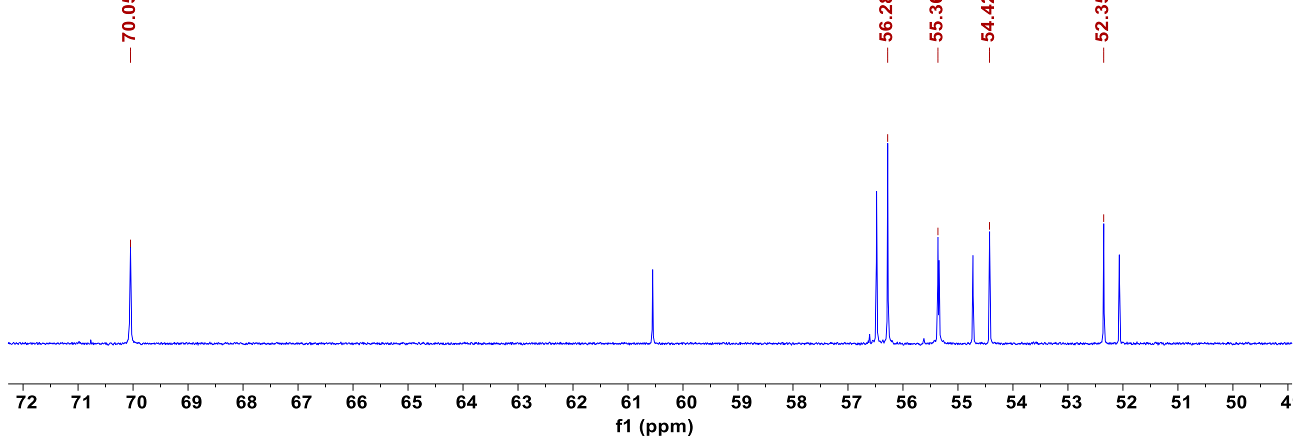

^{13}C NMR signals of the minor diastereomer of **7c**:

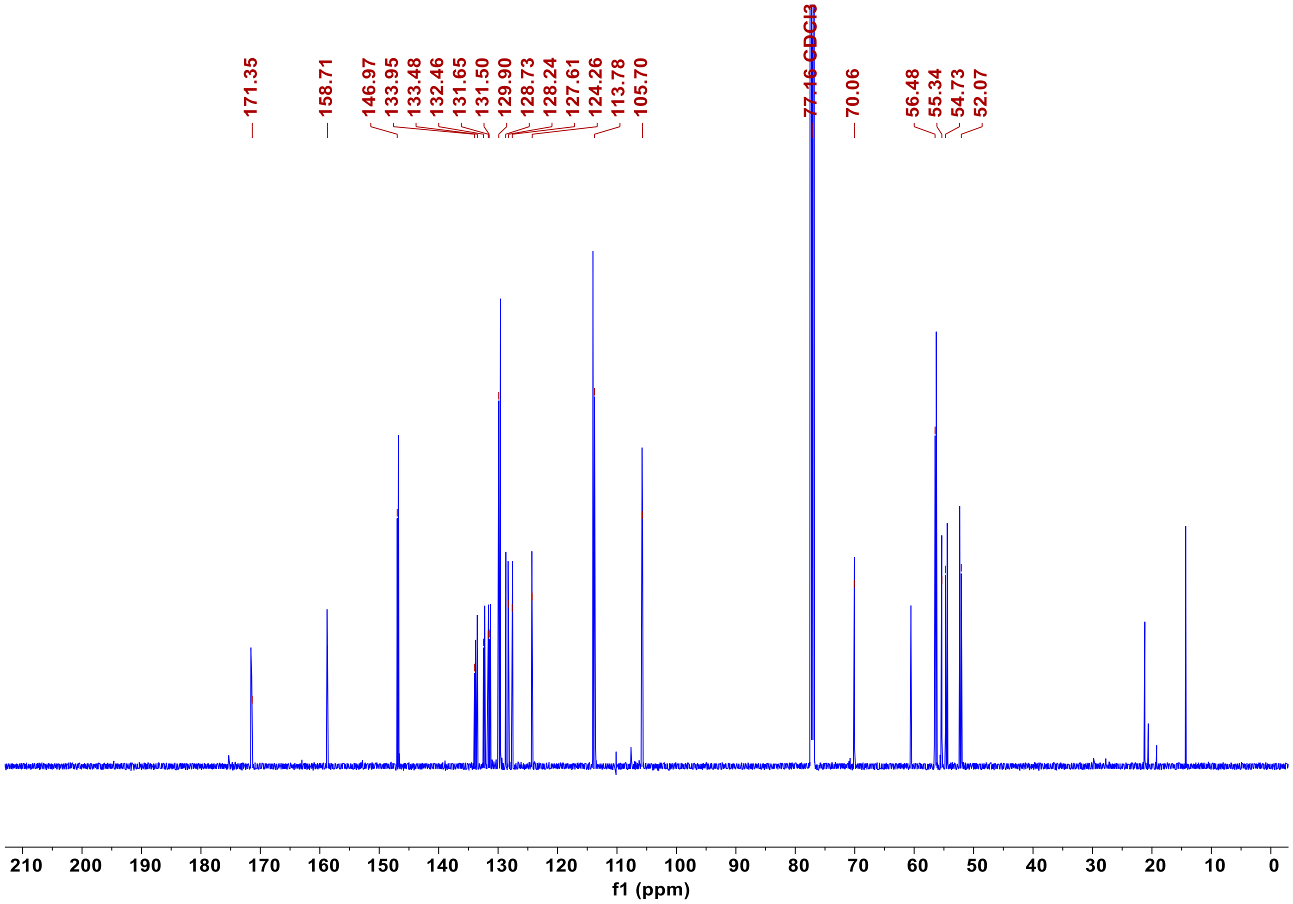

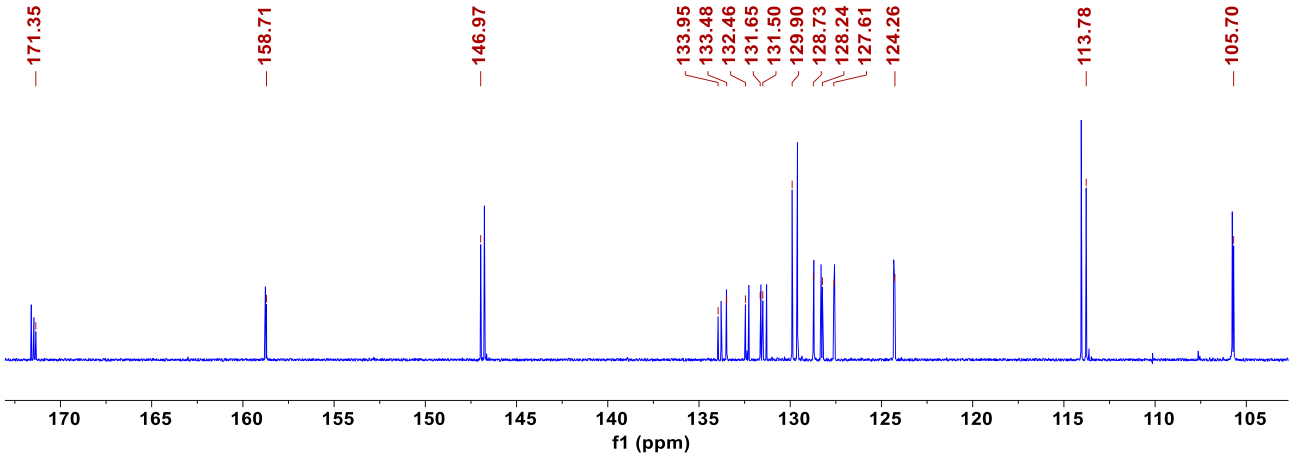

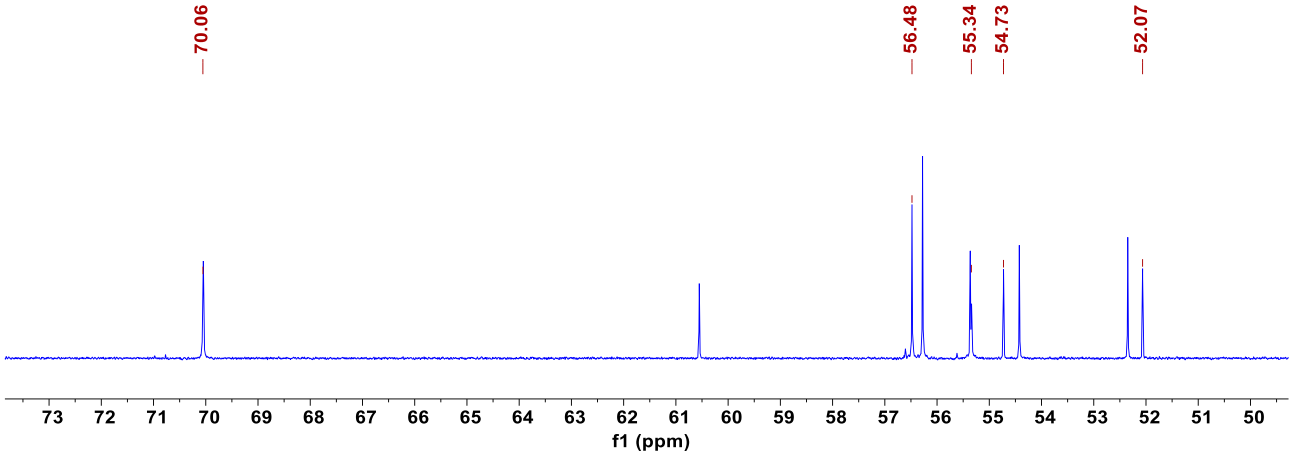

3-((3,5-Di-*tert*-butyl-4-hydroxyphenyl)(4-methoxyphenyl)methyl)benzofuran-2(3H)-one (**8**)

[1]**H NMR** (400 MHz, $CDCl_3$) of **8** (mixture of diastereomers):

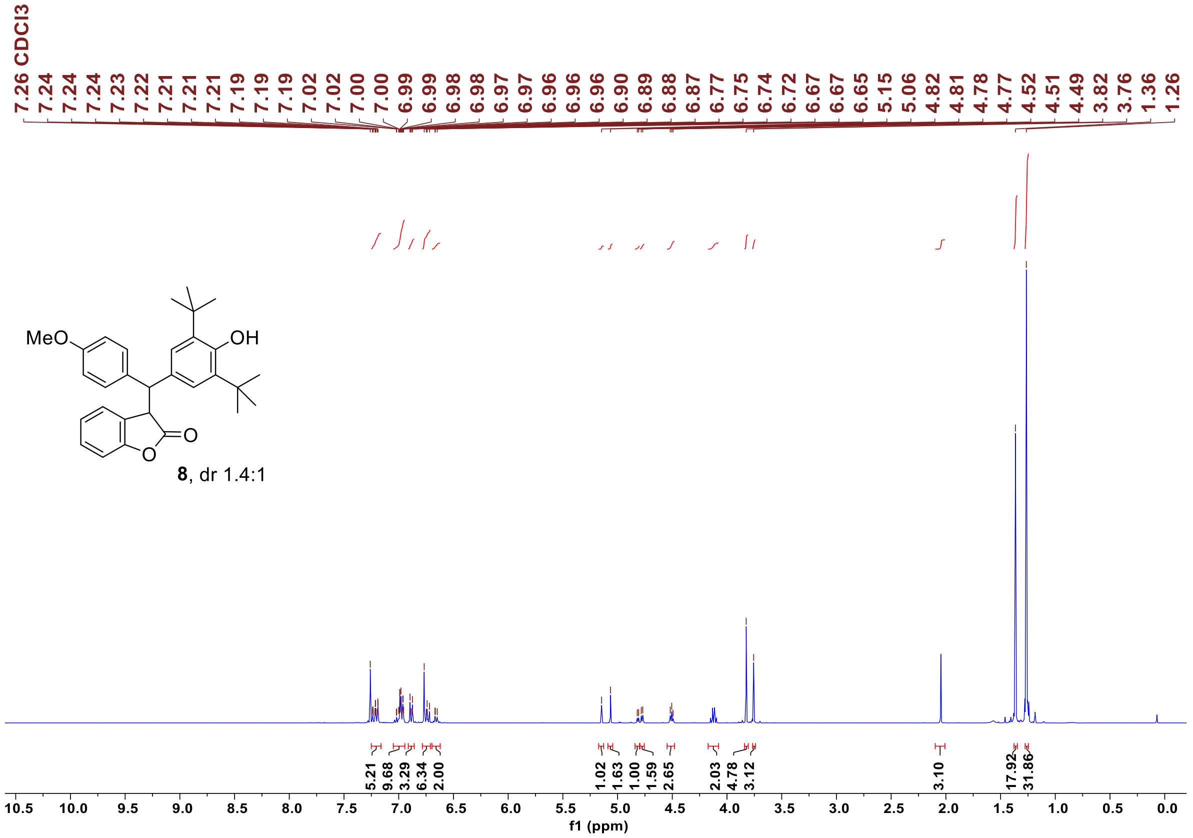

HMBC spectrum of **8** (mixture of diastereomers, in $CDCl_3$):

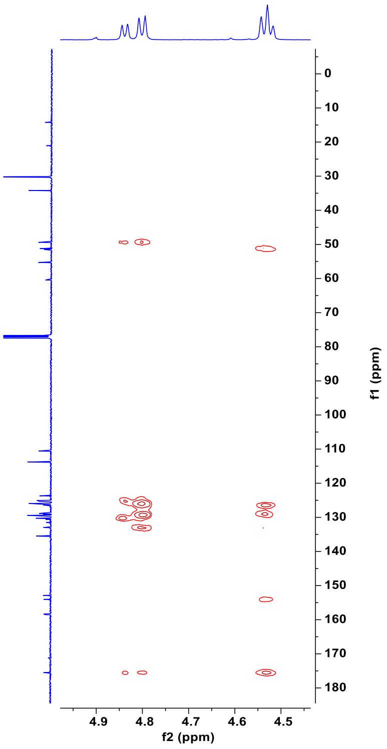

^{13}C{^{1}H} **NMR** (101 MHz, $CDCl_3$) of **8** (mixture of diastereomers):

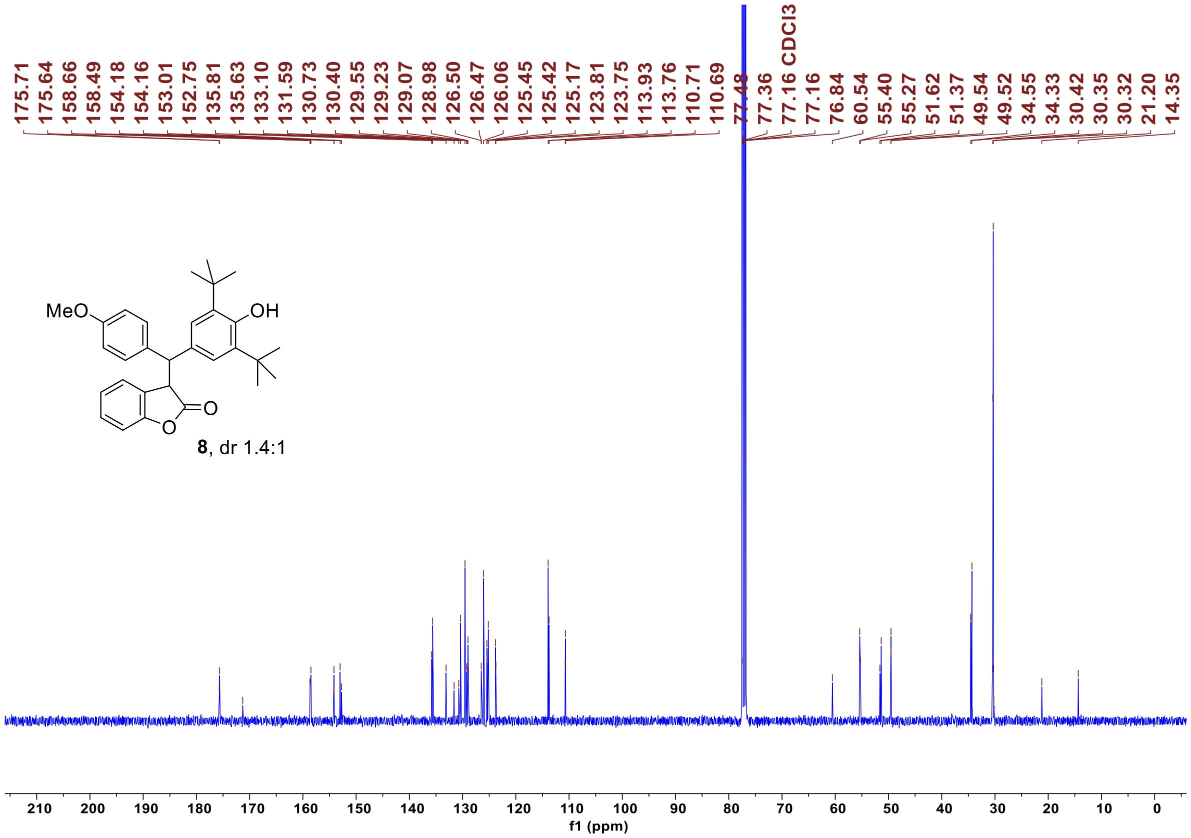

HSQC spectrum of **8** (mixture of diastereomers, in $CDCl_3$):

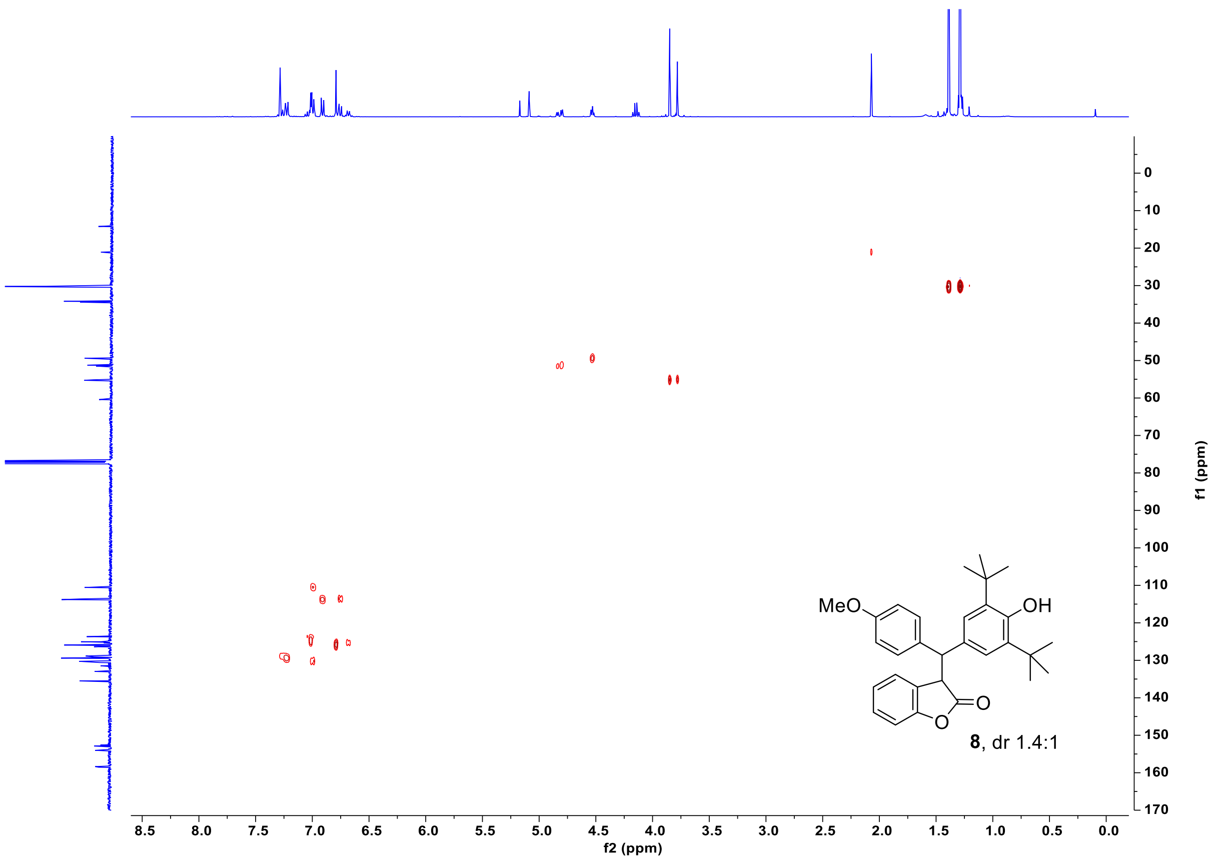

^{13}C NMR signals assigned to the major diastereomer of **8**:

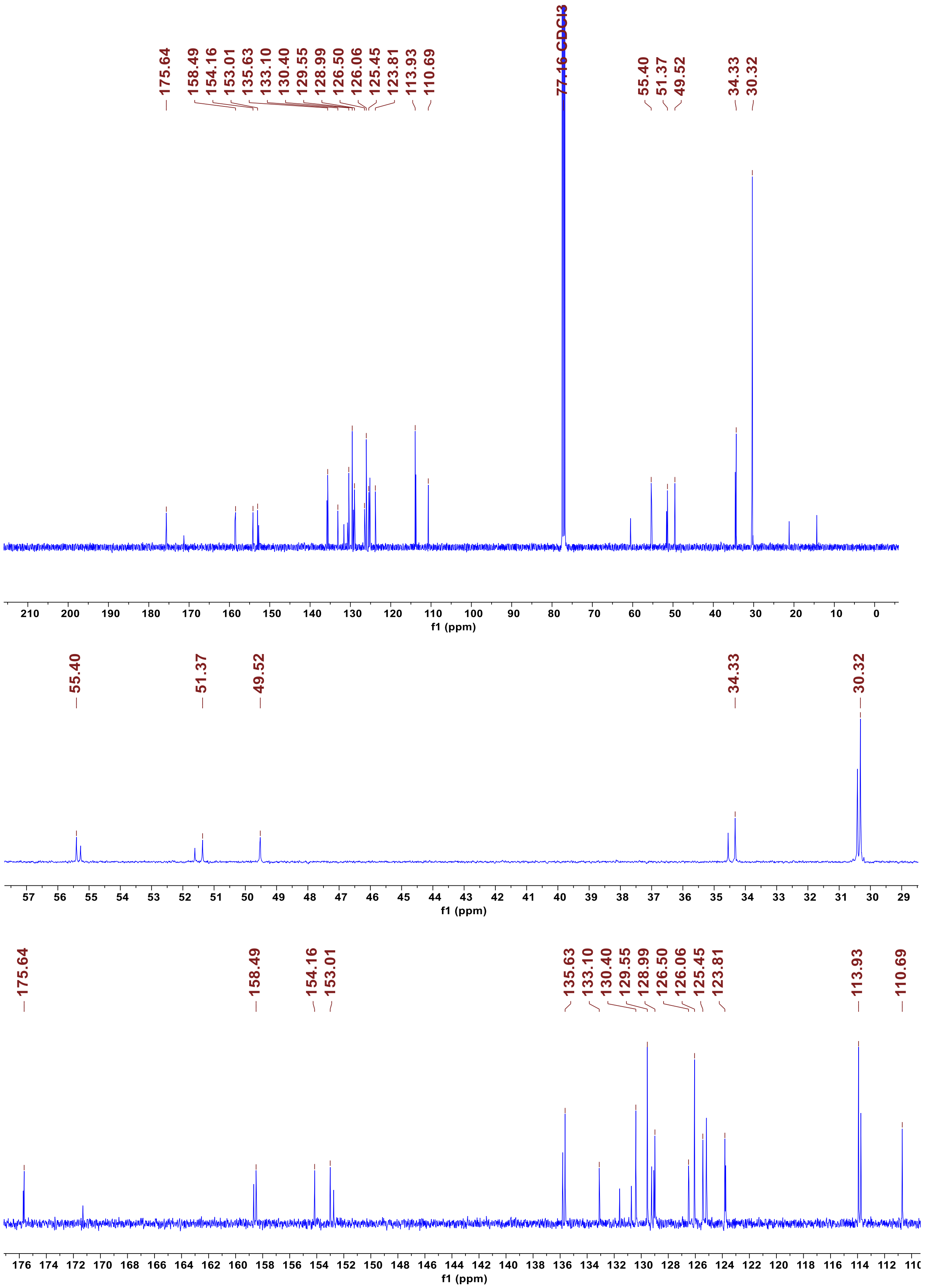

^{13}C NMR signals assigned to the minor diastereomer of **8**:

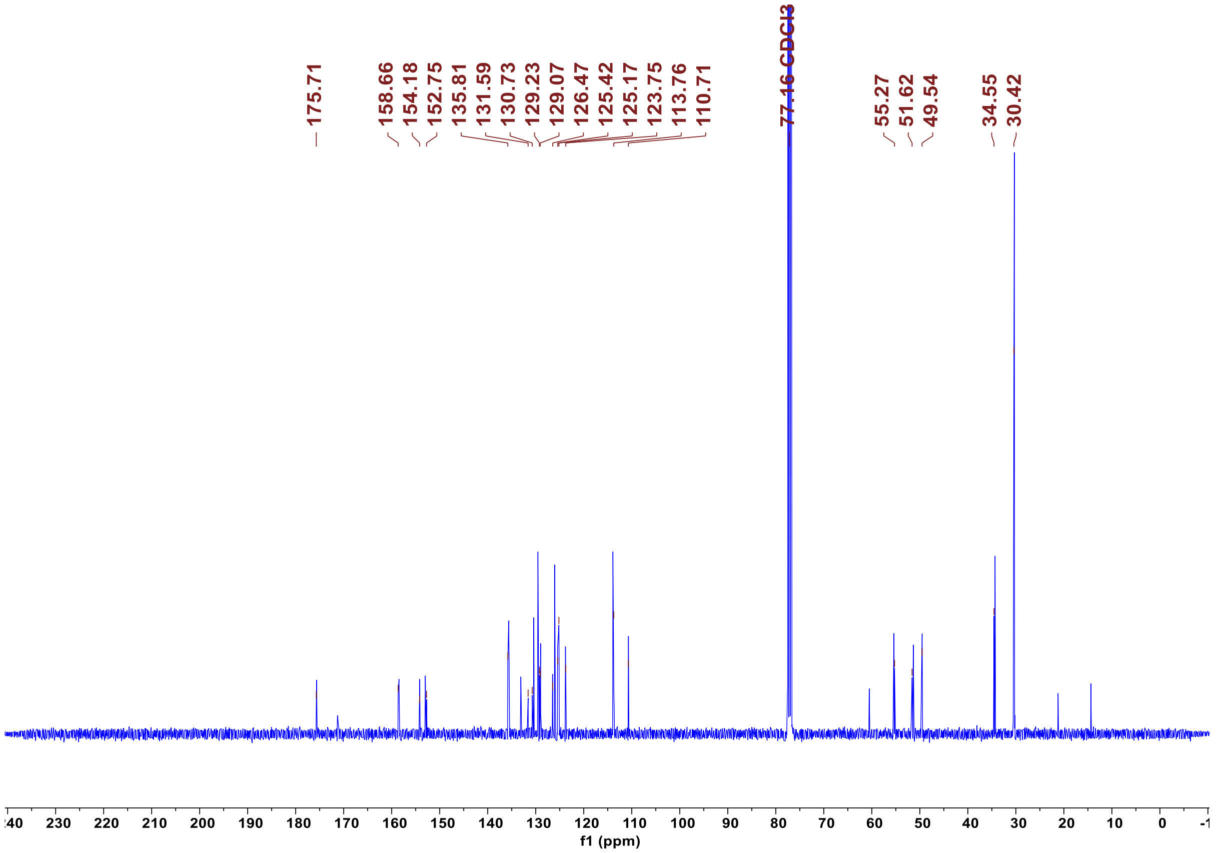

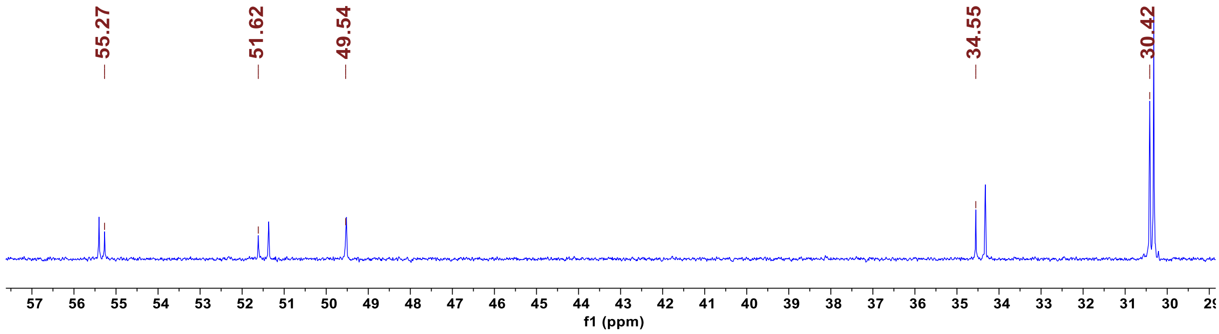

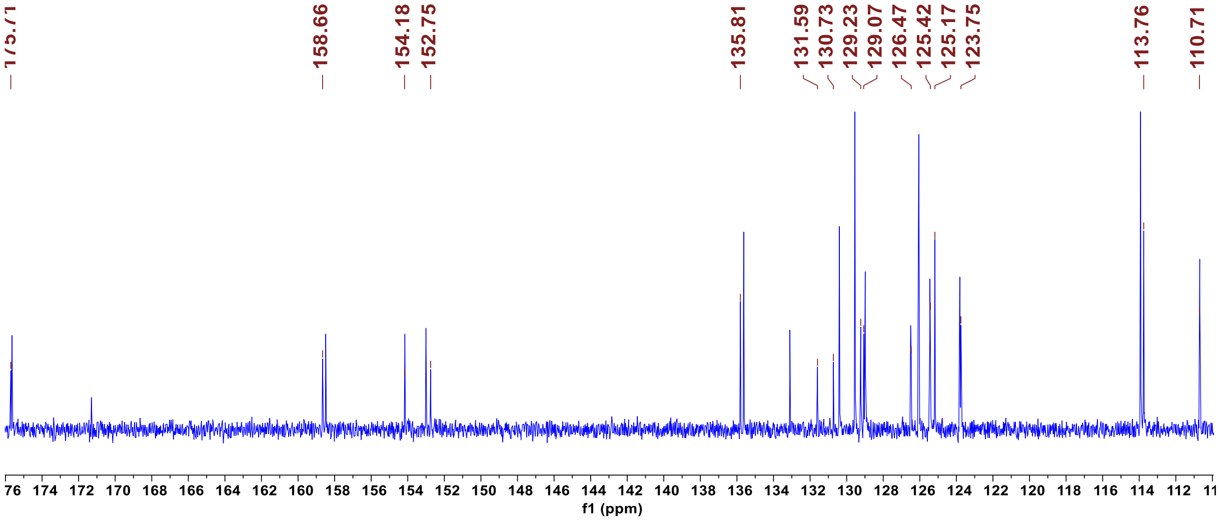

Dimethyl 3,3'-(2-oxo-2,3-dihydrobenzofuran-3,3-diyl)dipropionate (10)

^{1}H NMR (599 MHz, $CDCl_3$) of **10**:

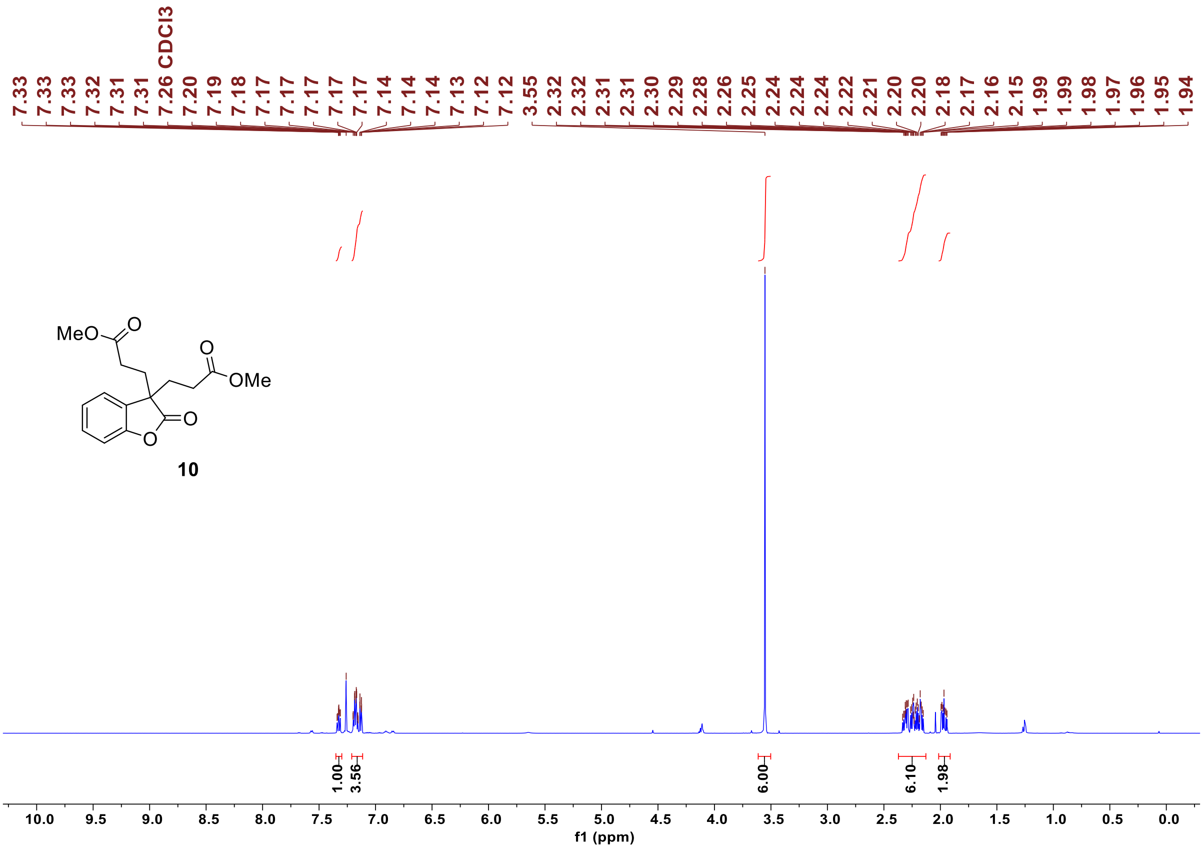

$^{13}C\{^1H\}$ NMR (151 MHz, $CDCl_3$) of **10**:

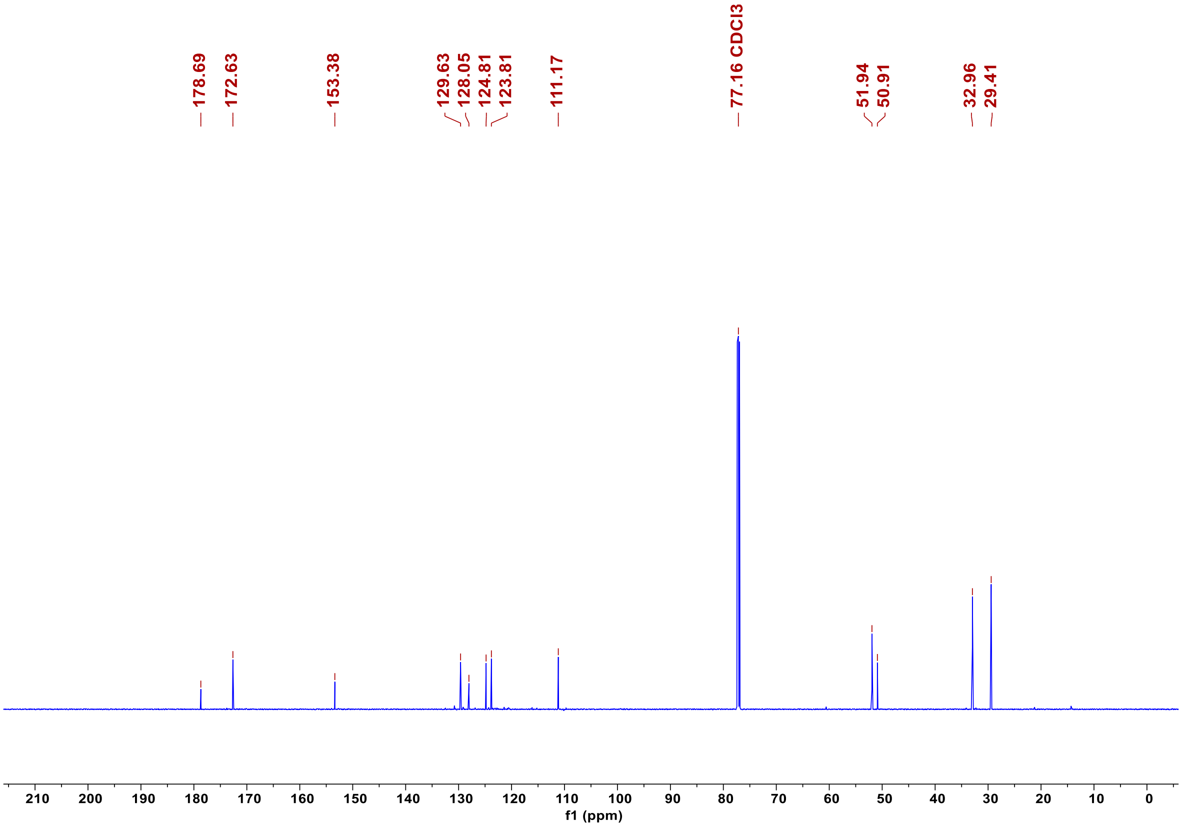

HSQC spectrum of **10** (in $CDCl_3$):

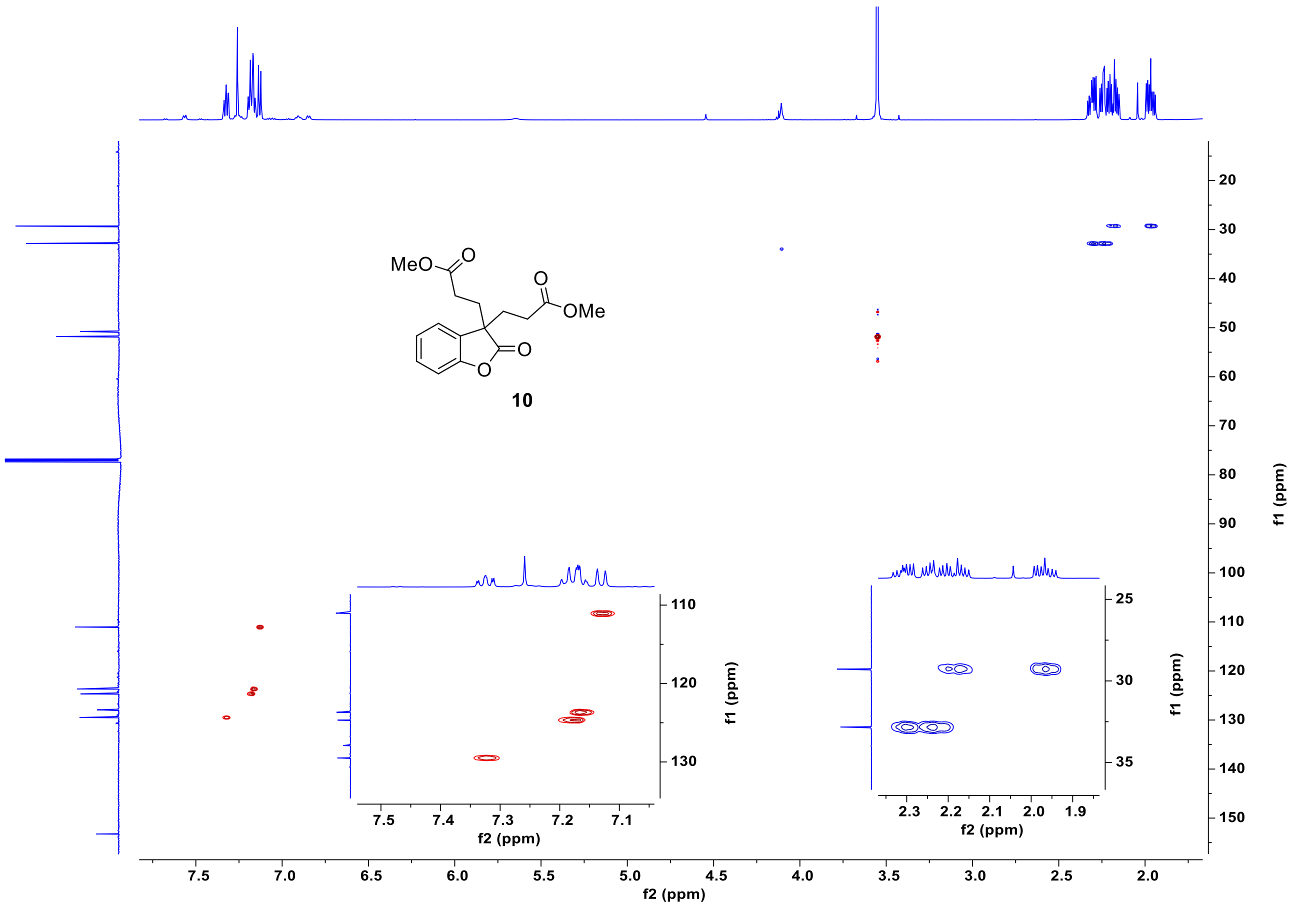

4,4'-(2-Oxo-2,3-dihydrobenzofuran-3,3-diyl)bis(butan-2-one) (12)

^{1}H NMR (599 MHz, $CDCl_3$) of **12**:

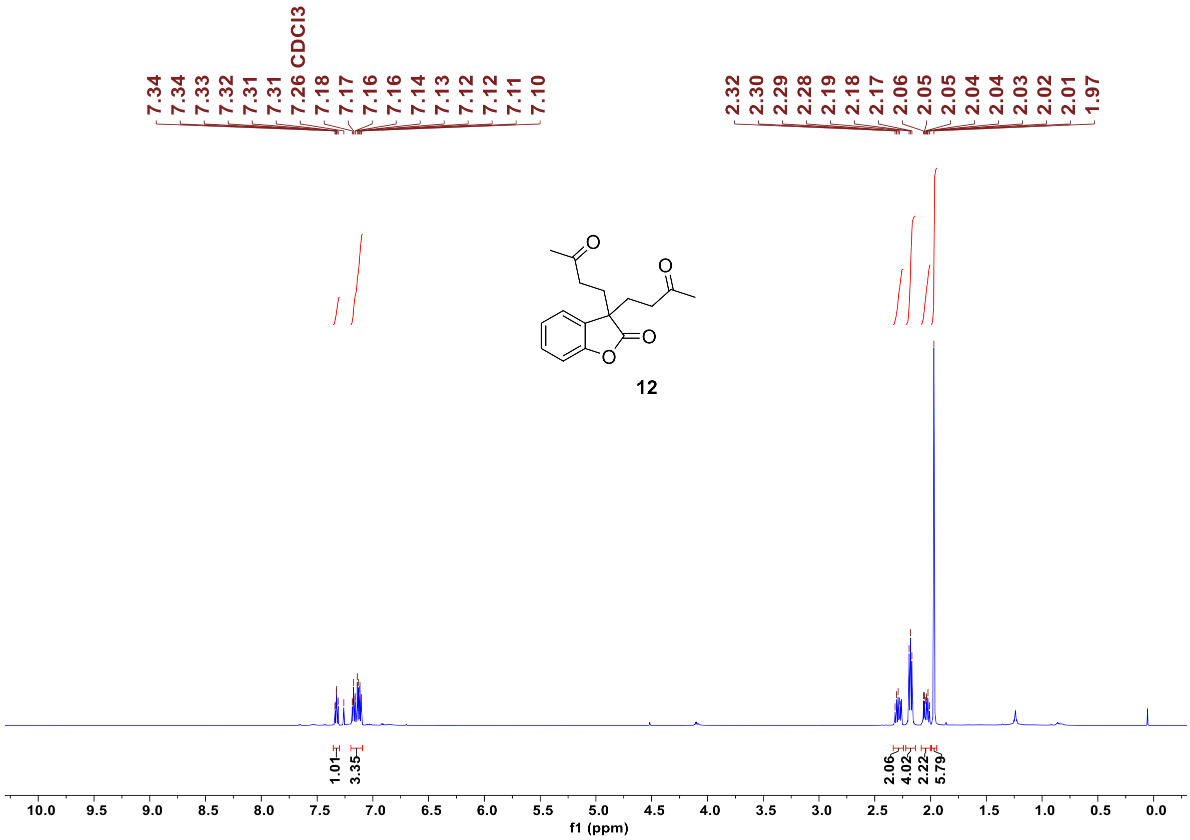

^{13}C{^{1}H} NMR (151 MHz, $CDCl_3$) of **12**:

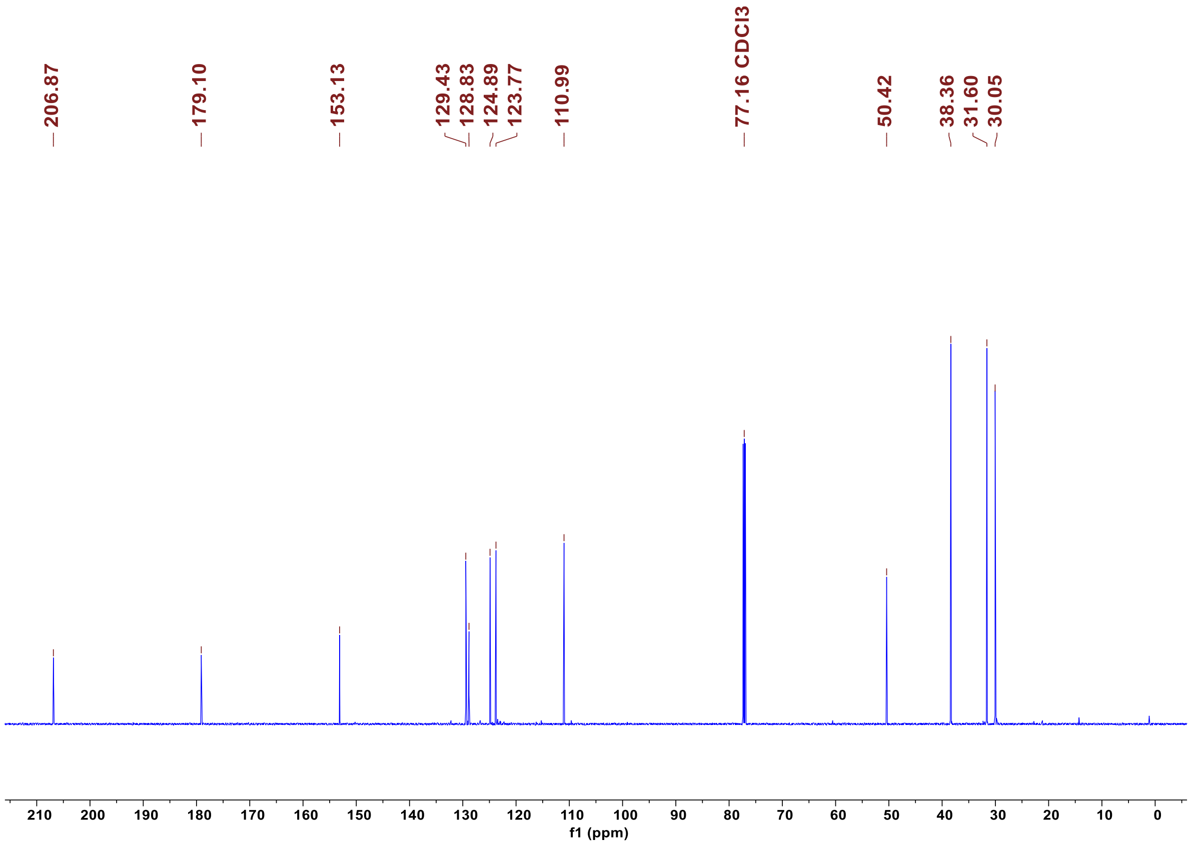

HSQC spectrum of **12** (in $CDCl_3$):

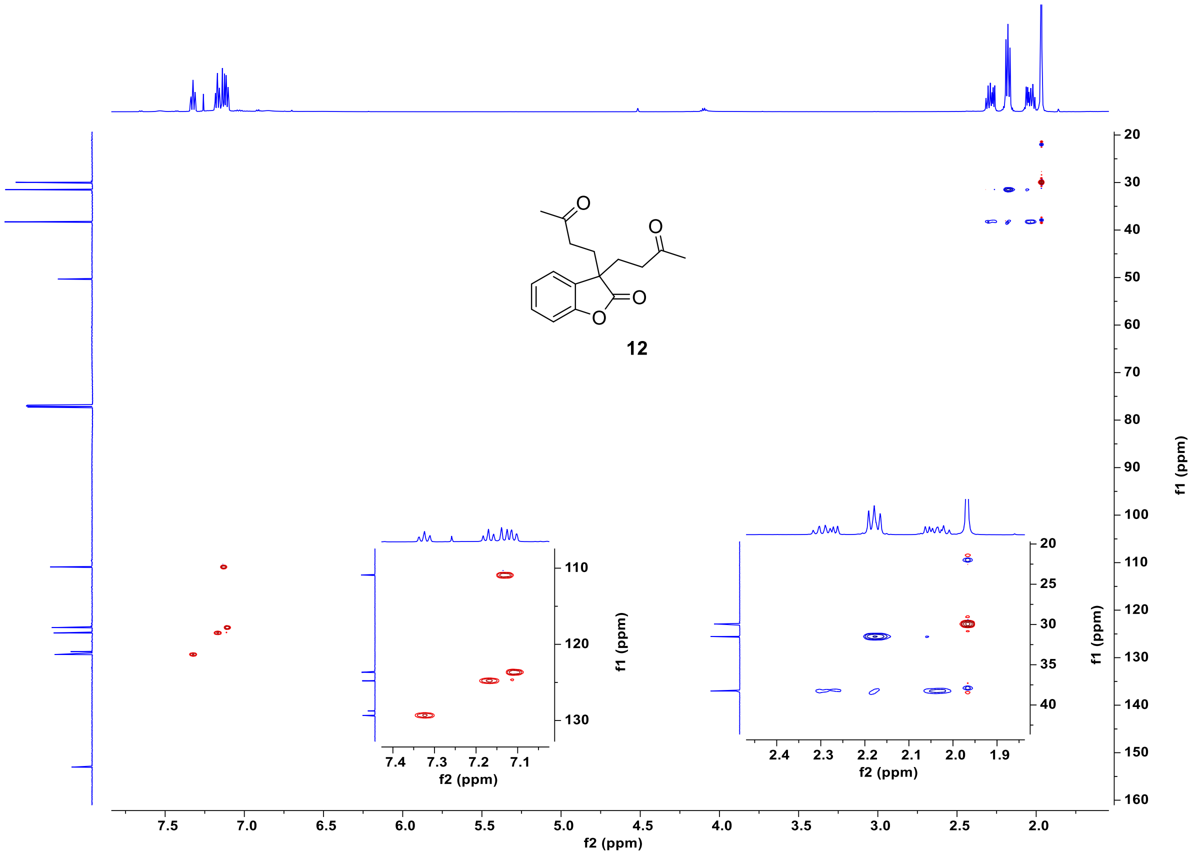

(R*)-4-((R*)-3-oxo-1,3-diphenylpropyl)isochroman-3-one (14a-1)

^{1}H NMR (400 MHz, $CDCl_3$)

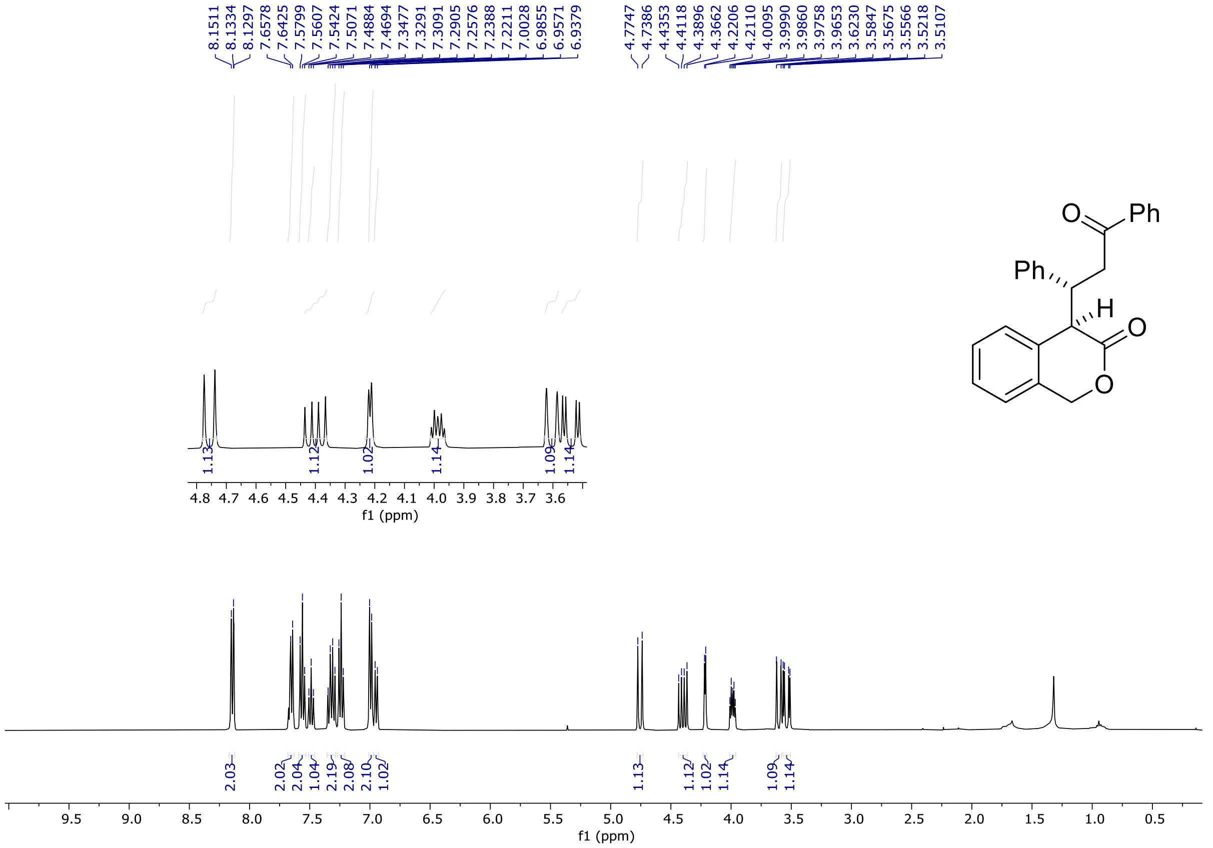

$^{13}C\{^{1}H\}$ NMR (150 MHz, $CDCl_3$)

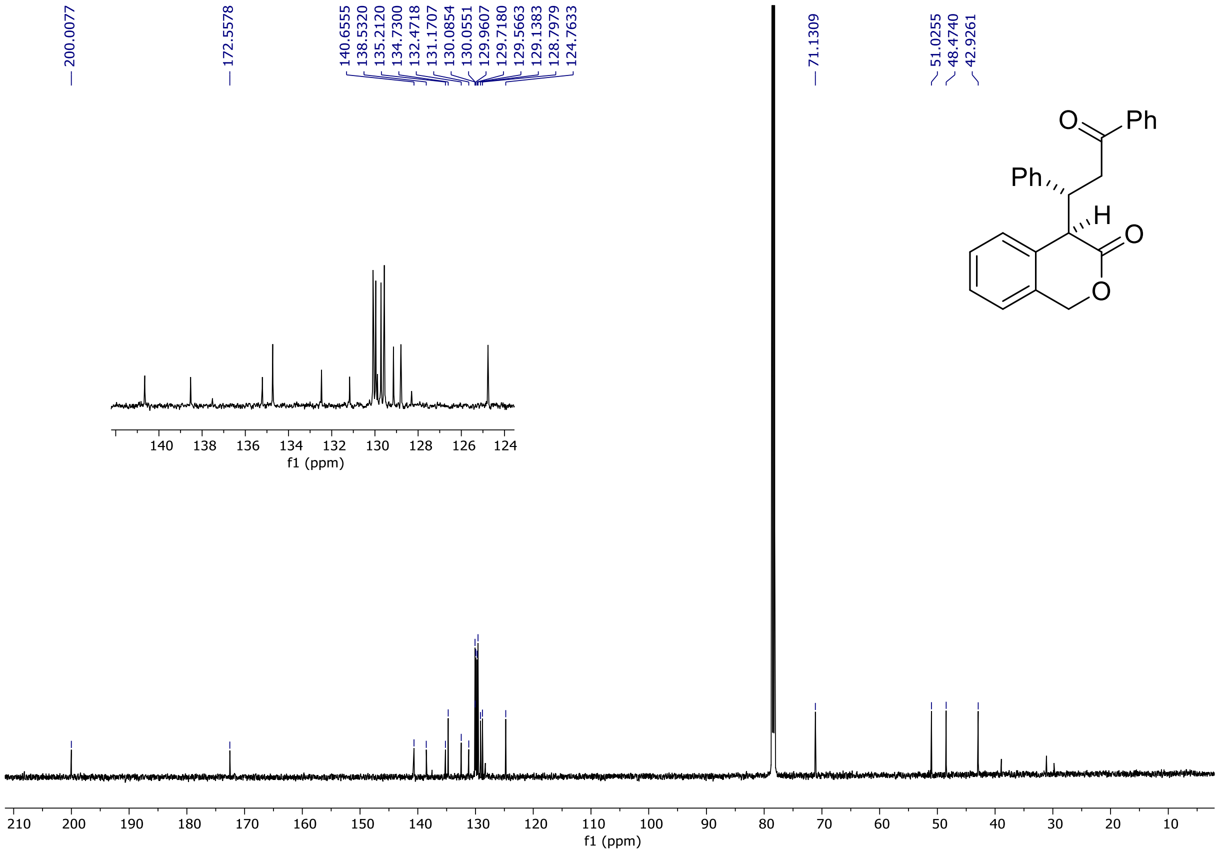

(R*)-4-((R*)-3-oxo-1,3-diphenylpropyl)isochroman-3-one (14a-2)

^{1}H NMR (400 MHz, $CDCl_3$)

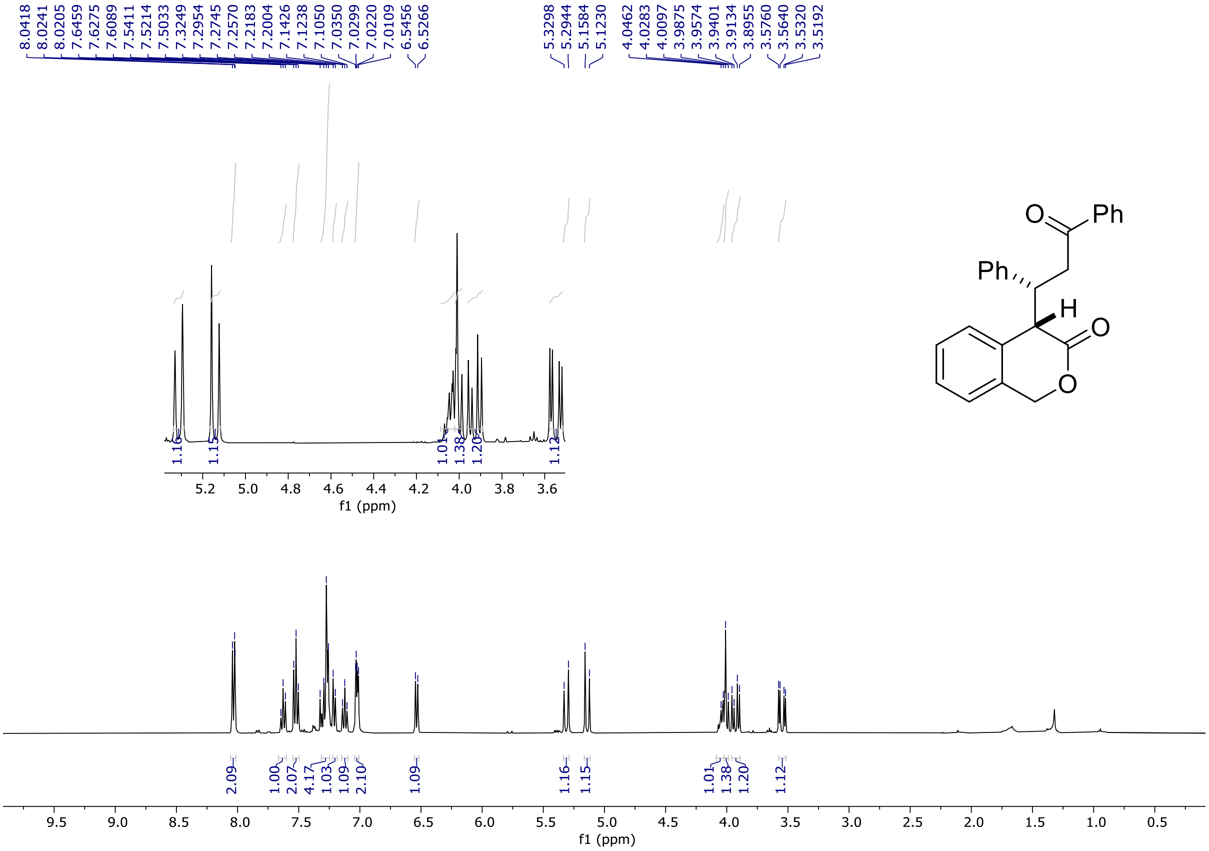

^{13}C{^{1}H} NMR (100 MHz, $CDCl_3$)

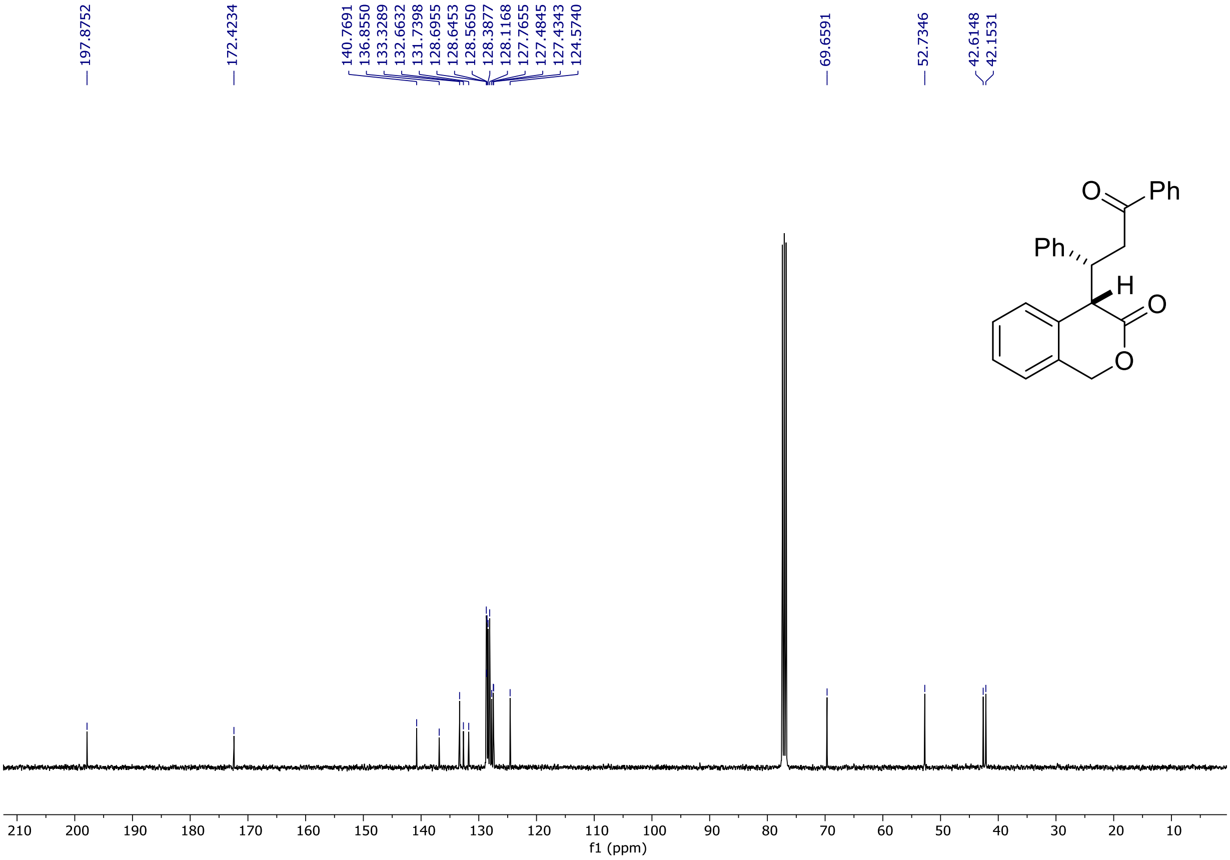

(R*)-4-((R*)-1-(4-chlorophenyl)-2-oxo-2-phenylethyl)isochroman-3-one (14b-1)

^{1}H NMR (400 MHz, $CDCl_3$)

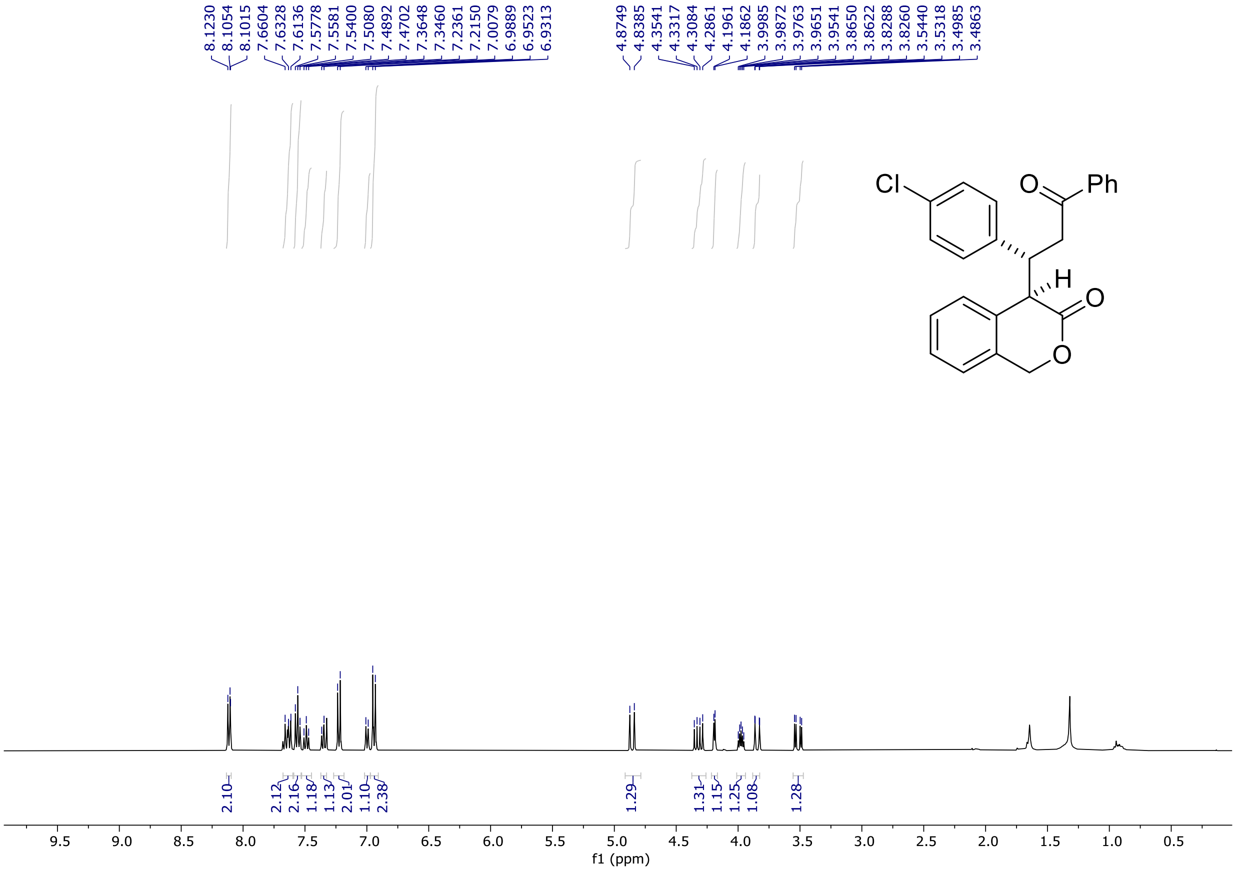

^{13}C{^{1}H} NMR (63 MHz, $CDCl_3$)

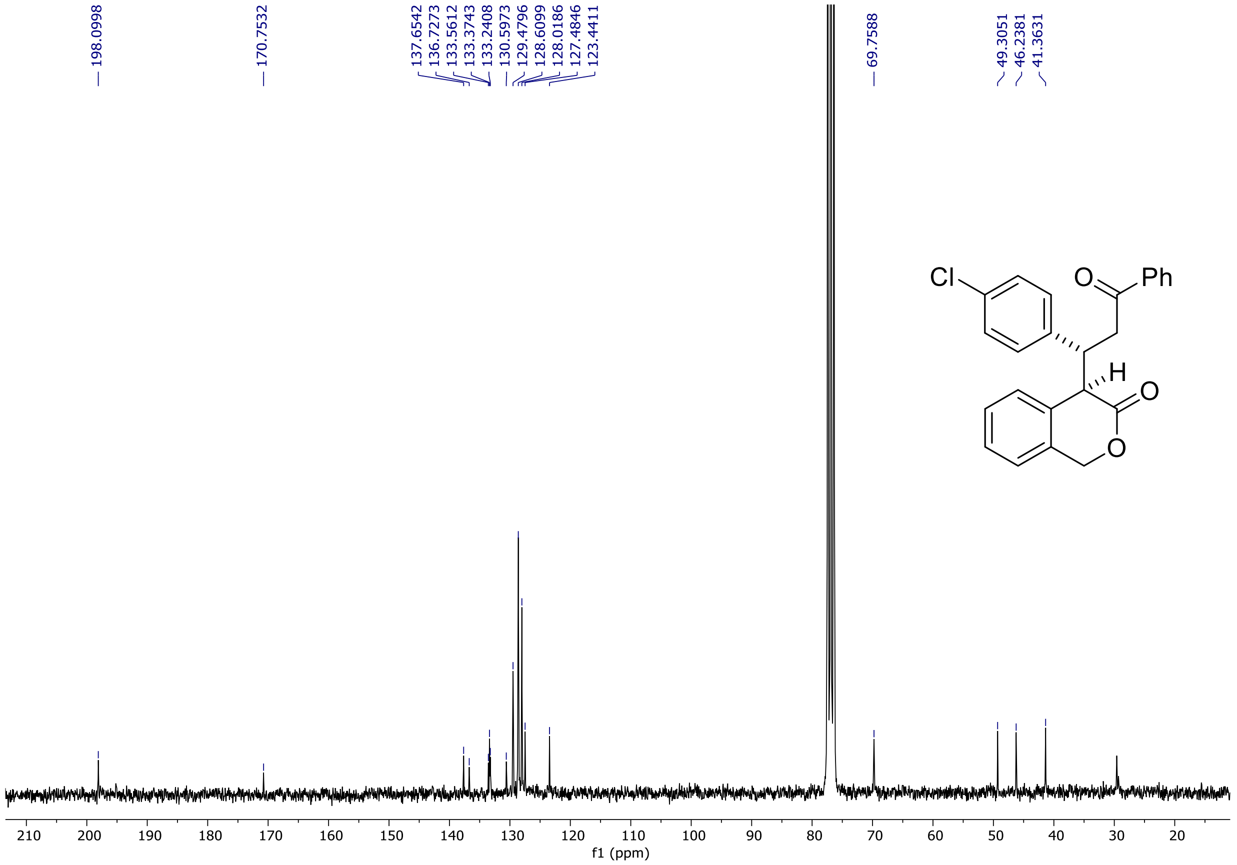

(S*)-4-((S*)-1-(4-chlorophenyl)-2-oxo-2-phenylethyl)isochroman-3-one (14b-2)

^{1}H NMR (400 MHz, $CDCl_3$)

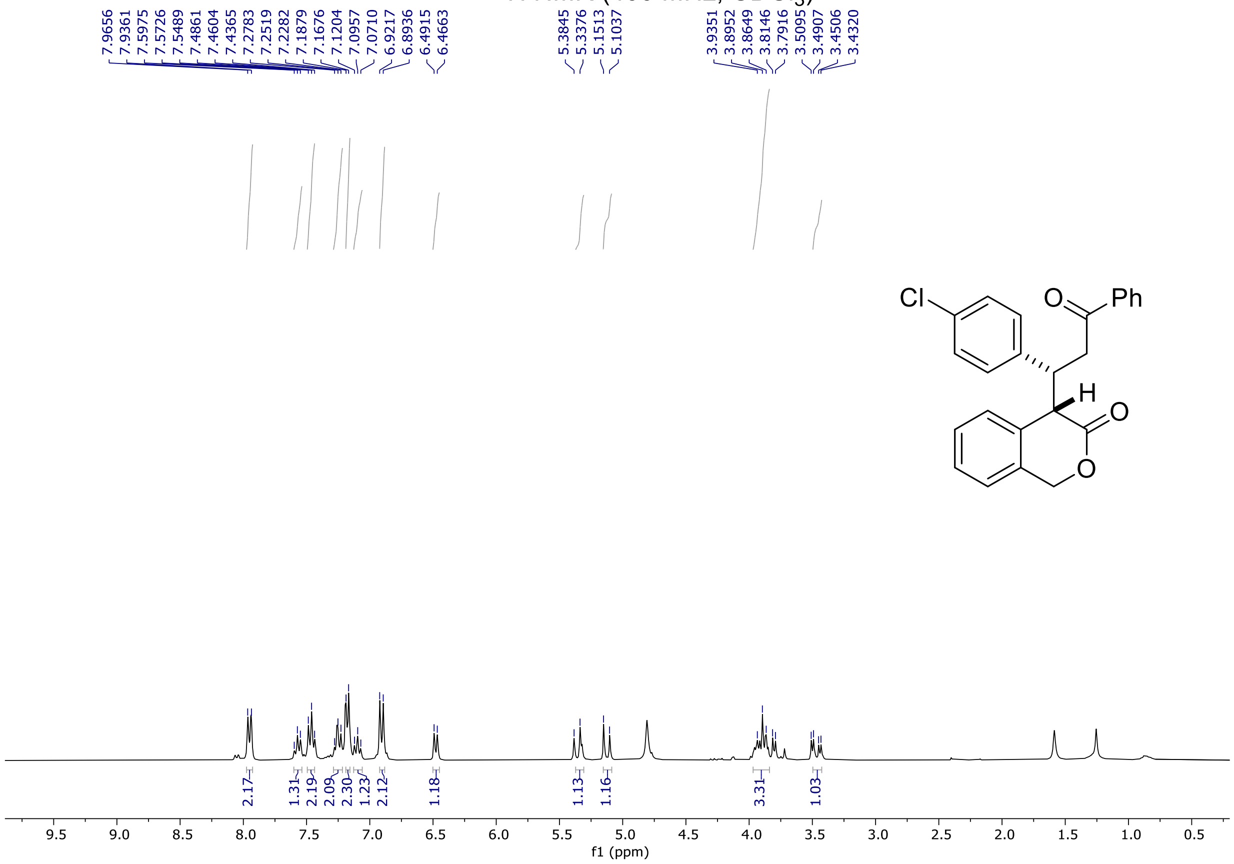

^{13}C{^{1}H} NMR (100 MHz, $CDCl_3$)

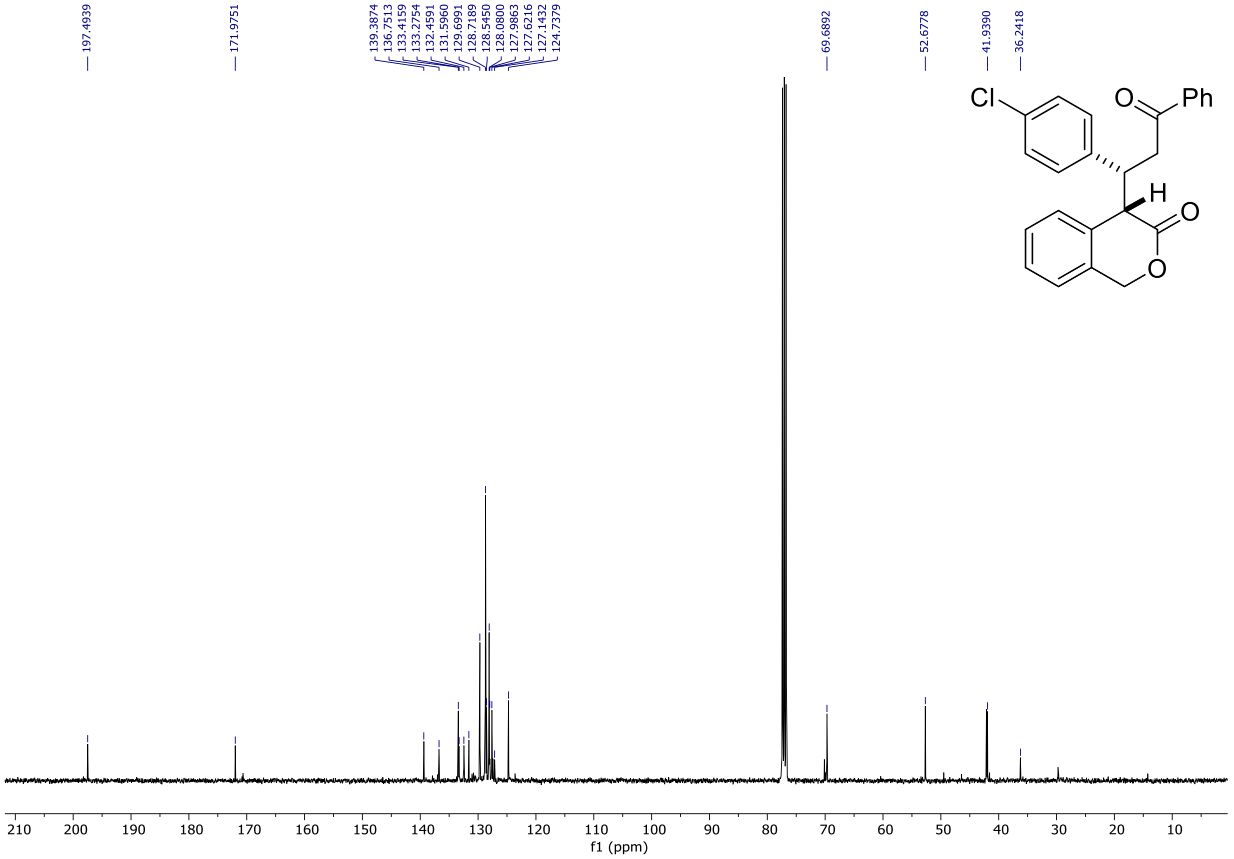

4-(1-(2-nitrophenyl)-3-oxo-3-phenylpropyl)isochroman-3-one (14c)

^{1}H NMR (400 MHz, CDCl$_3$)

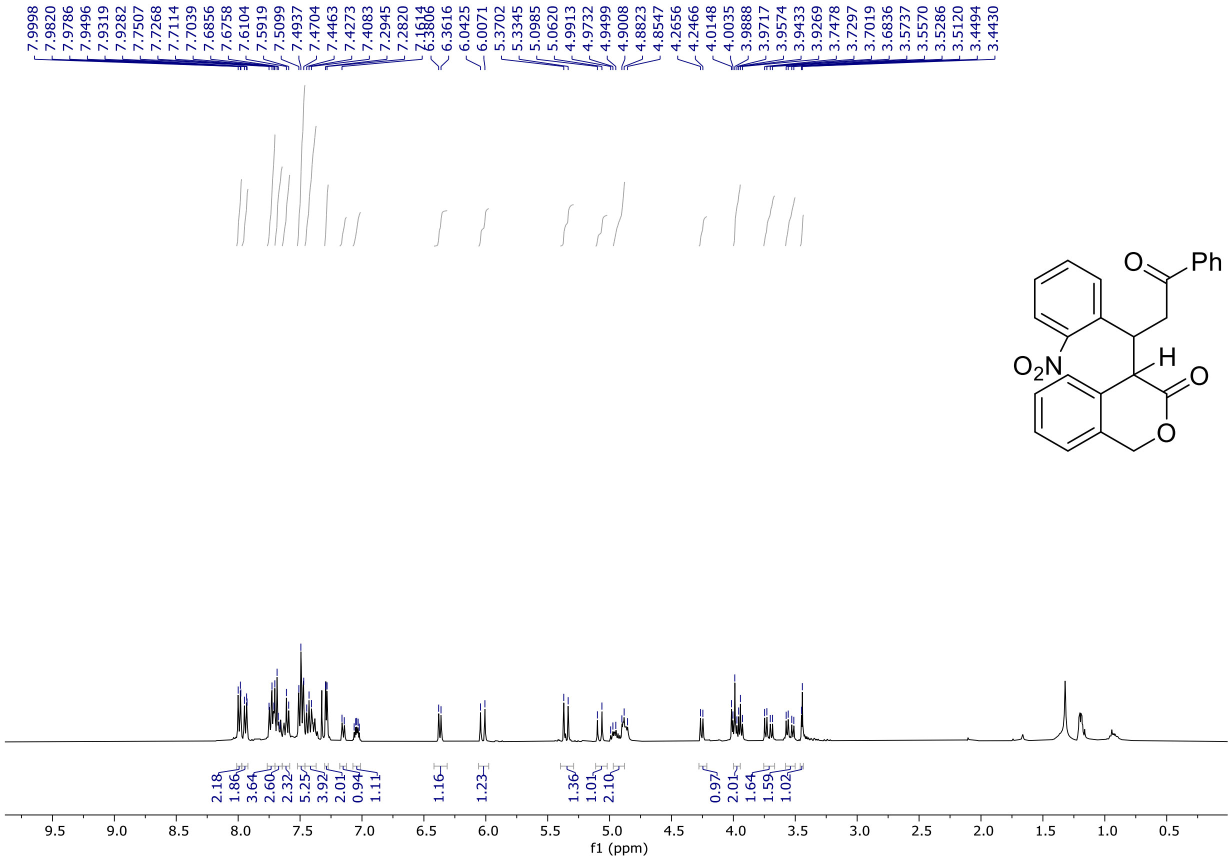

^{13}C{^{1}H} NMR (63 MHz, CDCl$_3$)

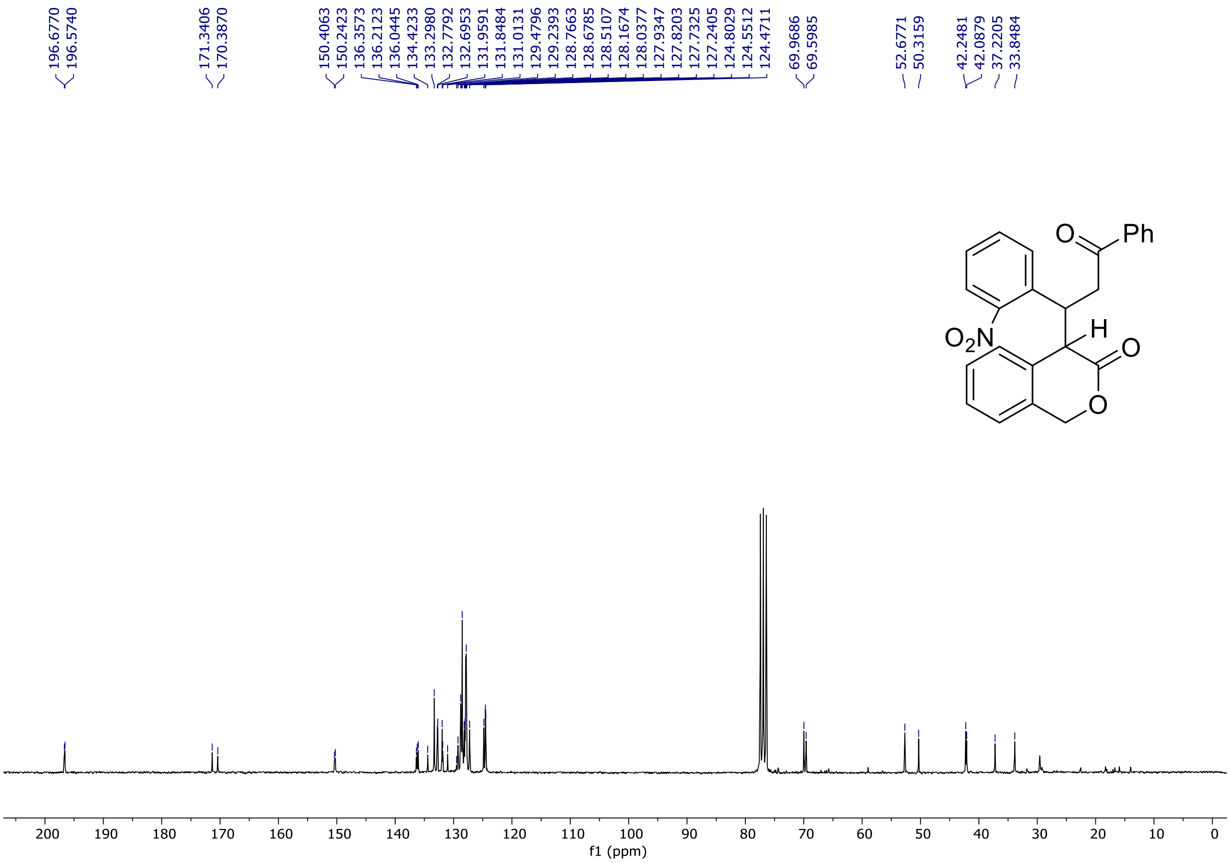

1-(2-chlorophenyl)-3-oxo-3-phenylpropyl)isochroman-3-one (14d)

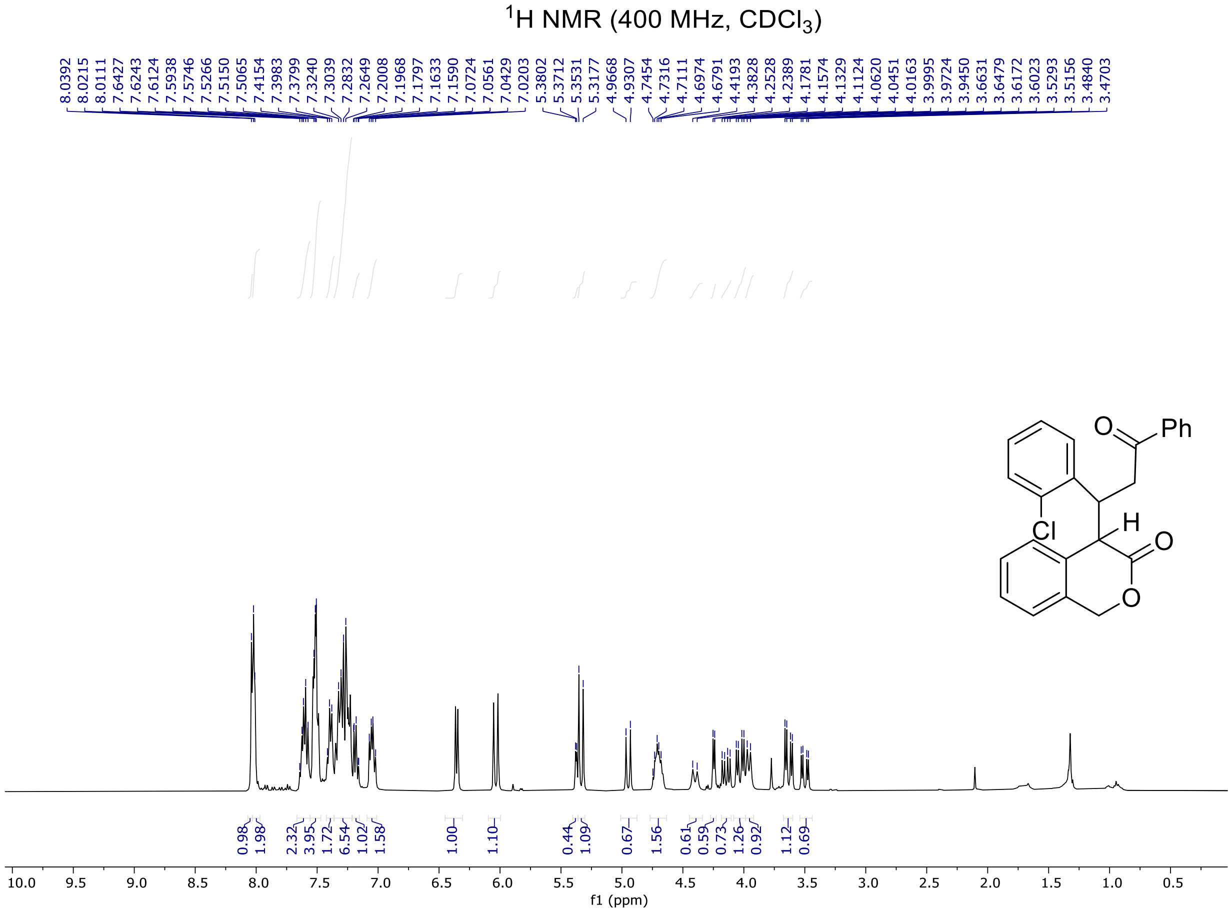

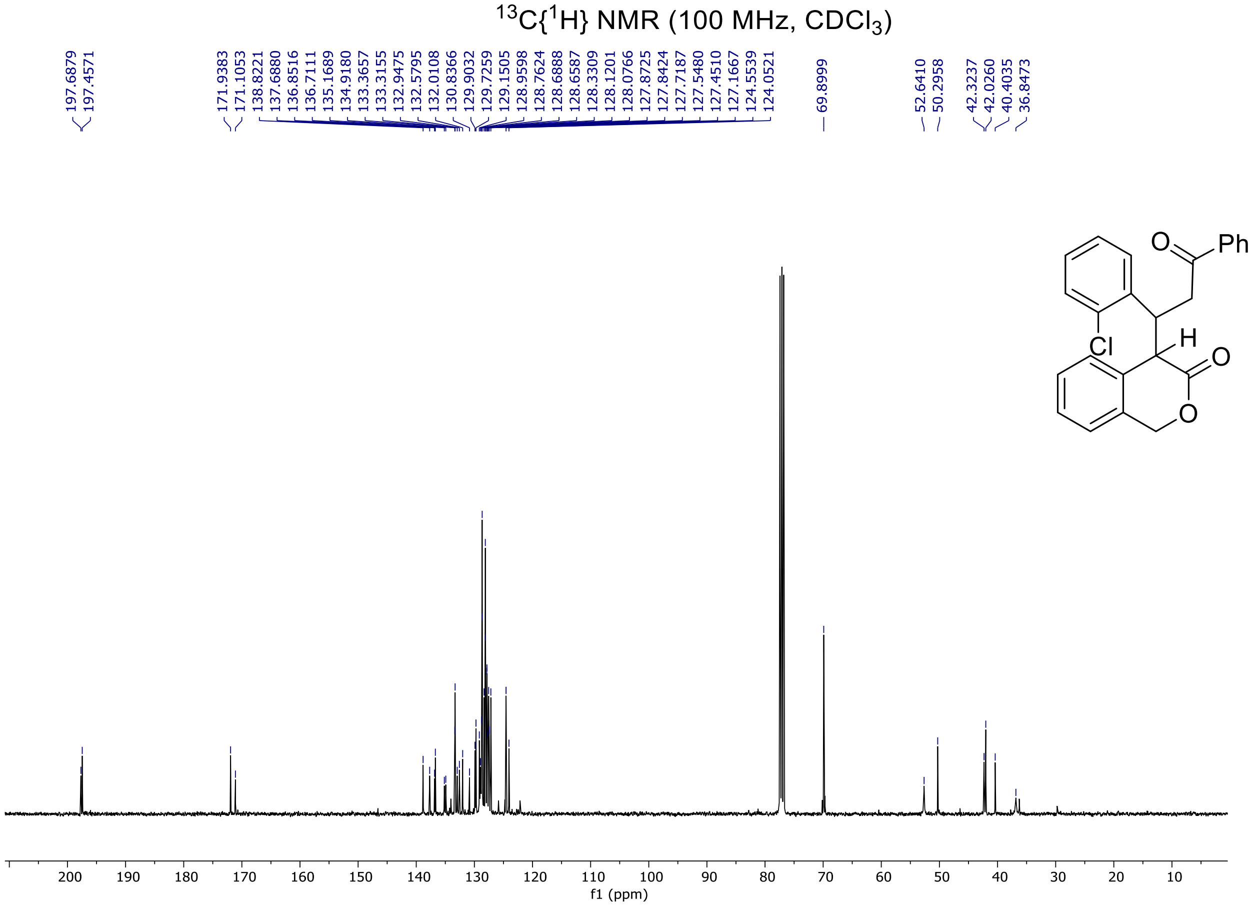

(R*)-4-((R*)-1-(4-fluorophenyl)-3-oxo-3-phenylpropyl)isochroman-3-one (14e-1)

^{1}H NMR (400 MHz, $CDCl_3$)

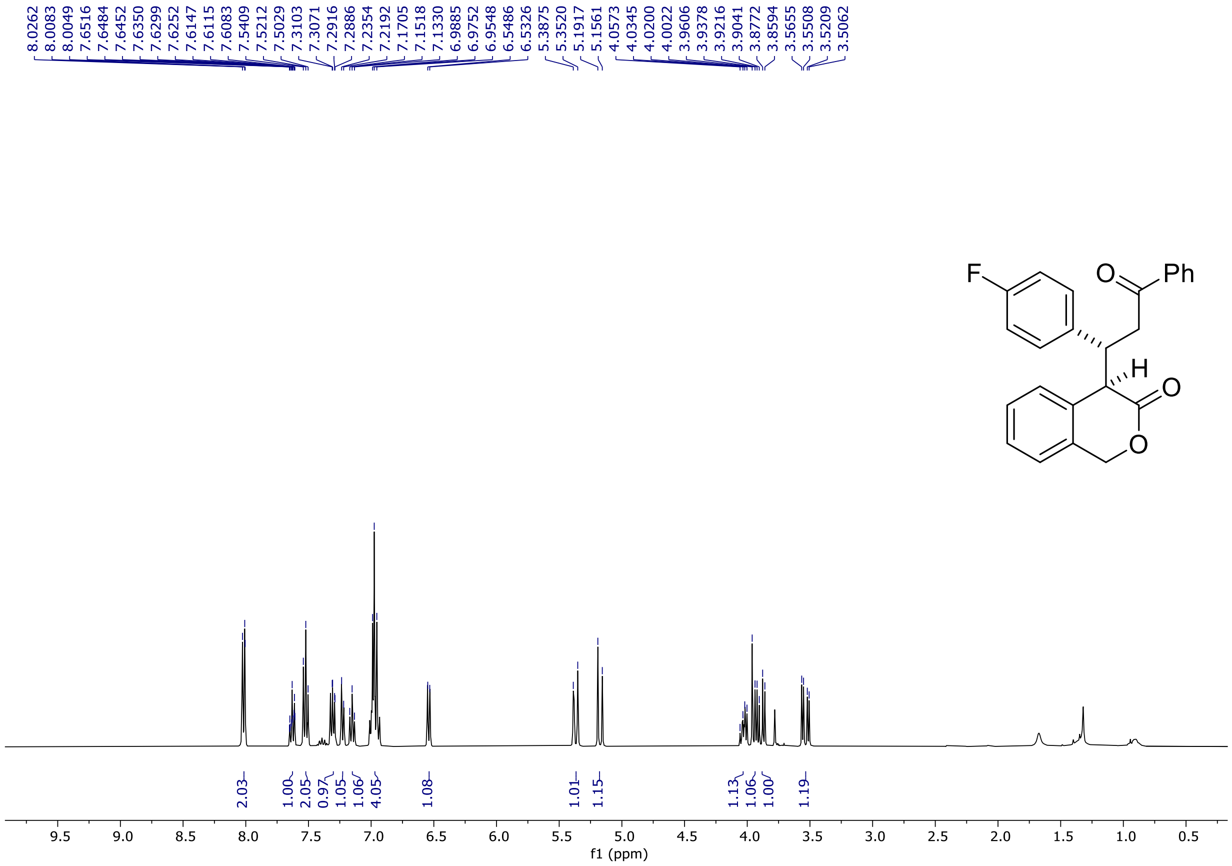

$^{13}C\{^{1}H\}$ NMR (100 MHz, $CDCl_3$)

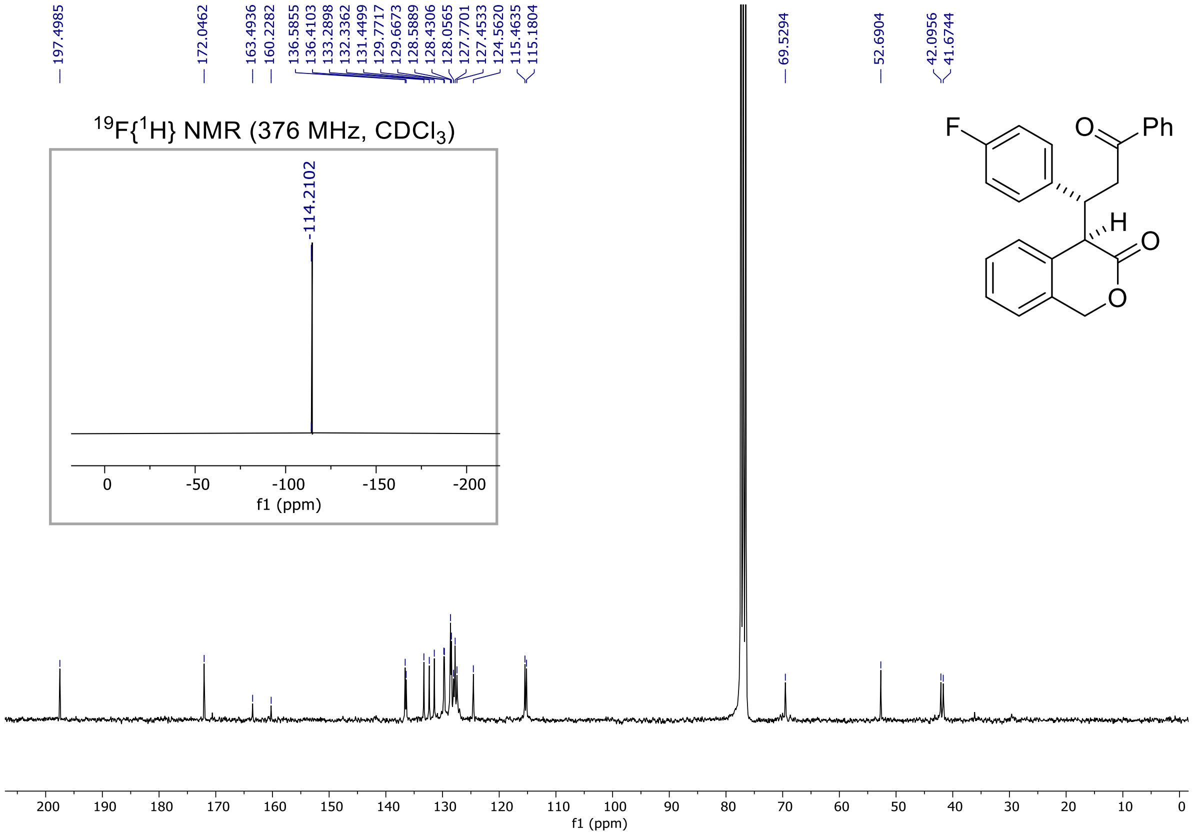

(R*)-4-((S*)-1-(4-fluorophenyl)-3-oxo-3-phenylpropyl)isochroman-3-one (14e-2)

^{1}H NMR (400 MHz, $CDCl_3$)

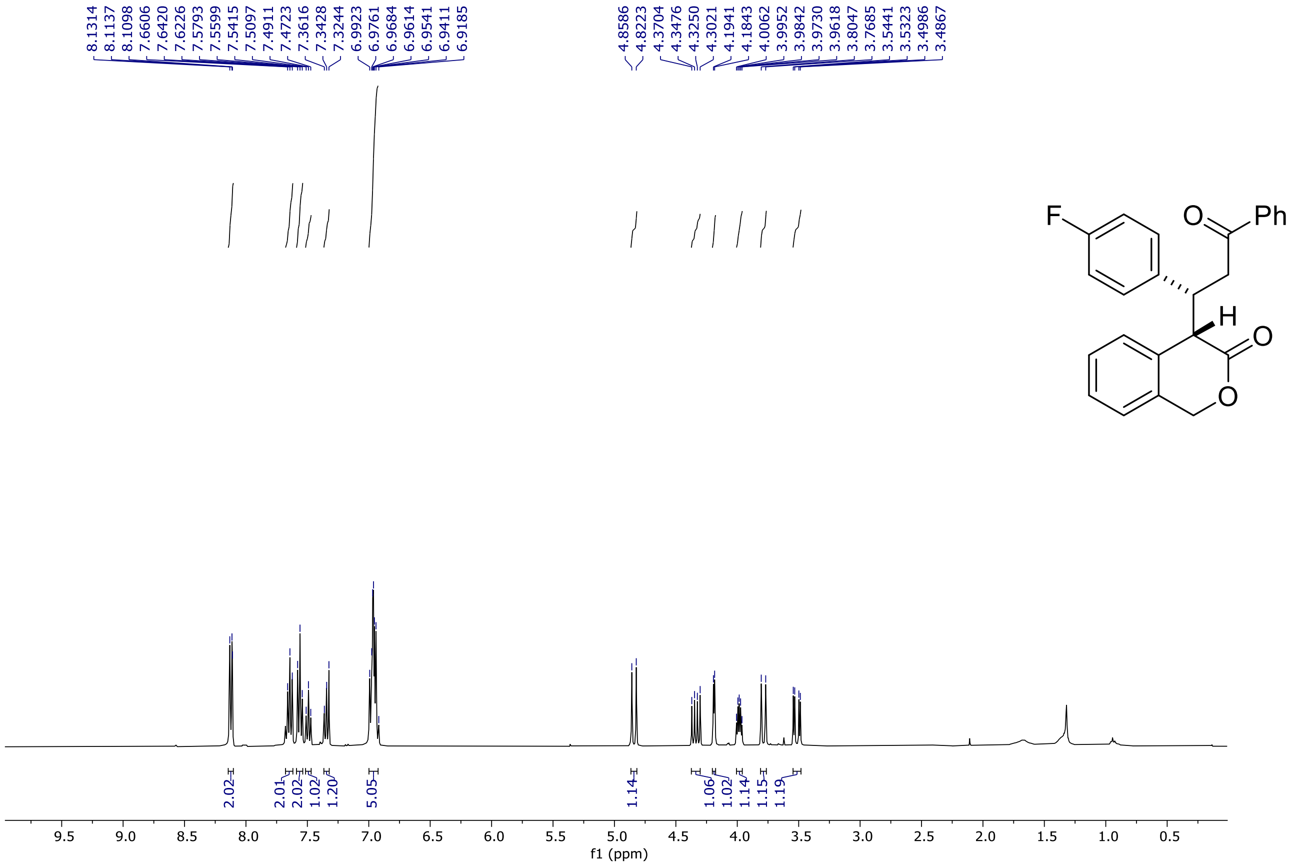

^{13}C{^{1}H} NMR (75 MHz, $CDCl_3$)

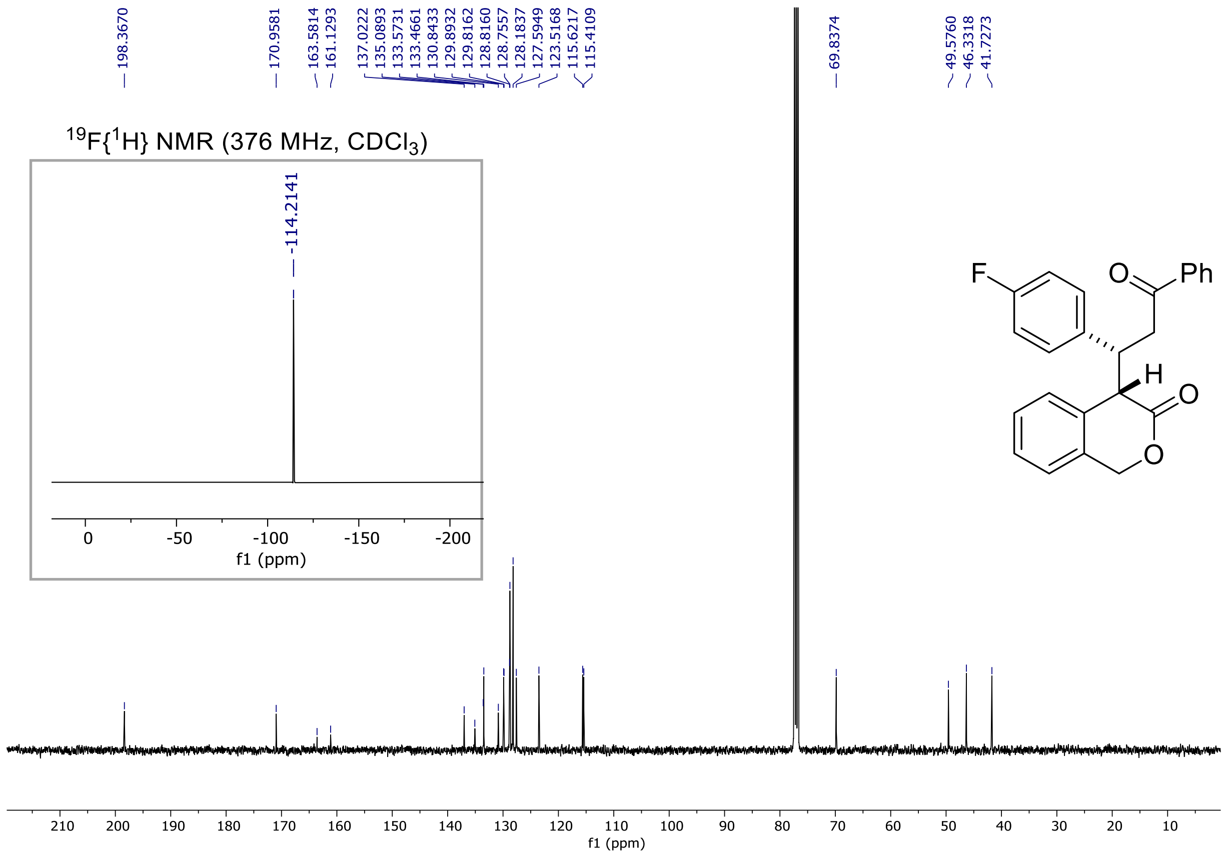

(R*)-4-((R*)-3-(4-methoxyphenyl)-3-oxo-1-phenylpropyl)isochroman-3-one (14f-1)

^{1}H NMR (400 MHz, $CDCl_3$)

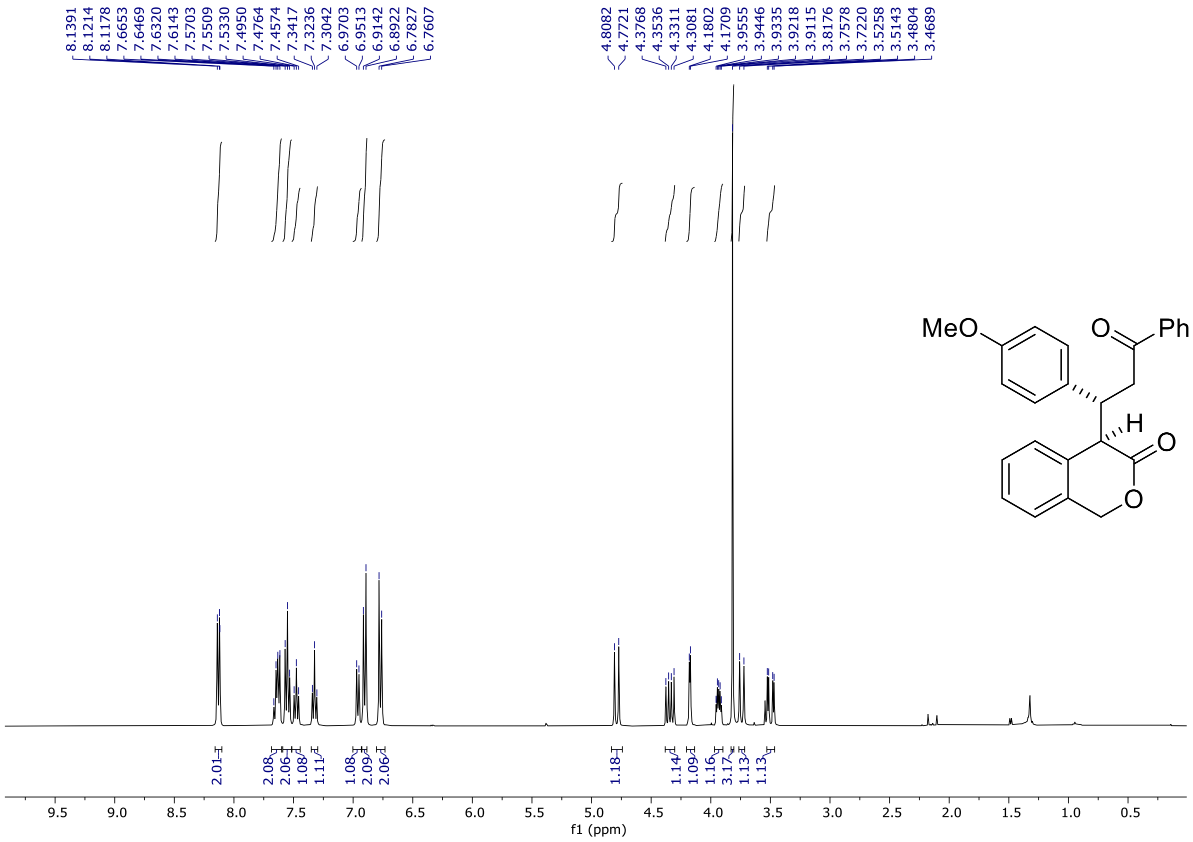

$^{13}C\{^1H\}$ NMR (100 MHz, $CDCl_3$)

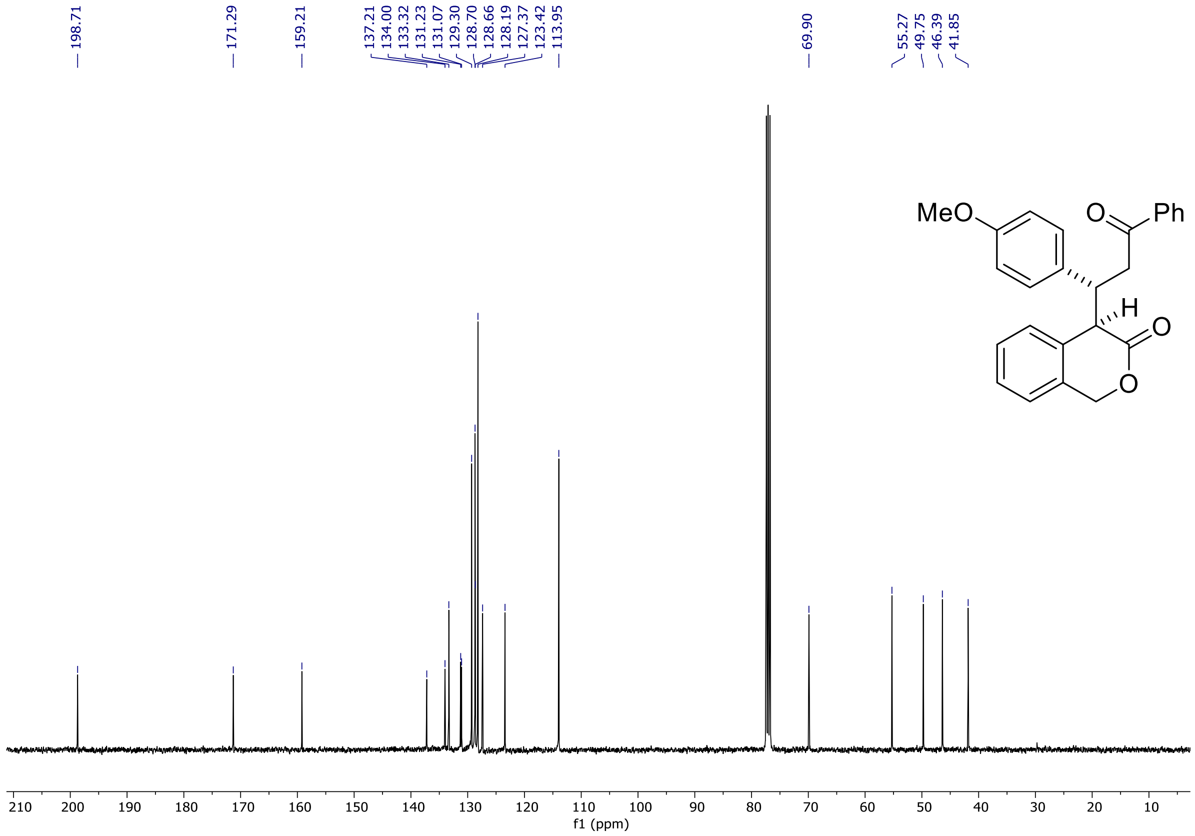

(R*)-4-((S*)-3-(4-methoxyphenyl)-3-oxo-1-phenylpropyl)isochroman-3-one (14f-2)

^{1}H NMR (400 MHz, $CDCl_3$)

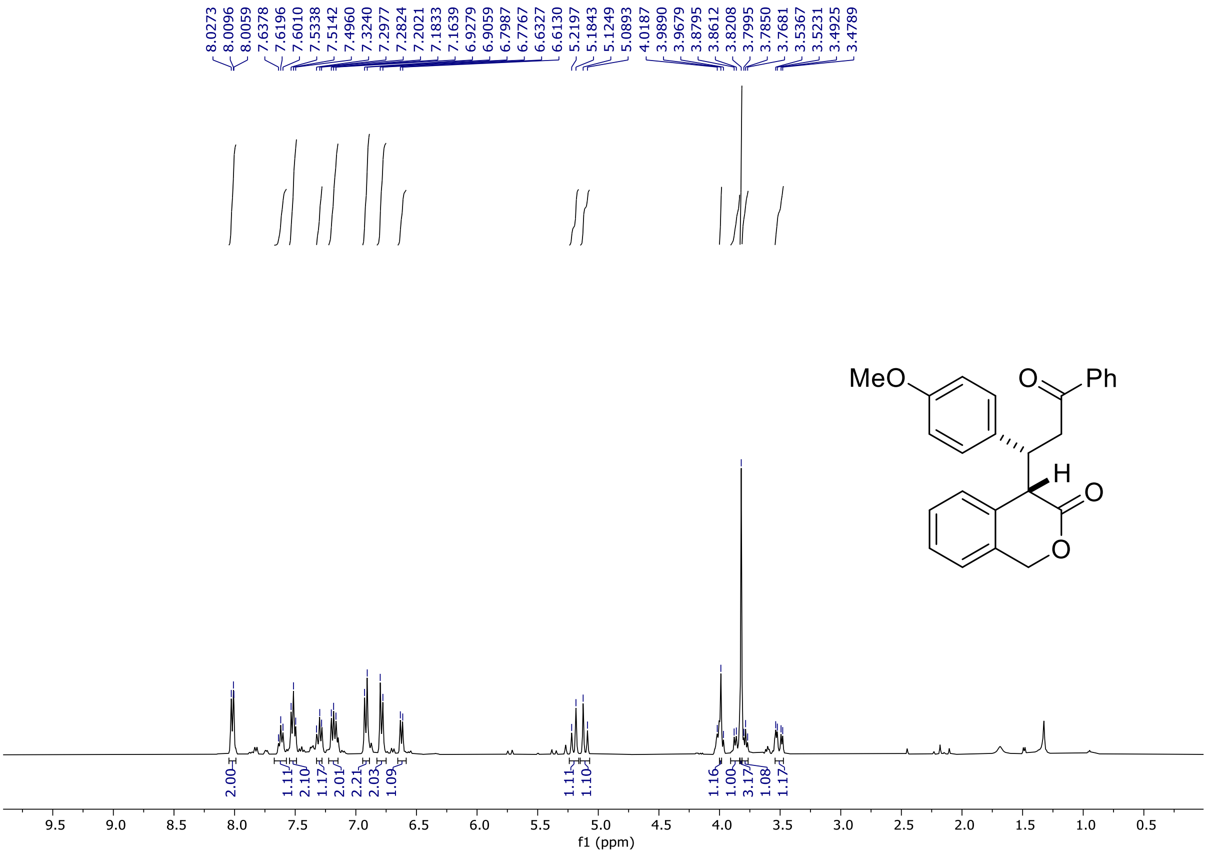

^{13}C{^{1}H} NMR (100 MHz, $CDCl_3$)

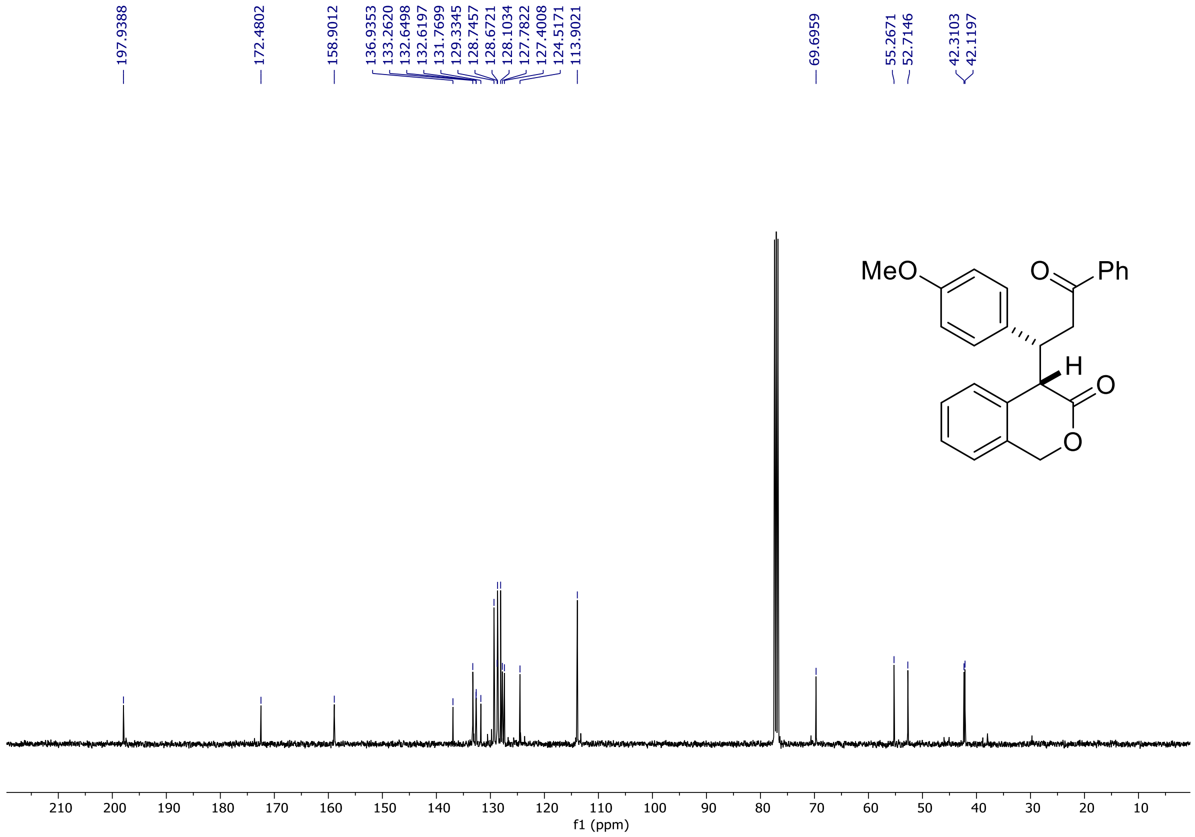

(R*)-4-((R*)-2-oxo-1-phenyl-2-(3-(trifluoromethyl)phenyl)ethyl)isochroman-3-one (14g-1)

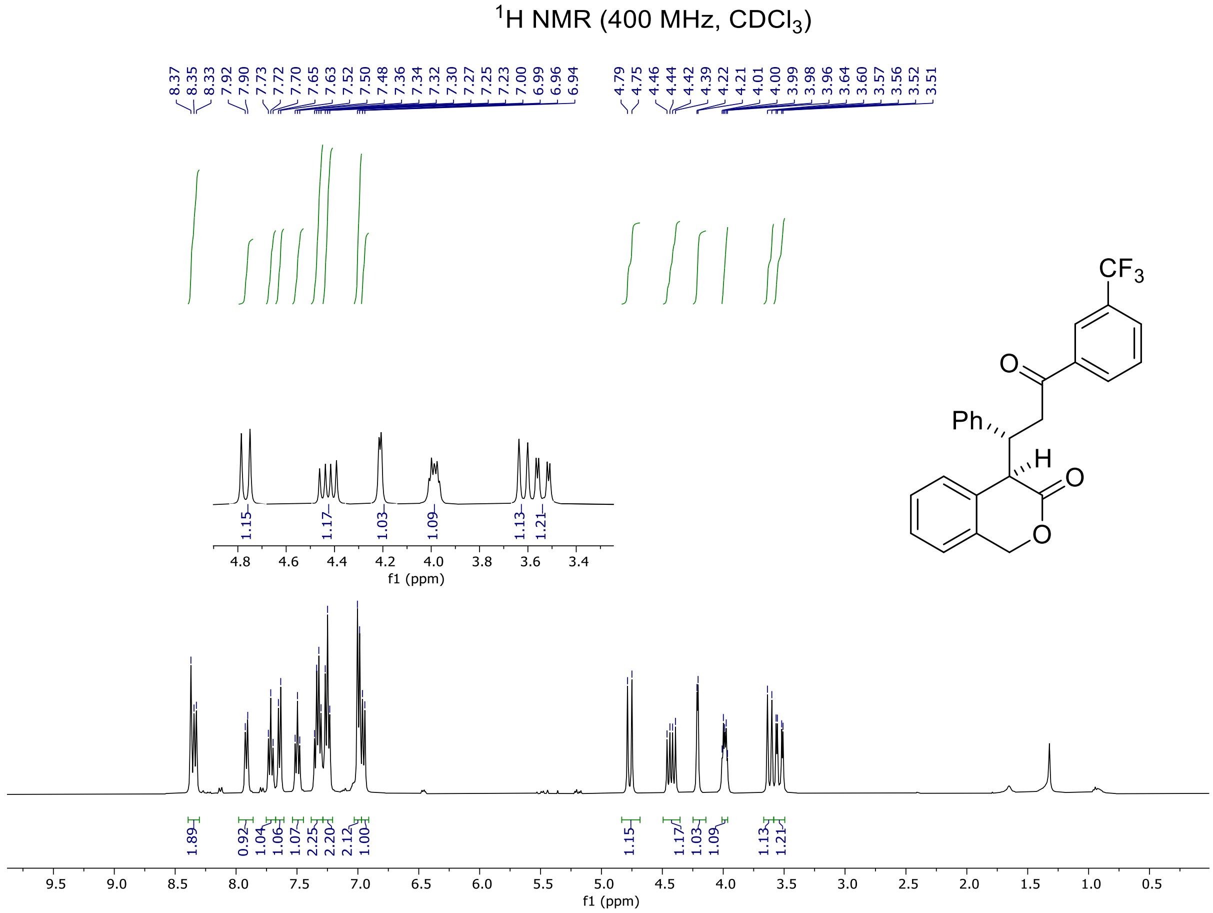

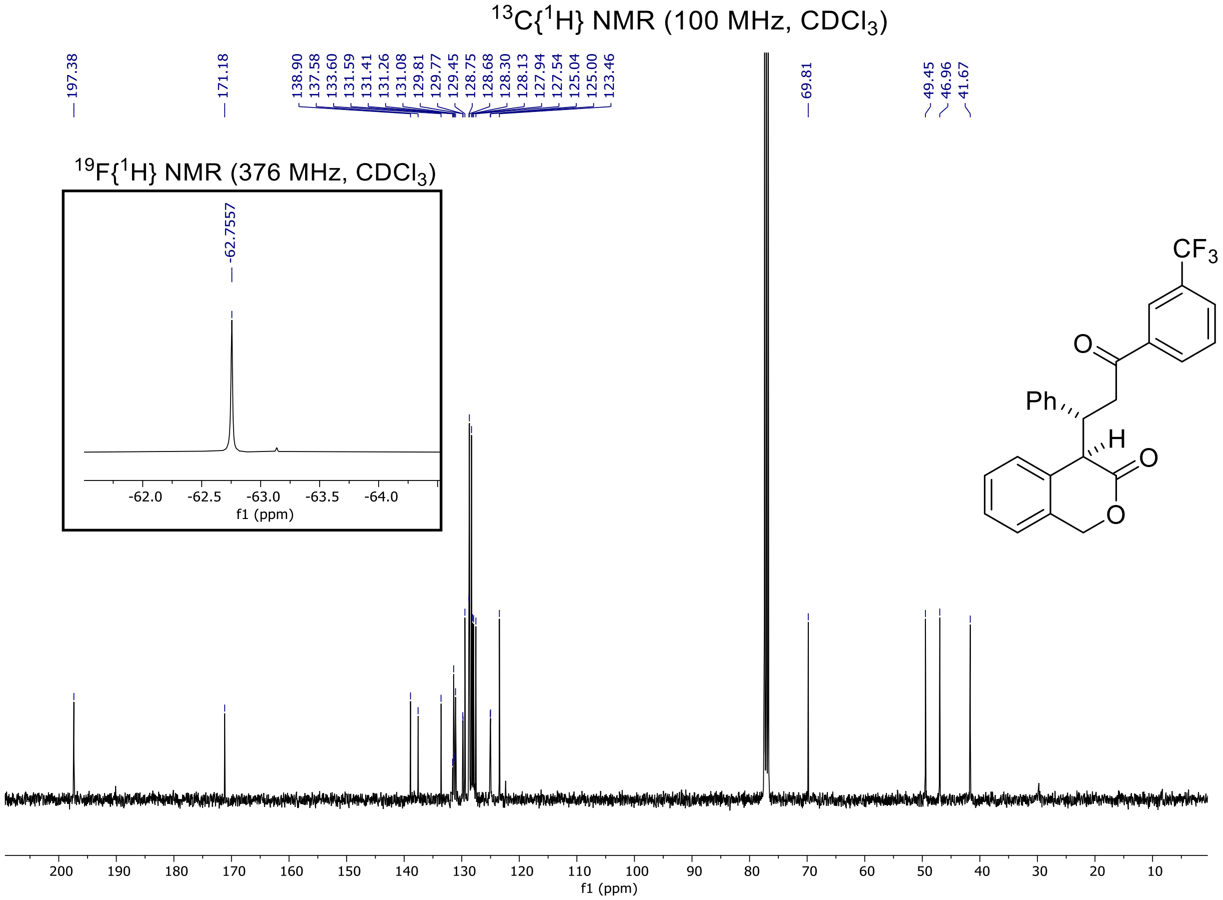

(R*)-4-((S*)-2-oxo-1-phenyl-2-(3-(trifluoromethyl)phenyl)ethyl)isochroman-3-one (14g-2)

^{1}H NMR (400 MHz, $CDCl_3$)

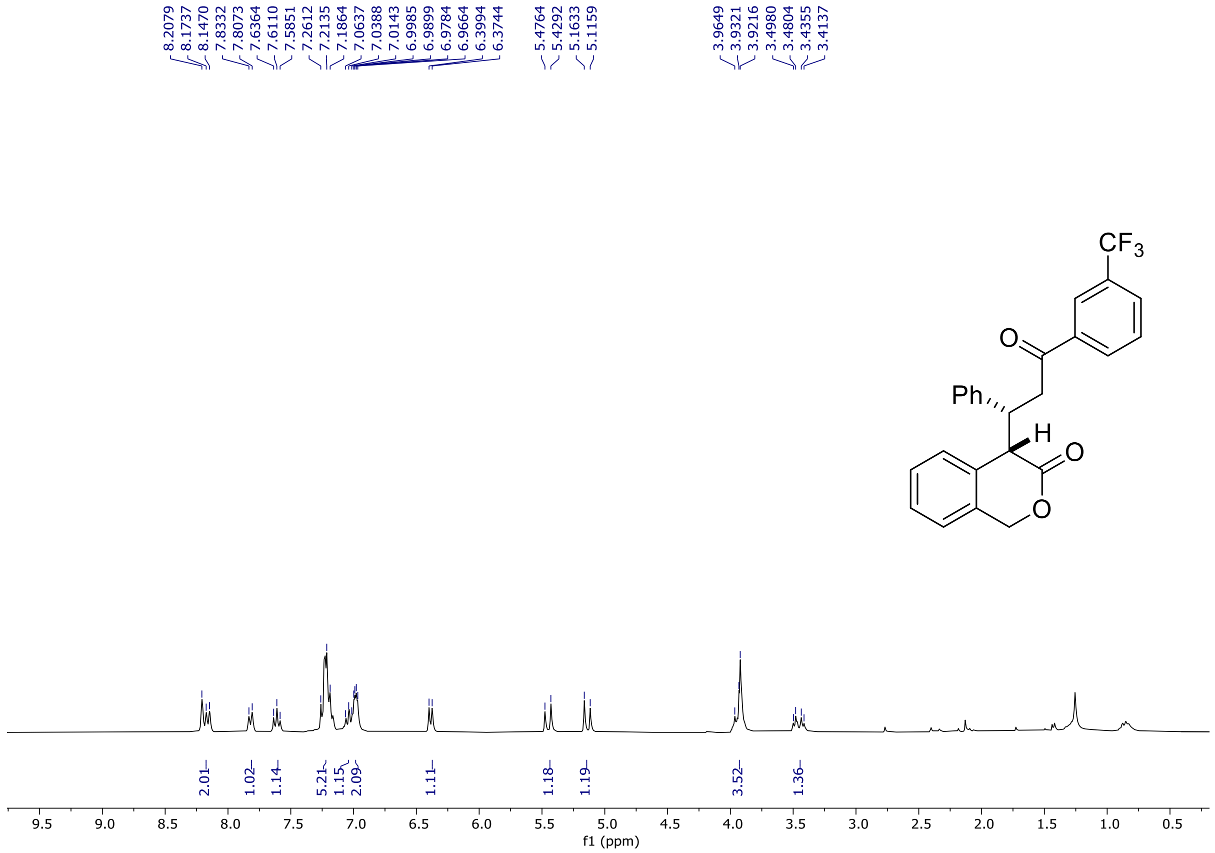

$^{13}C\{^{1}H\}$ NMR (100 MHz, $CDCl_3$)

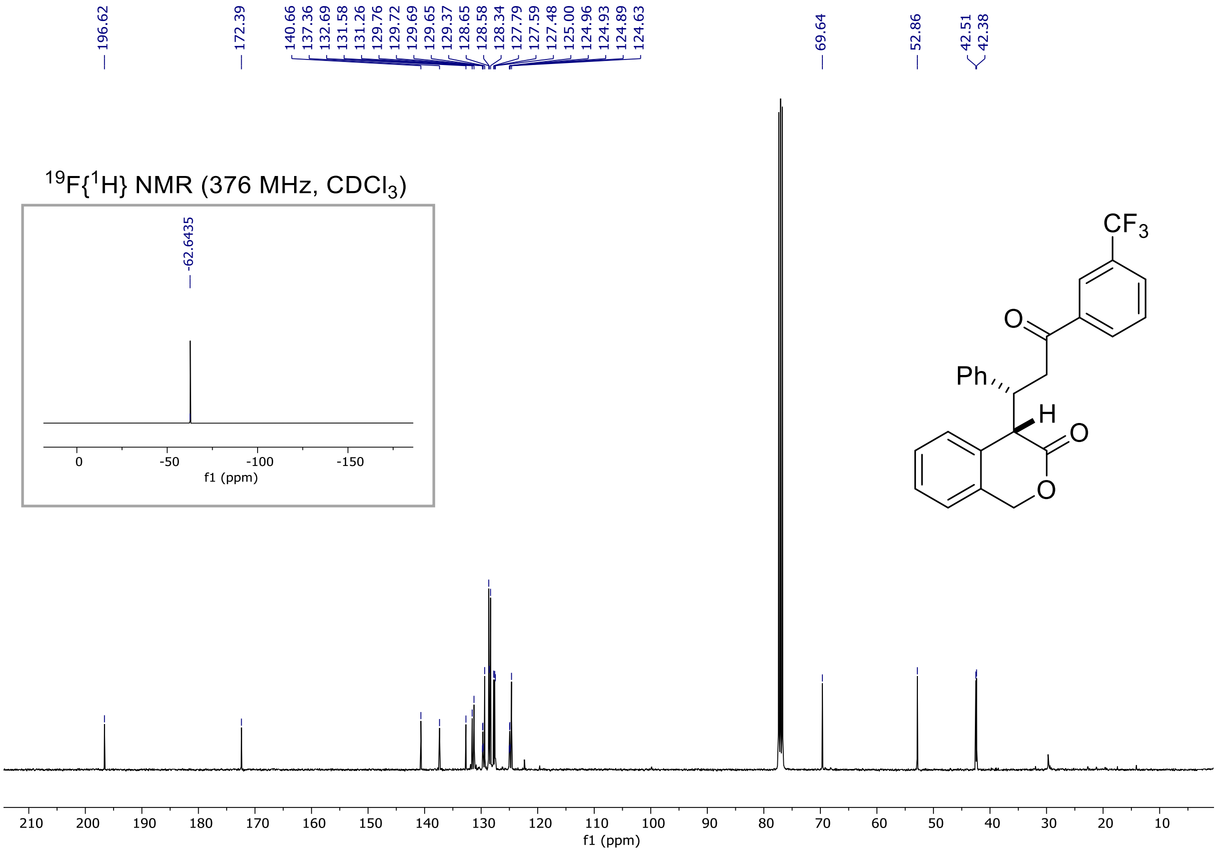

(R*)-4-((R*)-3-(4-nitrophenyl)-3-oxo-1-phenylpropyl)isochroman-3-one (14h)

^{1}H NMR (400 MHz, $CDCl_3$)

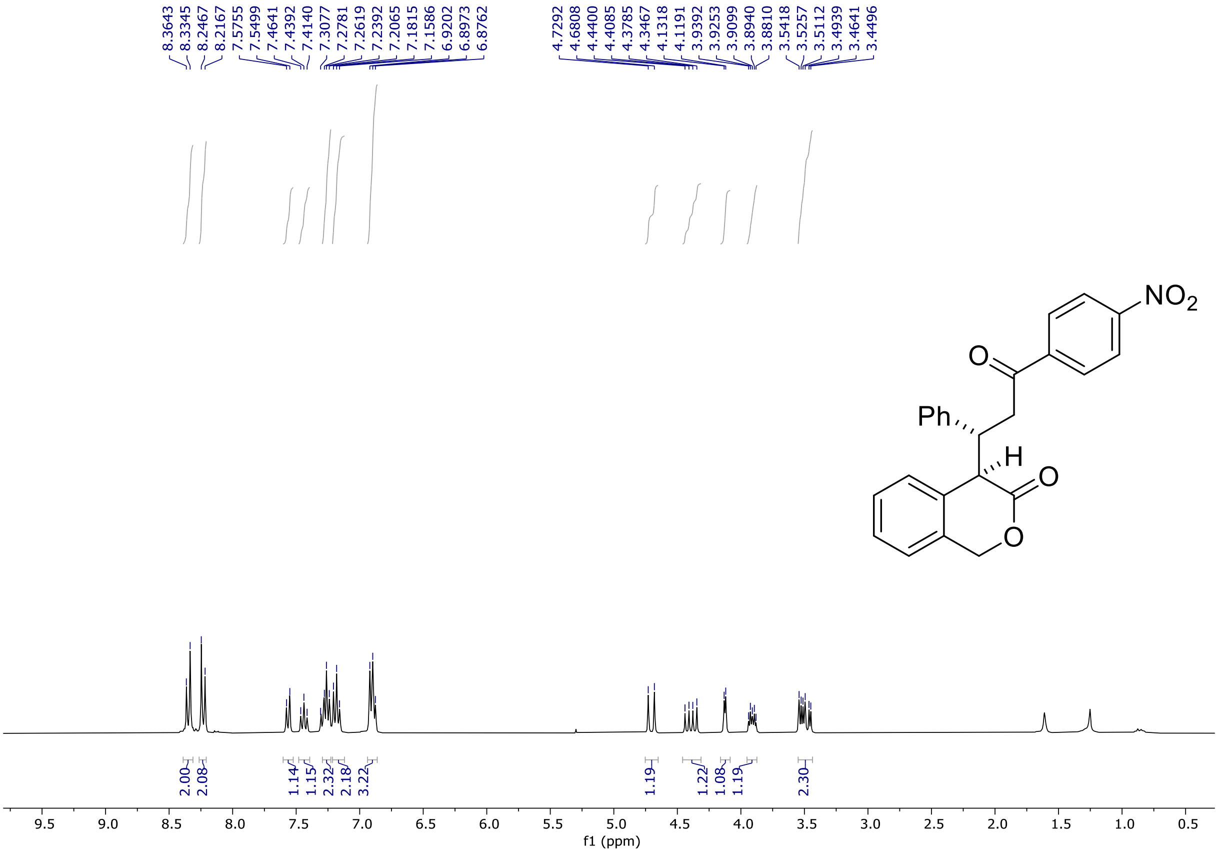

^{13}C{^{1}H} NMR (100 MHz, $CDCl_3$)

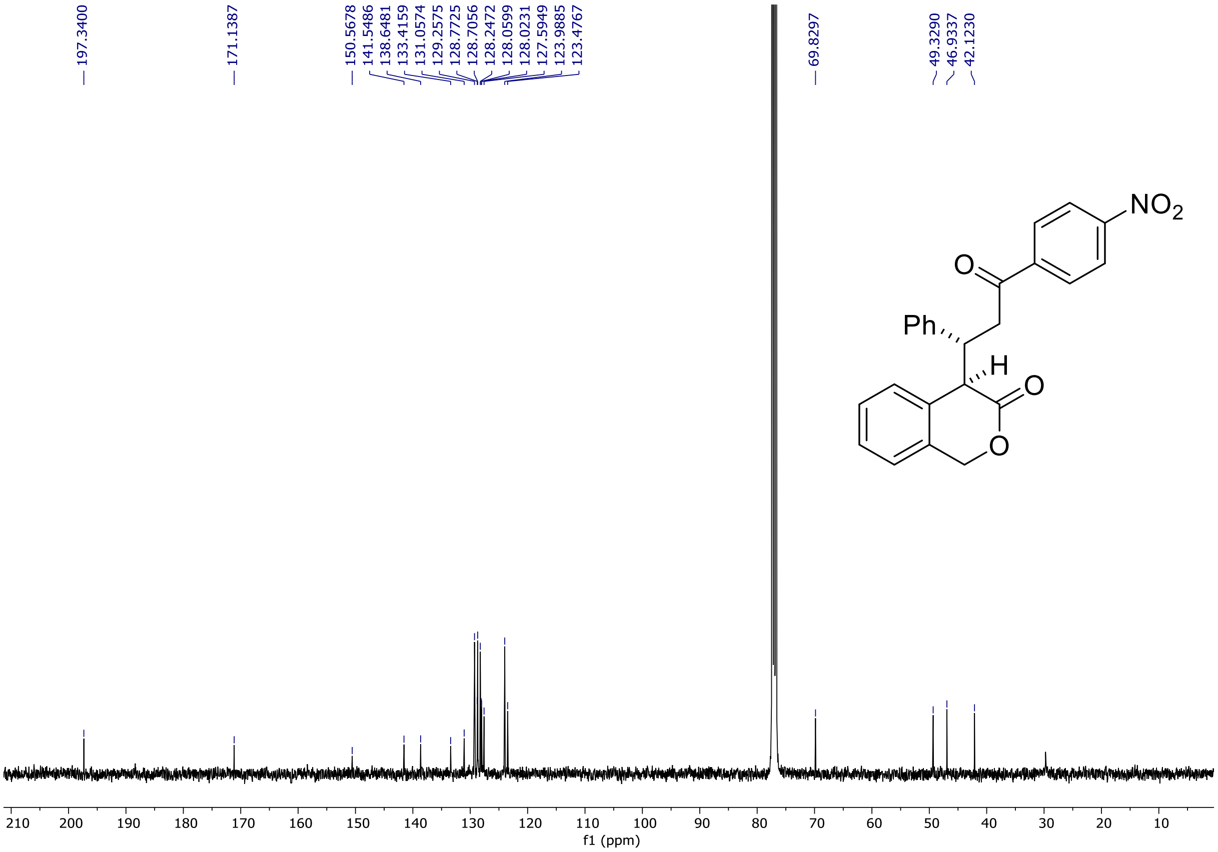

(R*)-4-((R*)-3-(4-methoxyphenyl)-3-oxo-1-phenylpropyl)isochroman-3-one (14i-1)

^{1}H NMR (400 MHz, $CDCl_3$)

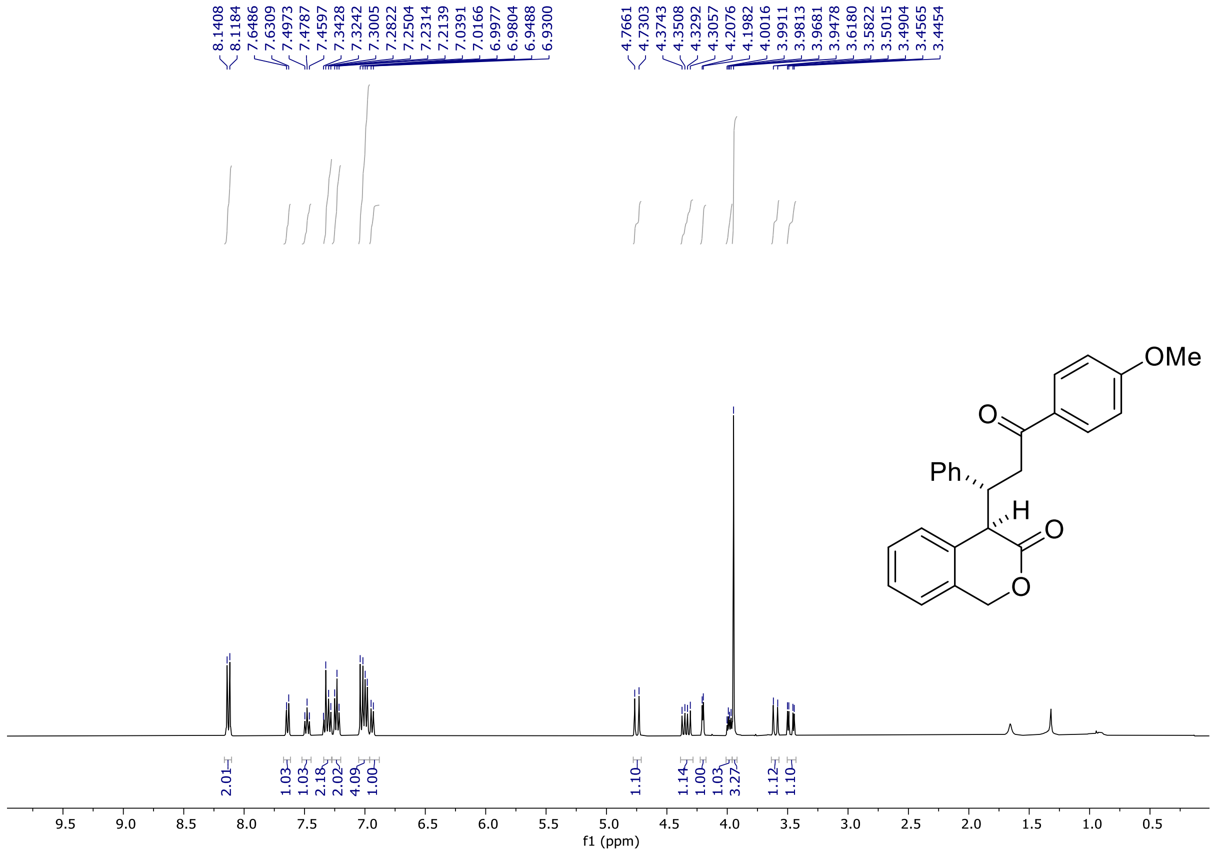

$^{13}C\{^1H\}$ NMR (100 MHz, $CDCl_3$)

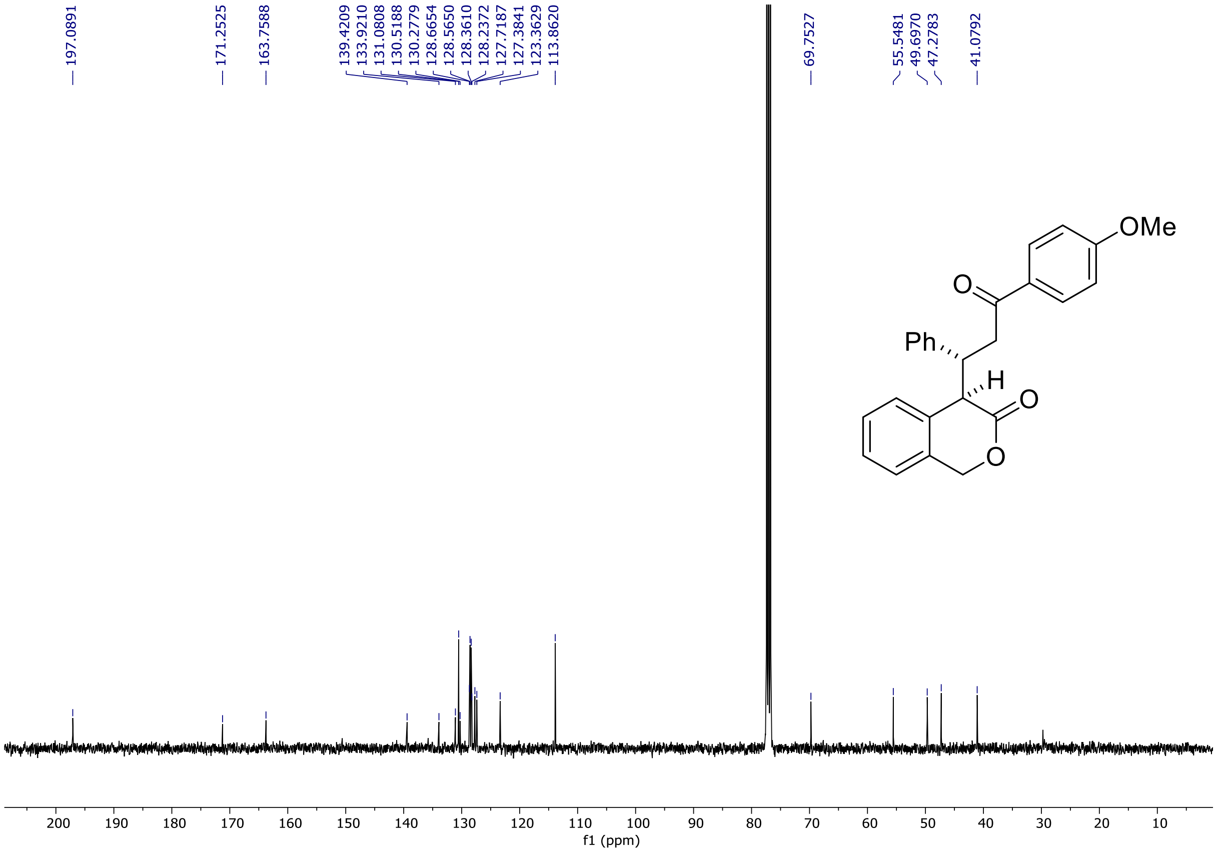

(R*)-4-((S*)-3-(4-methoxyphenyl)-3-oxo-1-phenylpropyl)isochroman-3-one (14i-2)

^{1}H NMR (400 MHz, $CDCl_3$)

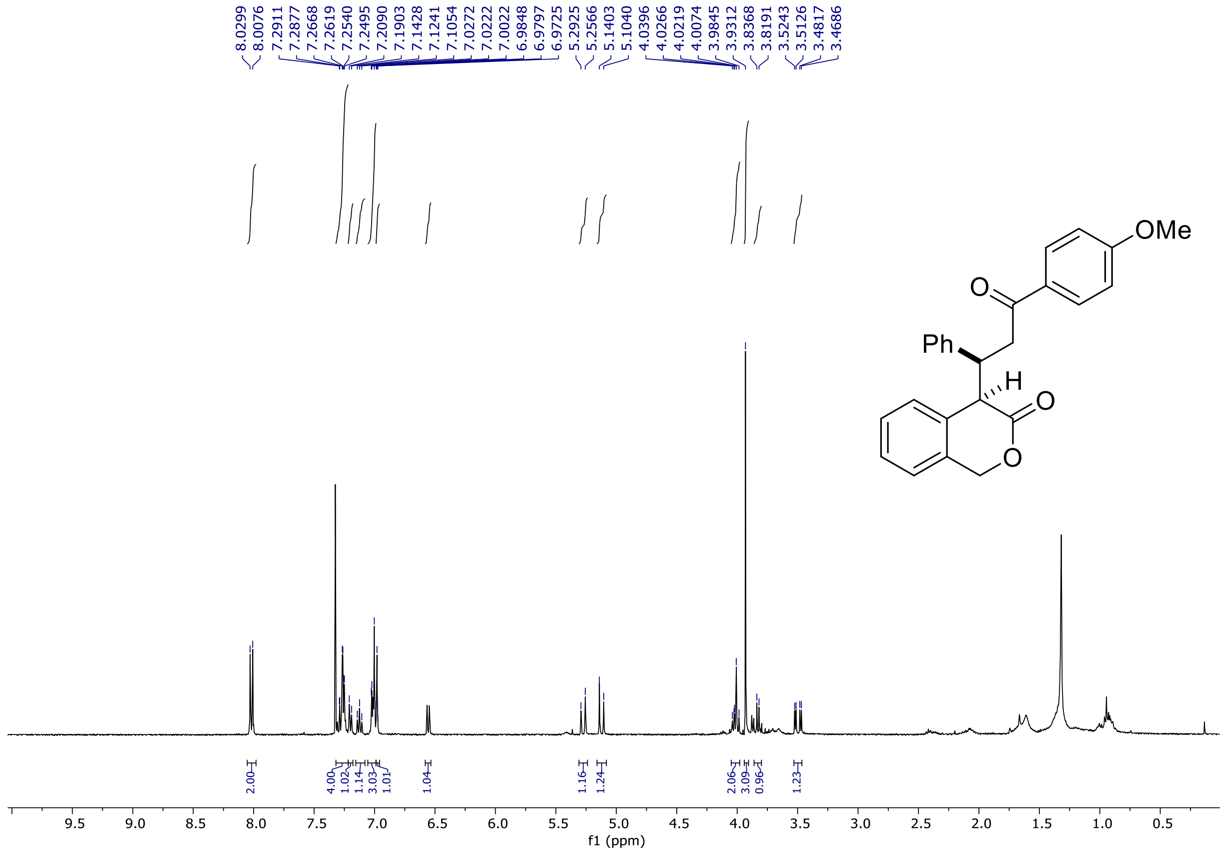

^{13}C{^{1}H} NMR (100 MHz, $CDCl_3$)

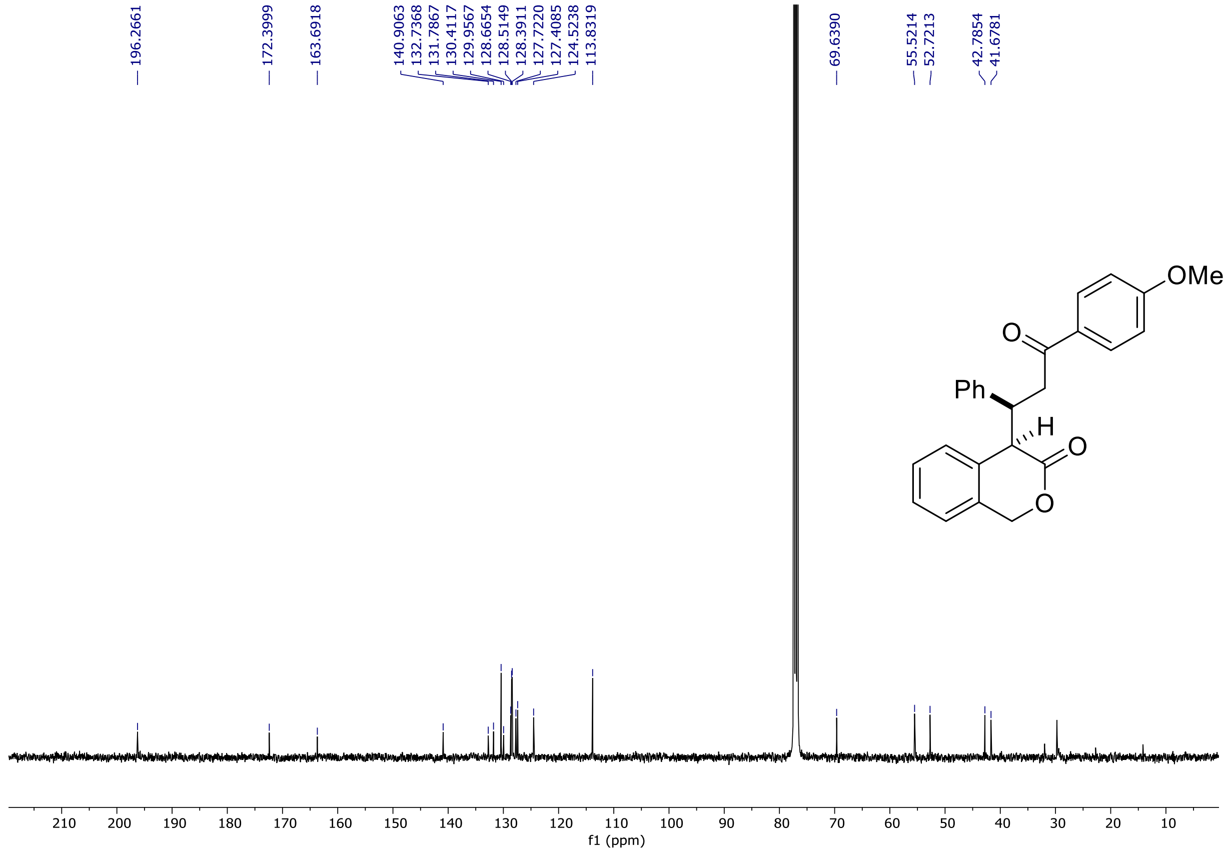

(furan-2-yl)-3-oxo-3-phenylpropyl)isochroman-3-one (14j)

^{1}H NMR (400 MHz, $CDCl_3$)

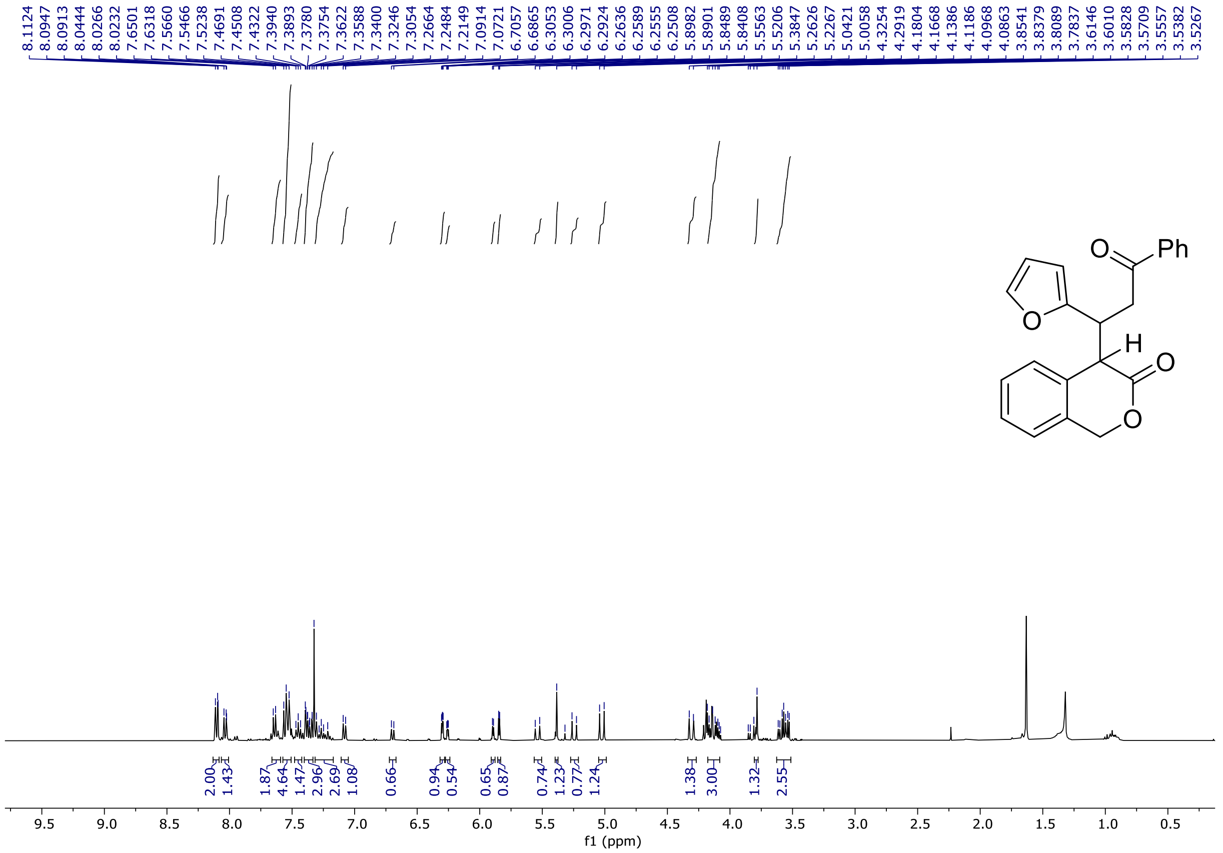

$^{13}C\{^{1}H\}$ NMR (75 MHz, $CDCl_3$)

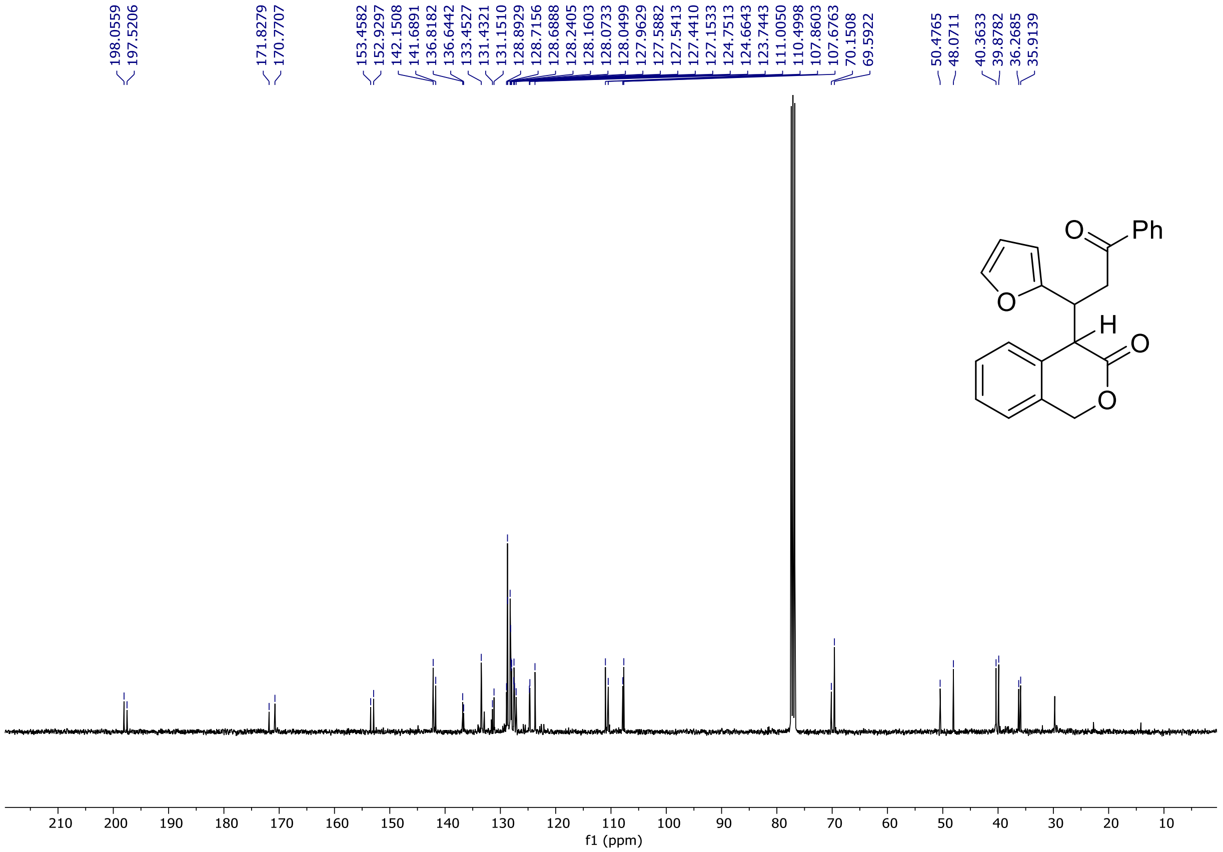

7-bromo-4-(3-oxo-1,3-diphenylpropyl)isochroman-3-one (14k)

^{1}H NMR (400 MHz, $CDCl_3$)

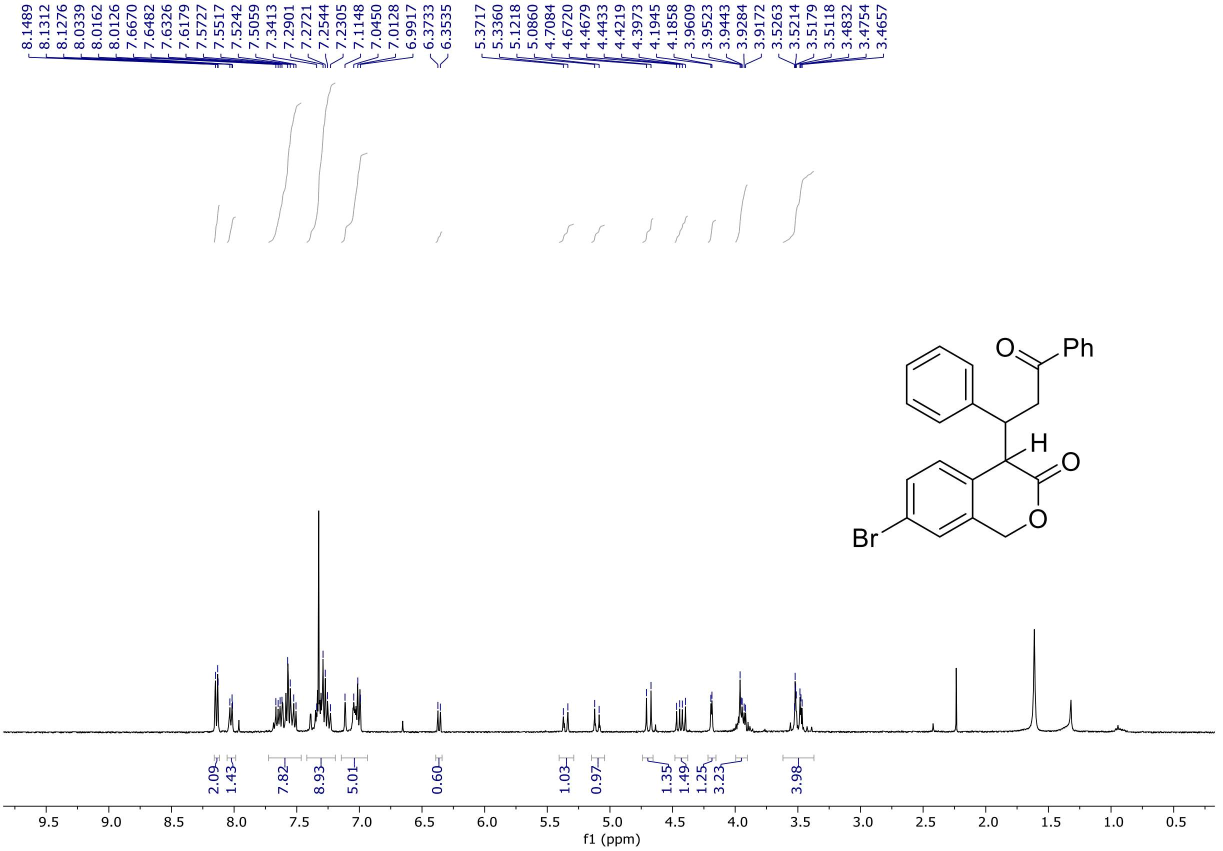

^{13}C{^{1}H} NMR (75 MHz, $CDCl_3$)

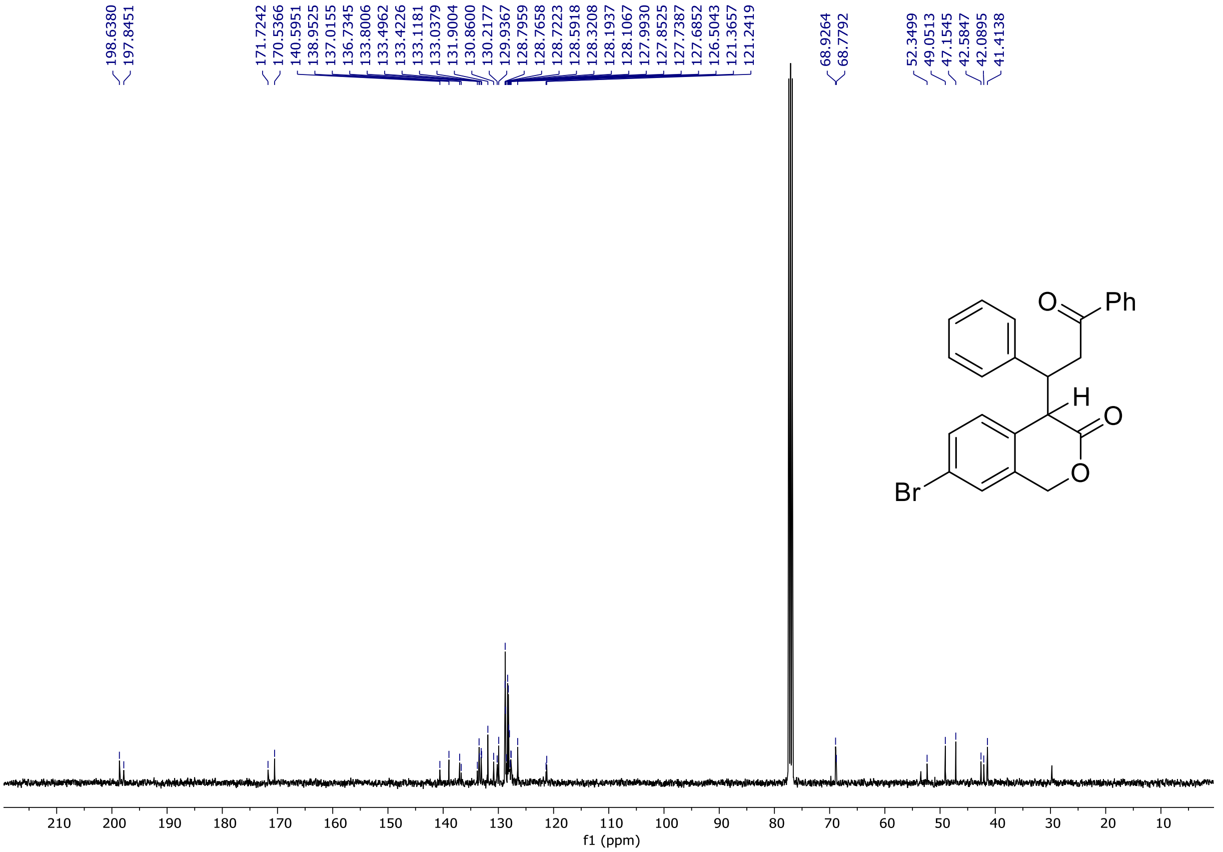

(R*)-6-methoxy-4-((R*)-3-oxo-1,3-diphenylpropyl)isochroman-3-one (14l-1)

^{1}H NMR (400 MHz, $CDCl_3$)

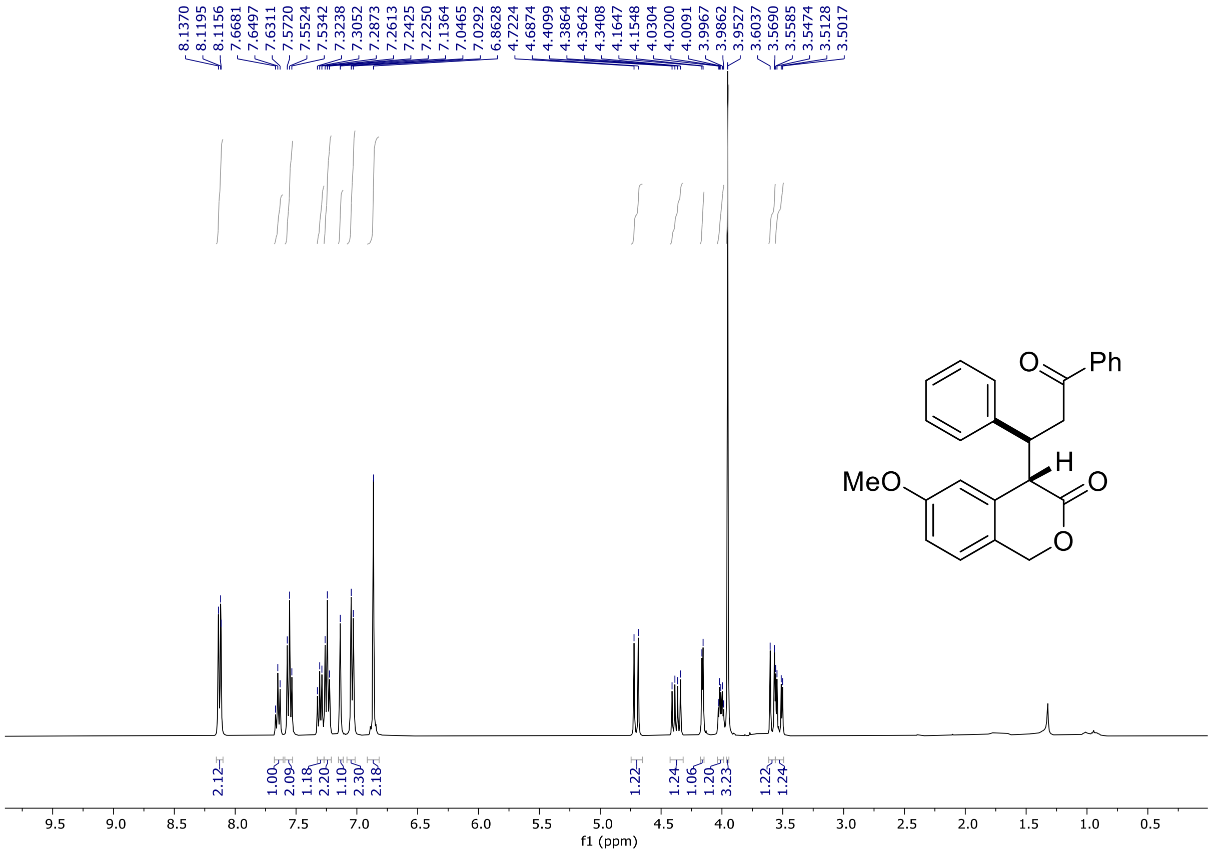

$^{13}C\{^{1}H\}$ NMR (100 MHz, $CDCl_3$)

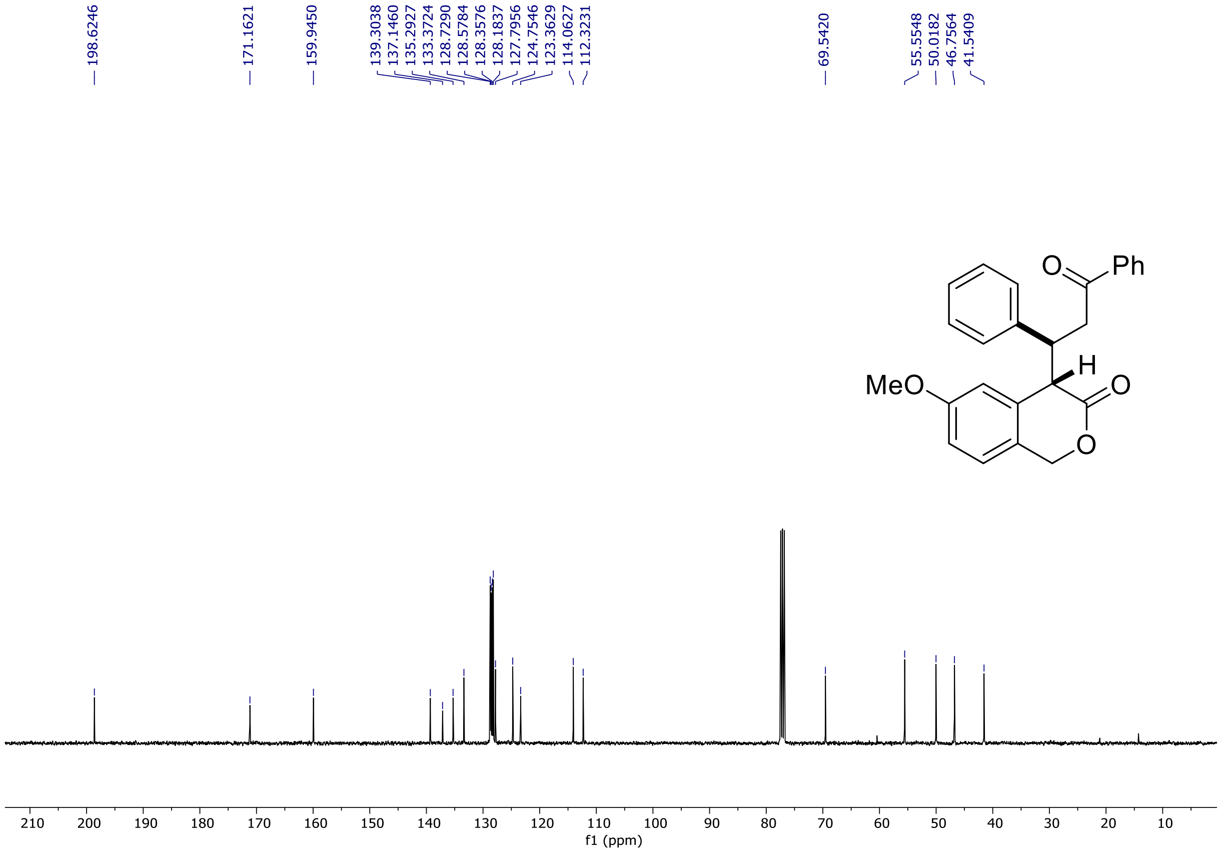

(R*)-6-methoxy-4-((S*)-3-oxo-1,3-diphenylpropyl)isochroman-3-one (14l-2)

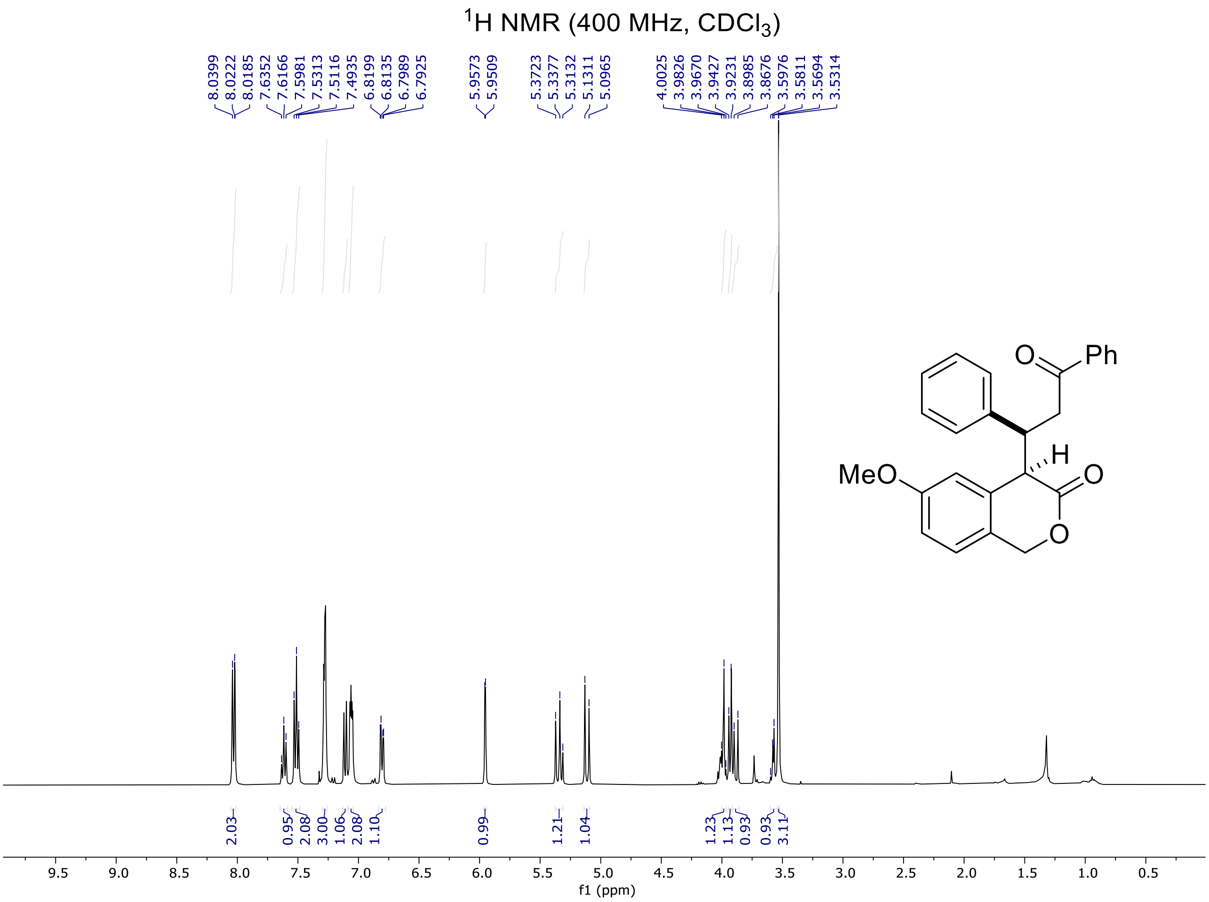

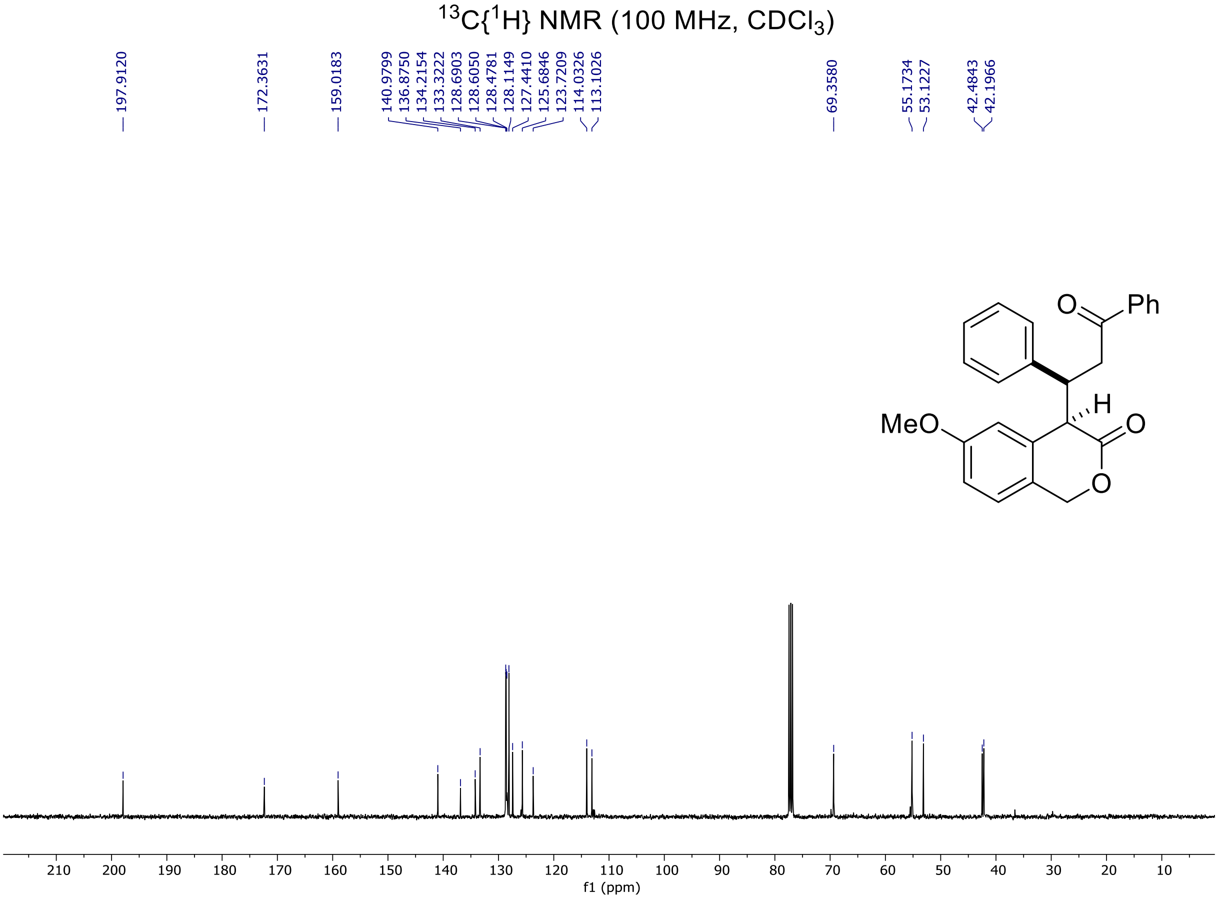

7-nitro-4-(3-oxo-1,3-diphenylpropyl)isochroman-3-one (14m)

^1H NMR (400 MHz, $CDCl_3$)

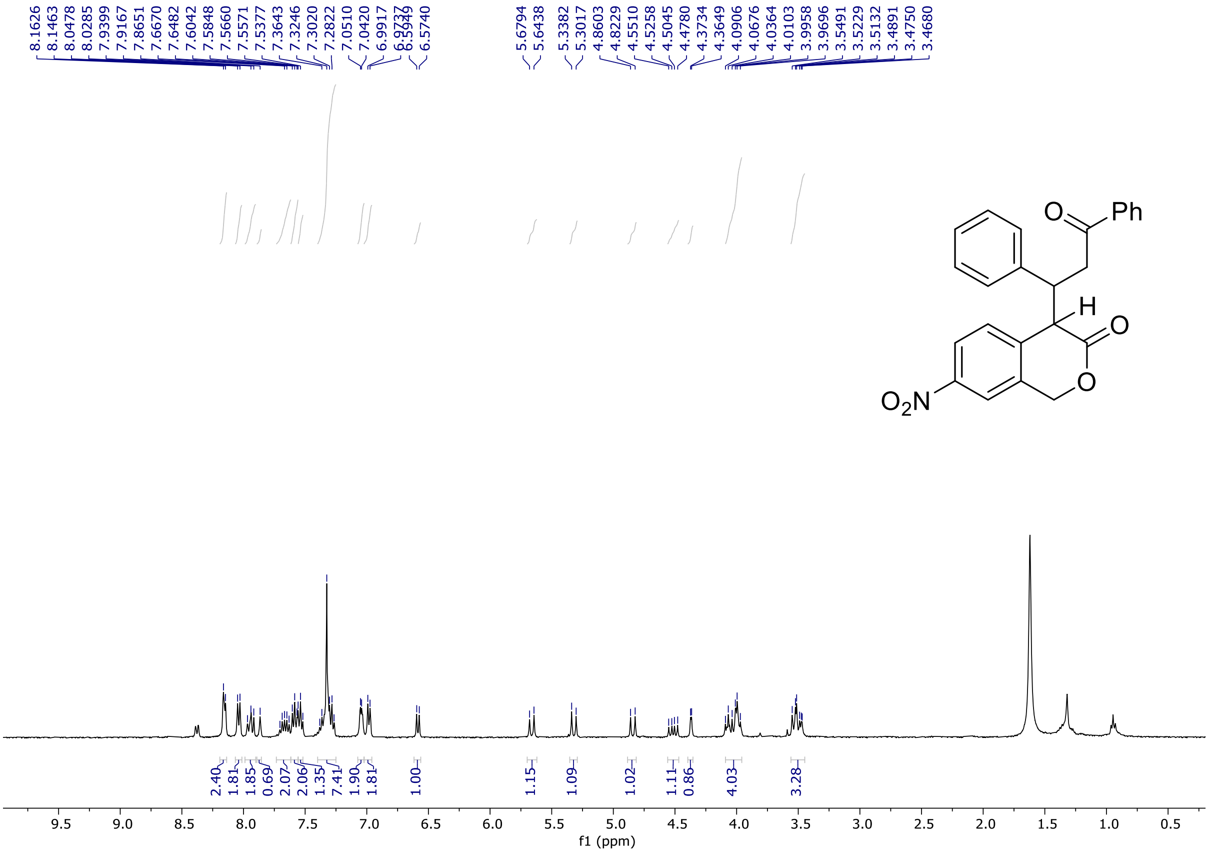

$^{13}C\{^1H\}$ NMR (100 MHz, $CDCl_3$)

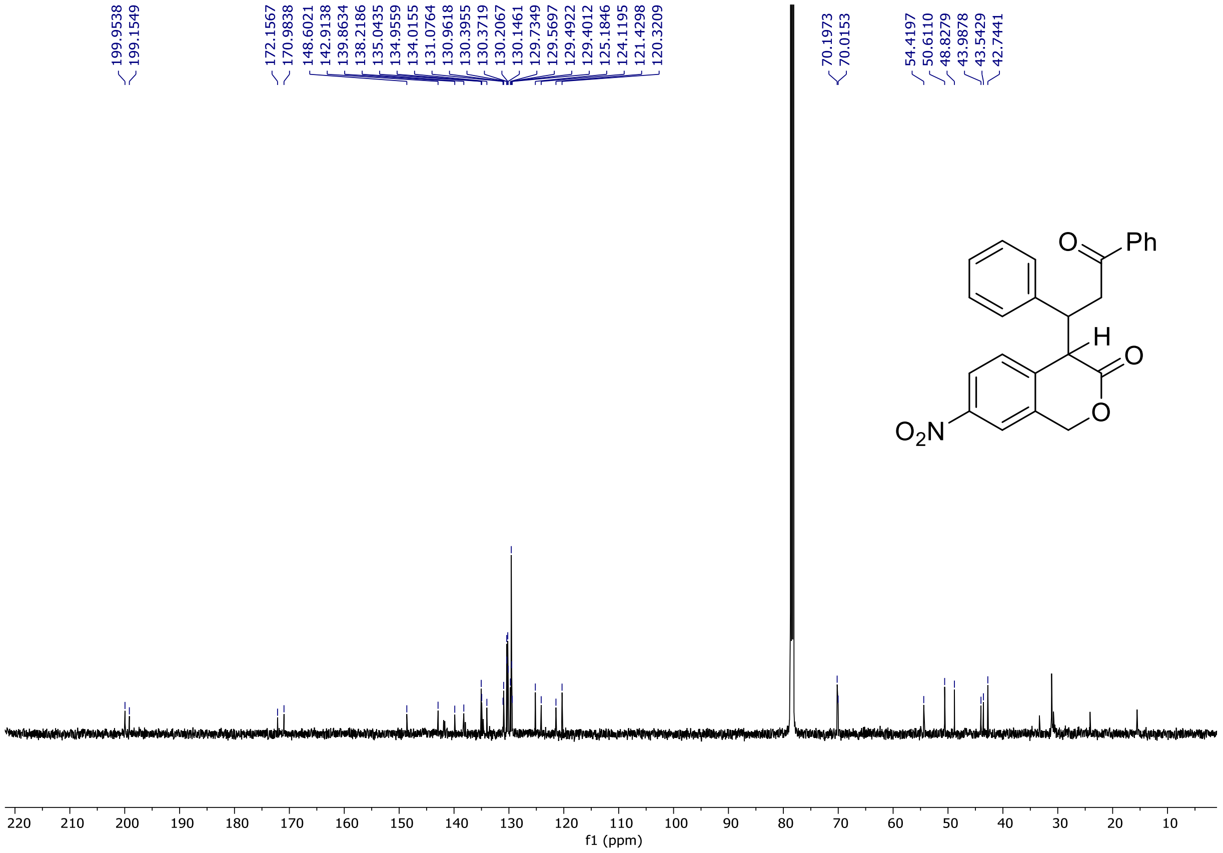

(R*)-6-chloro-4-((R*)-3-oxo-1,3-diphenylpropyl)isochroman-3-one (14n-1)

^{1}H NMR (400 MHz, $CDCl_3$)

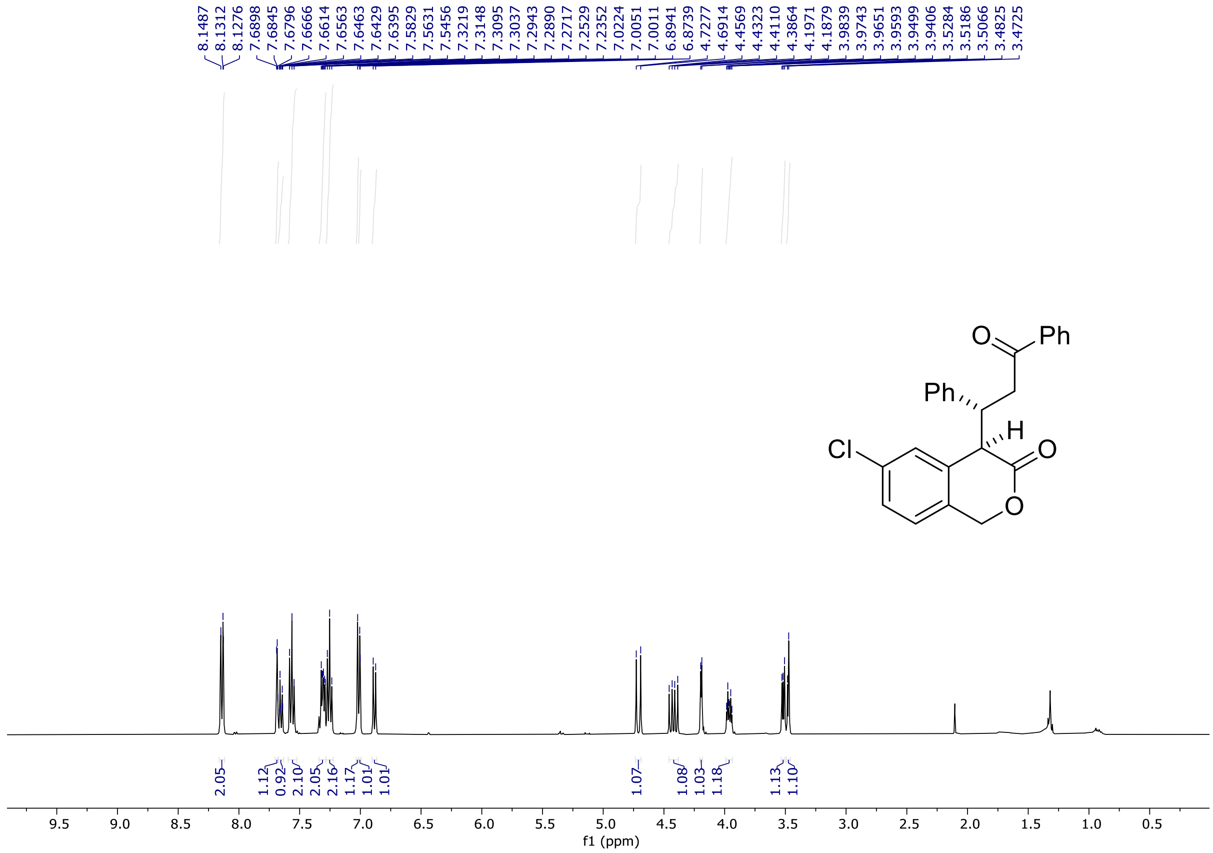

^{13}C{^{1}H} NMR (100 MHz, $CDCl_3$)

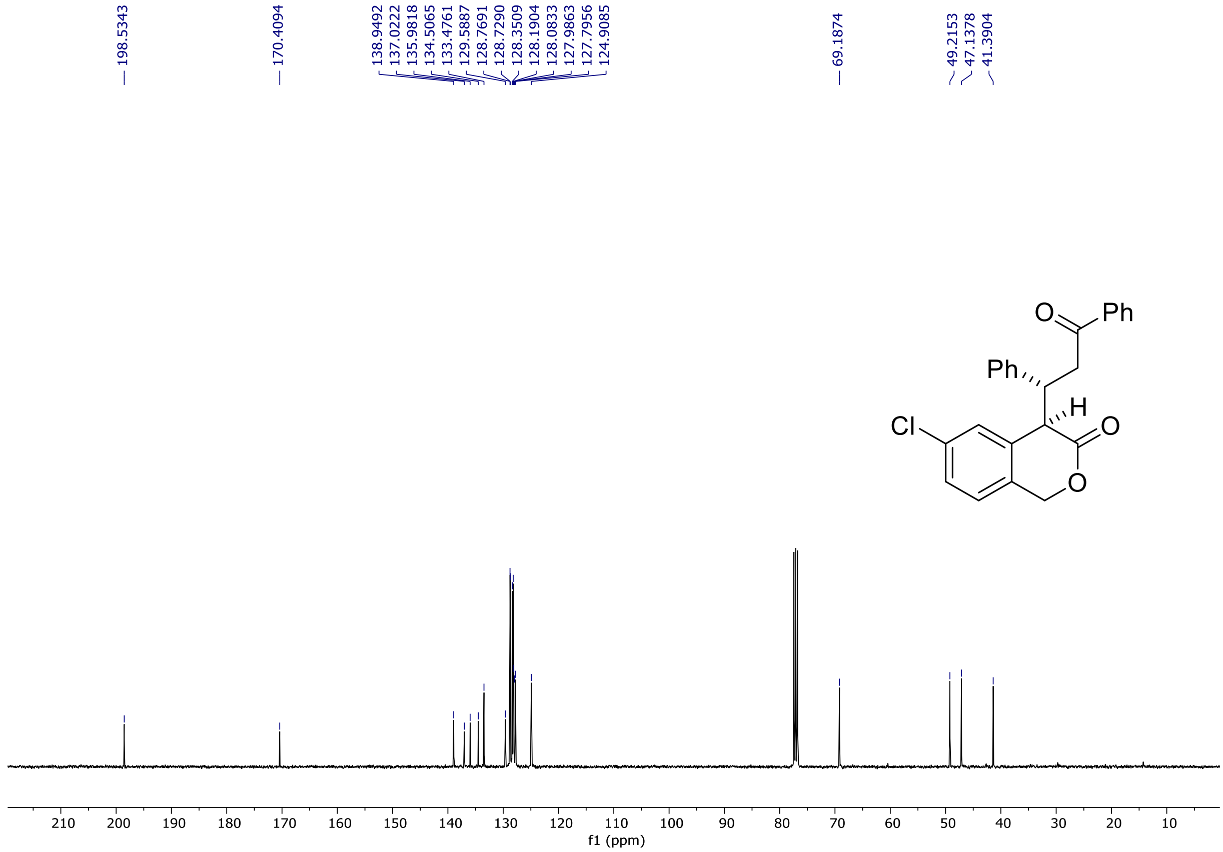

(R*)-6-chloro-4-((S*)-3-oxo-1,3-diphenylpropyl)isochroman-3-one (14n-2)

^{1}H NMR (400 MHz, $CDCl_3$)

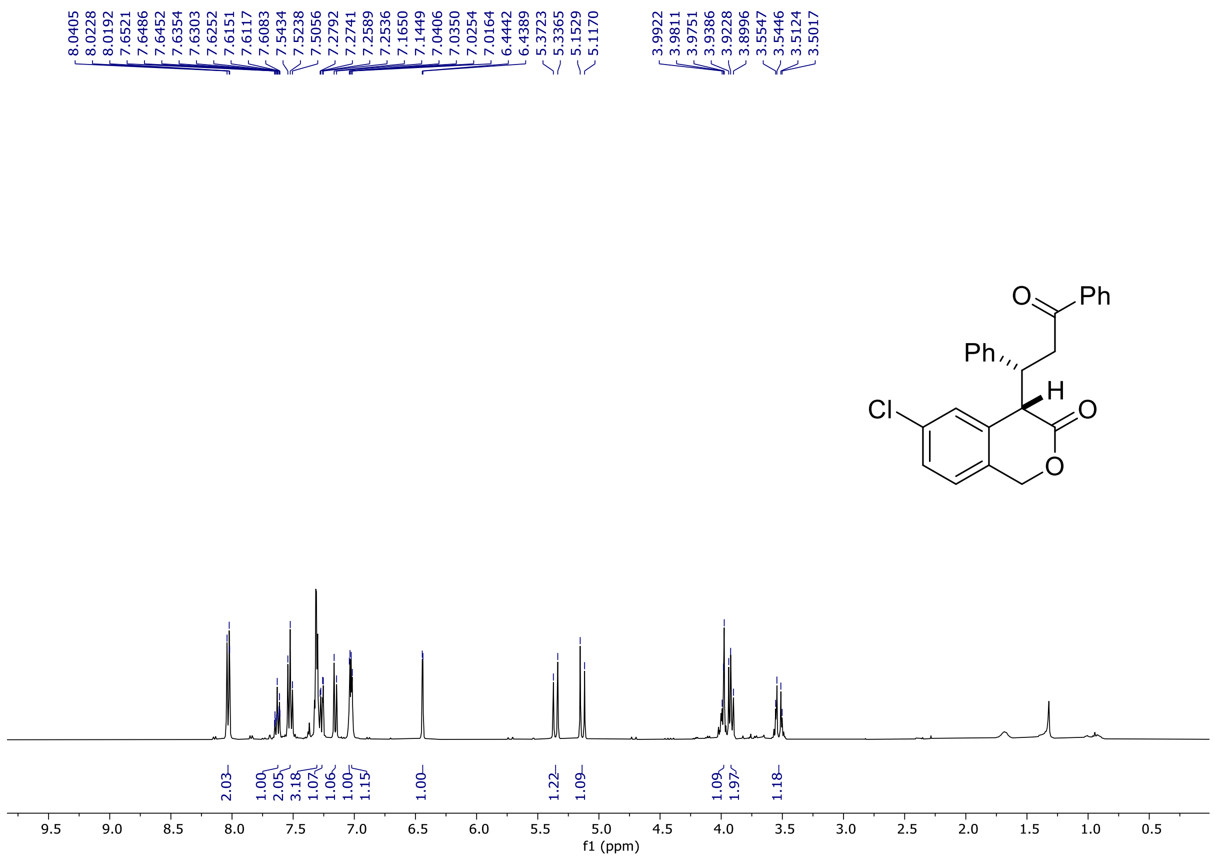

$^{13}C\{^{1}H\}$ NMR (75 MHz, $CDCl_3$)

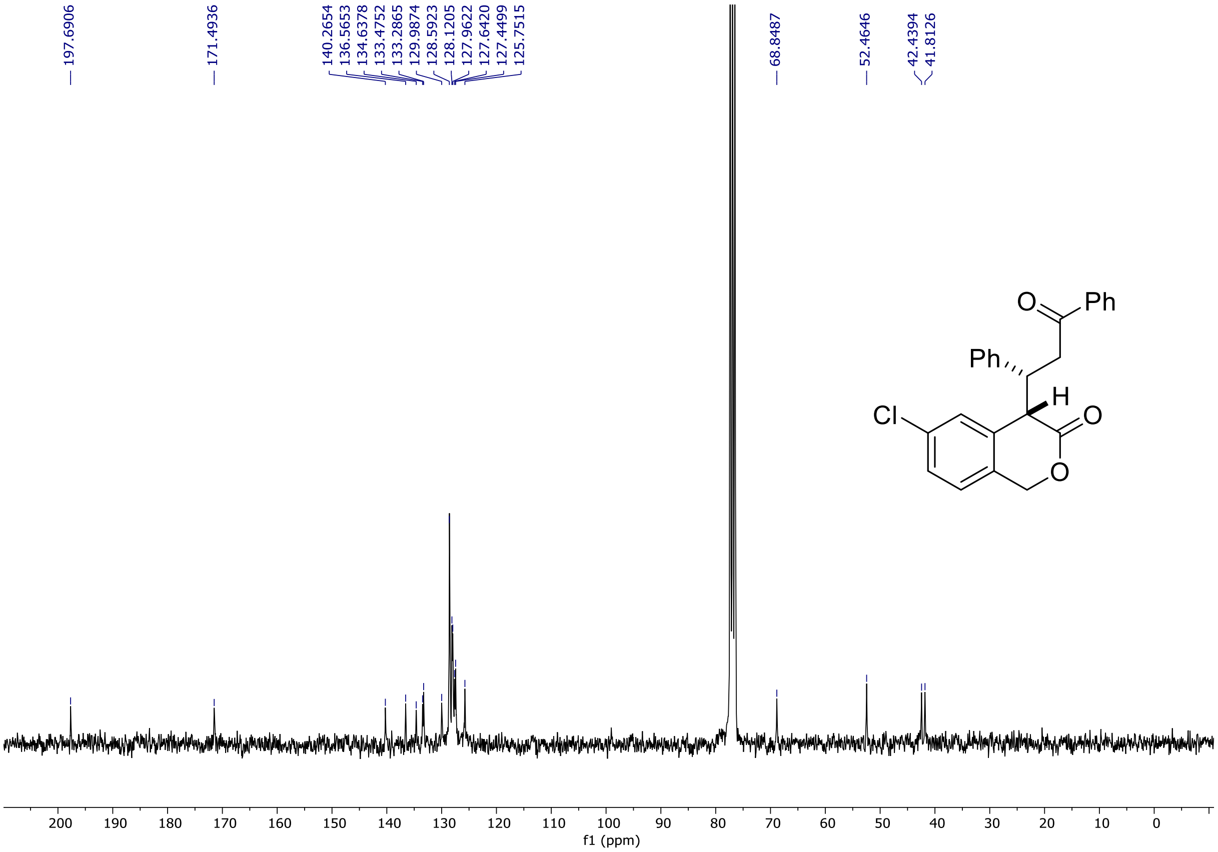

6-methoxy-4-(1-(4-methoxyphenyl)-3-oxo-3-phenylpropyl)isochroman-3-one (14o):

^{1}H NMR (400 MHz, $CDCl_3$)

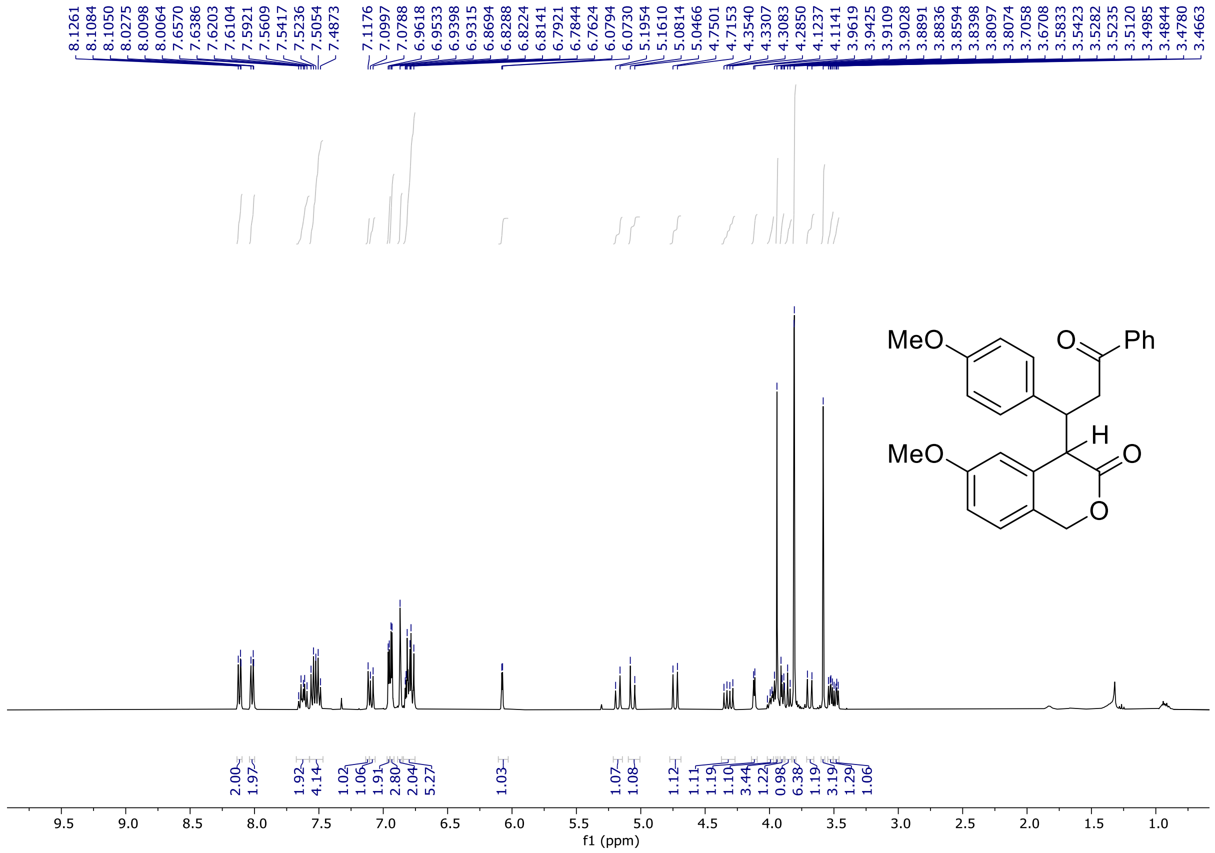

$^{13}C\{^{1}H\}$ NMR (75 MHz, $CDCl_3$)

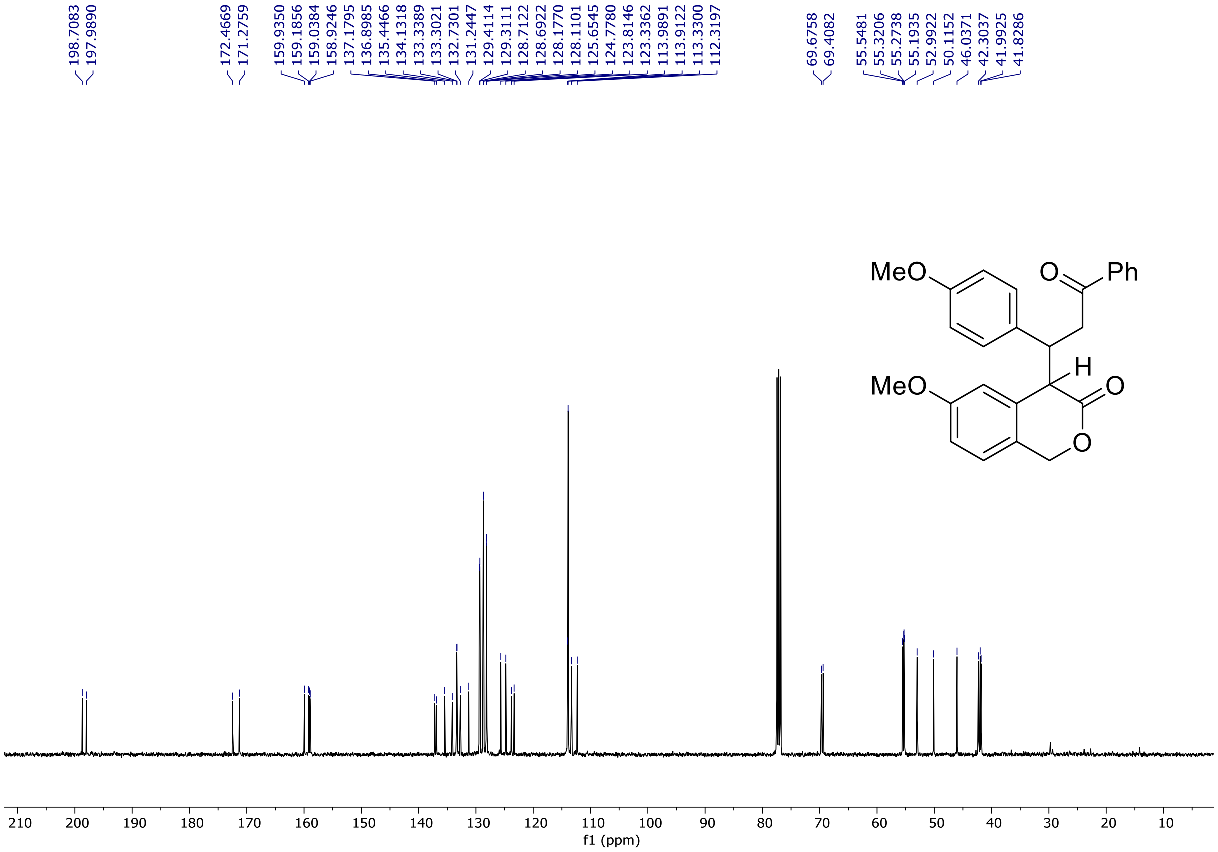

(R*)-8-fluoro-4-((R*)-3-oxo-1,3-diphenylpropyl)isochroman-3-one (14p-1)

^{1}H NMR (400 MHz, $CDCl_3$)

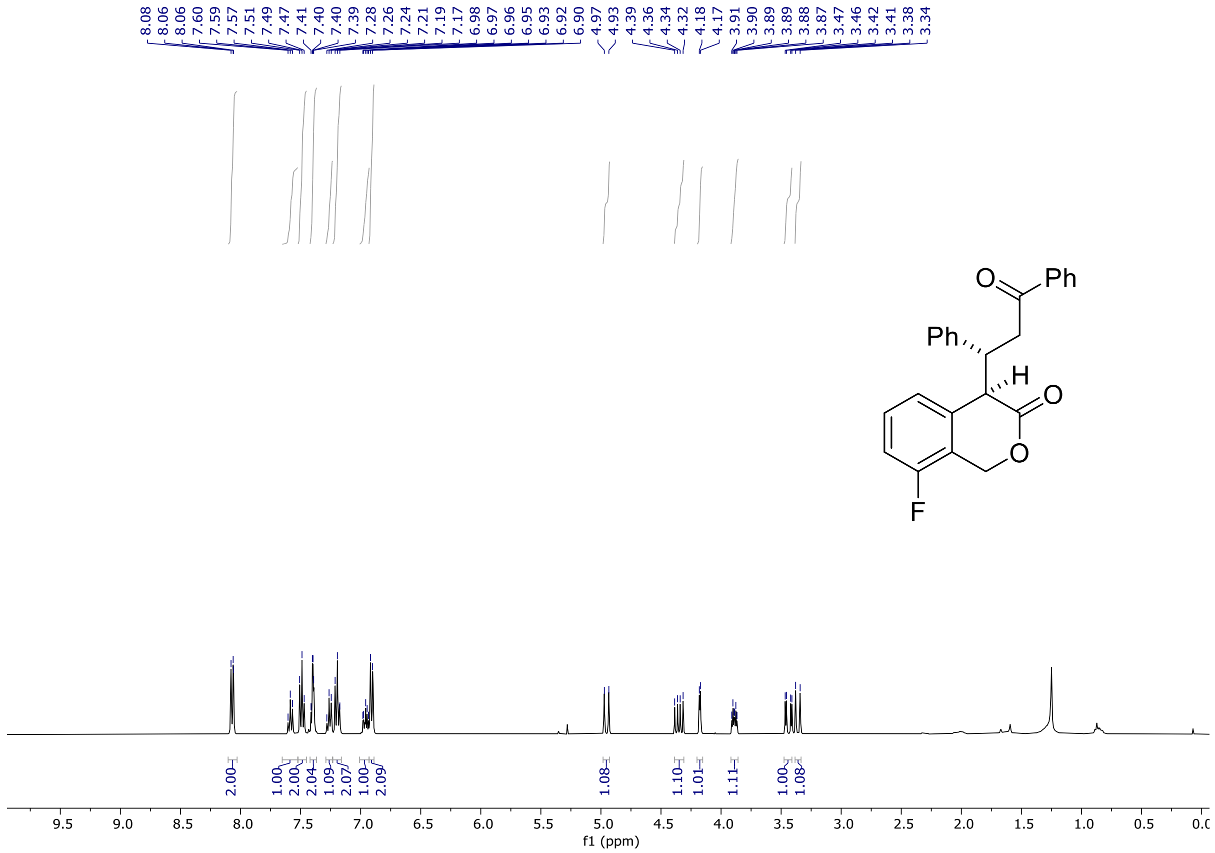

^{13}C{^{1}H} NMR (63 MHz, $CDCl_3$)

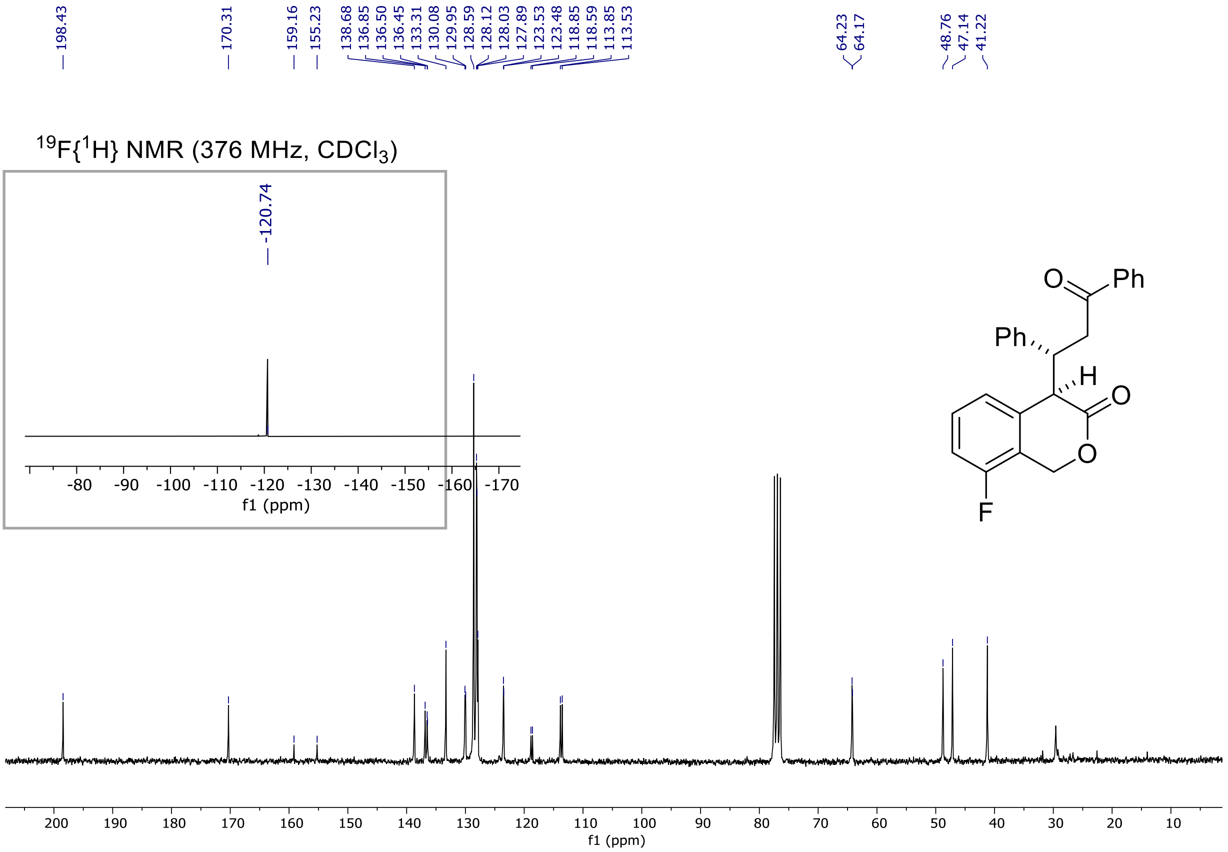

(R*)-8-fluoro-4-((S*)-3-oxo-1,3-diphenylpropyl)isochroman-3-one (14p-2)

^{1}H NMR (300 MHz, $CDCl_3$)

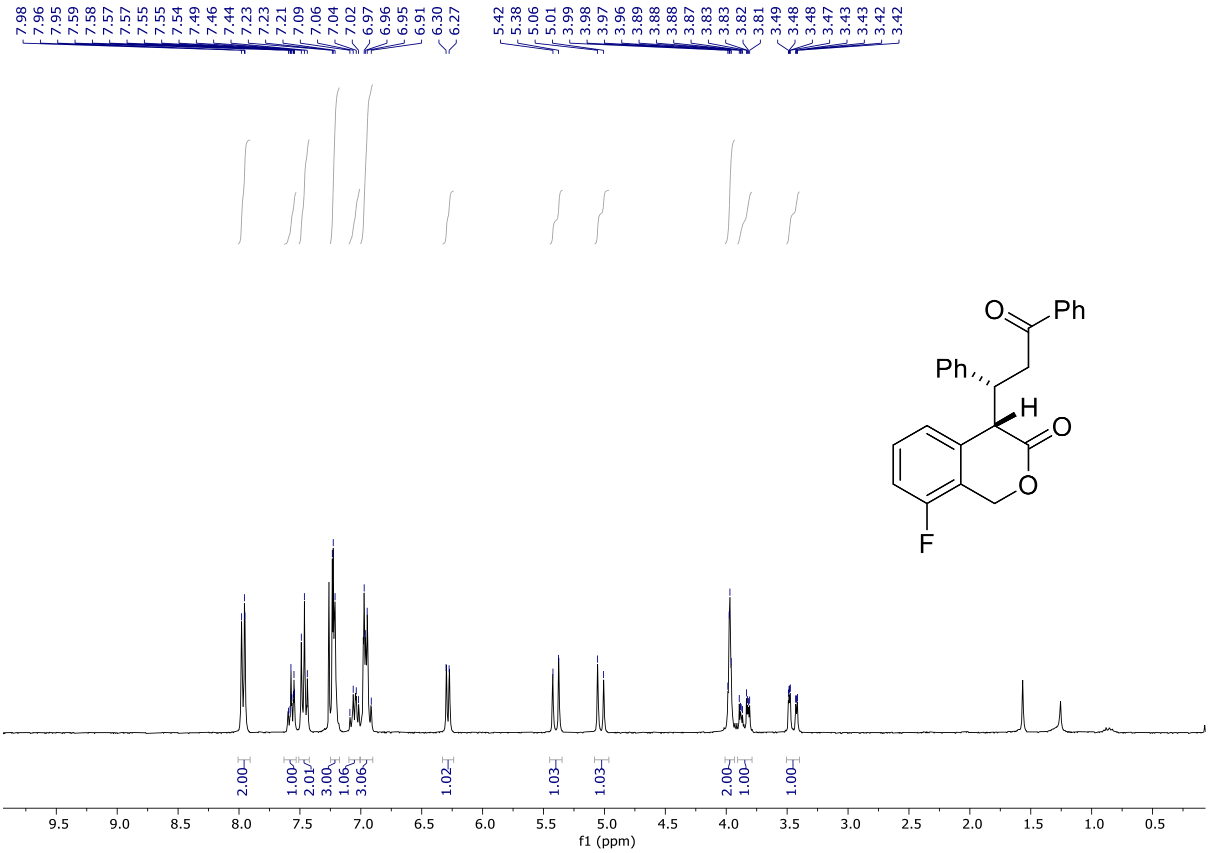

$^{13}C\{^{1}H\}$ NMR (63 MHz, $CDCl_3$)

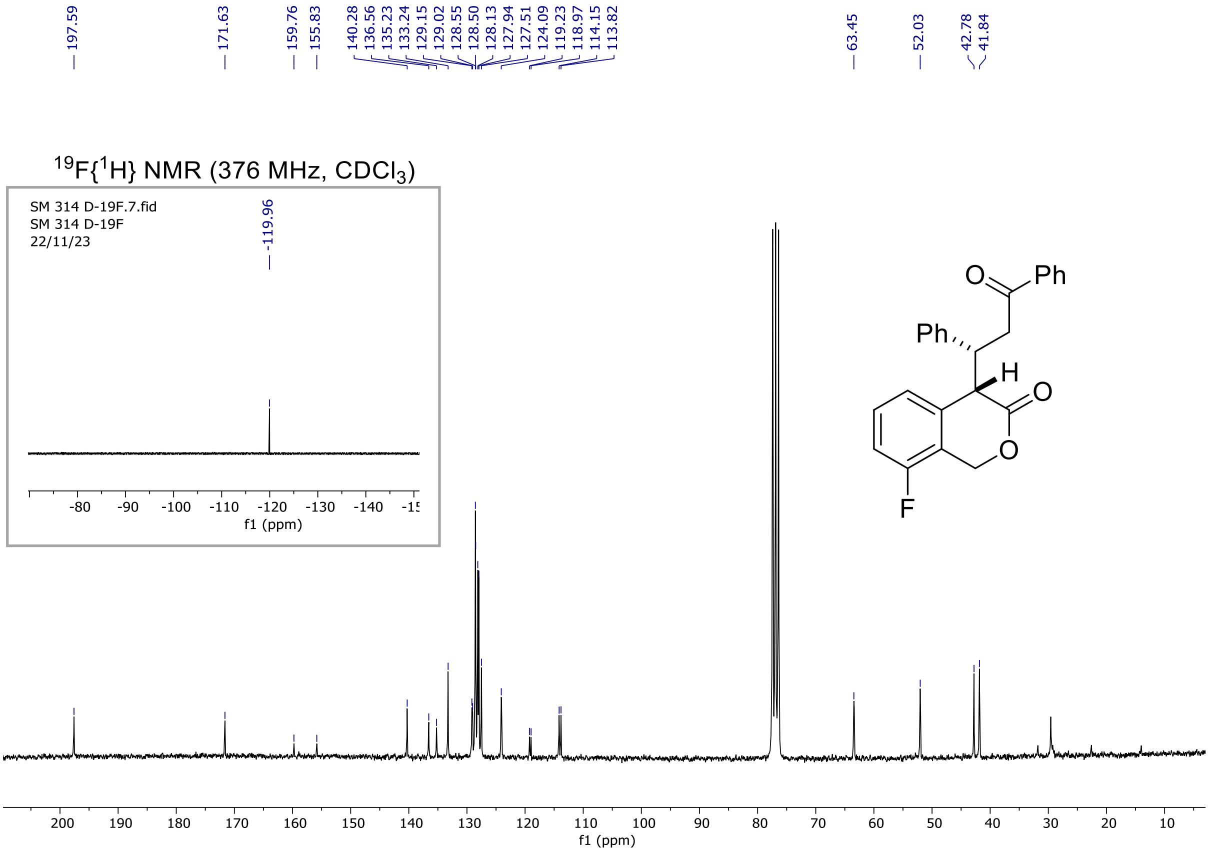

3-(3-oxo-1,3-diphenylpropyl)benzofuran-2(3H)-one (15a)

^{1}H NMR (400 MHz, $CDCl_3$)

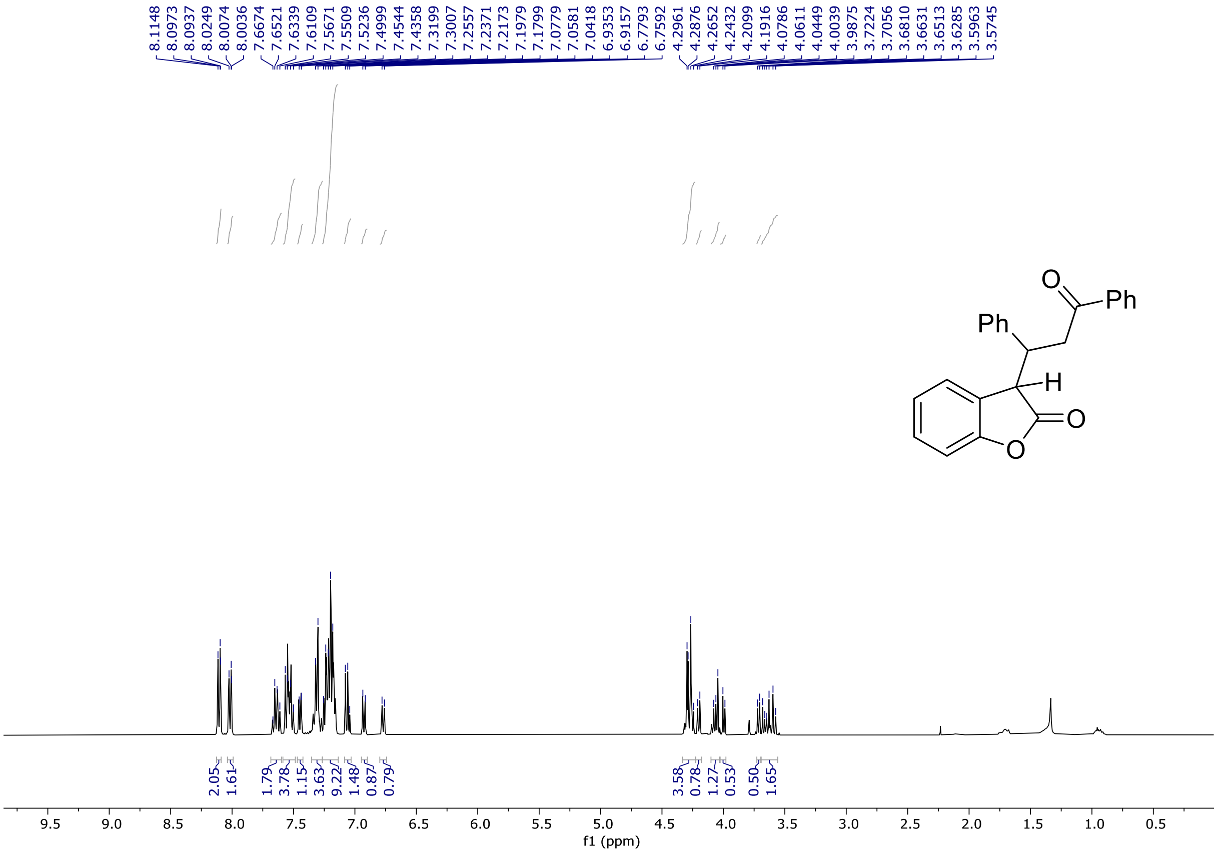

^{13}C{^{1}H} NMR (75 MHz, $CDCl_3$)

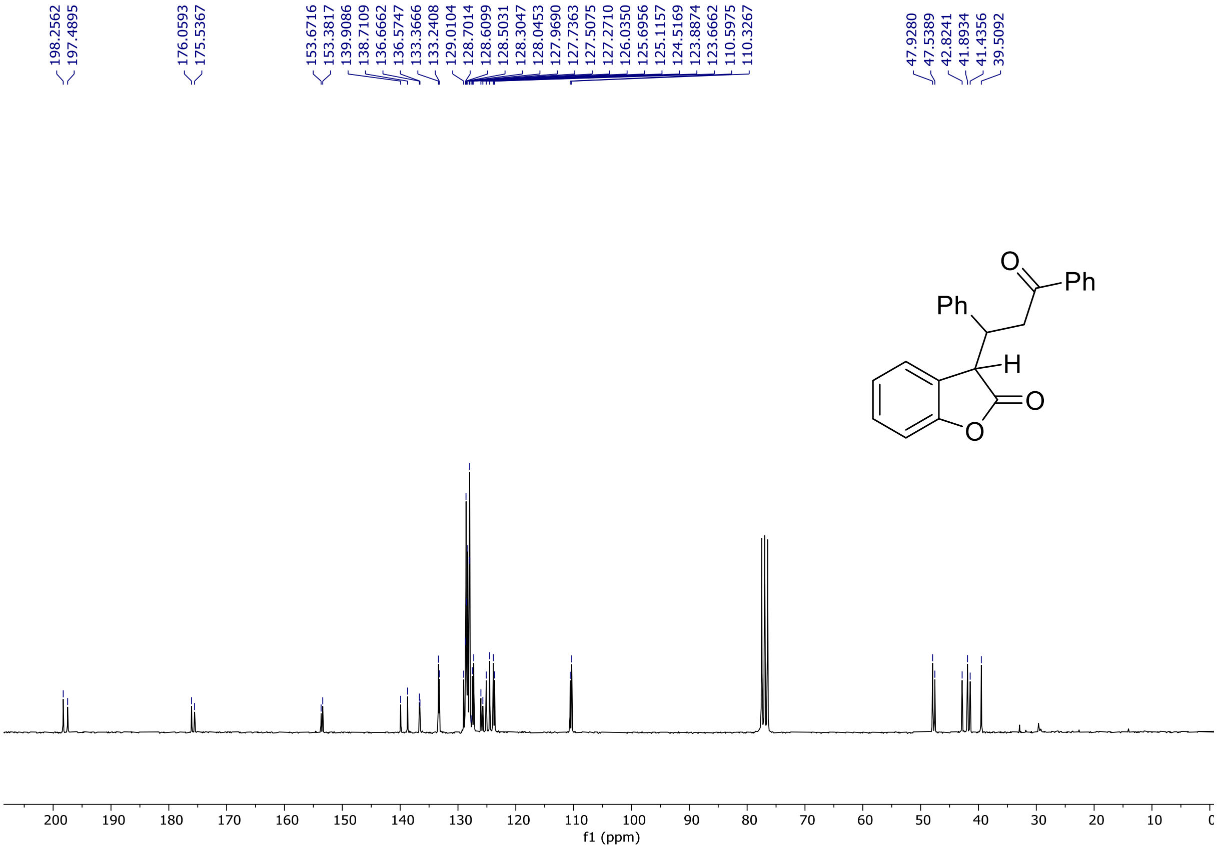

3-(1-(4-chlorophenyl)-2-oxo-2-phenylethyl)benzofuran-2(3H)-one (15b)

^{1}H NMR (400 MHz, $CDCl_3$)

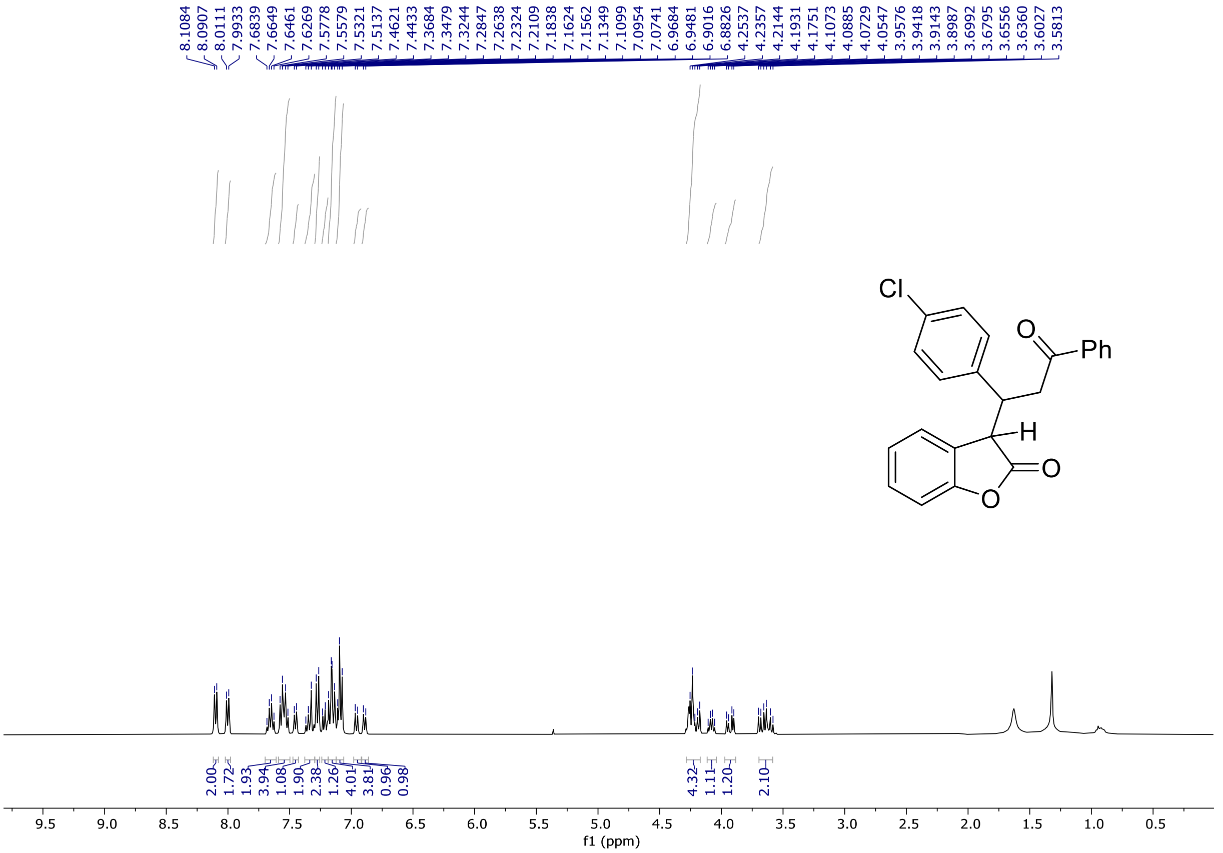

^{13}C{^{1}H} NMR (75 MHz, $CDCl_3$)

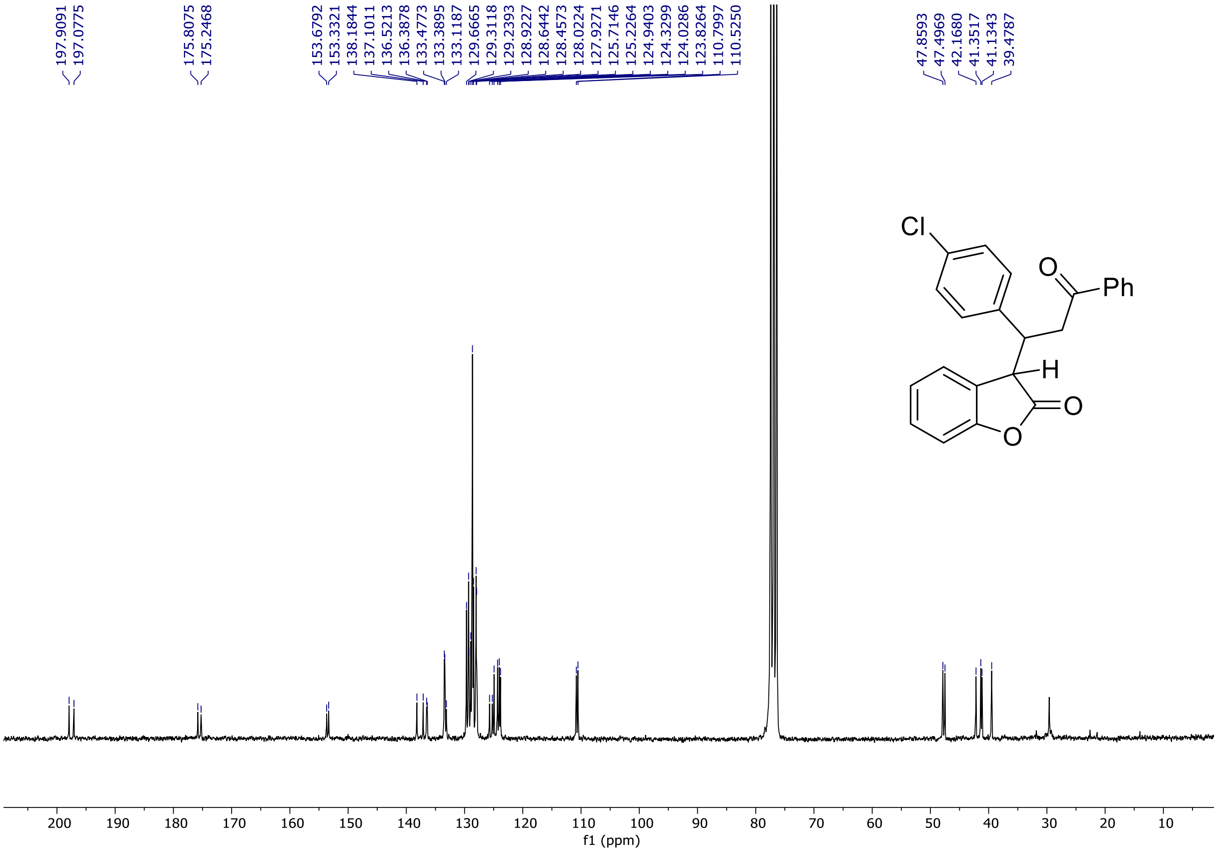

3-(1-(4-methoxyphenyl)-2-oxo-2-phenylethyl)benzofuran-2(3H)-one (15c)

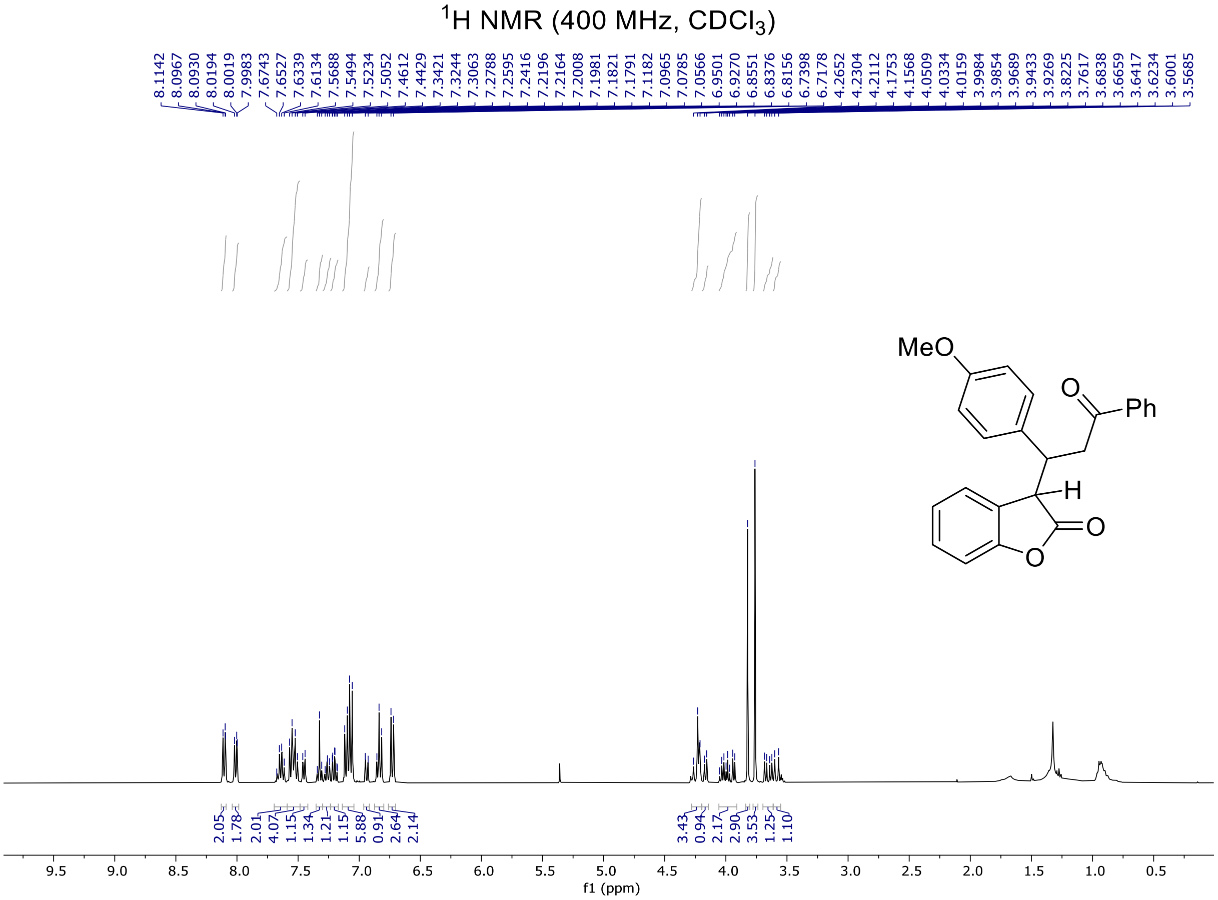

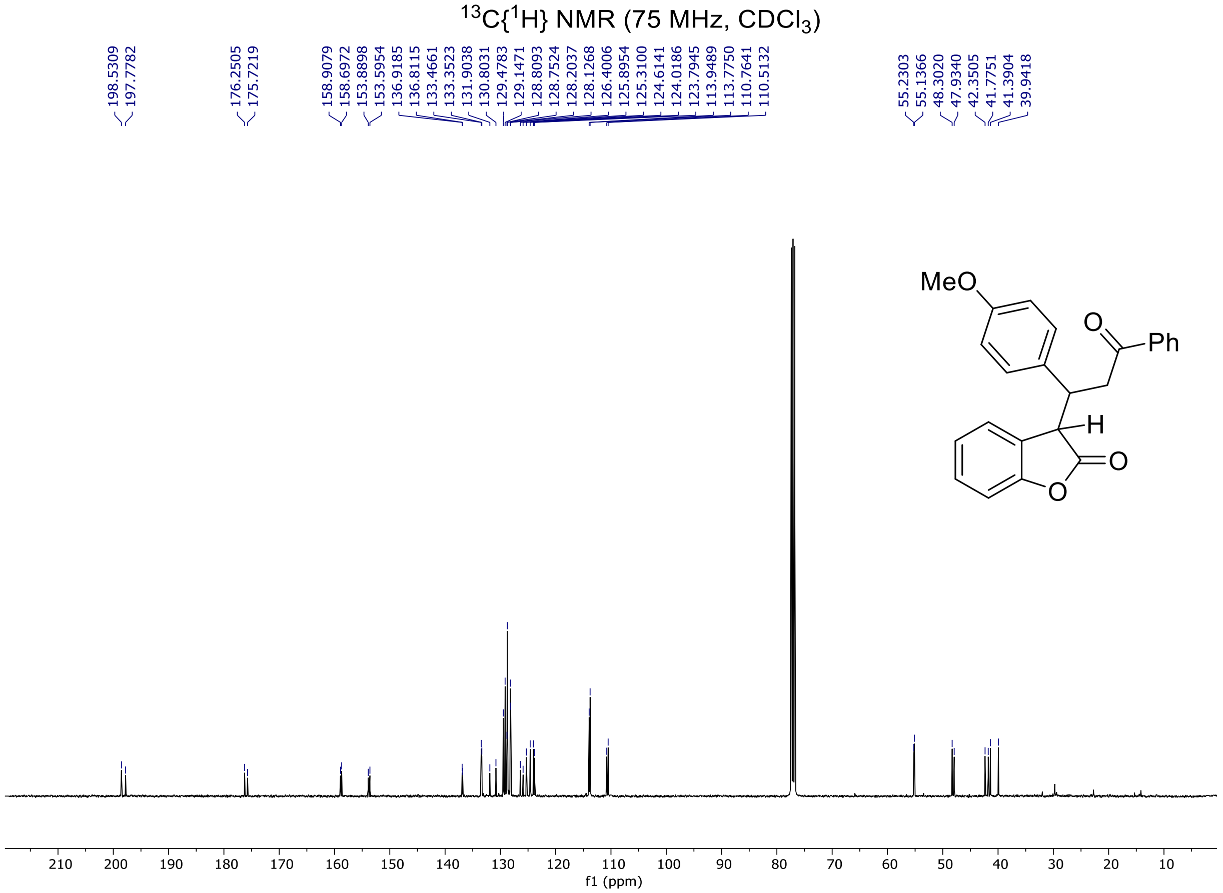

2-oxo-3-(2-oxo-1,2-diphenylethyl)-2,3-dihydrobenzofuran-5-yl acetate (mixture) (15d)

^{1}H NMR (400 MHz, CDCl$_3$)

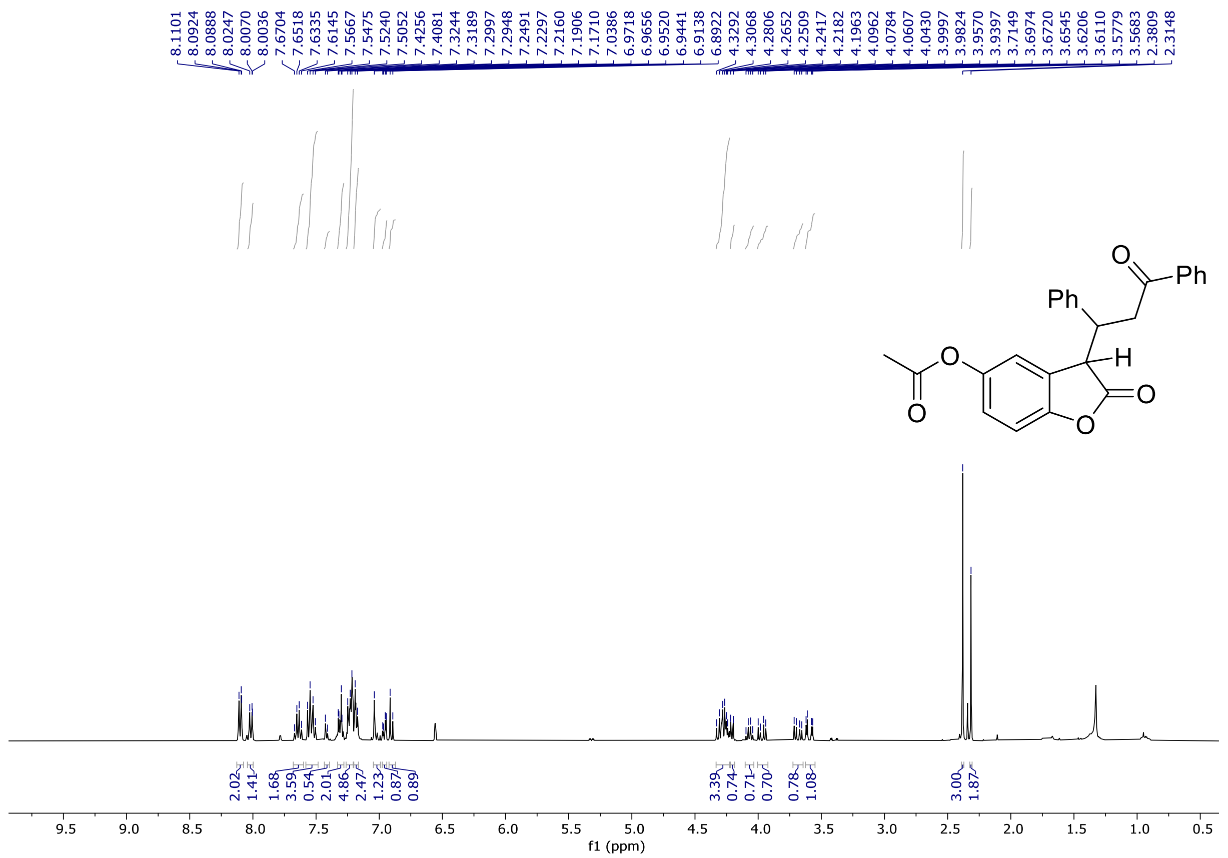

^{13}C{^{1}H} NMR (75 MHz, CDCl$_3$)

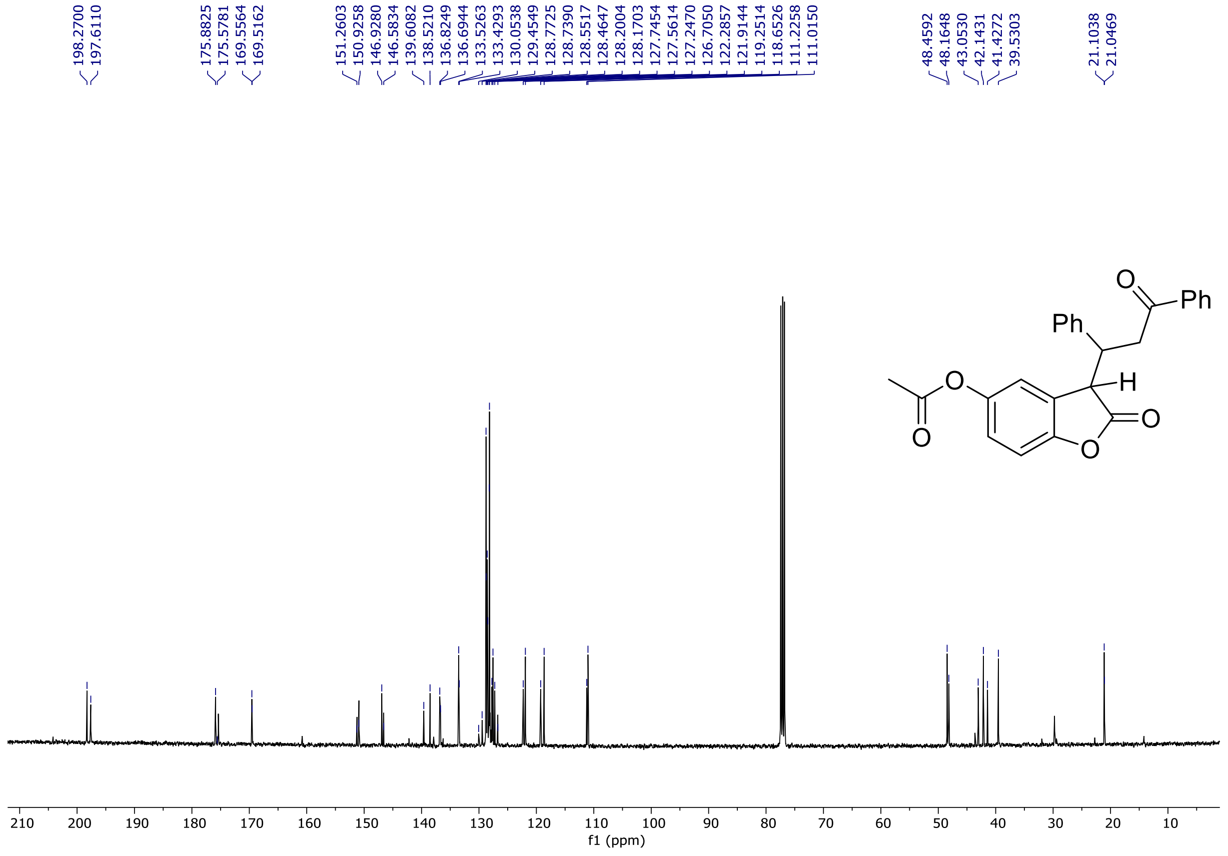

(R*)-2-oxo-3-((S*)-3-oxo-1,3-diphenylpropyl)-2,3-dihydrobenzofuran-5-yl acetate (15d-1)

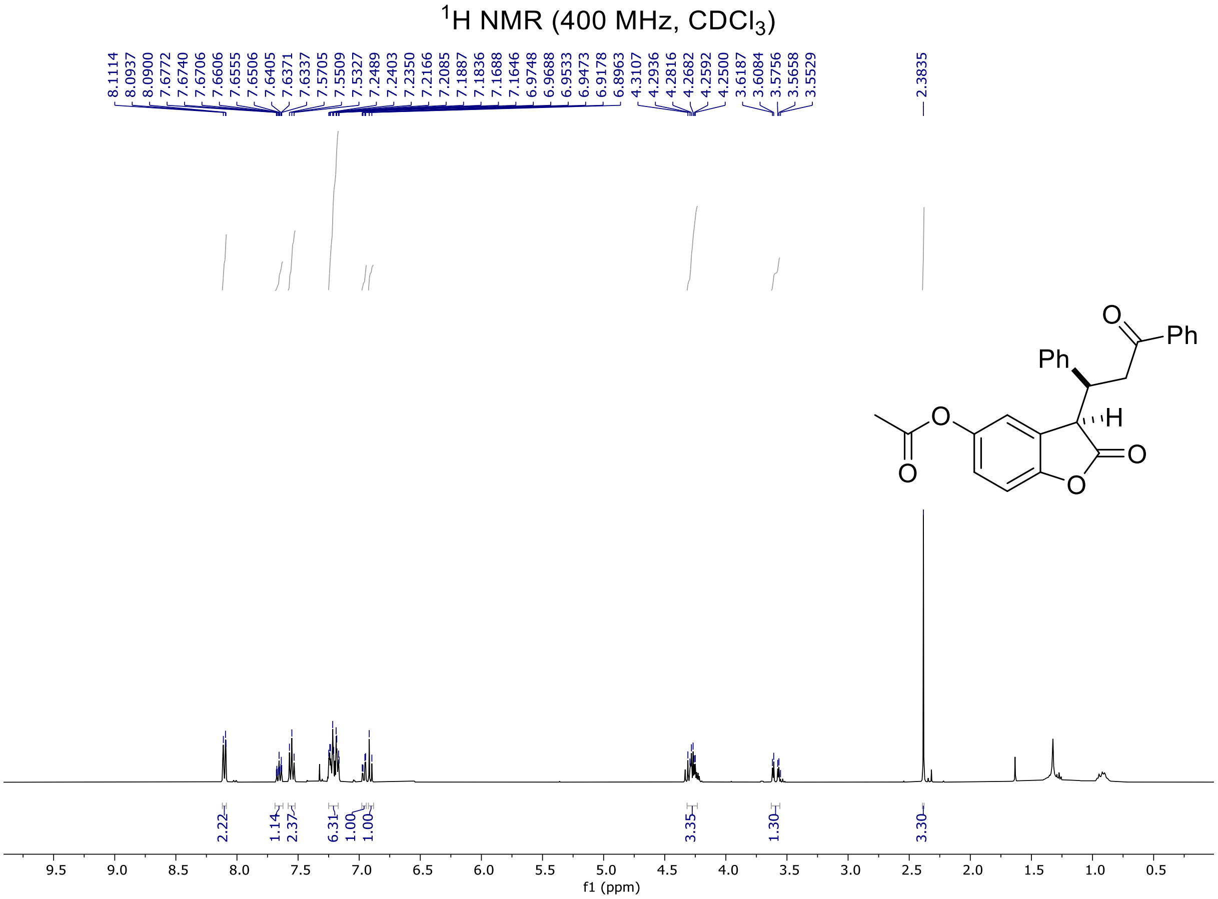

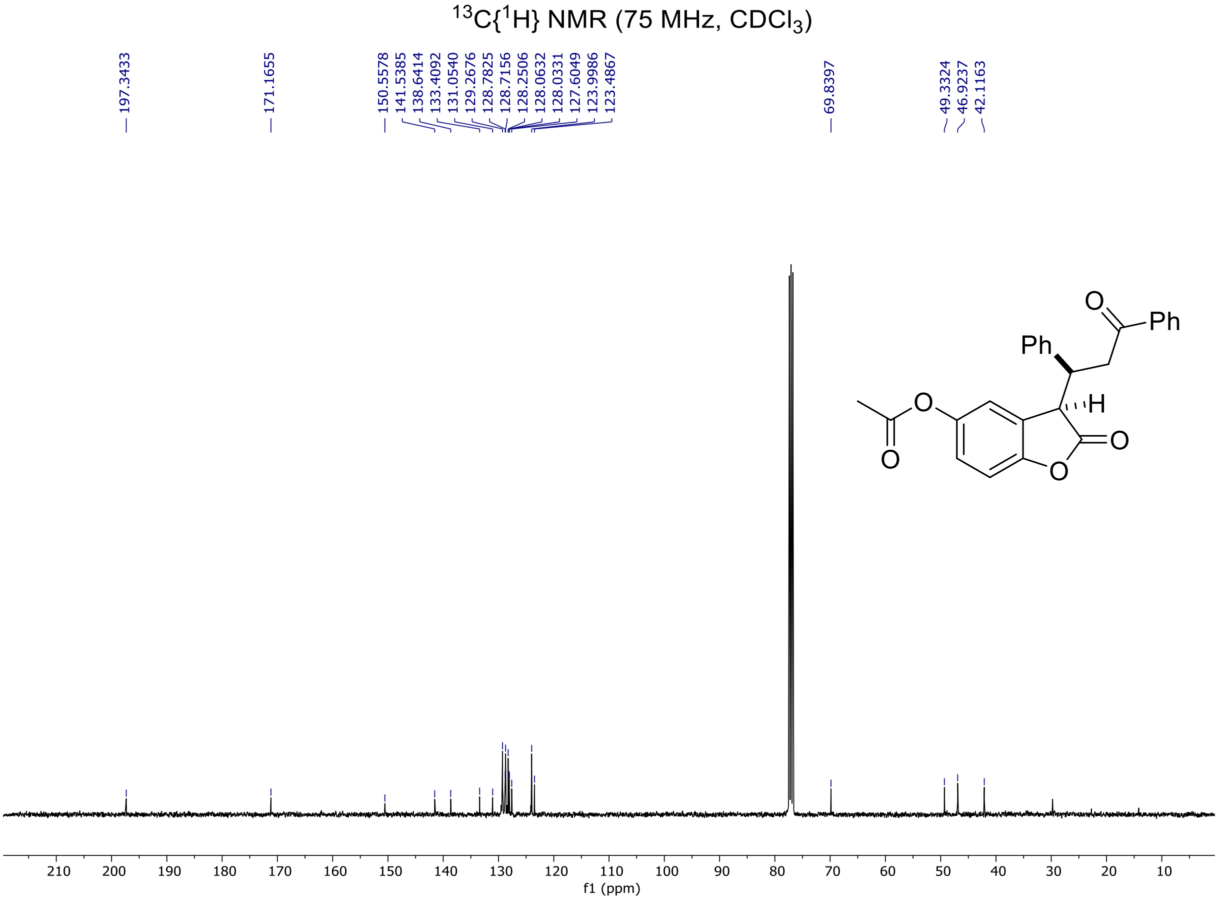

3-(3-(4-methoxyphenyl)-3-oxo-1-phenylpropyl)benzofuran-2(3H)-one (15e)

^{1}H NMR (400 MHz, CDCl$_3$)

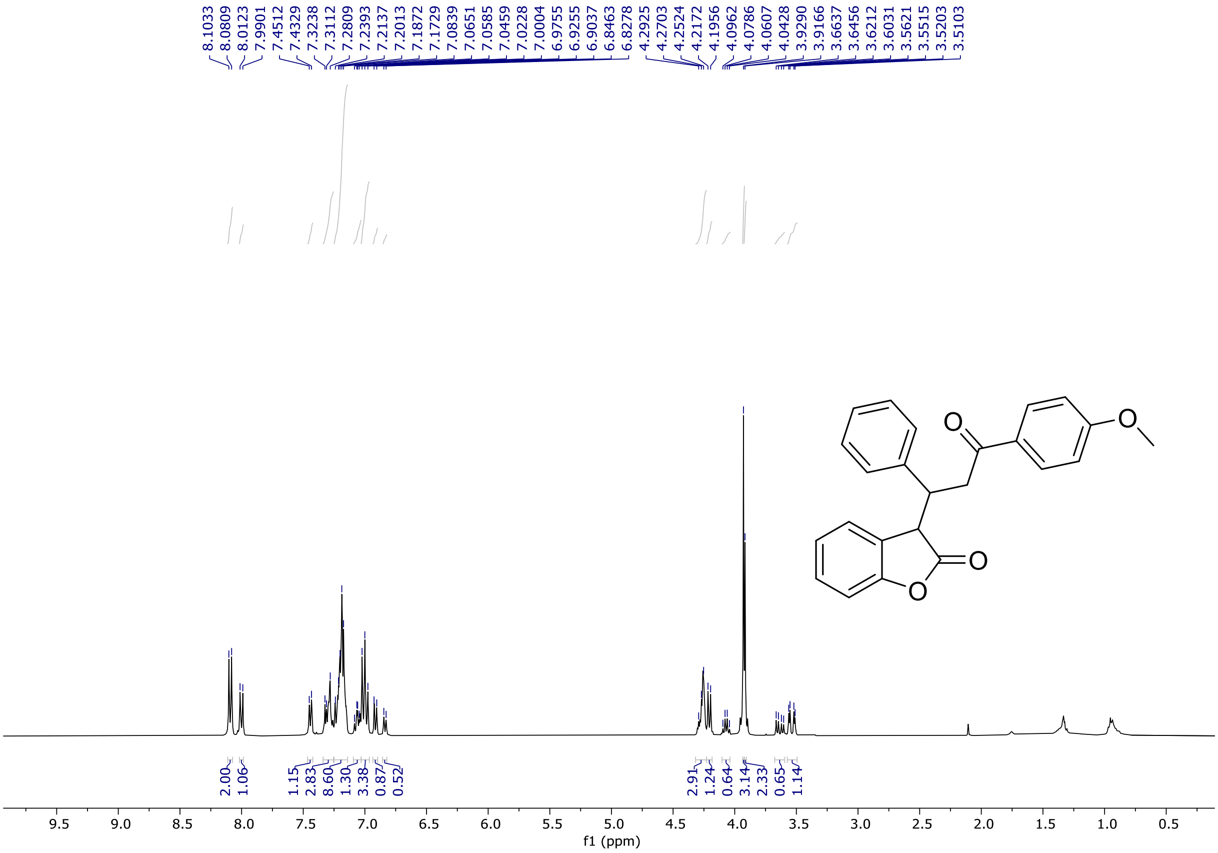

^{13}C{^{1}H} NMR (75 MHz, CDCl$_3$)

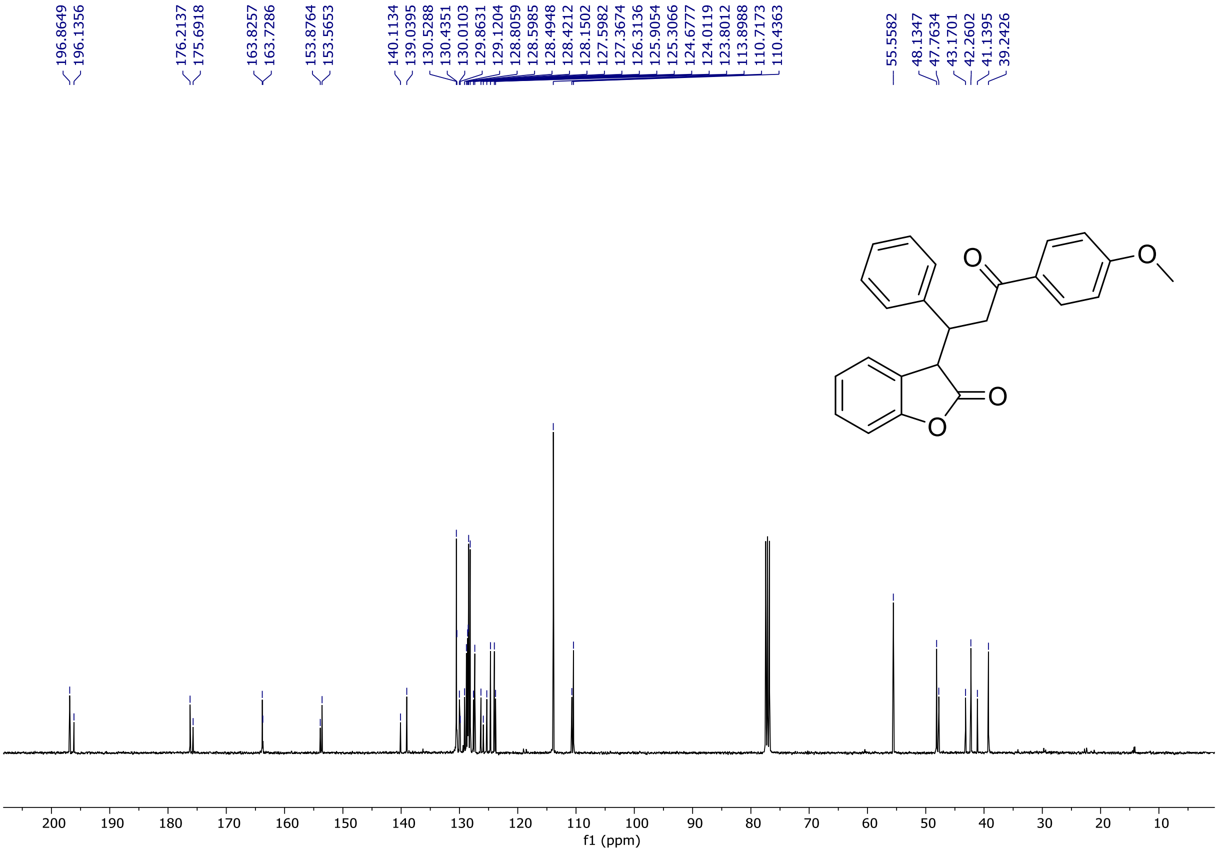

3-(1-(2-chlorophenyl)-3-oxo-3-phenylpropyl)benzofuran-2(3H)-one (15f)

^{1}H NMR (400 MHz, $CDCl_3$)

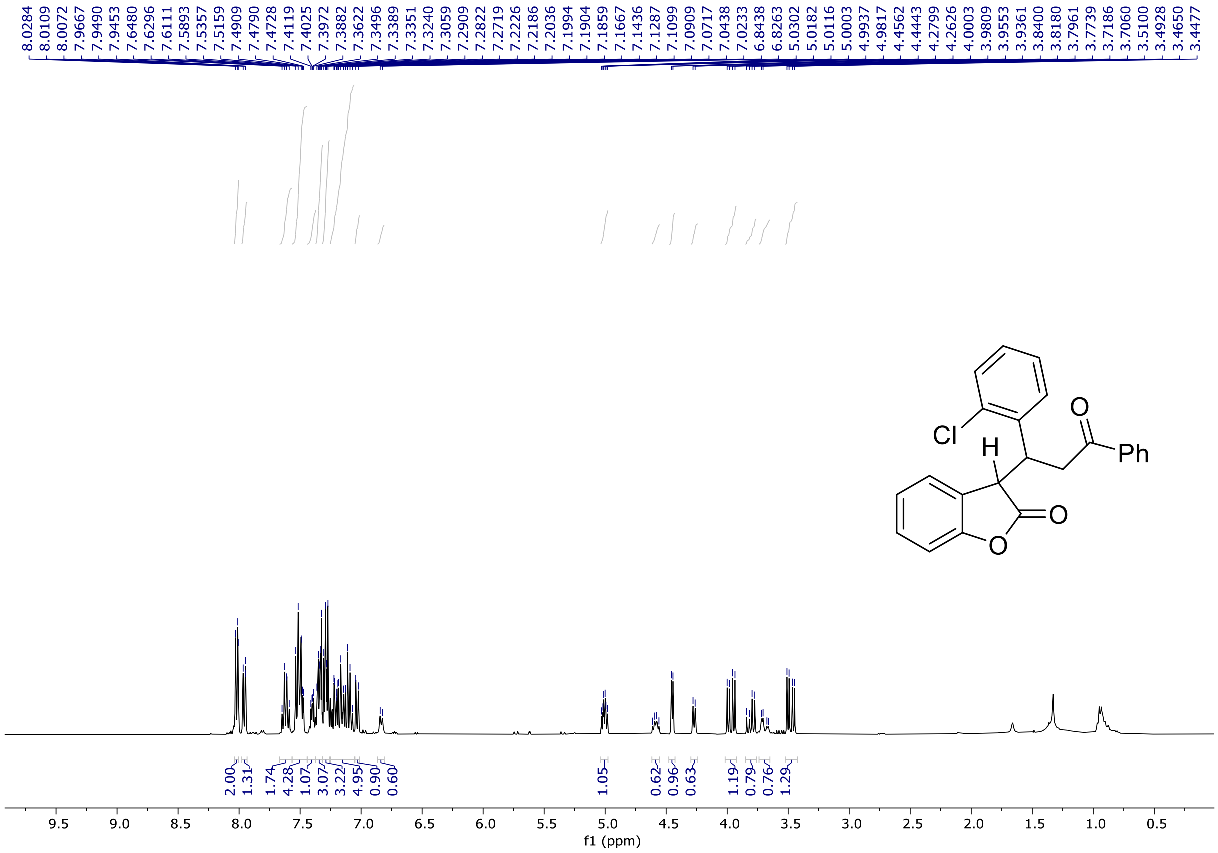

^{13}C{^{1}H} NMR (75 MHz, $CDCl_3$)

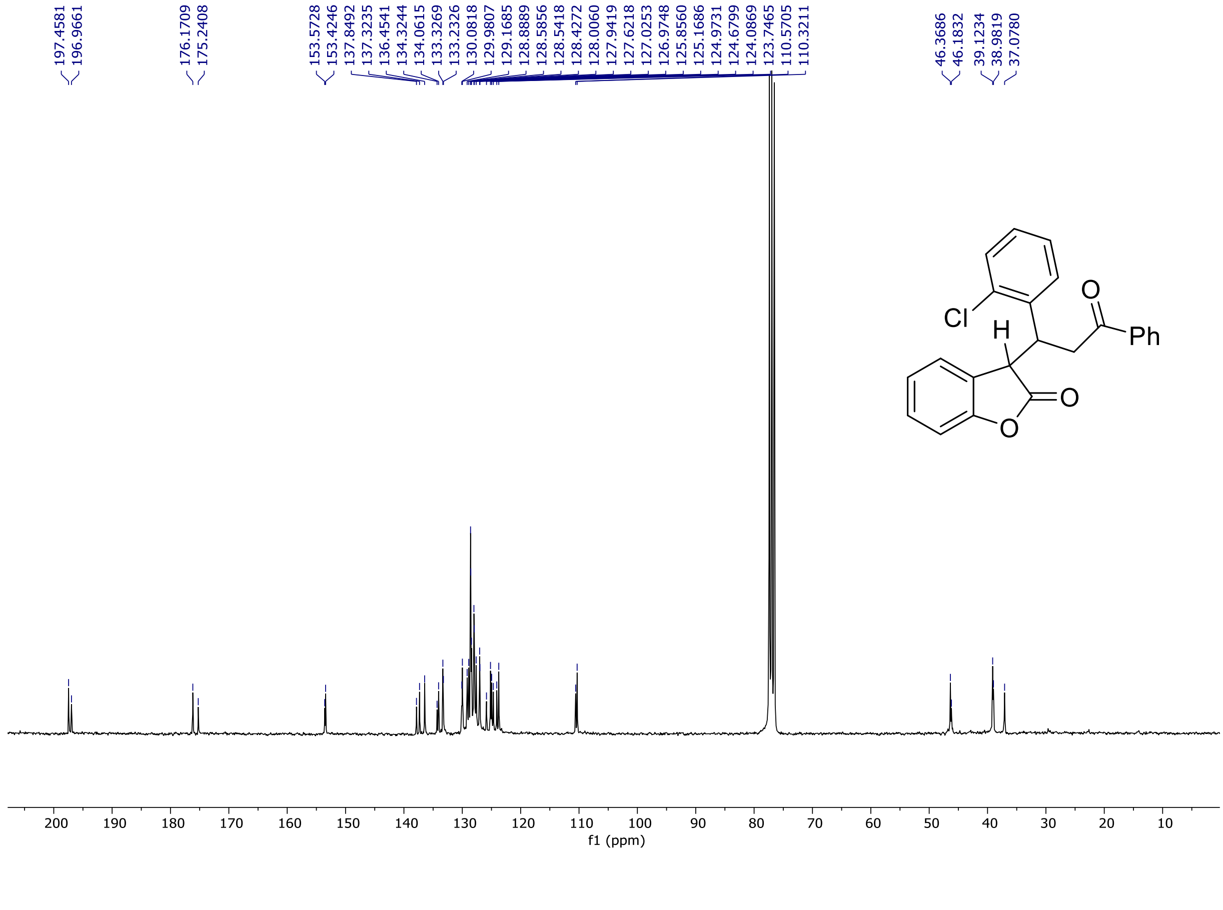

3-(1-(4-fluorophenyl)-3-oxo-3-phenylpropyl)benzofuran-2(3H)-one (15g)

^{1}H NMR (400 MHz, $CDCl_3$)

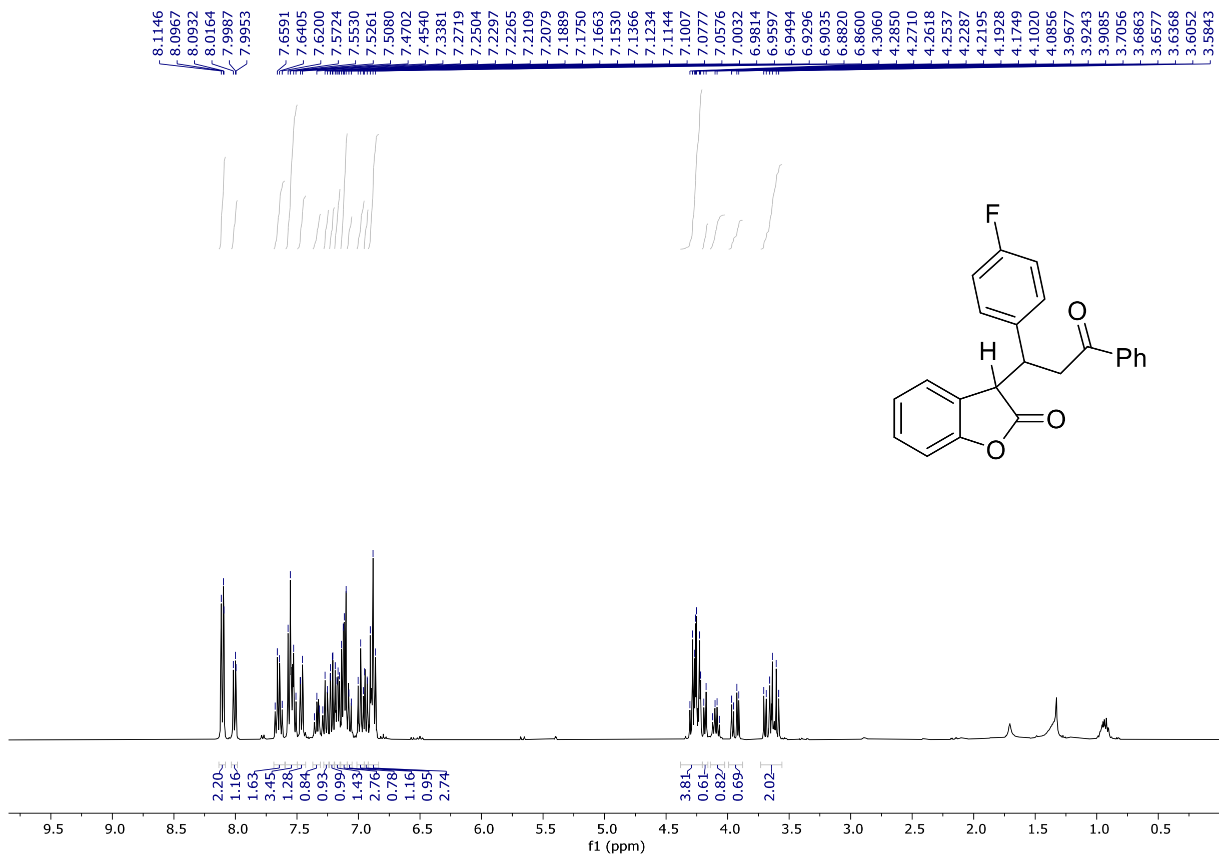

$^{13}C\{^{1}H\}$ NMR (100 MHz, $CDCl_3$)

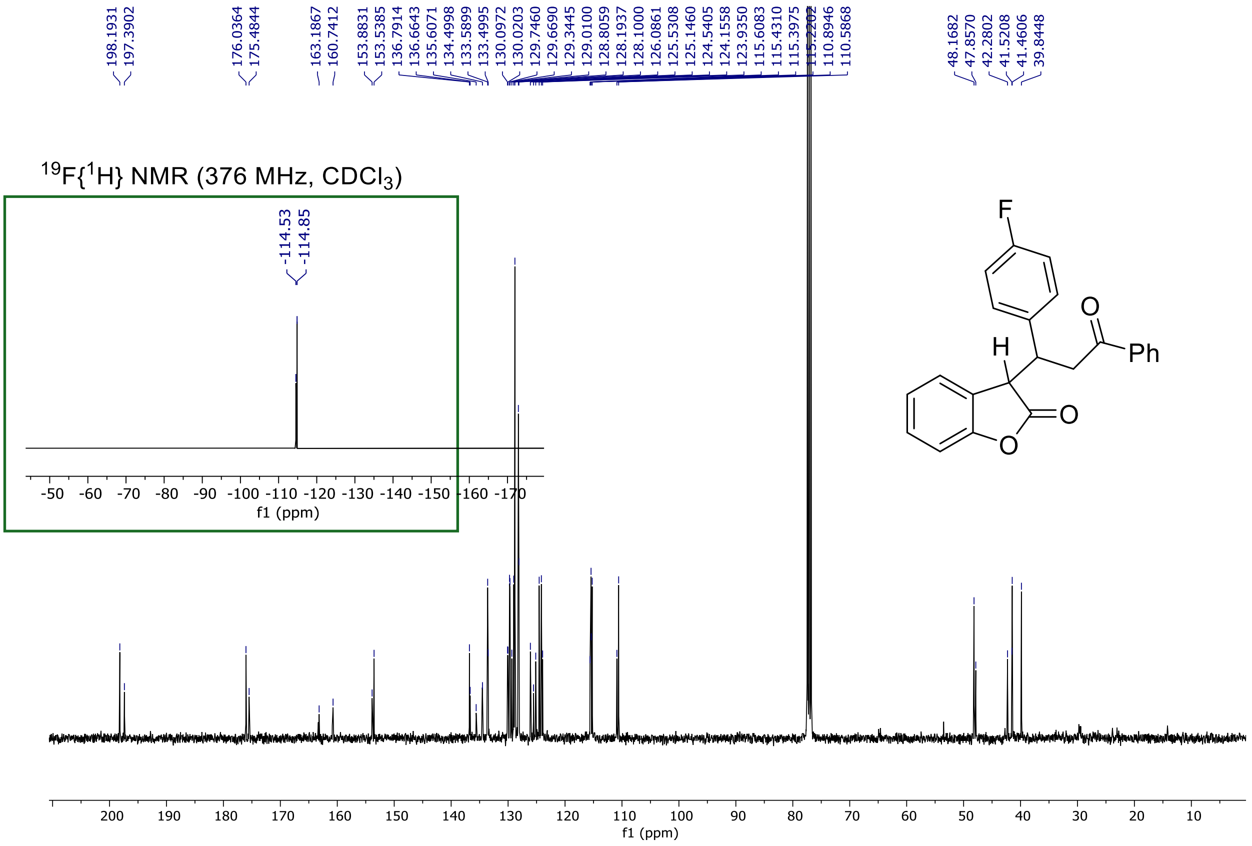

5-chloro-3-(3-oxo-1,3-diphenylpropyl)benzofuran-2(3H)-one (15h)

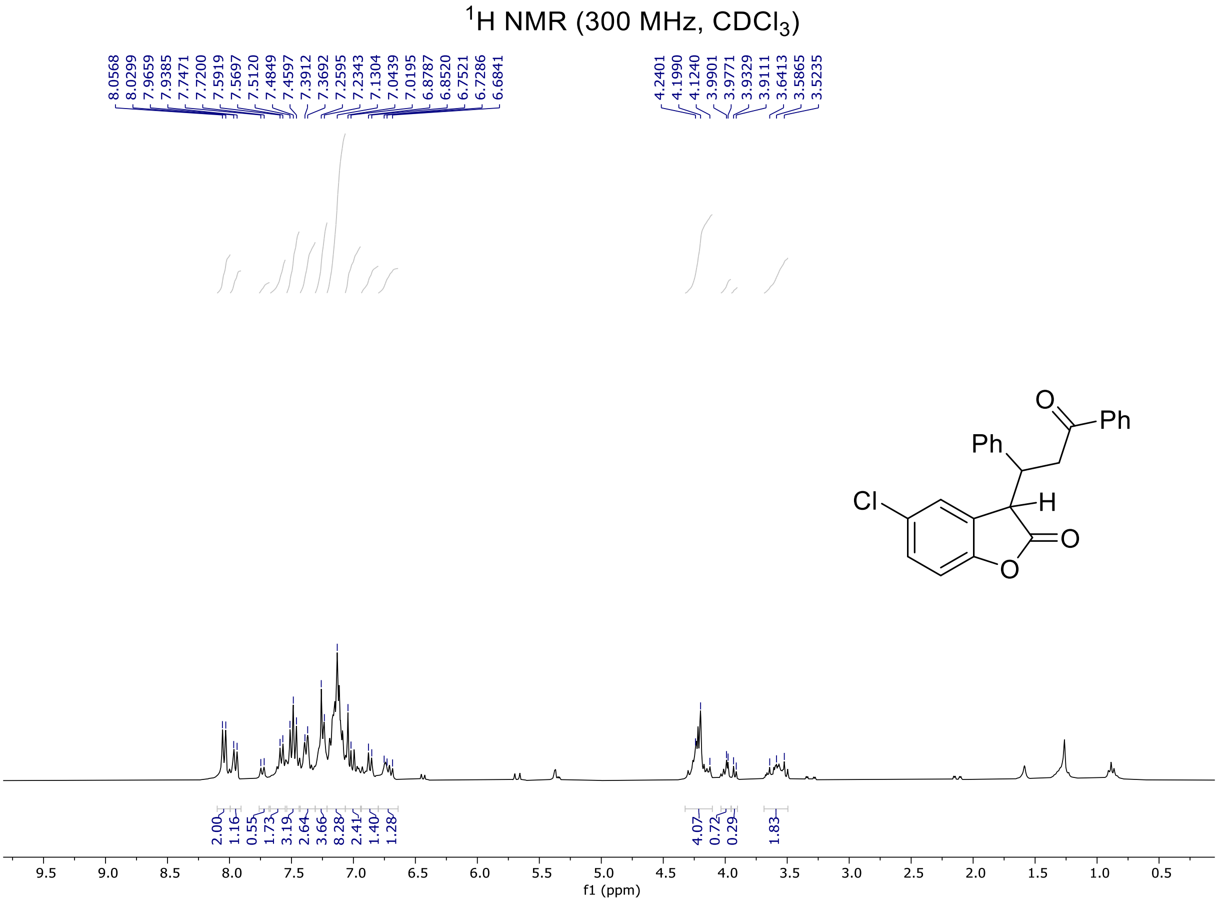

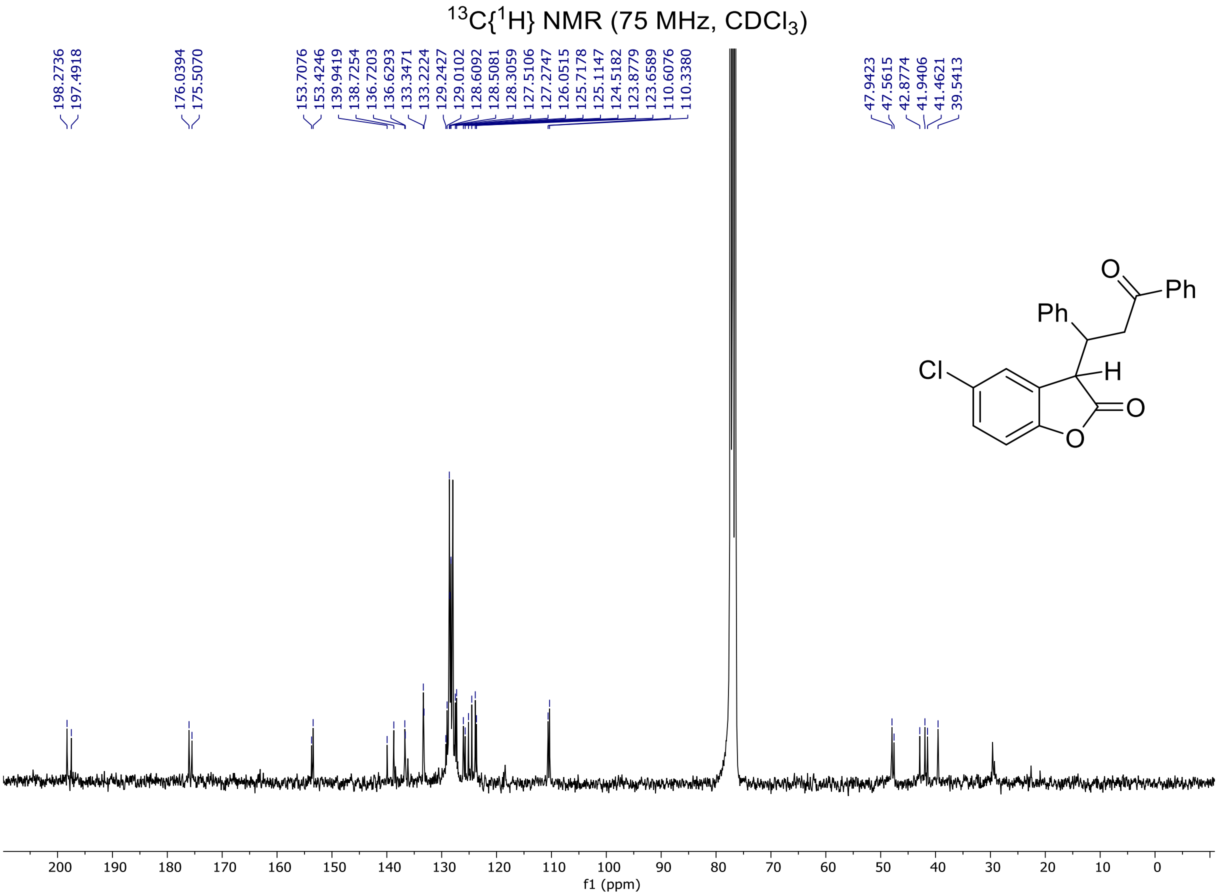

6. References

(S1) Serusi, L.; Di Mola, A.; Massa, A. *RSC Adv.* **2023**, *13*, 6557-6563.
(S2) Spangler, R. J.; Beckmann, B. G.; Kim J. H. *J. Org. Chem.* **1977**, 42, 2989–2995.
(S3) a) Kilikli, A. A.; Dengiz, C.; Özcan, S.; Balci, M. *Synthesis* **2011**, 3697-3705. b) Zhu, J.; Li, R.; Su, Y.; Gu, P. *J. Org. Chem.* **2019**, *84*, 5813.
(S4) Hawkinson, D. C.; Feiock, J. M.; Nevy, J. B.; Wu, Y. Z. *J. Org. Chem.* **1998**, *63*, 5345-5349.
(S5) Huang, Z.; Yang, X.; Yang, F.; Lu, T.; Zhou, Q. *Org. Lett*. **2017**, 19, 3524.
(S6) *SAINT*. Bruker AXS Inc., Madison, Wisconsin, USA, **2012**.
(S7) Sheldrick, G. M. *SADABS*, University of Göttingen, Germany, **1996**.
(S8) Sheldrick, G. M. *Acta Cryst*. **2015**, *A71*, 3-8.
(S9) Farrugia, L. J. *J. Appl. Cryst*. **2012**, *45*, 849-854.
(S10) *APEX3* (version 2015.5-2), Bruker AXS Inc., Madison, Wisconsin, USA, **2016**.
(S11) *SAINT* (version 8.34A), Bruker AXS Inc., Madison, Wisconsin, USA, **2013**.
(S12) *SADABS* (version 2014/5), Bruker AXS Inc., Madison, Wisconsin, USA, **2014**.
(S13) Sheldrick, G.M. *Acta Cryst*. **2008**, *A64*, 112-122.
(S14) Sheldrick, G.M. *Acta Cryst*. **2015**, *C71*, 3-8.
(S15) Dolomanov, O.V.; Bourhis, L.J.; Gildea, R.J.; Howard, J.A.K.; Puschmann, H. *J. Appl. Cryst*. **2009**, *42*, 339-341.
