# Supplementary material for: Lactone Enolates of Isochroman-3-ones and 2-Coumaranones: Quantification of Their Nucleophilicity in DMSO and Conjugate Additions to Chalcones
Source: J Org Chem. 2024 Apr 30;89(10):6915–28. doi: 10.1021/acs.joc.4c00277 (PMC11110064; doi:10.1021/acs.joc.4c00277)
Supplement: Supplementary file 2 — jo4c00277_si_002.zip [file jo4c00277_si_002.zip › 4+6a 3-isochro_NaH_BDM_1404/3-isochro_NaH_BDM_10eq.pdf]

Evaluation of kinetic data with ExpoFit V 1.3

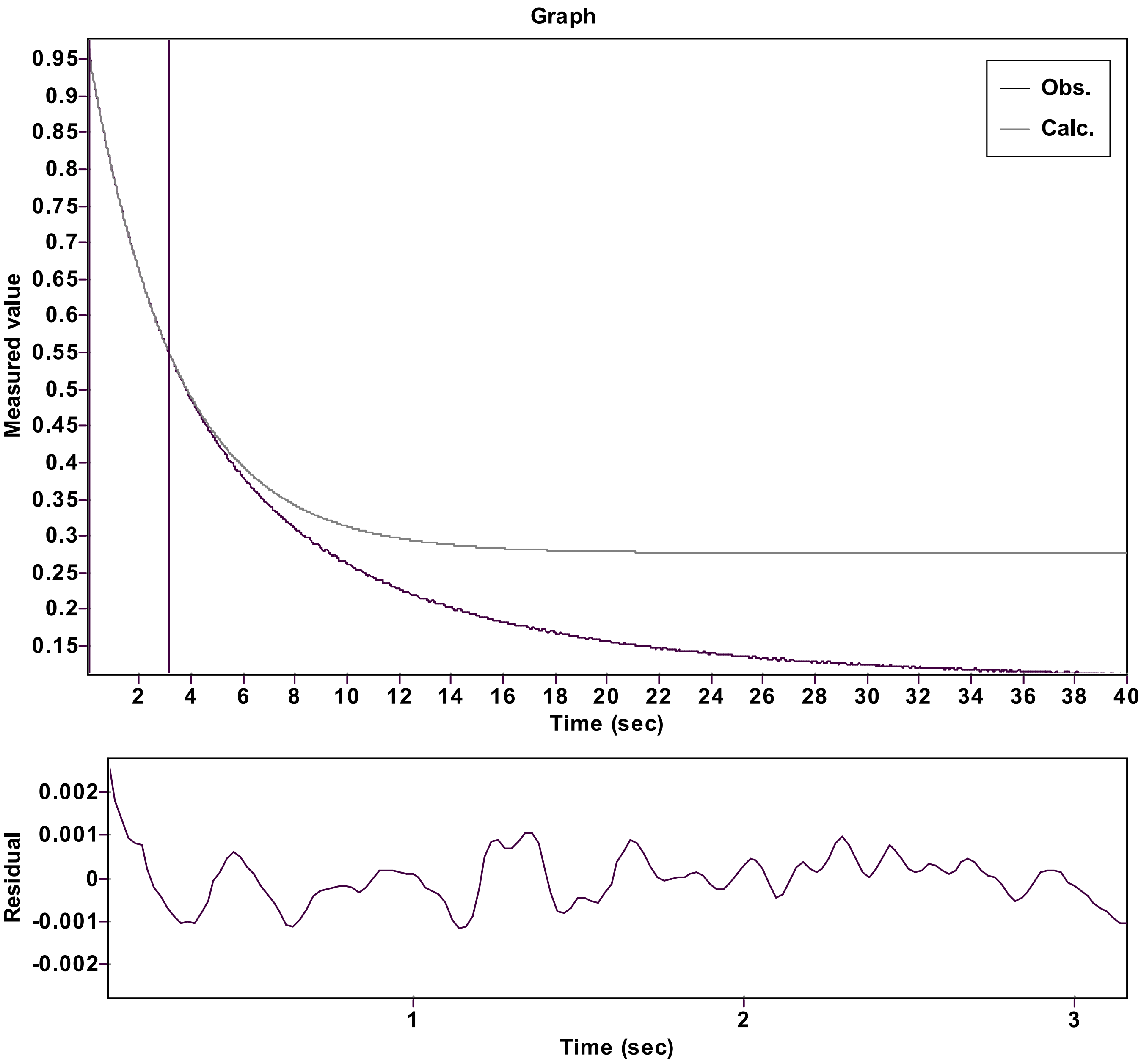

Function: y = A exp (-kx) + C (Exponential decrease)	Reference point: 0 (Zero)
Amp A = 0.692960845552670 ± 0.001462800358797	Quality r^2 = 0.9999736736549
Rate k = 0.295986001692491 ± 0.001063298465350	Data points = 155 of 2000
Final C = 0.276996989423280 ± 0.001585617246807	Conversion = 41.8 %

Start at position: 0.08 / 0.956521 (2.2 %)	End at position: 3.16 / 0.547905 (44.0 %)

ExpoFit file: 3-isochro_NaH_BDM_10eq.exp	Date of file: 14/04/2023 11:38:10
Source file: 3-isochro_NaH_BDM_10eq.txt	Date of file: 14/04/2023 11:00:46
Type of source file: Universal ASCII - file data	

2007 by Dr. Kempf	Date of print: 14/04/2023 11:39:19
