# Supplementary material for: Lactone Enolates of Isochroman-3-ones and 2-Coumaranones: Quantification of Their Nucleophilicity in DMSO and Conjugate Additions to Chalcones
Source: J Org Chem. 2024 Apr 30;89(10):6915–28. doi: 10.1021/acs.joc.4c00277 (PMC11110064; doi:10.1021/acs.joc.4c00277)
Supplement: Supplementary file 2 — jo4c00277_si_002.zip [file jo4c00277_si_002.zip › 4+6a 3-isochro_NaH_BDM_1404/3-isochro_NaH_BDM_20eq.pdf]

Evaluation of kinetic data with ExpoFit V 1.3

Graph

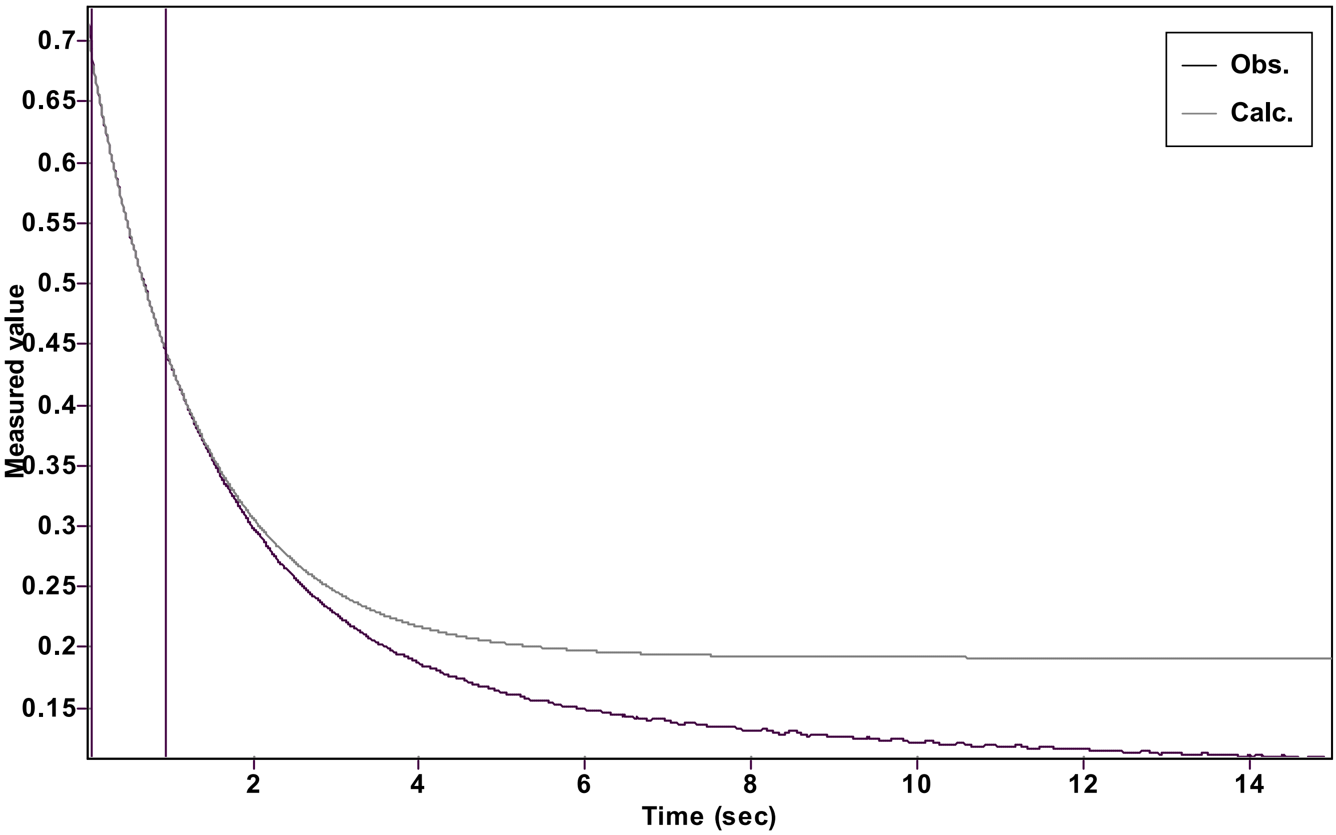

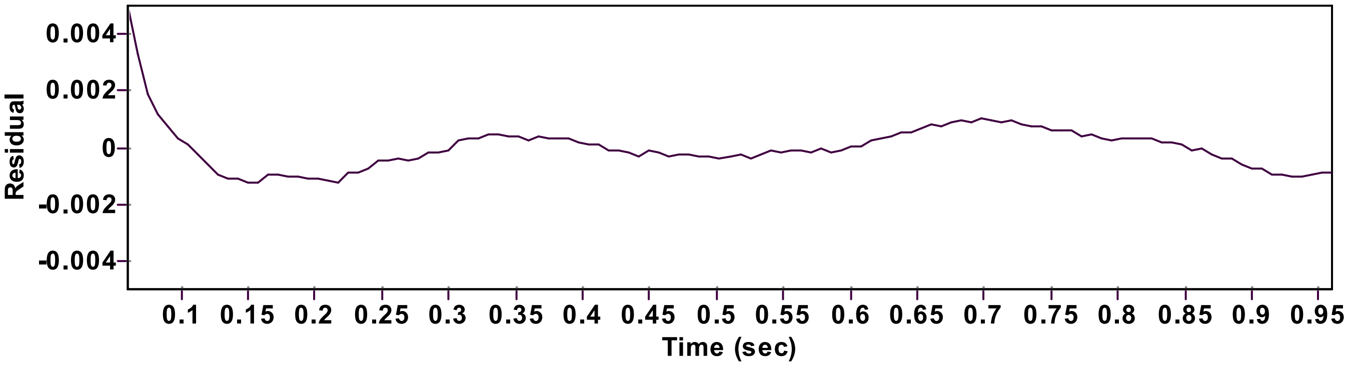

Function: y = A exp (-kx) + C (Exponential decrease)	Reference point: 0 (Zero)
Amp A = 0.515920698703860 ± 0.004217665624806	Quality r^2 = 0.9998610893296
Rate k = 0.749868398443423 ± 0.009367626160602	Data points = 121 of 2000
Final C = 0.191395942687874 ± 0.004467995044954	Conversion = 34.0 %

Start at position: 0.06 / 0.689575 (5.3 %)

End at position: 0.96 / 0.441678 (39.4 %)

ExpoFit file:	3-isochro_NaH_BDM_20eq.exp	Date of file:	14/04/2023 14:38:52
Source file:	3-isochro_NaH_BDM_20eq.txt	Date of file:	14/04/2023 11:26:32
Type of source file:	Universal ASCII - file data		

2007 by Dr. Kempf

Date of print: 14/04/2023 14:39:20
