# Supplementary material for: Lactone Enolates of Isochroman-3-ones and 2-Coumaranones: Quantification of Their Nucleophilicity in DMSO and Conjugate Additions to Chalcones
Source: J Org Chem. 2024 Apr 30;89(10):6915–28. doi: 10.1021/acs.joc.4c00277 (PMC11110064; doi:10.1021/acs.joc.4c00277)
Supplement: Supplementary file 2 — jo4c00277_si_002.zip [file jo4c00277_si_002.zip › 4+6a 3-isochro_NaH_BDM_1404/3-isochro_NaH_BDM_30eq.pdf]

Evaluation of kinetic data with ExpoFit V 1.3

Graph

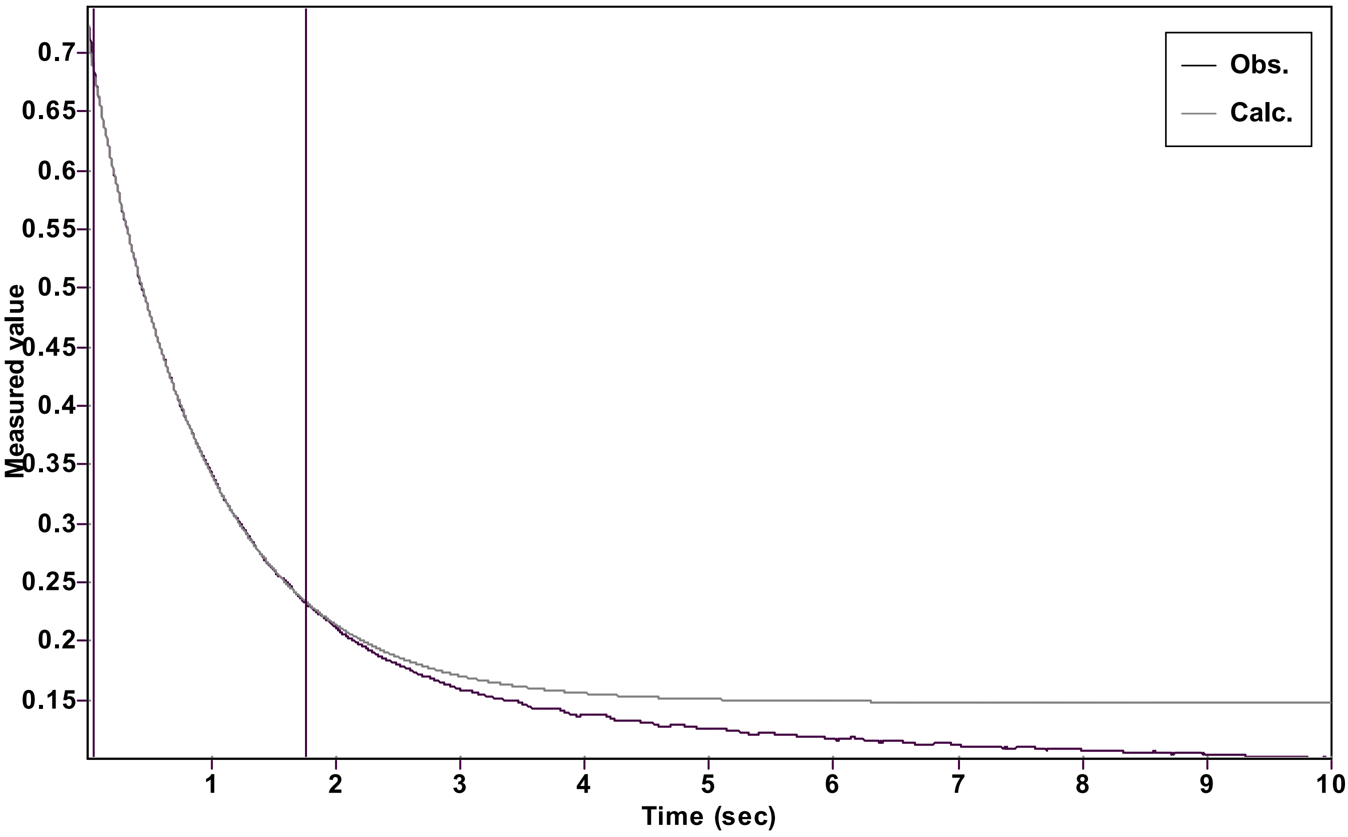

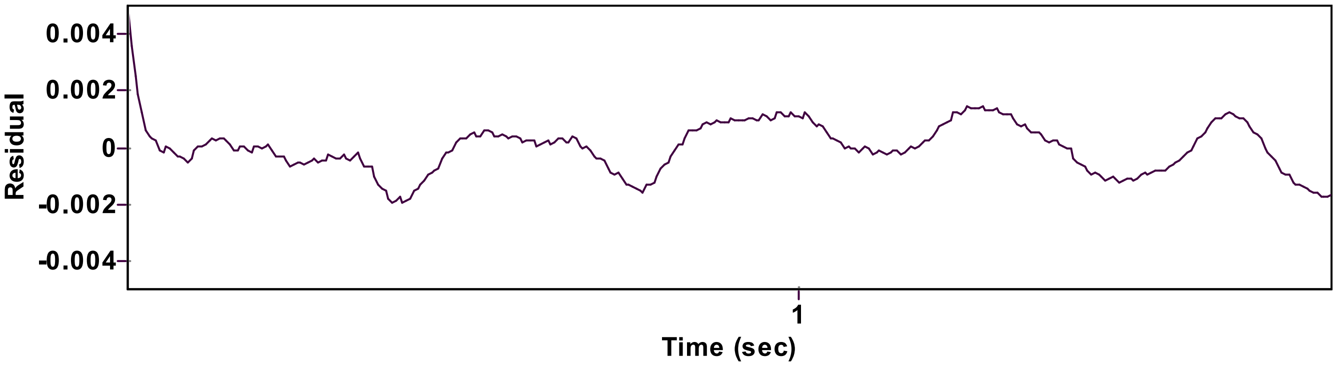

Function: y = A exp (-kx) + C (Exponential decrease)	Reference point: 0 (Zero)
Amp A = 0.569236136897434 ± 0.000324273700609<br>Rate k = 1.077576760262663 ± 0.001865087180498<br>Final C = 0.147993580583781 ± 0.000419354603685	Quality r^2 = 0.9999508733899<br>Data points = 342 of 2000<br>Conversion = 62.2 %
Start at position: 0.05 / 0.69238 (6.4 %)	End at position: 1.755 / 0.232227 (68.6 %)
ExpoFit file: 3-isochro_NaH_BDM_30eq.exp<br>Source file: 3-isochro_NaH_BDM_30eq.txt<br>Type of source file: Universal ASCII - file data	Date of file: 14/04/2023 14:40:40<br>Date of file: 14/04/2023 11:21:46
2007 by Dr. Kempf	Date of print: 14/04/2023 14:41:45
