# Supplementary material for: Lactone Enolates of Isochroman-3-ones and 2-Coumaranones: Quantification of Their Nucleophilicity in DMSO and Conjugate Additions to Chalcones
Source: J Org Chem. 2024 Apr 30;89(10):6915–28. doi: 10.1021/acs.joc.4c00277 (PMC11110064; doi:10.1021/acs.joc.4c00277)
Supplement: Supplementary file 2 — jo4c00277_si_002.zip [file jo4c00277_si_002.zip › 4+6a 3-isochro_NaH_BDM_1404/3-isochro_NaH_BDM_40eq.pdf]

Evaluation of kinetic data with ExpoFit V 1.3

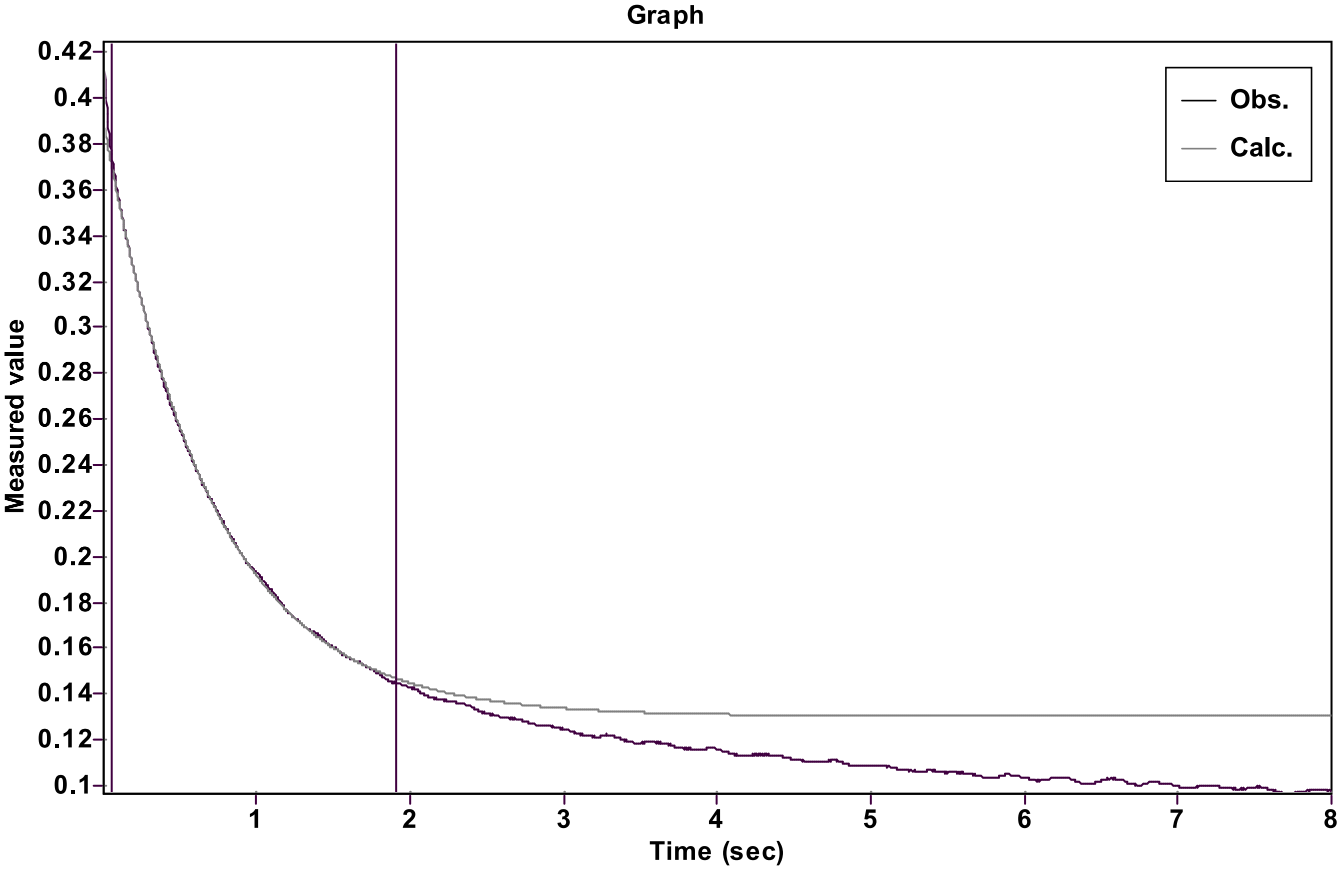

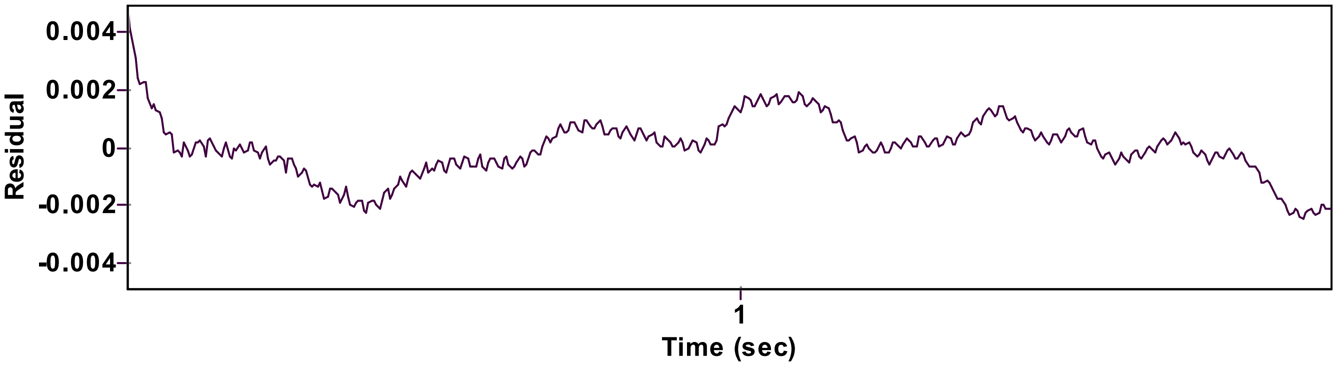

**Function:** y = A exp (-kx) + C (Exponential decrease)	**Reference point:** 0 (Zero)
**Amp A =** 0.260445797184460 ± 0.000204928684016<br>**Rate k =** 1.446632138166796 ± 0.003816084207923<br>**Final C =** 0.130371931616298 ± 0.000214527198969	**Quality** r^2 = 0.9997158824421<br>**Data points** = 465 of 2000<br>**Conversion** = 54.3 %
**Start at position:** 0.056 / 0.375457 (11.6 %)	**End at position:** 1.912 / 0.144653 (65.9 %)

ExpoFit file: 3-isochro_NaH_BDM_40eq.exp — **Date of file:** 14/04/2023 14:43:00

Source file: 3-isochro_NaH_BDM_40eq.txt — **Date of file:** 14/04/2023 11:29:54

Type of source file: Universal ASCII - file data

2007 by Dr. Kempf — **Date of print:** 14/04/2023 14:43:19
