# Supplementary material for: Lactone Enolates of Isochroman-3-ones and 2-Coumaranones: Quantification of Their Nucleophilicity in DMSO and Conjugate Additions to Chalcones
Source: J Org Chem. 2024 Apr 30;89(10):6915–28. doi: 10.1021/acs.joc.4c00277 (PMC11110064; doi:10.1021/acs.joc.4c00277)
Supplement: Supplementary file 2 — jo4c00277_si_002.zip [file jo4c00277_si_002.zip › 4+6b 3-isochro_NaH_Jul-tbu1304_repeat 2/3-isochro_NaH_Jul-tbu1304_20eq_2.pdf]

Evaluation of kinetic data with ExpoFit V 1.3

Graph

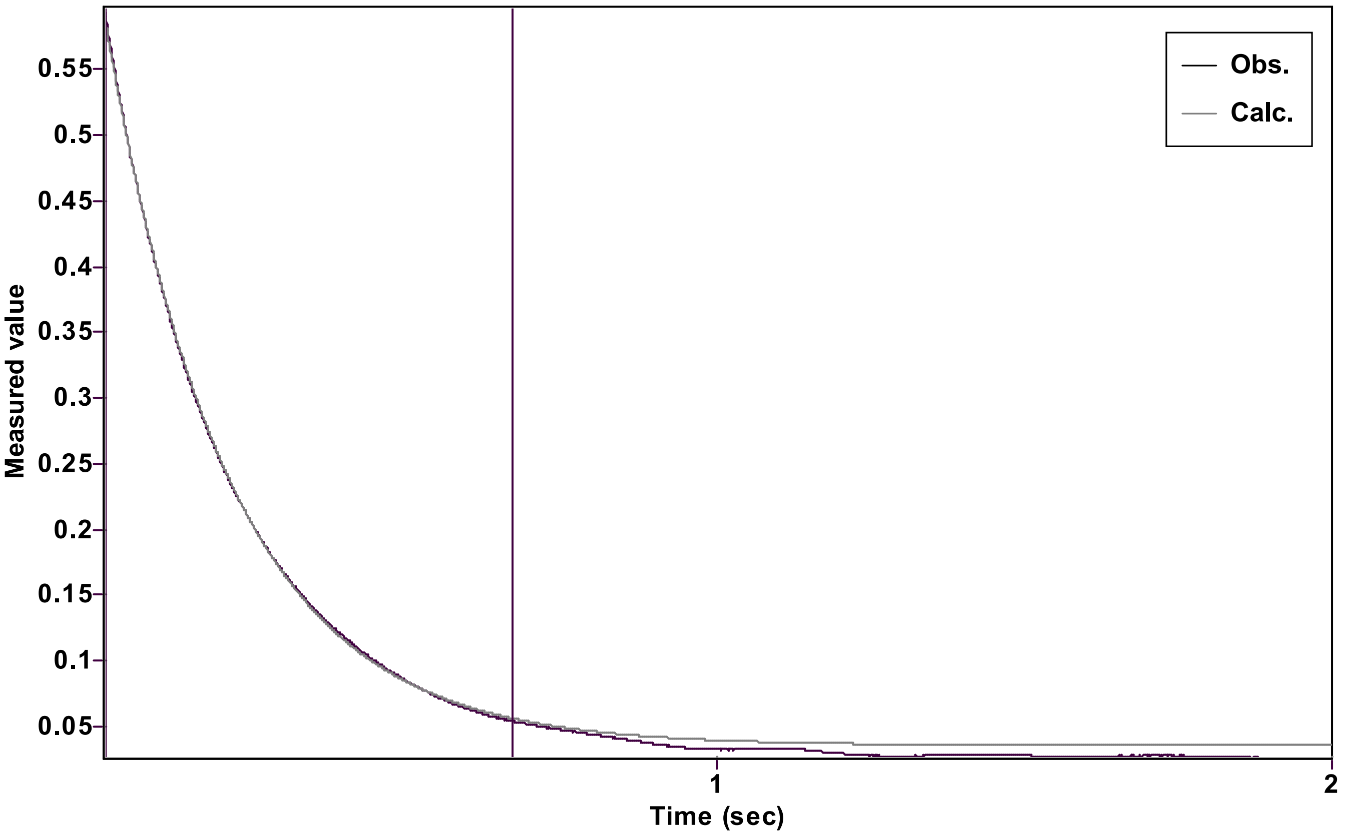

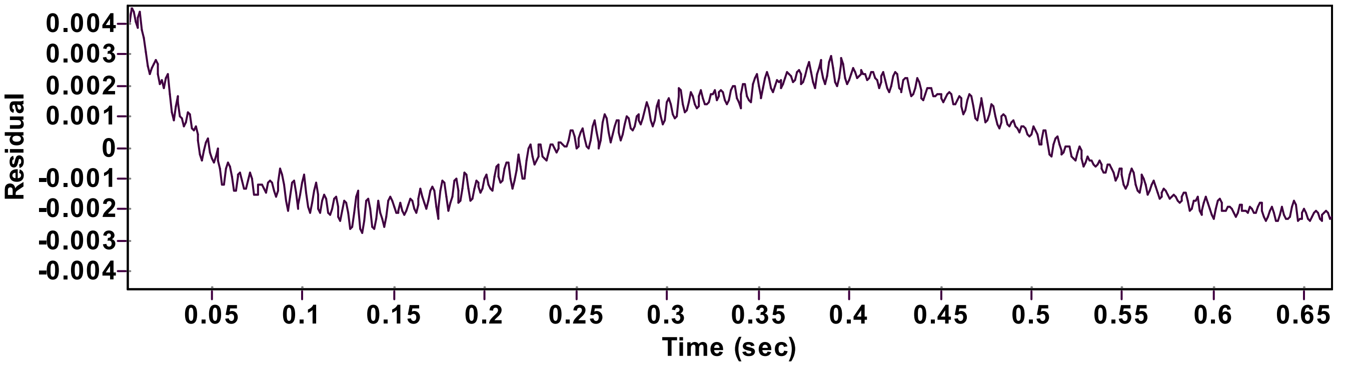

**Function:** y = A exp (-kx) + C (Exponential decrease)	**Reference point:** 0 (Zero)
**Amp A =** 0.561366962154428 ± 0.000256598745908	**Quality** r^2 = 0.9998630943129
**Rate k =** 4.960519527721205 ± 0.006643221469039	**Data points** = 662 of 2000
**Final C =** 0.035640356185450 ± 0.000211676608466	**Conversion** = 89.7 %
**Start at position:** 0.004 / 0.590545 (1.3 %)	**End at position:** 0.665 / 0.0541533 (90.9 %)
**ExpoFit file:** 3-isochro_NaH_Jul-tbu1304_20eq_2.exp	**Date of file:** 13/04/2023 14:32:32
**Source file:** 3-isochro_NaH_Jul-tbu1304_20eq_2.txt	**Date of file:** 13/04/2023 14:15:36
**Type of source file:** Universal ASCII - file data	
2007 by Dr. Kempf	**Date of print:** 13/04/2023 14:32:46
