# Supplementary material for: Lactone Enolates of Isochroman-3-ones and 2-Coumaranones: Quantification of Their Nucleophilicity in DMSO and Conjugate Additions to Chalcones
Source: J Org Chem. 2024 Apr 30;89(10):6915–28. doi: 10.1021/acs.joc.4c00277 (PMC11110064; doi:10.1021/acs.joc.4c00277)
Supplement: Supplementary file 2 — jo4c00277_si_002.zip [file jo4c00277_si_002.zip › 4+6b 3-isochro_NaH_Jul-tbu1304_repeat 2/3-isochro_NaH_Jul-tbu1304_30eq_2.pdf]

Evaluation of kinetic data with ExpoFit V 1.3

Graph

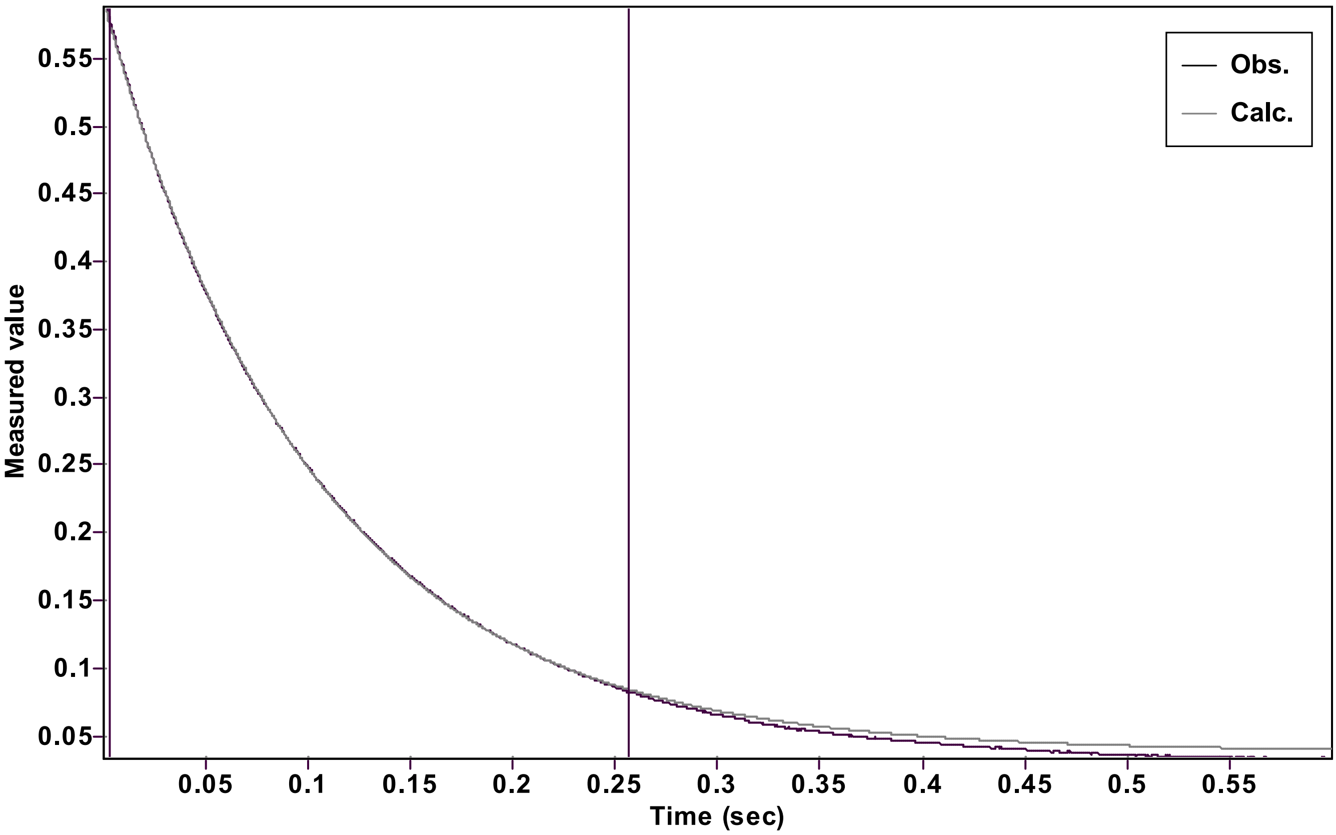

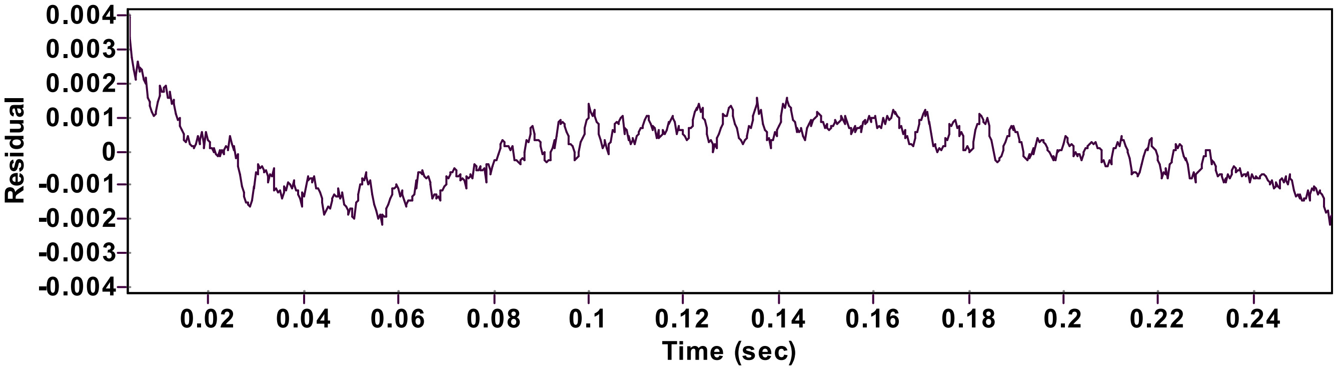

Function: y = A exp (-kx) + C (Exponential decrease)	Reference point: 0 (Zero)
Amp A = 0.554419764329734 ± 0.000137939220876<br>Rate k = 9.722969999526907 ± 0.008005352903657<br>Final C = 0.038476762785191 ± 0.000160574364912	Quality r^2 = 0.9999557340422<br>Data points = 846 of 2000<br>Conversion = 84.8 %
Start at position: 0.003 / 0.581135 (1.2 %)	End at position: 0.2565 / 0.0824102 (86.0 %)

ExpoFit file: 3-isochro_NaH_Jul-tbu1304_30eq_2.exp — Date of file: 13/04/2023 14:34:14

Source file: 3-isochro_NaH_Jul-tbu1304_30eq_2.txt — Date of file: 13/04/2023 14:19:12

Type of source file: Universal ASCII - file data

2007 by Dr. Kempf — Date of print: 13/04/2023 14:36:58
