# Supplementary material for: Lactone Enolates of Isochroman-3-ones and 2-Coumaranones: Quantification of Their Nucleophilicity in DMSO and Conjugate Additions to Chalcones
Source: J Org Chem. 2024 Apr 30;89(10):6915–28. doi: 10.1021/acs.joc.4c00277 (PMC11110064; doi:10.1021/acs.joc.4c00277)
Supplement: Supplementary file 2 — jo4c00277_si_002.zip [file jo4c00277_si_002.zip › 4+6b 3-isochro_NaH_Jul-tbu1304_repeat 2/3-isochro_NaH_Jul-tbu1304_40eq_2.pdf]

Evaluation of kinetic data with ExpoFit V 1.3

Graph

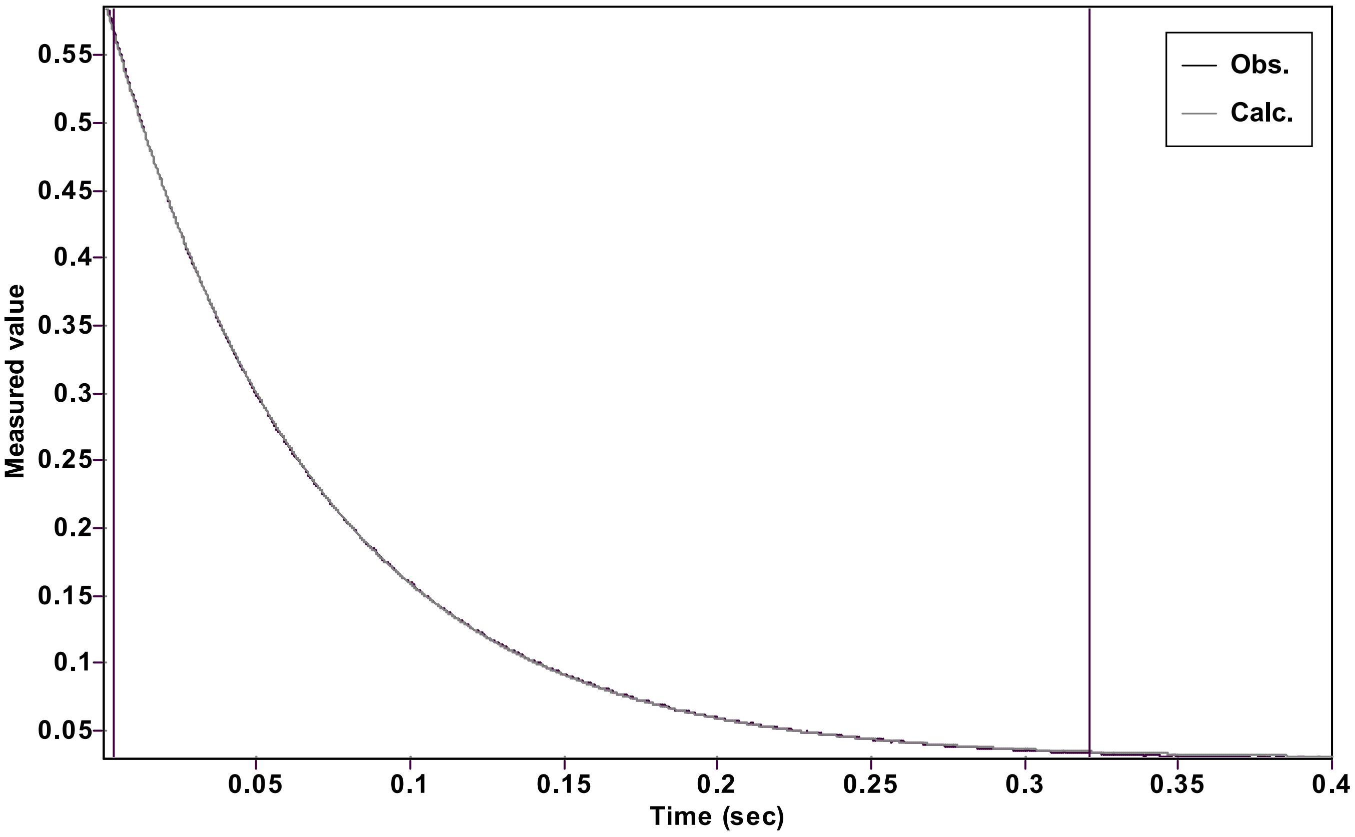

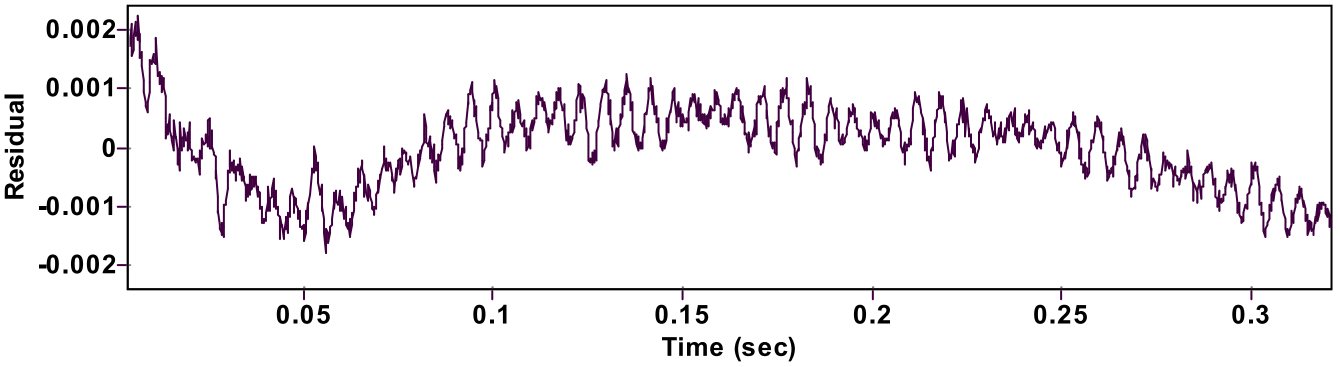

Function: y = A exp (-kx) + C (Exponential decrease)	Reference point: 0 (Zero)
Amp A = 0.565645393907437 ± 0.000082563815161<br>Rate k = 14.75266561156641 ± 0.004617660670498<br>Final C = 0.029240834836262 ± 0.000035877085873	Quality r^2 = 0.9999738700595<br>Data points = 1589 of 2000<br>Conversion = 91.3 %
Start at position: 0.0036 / 0.56803 (3.1 %)	End at position: 0.3212 / 0.0328539 (94.4 %)
ExpoFit file: 3-isochro_NaH_Jul-tbu1304_40eq_2.exp<br>Source file: 3-isochro_NaH_Jul-tbu1304_40eq_2.txt<br>Type of source file: Universal ASCII - file data	Date of file: 13/04/2023 14:38:04<br>Date of file: 13/04/2023 14:23:28
2007 by Dr. Kempf	Date of print: 13/04/2023 14:38:15
