# Supplementary material for: Lactone Enolates of Isochroman-3-ones and 2-Coumaranones: Quantification of Their Nucleophilicity in DMSO and Conjugate Additions to Chalcones
Source: J Org Chem. 2024 Apr 30;89(10):6915–28. doi: 10.1021/acs.joc.4c00277 (PMC11110064; doi:10.1021/acs.joc.4c00277)
Supplement: Supplementary file 2 — jo4c00277_si_002.zip [file jo4c00277_si_002.zip › 4+6b 3-isochro_NaH_Jul-tbu1304_repeat 2/3-isochro_NaH_Jul-tbu1304_50eq_2.pdf]

Evaluation of kinetic data with ExpoFit V 1.3

Graph

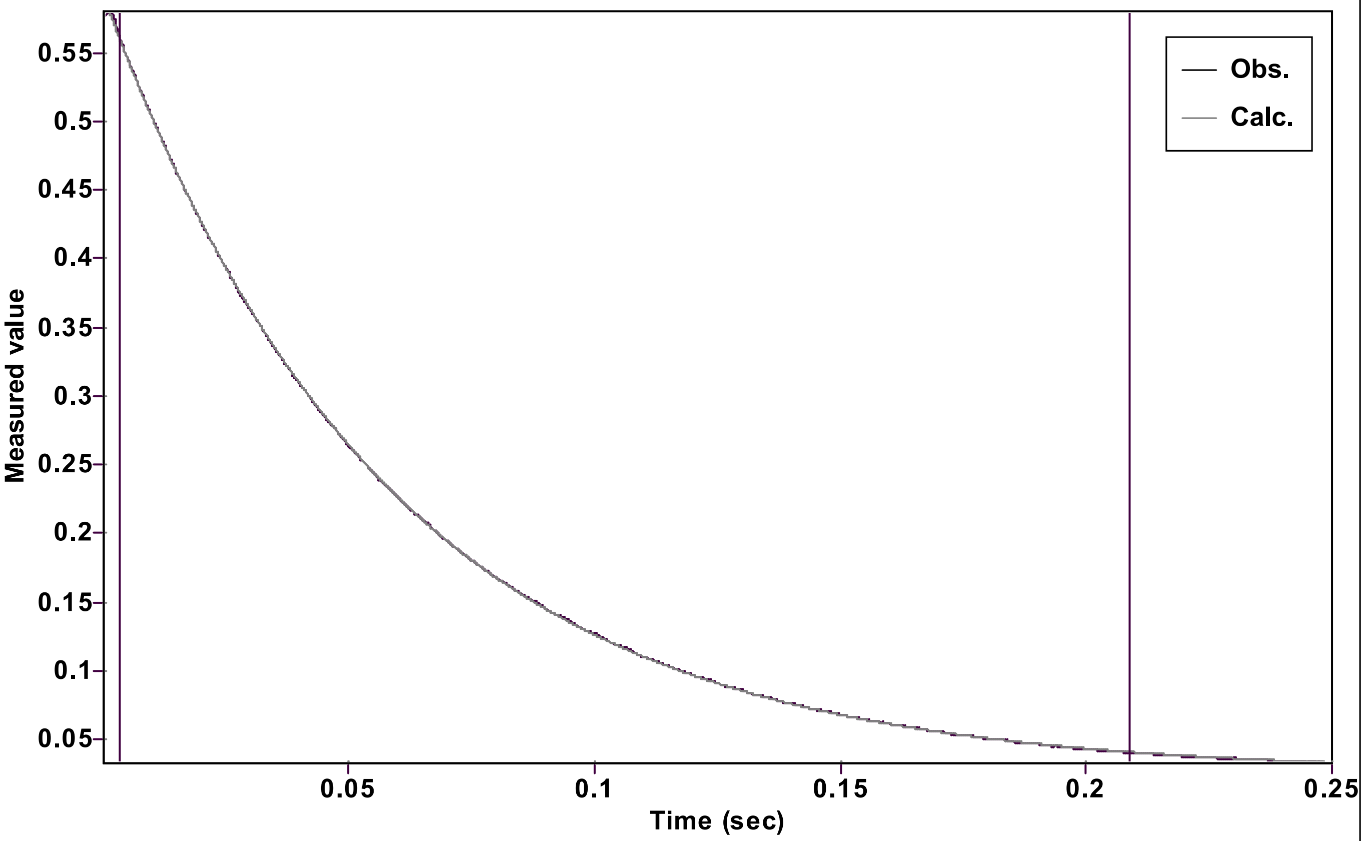

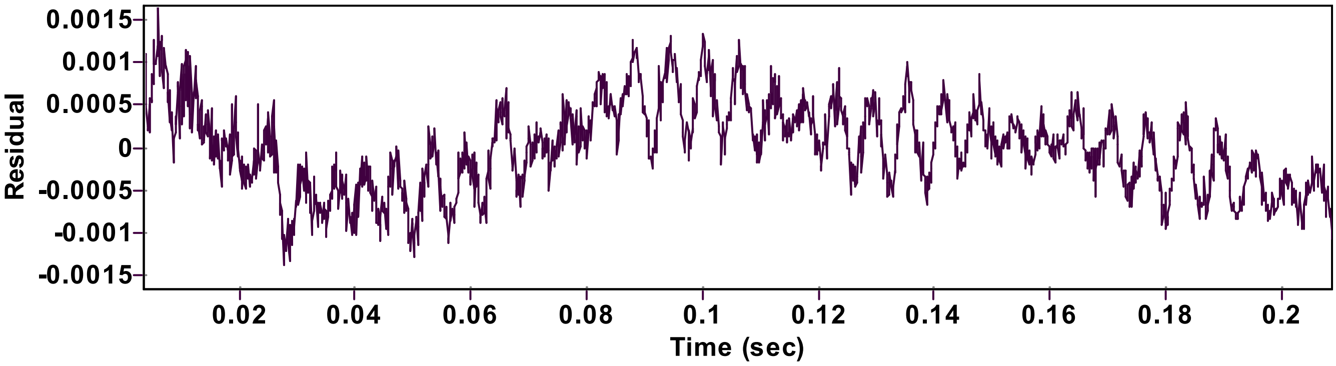

Function: y = A exp (-kx) + C (Exponential decrease)	Reference point: 0 (Zero)
Amp A = 0.566984151530013 ± 0.000054100258268	Quality r^2 = 0.9999937813141
Rate k = 17.30319859662310 ± 0.004435916794397	Data points = 1643 of 2000
Final C = 0.025461182821981 ± 0.000036746218727	Conversion = 89.7 %
Start at position: 0.0035 / 0.560254 (3.5 %)	End at position: 0.20875 / 0.0397061 (93.2 %)
ExpoFit file: 3-isochro_NaH_Jul-tbu1304_50eq_2.exp	Date of file: 13/04/2023 14:41:26
Source file: 3-isochro_NaH_Jul-tbu1304_50eq_2.txt	Date of file: 13/04/2023 14:26:42
Type of source file: Universal ASCII - file data	
2007 by Dr. Kempf	Date of print: 13/04/2023 14:41:37
