# Supplementary material for: Lactone Enolates of Isochroman-3-ones and 2-Coumaranones: Quantification of Their Nucleophilicity in DMSO and Conjugate Additions to Chalcones
Source: J Org Chem. 2024 Apr 30;89(10):6915–28. doi: 10.1021/acs.joc.4c00277 (PMC11110064; doi:10.1021/acs.joc.4c00277)
Supplement: Supplementary file 2 — jo4c00277_si_002.zip [file jo4c00277_si_002.zip › 4+6c 3-isochro_crown_NaH_dma-QM1704/3-isochro_crown_NaH_dma-QM_10eq.pdf]

Evaluation of kinetic data with ExpoFit V 1.3

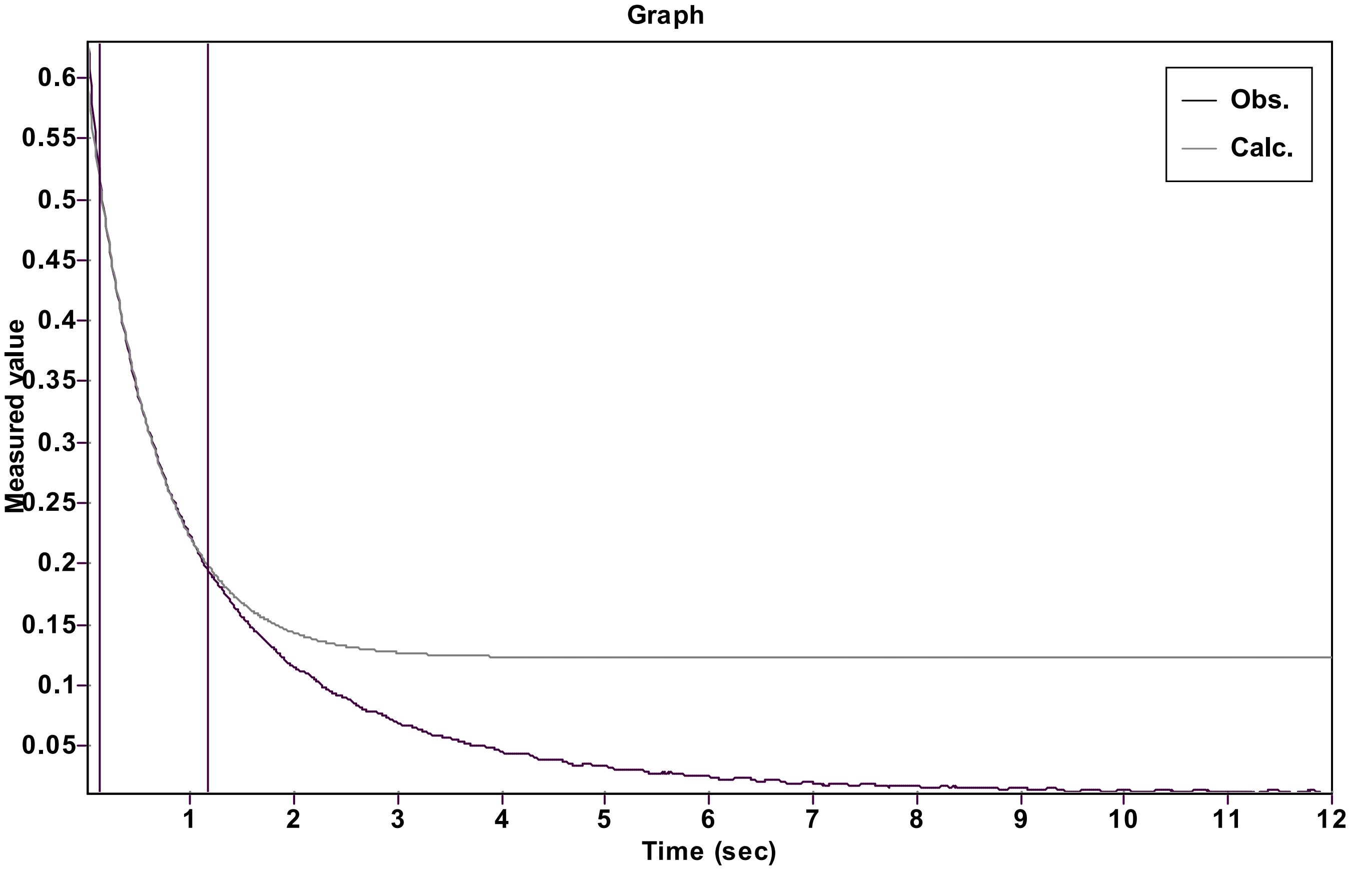

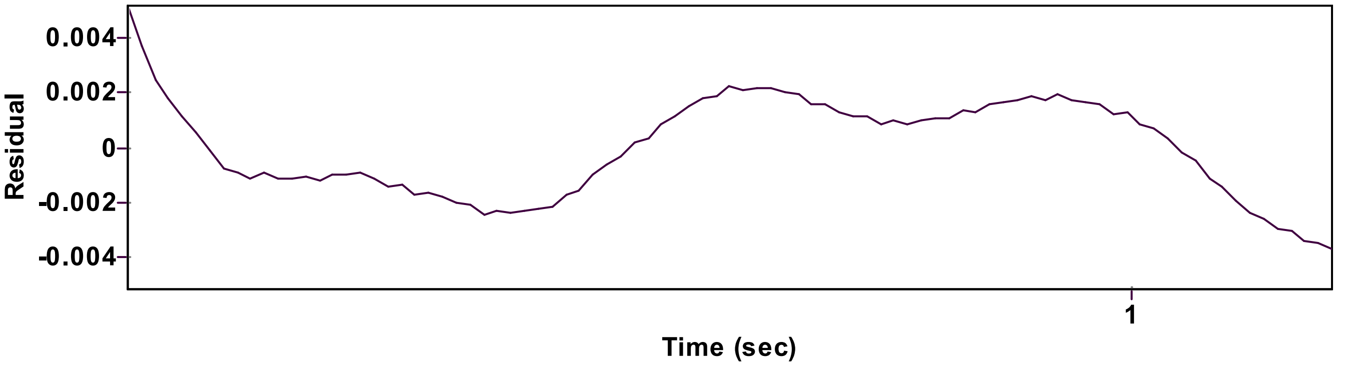

Function: y = A exp (-kx) + C (Exponential decrease)	Reference point: 0 (Zero)
Amp A = 0.479682013678411 ± 0.001294372049062<br>Rate k = 1.576321740998694 ± 0.016389681877657<br>Final C = 0.122775685006831 ± 0.001980848216786	Quality r^2 = 0.9996143853889<br>Data points = 89 of 1000<br>Conversion = 52.5 %
Start at position: 0.12 / 0.524999 (16.6 %)	End at position: 1.176 / 0.194195 (69.2 %)
ExpoFit file: 3-isochro_crown_NaH_dma-QM_10eq.exp<br>Source file: 3-isochro_crown_NaH_dma-QM_10eq.txt<br>Type of source file: Universal ASCII - file data	Date of file: 17/04/2023 13:33:54<br>Date of file: 17/04/2023 11:20:48
2007 by Dr. Kempf	Date of print: 17/04/2023 13:34:19
