# Supplementary material for: Lactone Enolates of Isochroman-3-ones and 2-Coumaranones: Quantification of Their Nucleophilicity in DMSO and Conjugate Additions to Chalcones
Source: J Org Chem. 2024 Apr 30;89(10):6915–28. doi: 10.1021/acs.joc.4c00277 (PMC11110064; doi:10.1021/acs.joc.4c00277)
Supplement: Supplementary file 2 — jo4c00277_si_002.zip [file jo4c00277_si_002.zip › 4+6c 3-isochro_crown_NaH_dma-QM1704/3-isochro_crown_NaH_dma-QM_20eq.pdf]

Evaluation of kinetic data with ExpoFit V 1.3

Graph

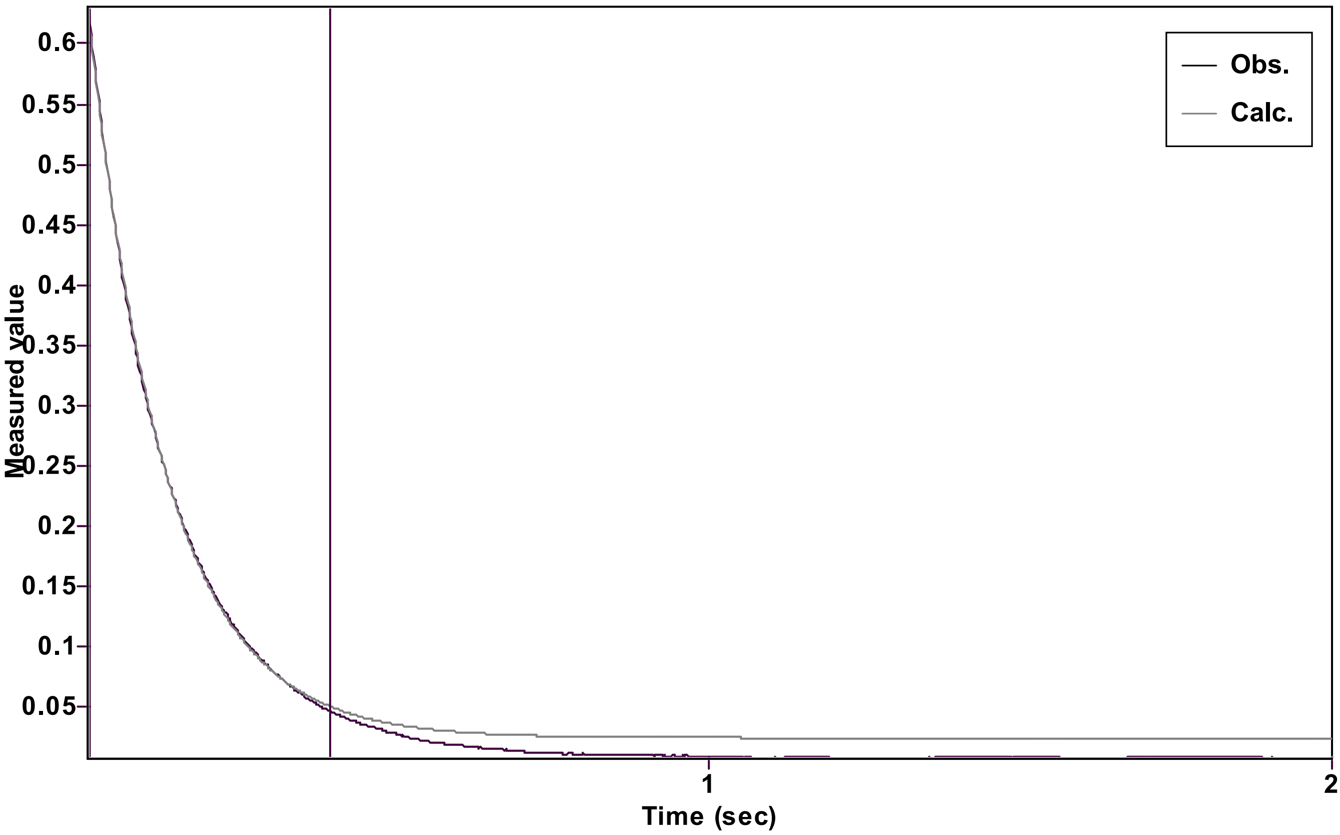

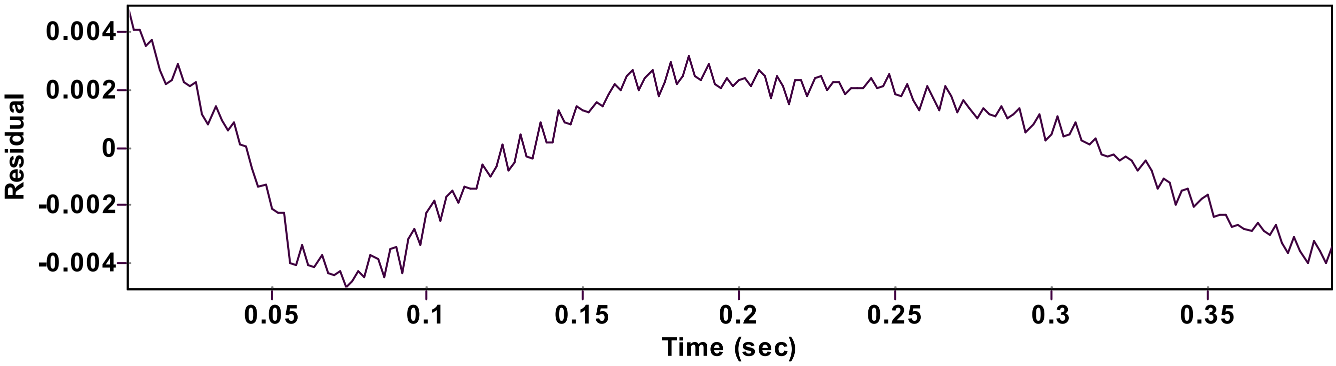

**Function:** y = A exp (-kx) + C (Exponential decrease)	**Reference point:** 0 (Zero)
**Amp A =** 0.618108011577652 ± 0.000671309141530<br>**Rate k =** 8.091546827661963 ± 0.026552344017372<br>**Final C =** 0.024190484930985 ± 0.000588849588080	**Quality** r^2 = 0.9997748463974<br>**Data points** = 194 of 1000<br>**Conversion** = 92.0 %
**Start at position:** 0.004 / 0.627492 (0.5 %)	**End at position:** 0.39 / 0.0470748 (92.5 %)

ExpoFit file: 3-isochro_crown_NaH_dma-QM_20eq.exp — **Date of file:** 17/04/2023 13:35:06

Source file: 3-isochro_crown_NaH_dma-QM_20eq.txt — **Date of file:** 17/04/2023 11:24:20

Type of source file: Universal ASCII - file data

2007 by Dr. Kempf — **Date of print:** 17/04/2023 13:35:14
