# Supplementary material for: Lactone Enolates of Isochroman-3-ones and 2-Coumaranones: Quantification of Their Nucleophilicity in DMSO and Conjugate Additions to Chalcones
Source: J Org Chem. 2024 Apr 30;89(10):6915–28. doi: 10.1021/acs.joc.4c00277 (PMC11110064; doi:10.1021/acs.joc.4c00277)
Supplement: Supplementary file 2 — jo4c00277_si_002.zip [file jo4c00277_si_002.zip › 4+6c 3-isochro_crown_NaH_dma-QM1704/3-isochro_crown_NaH_dma-QM_30eq.pdf]

Evaluation of kinetic data with ExpoFit V 1.3

Graph

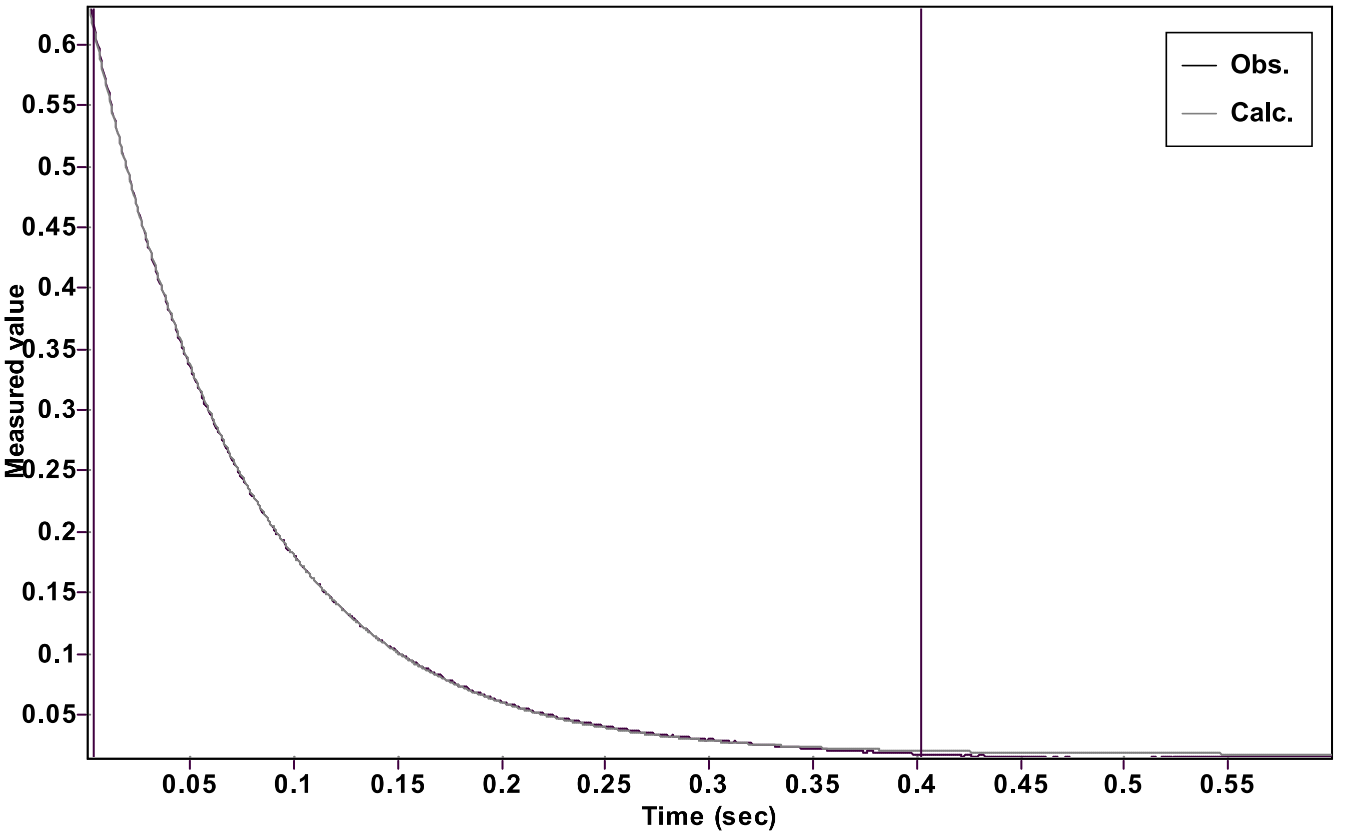

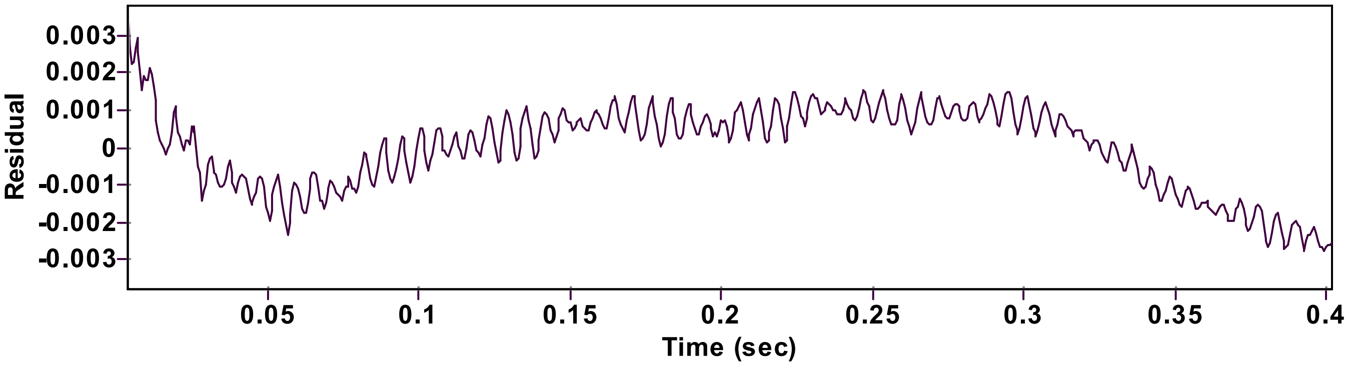

Function: y = A exp (-kx) + C (Exponential decrease)	Reference point: 0 (Zero)
Amp A = 0.624842392264916 ± 0.000224170843251	Quality r^2 = 0.9999387552287
Rate k = 13.42868509699803 ± 0.009514606447456	Data points = 665 of 1000
Final C = 0.017245717692077 ± 0.000081371104636	Conversion = 94.9 %
Start at position: 0.0036 / 0.616398 (2.4 %)	End at position: 0.402 / 0.0175391 (97.2 %)
ExpoFit file: 3-isochro_crown_NaH_dma-QM_30eq.exp	Date of file: 17/04/2023 13:37:04
Source file: 3-isochro_crown_NaH_dma-QM_30eq.txt	Date of file: 17/04/2023 11:27:44
Type of source file: Universal ASCII - file data	
2007 by Dr. Kempf	Date of print: 17/04/2023 13:37:13
