# Supplementary material for: Lactone Enolates of Isochroman-3-ones and 2-Coumaranones: Quantification of Their Nucleophilicity in DMSO and Conjugate Additions to Chalcones
Source: J Org Chem. 2024 Apr 30;89(10):6915–28. doi: 10.1021/acs.joc.4c00277 (PMC11110064; doi:10.1021/acs.joc.4c00277)
Supplement: Supplementary file 2 — jo4c00277_si_002.zip [file jo4c00277_si_002.zip › 4+6c 3-isochro_crown_NaH_dma-QM1704/3-isochro_crown_NaH_dma-QM_40eq.pdf]

Evaluation of kinetic data with ExpoFit V 1.3

Graph

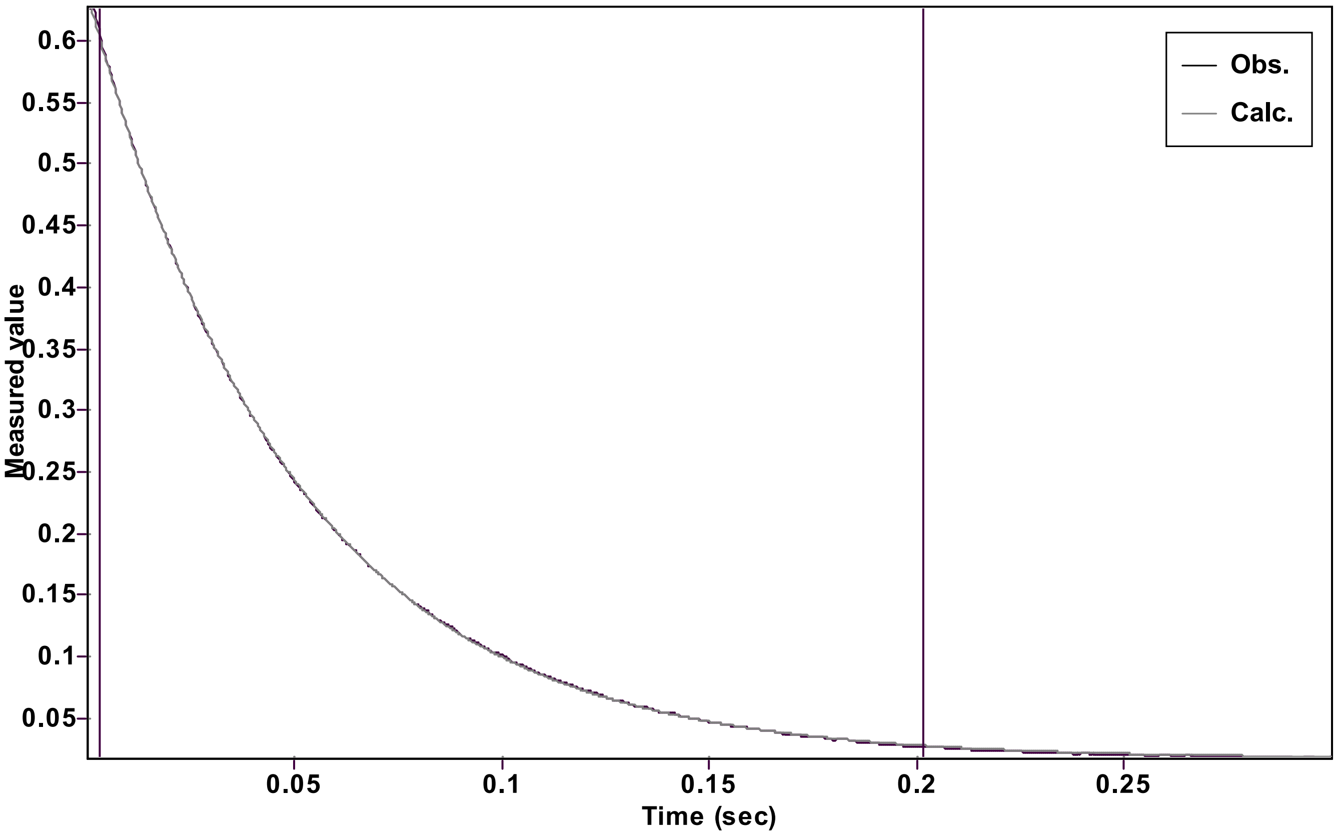

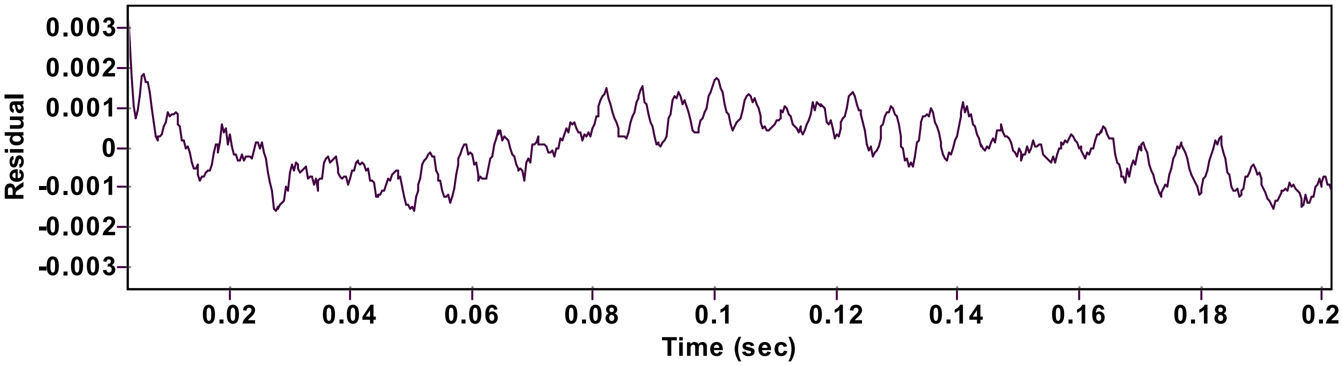

Function: y = A exp (-kx) + C (Exponential decrease)	Reference point: 0 (Zero)
Amp A = 0.624016690635938 ± 0.000135561524607<br>Rate k = 20.25607142290986 ± 0.010542409273503<br>Final C = 0.017432260855796 ± 0.000073670248836	Quality r^2 = 0.9999759435449<br>Data points = 662 of 1000<br>Conversion = 92.1 %
Start at position: 0.0033 / 0.604634 (3.7 %)	End at position: 0.2016 / 0.0268721 (95.7 %)
ExpoFit file: 3-isochro_crown_NaH_dma-QM_40eq.exp<br>Source file: 3-isochro_crown_NaH_dma-QM_40eq.txt<br>Type of source file: Universal ASCII - file data	Date of file: 17/04/2023 13:38:30<br>Date of file: 17/04/2023 11:30:06
2007 by Dr. Kempf	Date of print: 17/04/2023 13:40:09
