# Supplementary material for: Lactone Enolates of Isochroman-3-ones and 2-Coumaranones: Quantification of Their Nucleophilicity in DMSO and Conjugate Additions to Chalcones
Source: J Org Chem. 2024 Apr 30;89(10):6915–28. doi: 10.1021/acs.joc.4c00277 (PMC11110064; doi:10.1021/acs.joc.4c00277)
Supplement: Supplementary file 2 — jo4c00277_si_002.zip [file jo4c00277_si_002.zip › 4+6c 3-isochro_crown_NaH_dma-QM1704/3-isochro_crown_NaH_dma-QM_50eq.pdf]

Evaluation of kinetic data with ExpoFit V 1.3

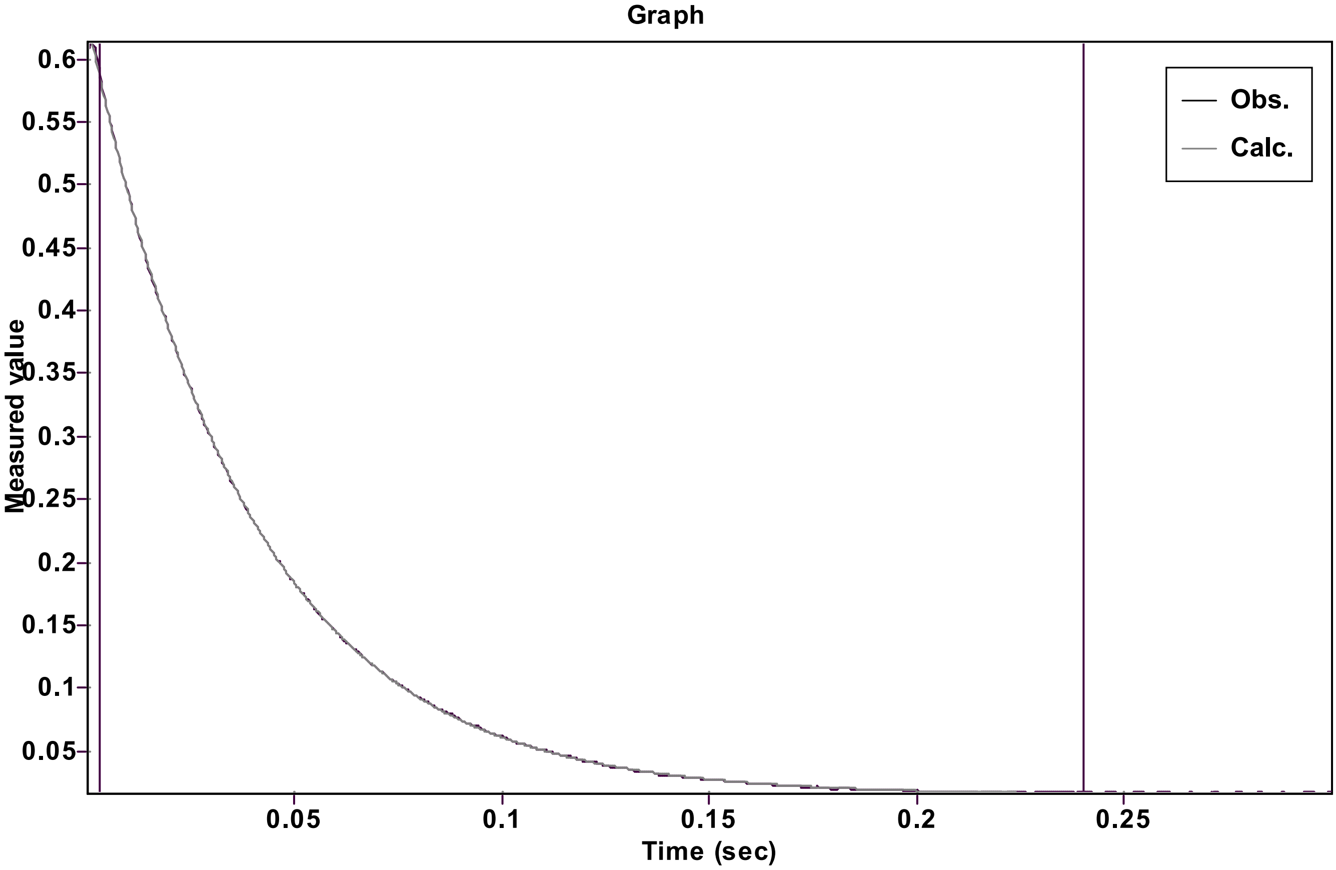

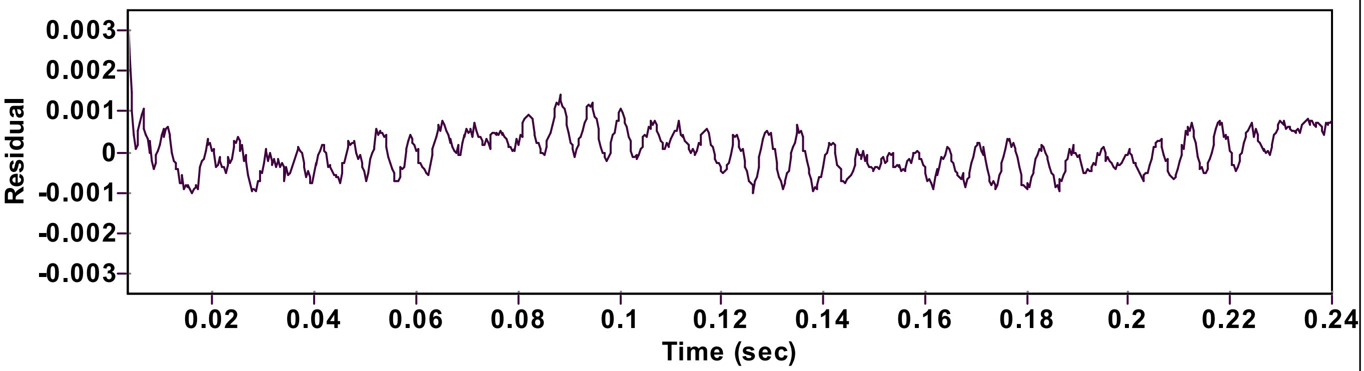

Function: y = A exp (-kx) + C (Exponential decrease)	Reference point: 0 (Zero)
Amp A = 0.620493693027799 ± 0.000105760563866<br>Rate k = 26.01870435101102 ± 0.007790333729756<br>Final C = 0.014988448035887 ± 0.000029607214363	Quality r^2 = 0.9999857123441<br>Data points = 790 of 1000<br>Conversion = 93.0 %
Start at position: 0.0033 / 0.58795 (4.2 %)	End at position: 0.24 / 0.0169818 (97.2 %)
ExpoFit file: 3-isochro_crown_NaH_dma-QM_50eq.exp<br>Source file: 3-isochro_crown_NaH_dma-QM_50eq.txt<br>Type of source file: Universal ASCII - file data	Date of file: 17/04/2023 13:39:22<br>Date of file: 17/04/2023 11:32:32
2007 by Dr. Kempf	Date of print: 17/04/2023 13:39:30
