# Supplementary material for: Lactone Enolates of Isochroman-3-ones and 2-Coumaranones: Quantification of Their Nucleophilicity in DMSO and Conjugate Additions to Chalcones
Source: J Org Chem. 2024 Apr 30;89(10):6915–28. doi: 10.1021/acs.joc.4c00277 (PMC11110064; doi:10.1021/acs.joc.4c00277)
Supplement: Supplementary file 2 — jo4c00277_si_002.zip [file jo4c00277_si_002.zip › 4+6d 3-isochro-15-crown-5_NaH_OMe-OMe/3-isochro-_NaH_OMe-OMe_20eq.pdf]

Evaluation of kinetic data with ExpoFit V 1.3

Graph

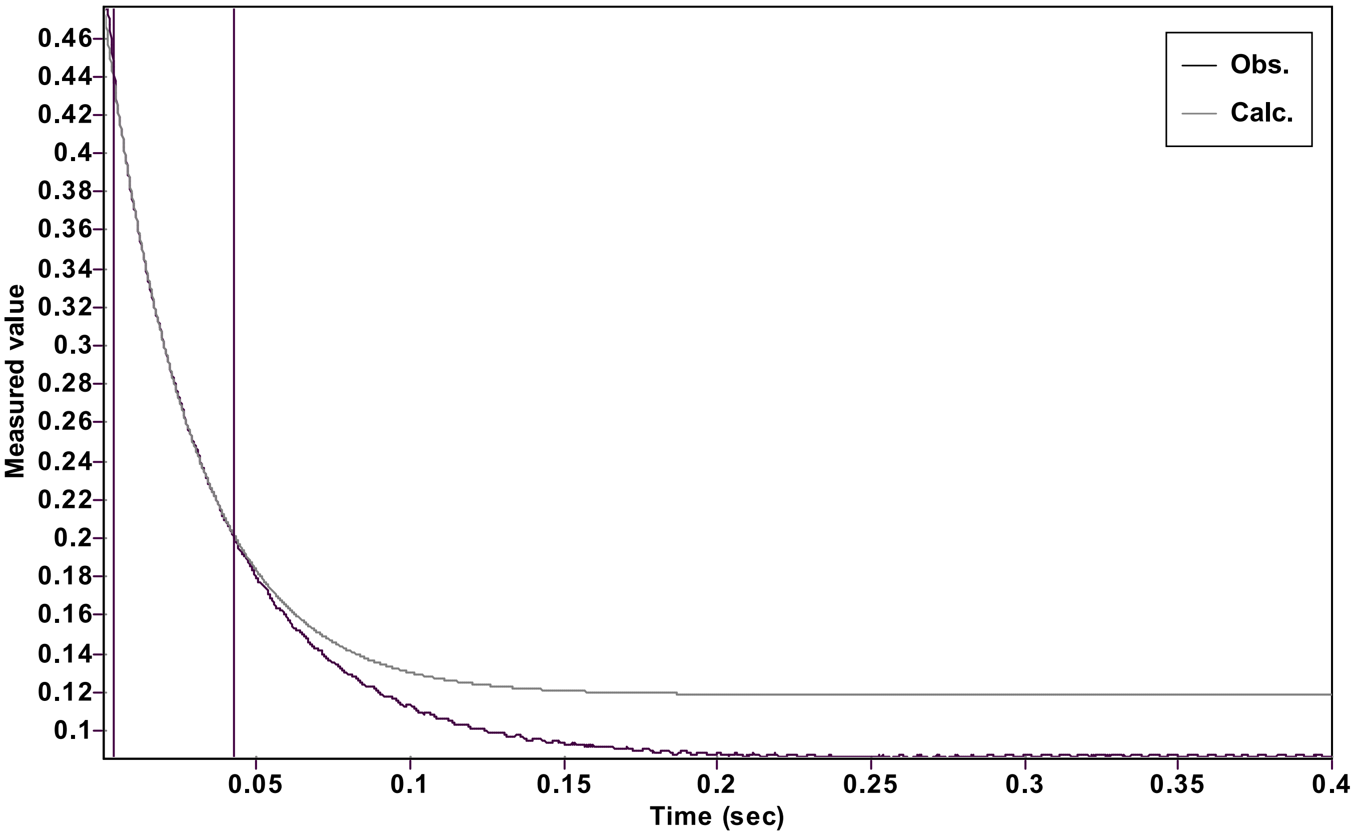

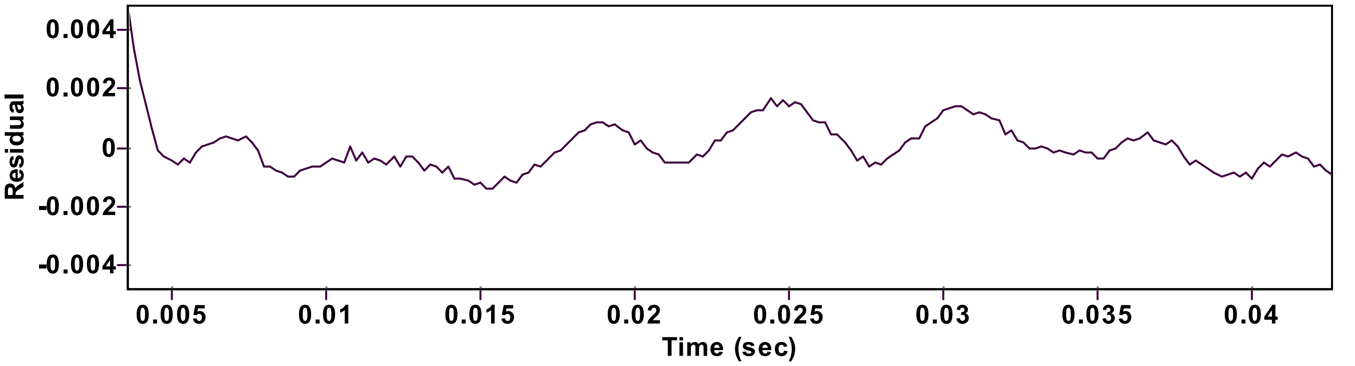

**Function:** y = A exp (-kx) + C (Exponential decrease)	**Reference point:** 0 (Zero)
**Amp A =** 0.359482633368994 ± 0.000700202222955	**Quality** r^2 = 0.9998469153220
**Rate k =** 34.38372903978978 ± 0.183745579580250	**Data points** = 196 of 2000
**Final C =** 0.118579593728378 ± 0.000923806073531	**Conversion** = 50.4 %
**Start at position:** 0.0036 / 0.440999 (7.4 %)	**End at position:** 0.0426 / 0.200743 (57.8 %)
**ExpoFit file:** File not saved	**Date of file:** Not available
**Source file:** 3-isochro_NaH_OMe-OMe_20eq.txt	**Date of file:** 12/04/2023 14:03:32
**Type of source file:** Universal ASCII - file data	
2007 by Dr. Kempf	**Date of print:** 12/04/2023 14:36:24
