# Supplementary material for: Lactone Enolates of Isochroman-3-ones and 2-Coumaranones: Quantification of Their Nucleophilicity in DMSO and Conjugate Additions to Chalcones
Source: J Org Chem. 2024 Apr 30;89(10):6915–28. doi: 10.1021/acs.joc.4c00277 (PMC11110064; doi:10.1021/acs.joc.4c00277)
Supplement: Supplementary file 2 — jo4c00277_si_002.zip [file jo4c00277_si_002.zip › 4+6d 3-isochro-15-crown-5_NaH_OMe-OMe/3-isochro-_NaH_OMe-OMe_40eq.pdf]

# Evaluation of kinetic data with ExpoFit V 1.3

Graph

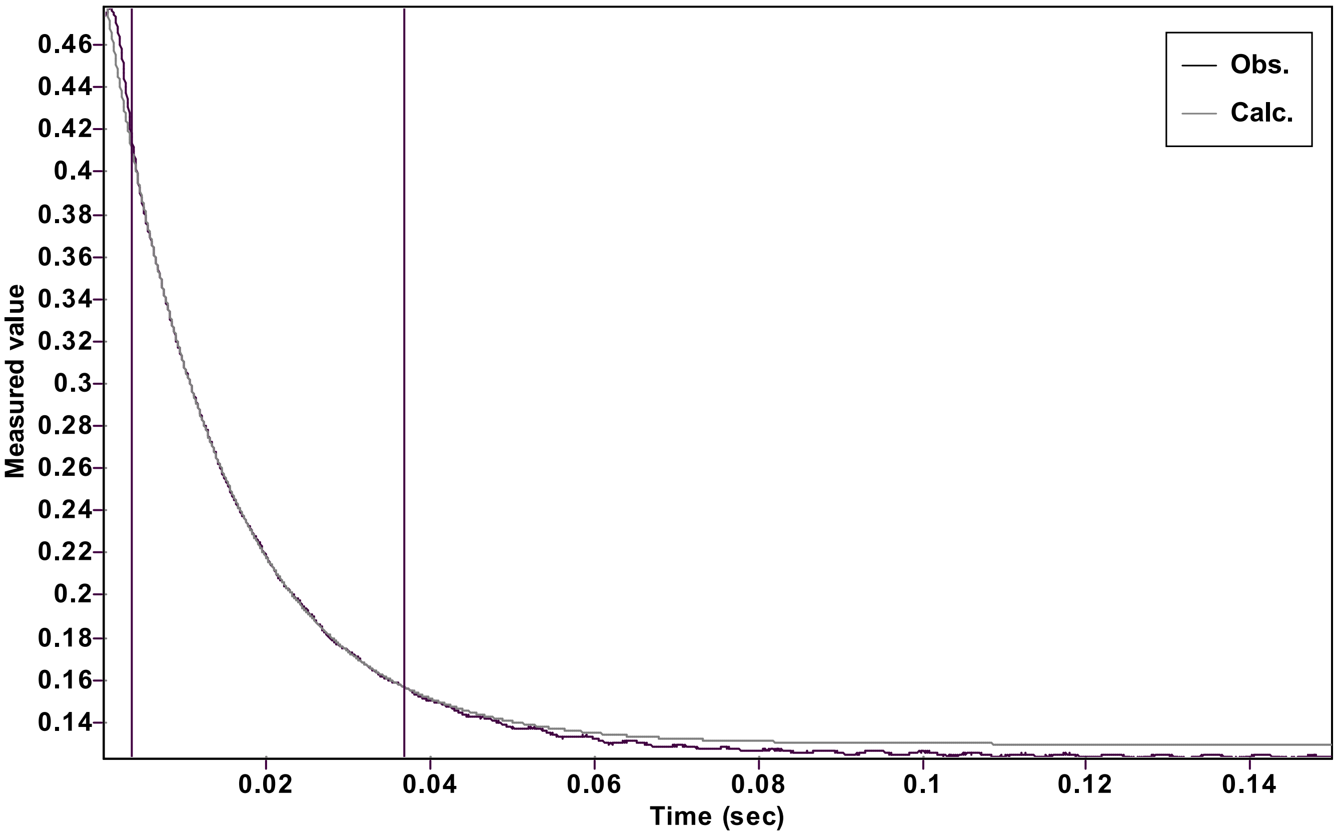


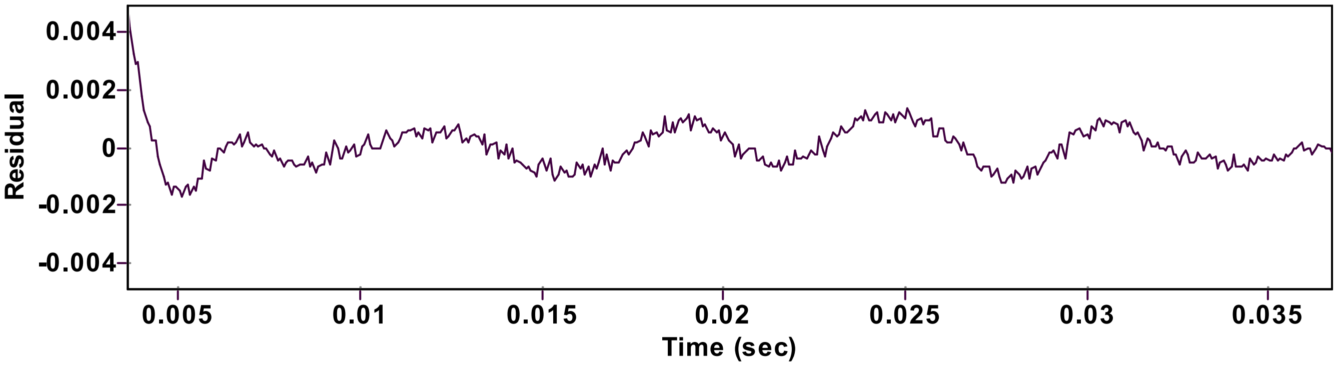


| | |
|---|---|
| Function: y = A exp ( -kx ) + C (Exponential decrease) | Reference point: 0 (Zero) |
| Amp A = 0.360479036154410 ± 0.000188335726162 | Quality $r^2$ = 0.9998898869790 |
| Rate k = 70.55536461796571 ± 0.131541867554399 | Data points = 443 of 2000 |
| Final C = 0.129609048100090 ± 0.000196707838683 | Conversion = 53.9 % |
| Start at position: 0.0036 / 0.414094 (13.4 %) | End at position: 0.03675 / 0.15642 (67.3 %) |
| ExpoFit file: 3-isochroNaH_OMe-OMe_40eq.exp | Date of file: 12/04/2023 14:39:56 |
| Source file: 3-isochroNaH_OMe-OMe_40eq.txt | Date of file: 12/04/2023 14:16:22 |
| Type of source file: Universal ASCII - file data | |
| 2007 by Dr. Kempf | Date of print: 12/04/2023 14:40:07 |
