# Supplementary material for: Lactone Enolates of Isochroman-3-ones and 2-Coumaranones: Quantification of Their Nucleophilicity in DMSO and Conjugate Additions to Chalcones
Source: J Org Chem. 2024 Apr 30;89(10):6915–28. doi: 10.1021/acs.joc.4c00277 (PMC11110064; doi:10.1021/acs.joc.4c00277)
Supplement: Supplementary file 2 — jo4c00277_si_002.zip [file jo4c00277_si_002.zip › 4+6d 3-isochro-15-crown-5_NaH_OMe-OMe/3-isochro-15-crown-5_NaH_OMe-OMe_10eq.pdf]

Evaluation of kinetic data with ExpoFit V 1.3

Graph

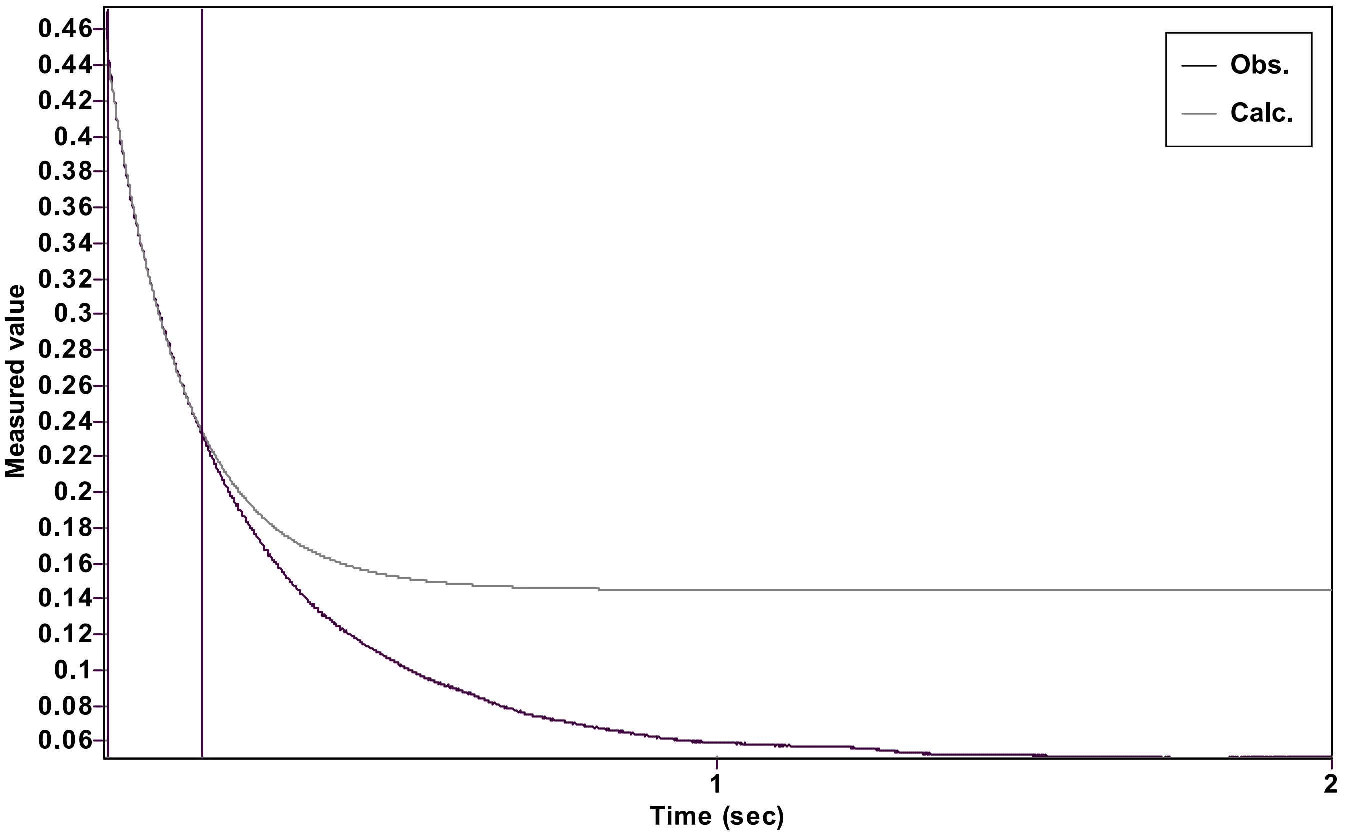

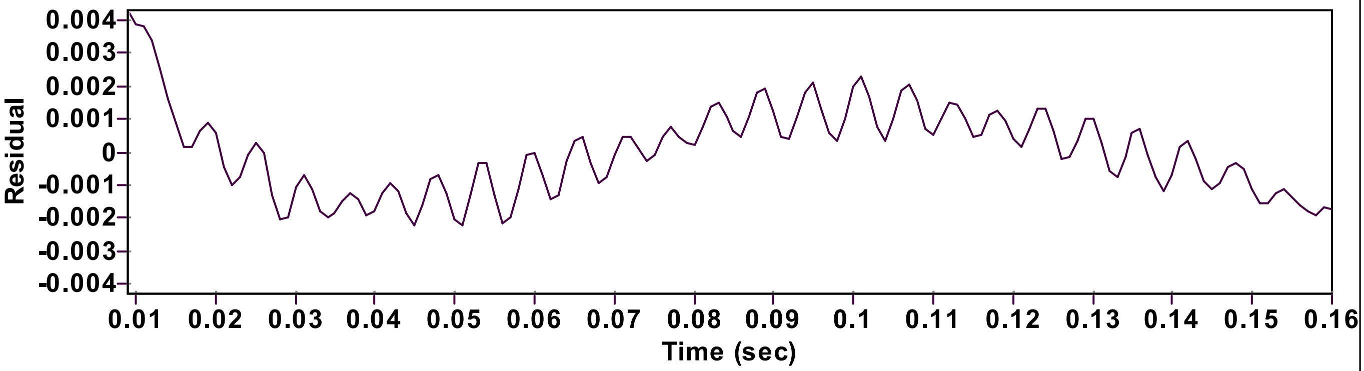

Function: y = A exp (-kx) + C (Exponential decrease)	Reference point: 0 (Zero)
Amp A = 0.316250522518921 ± 0.001753960438786	Quality r^2 = 0.9995067838691
Rate k = 7.858889983805184 ± 0.095874054893319	Data points = 152 of 2000
Final C = 0.144950154895289 ± 0.002085252459710	Conversion = 44.6 %
Start at position: 0.009 / 0.443892 (6.1 %)	End at position: 0.16 / 0.233174 (50.7 %)
ExpoFit file: File not saved	Date of file: Not available
Source file: 3-isochro_15-crown-5_NaH_OMeOMe_10eq.txt	Date of file: 12/04/2023 13:59:28
Type of source file: Universal ASCII - file data	
2007 by Dr. Kempf	Date of print: 12/04/2023 14:34:19
