# Supplementary material for: Lactone Enolates of Isochroman-3-ones and 2-Coumaranones: Quantification of Their Nucleophilicity in DMSO and Conjugate Additions to Chalcones
Source: J Org Chem. 2024 Apr 30;89(10):6915–28. doi: 10.1021/acs.joc.4c00277 (PMC11110064; doi:10.1021/acs.joc.4c00277)
Supplement: Supplementary file 2 — jo4c00277_si_002.zip [file jo4c00277_si_002.zip › 4+6d 3-isochro-15-crown-5_NaH_OMe-OMe/3-isochro-15-crown-5_NaH_OMe-OMe_30eq.pdf]

Evaluation of kinetic data with ExpoFit V 1.3

Graph

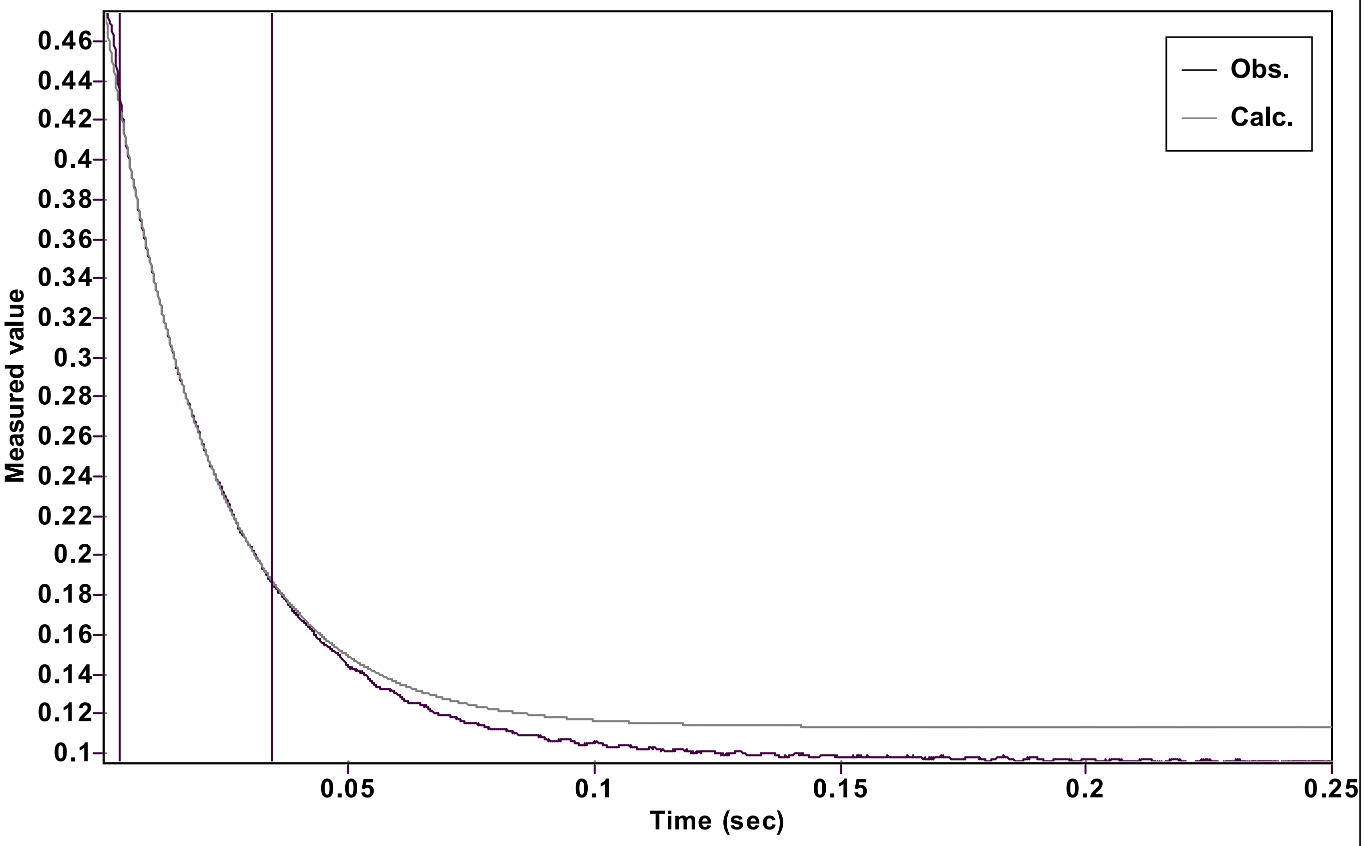

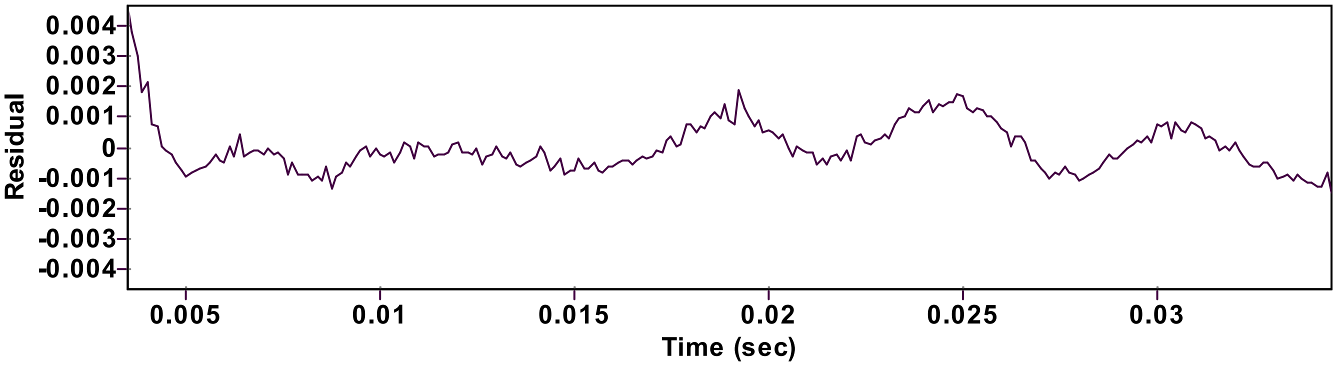

Function: y = A exp (-kx) + C (Exponential decrease)	Reference point: 0 (Zero)
Amp A = 0.367530304165870 ± 0.000487887333081	Quality r^2 = 0.9998544211223
Rate k = 46.62202001036060 ± 0.200978420763126	Data points = 249 of 2000
Final C = 0.113273217072665 ± 0.000702475046478	Conversion = 51.5 %
Start at position: 0.0035 / 0.430093 (9.5 %)	End at position: 0.0345 / 0.185382 (61.0 %)

ExpoFit file: 3-isochro_15-crown-5_NaH_OMe-OMe_30eq.exp Date of file: 12/04/2023 14:38:08

Source file: 3-isochro_15-crown-5_NaH_OMe-OMe_30eq.txt Date of file: 12/04/2023 14:11:54

Type of source file: Universal ASCII - file data

2007 by Dr. Kempf Date of print: 12/04/2023 14:38:19
