# Supplementary material for: Lactone Enolates of Isochroman-3-ones and 2-Coumaranones: Quantification of Their Nucleophilicity in DMSO and Conjugate Additions to Chalcones
Source: J Org Chem. 2024 Apr 30;89(10):6915–28. doi: 10.1021/acs.joc.4c00277 (PMC11110064; doi:10.1021/acs.joc.4c00277)
Supplement: Supplementary file 2 — jo4c00277_si_002.zip [file jo4c00277_si_002.zip › 5+6c coumaranone_dma-tBu/dma-tBu_10equicarbanion.pdf]

Evaluation of kinetic data with ExpoFit V 1.3

Graph

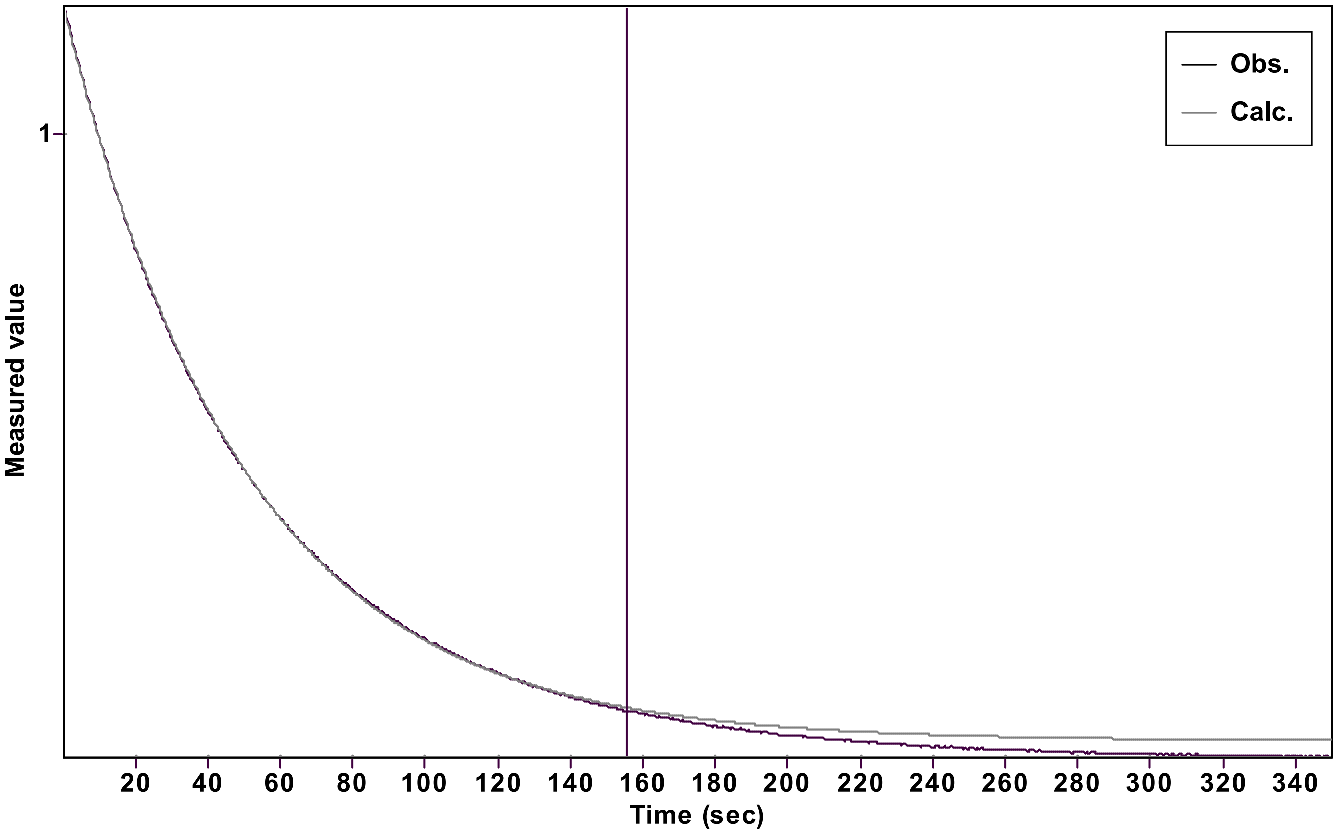

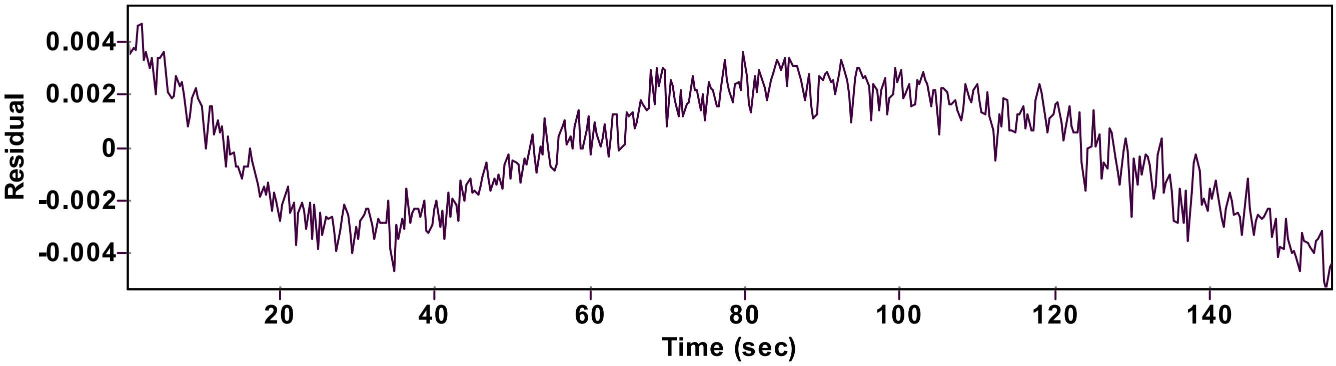

Function: y = A exp (-kx) + C (Exponential decrease)	Reference point: C (of function)

Amp A =	1.078657820804419	卤	0.000412296920655	Quality r^2 =	0.9999355602548
Rate k =	0.019944419844166	卤	0.000023159855589	Data points =	445 of 1000
Final C =	0.113531450787535	卤	0.000374564340881	Conversion =	95.9 %

Start at position: 0.35 / 1.18906 (0.0 %)	End at position: 155.75 / 0.157465 (95.9 %)

ExpoFit file: File not saved	Date of file: Not available
Source file: dma-tBu_10equicarbanion.txt	Date of file: 13/02/2023 14:18:14
Type of source file: Universal ASCII - file data	

2007 by Dr. Kempf	Date of print: 13/02/2023 15:02:37
