# Supplementary material for: Lactone Enolates of Isochroman-3-ones and 2-Coumaranones: Quantification of Their Nucleophilicity in DMSO and Conjugate Additions to Chalcones
Source: J Org Chem. 2024 Apr 30;89(10):6915–28. doi: 10.1021/acs.joc.4c00277 (PMC11110064; doi:10.1021/acs.joc.4c00277)
Supplement: Supplementary file 2 — jo4c00277_si_002.zip [file jo4c00277_si_002.zip › 5+6c coumaranone_dma-tBu/dma-tBu_20equicarbanion.pdf]

# Evaluation of kinetic data with ExpoFit V 1.3

Graph

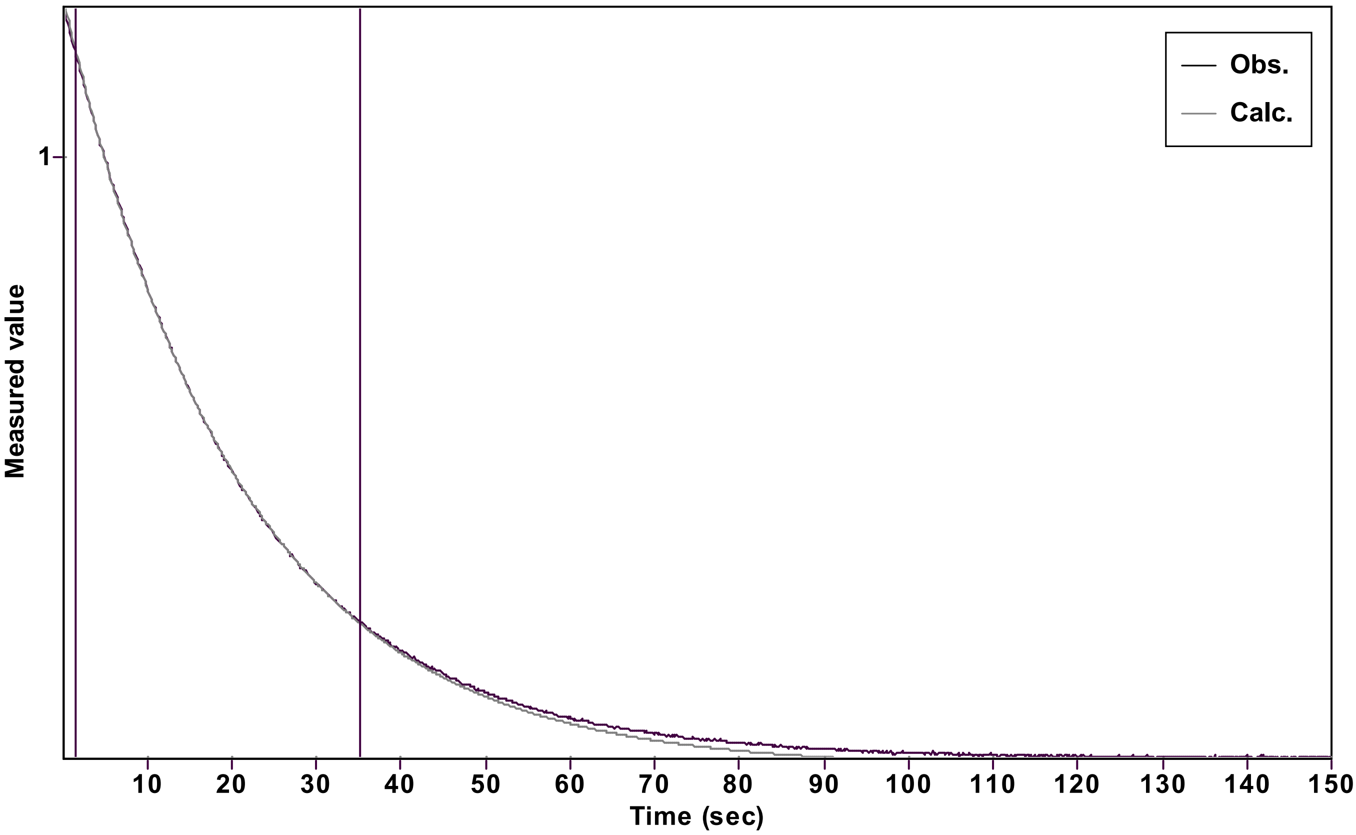


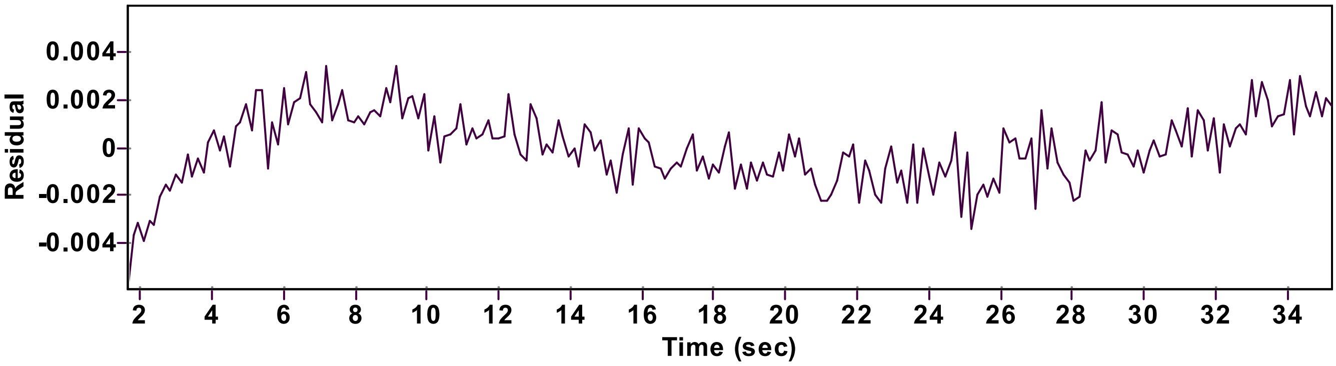


| Function: y = A exp ( -kx ) + C (Exponential decrease) | Reference point: C (of function) |
|---|---|
| Amp A = 1.108831840090872 鹵 0.000847500911236 | Quality $r^2$ = 0.9999596193552 |
| Rate k = 0.047711476363966 鹵 0.000104938515724 | Data points = 225 of 1000 |
| Final C = 0.124147784157702 鹵 0.001106003508416 | Conversion = 74.3 % |

Start at position: 1.65 / 1.14305 (6.6 %)

End at position: 35.25 / 0.332175 (80.9 %)

ExpoFit file: File not saved — Date of file: Not available

Source file: dma-tBu_20equicarbanion.txt — Date of file: 13/02/2023 14:30:40

Type of source file: Universal ASCII - file data

2007 by Dr. Kempf

Date of print: 13/02/2023 15:06:18
