# Supplementary material for: Lactone Enolates of Isochroman-3-ones and 2-Coumaranones: Quantification of Their Nucleophilicity in DMSO and Conjugate Additions to Chalcones
Source: J Org Chem. 2024 Apr 30;89(10):6915–28. doi: 10.1021/acs.joc.4c00277 (PMC11110064; doi:10.1021/acs.joc.4c00277)
Supplement: Supplementary file 2 — jo4c00277_si_002.zip [file jo4c00277_si_002.zip › 5+6c coumaranone_dma-tBu/dma-tBu_30equicarbanion.pdf]

Evaluation of kinetic data with ExpoFit V 1.3

Graph

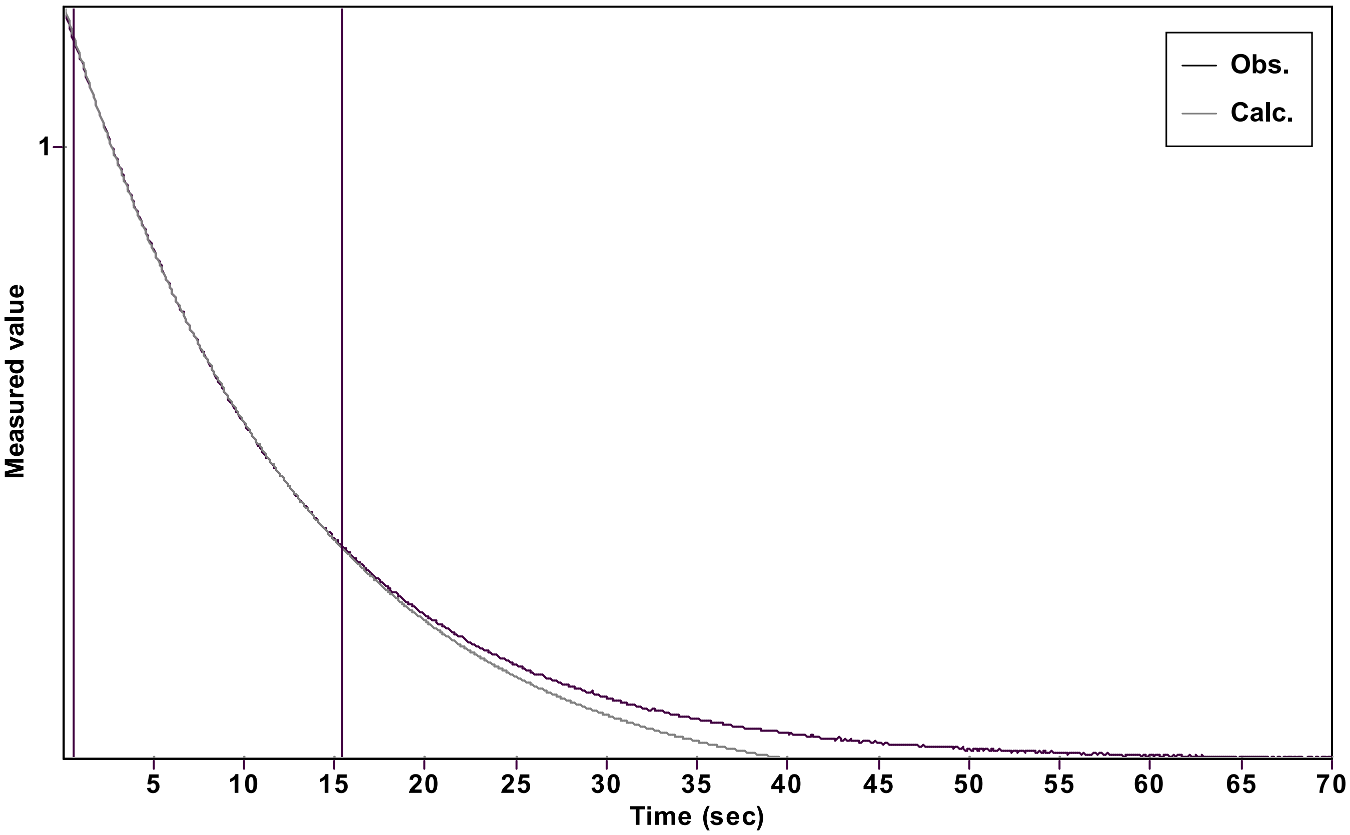

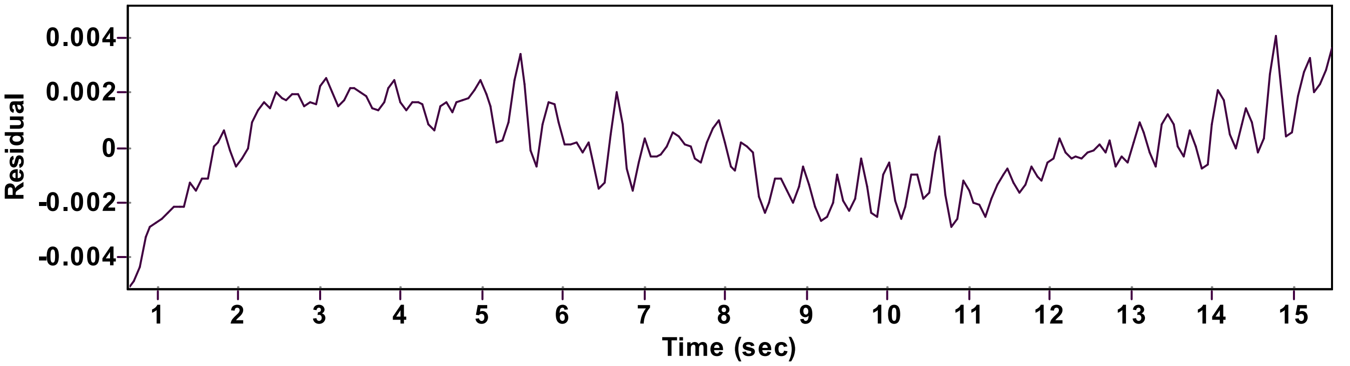

**Function:** y = A exp (-kx) + C (Exponential decrease)	**Reference point:** C (of function)
**Amp A =** 1.159277789908422 卤 0.002170920361159<br>**Rate k =** 0.075075796419399 卤 0.000282300929749<br>**Final C =** 0.053830303016421 卤 0.002485457190447	**Quality** r^2 = 0.9999414690373<br>**Data points** = 213 of 1000<br>**Conversion** = 63.9 %
**Start at position:** 0.63 / 1.15439 (4.2 %)	**End at position:** 15.47 / 0.420368 (68.1 %)
**ExpoFit file:** File not saved<br>**Source file:** dma-tBu_30equicarbanion.txt<br>**Type of source file:** Universal ASCII - file data	**Date of file:** Not available<br>**Date of file:** 13/02/2023 14:44:18
2007 by Dr. Kempf	**Date of print:** 13/02/2023 15:09:15
