# Supplementary material for: Lactone Enolates of Isochroman-3-ones and 2-Coumaranones: Quantification of Their Nucleophilicity in DMSO and Conjugate Additions to Chalcones
Source: J Org Chem. 2024 Apr 30;89(10):6915–28. doi: 10.1021/acs.joc.4c00277 (PMC11110064; doi:10.1021/acs.joc.4c00277)
Supplement: Supplementary file 2 — jo4c00277_si_002.zip [file jo4c00277_si_002.zip › 5+6c coumaranone_dma-tBu/dma-tBu_40equicarbanion.pdf]

Evaluation of kinetic data with ExpoFit V 1.3

Graph

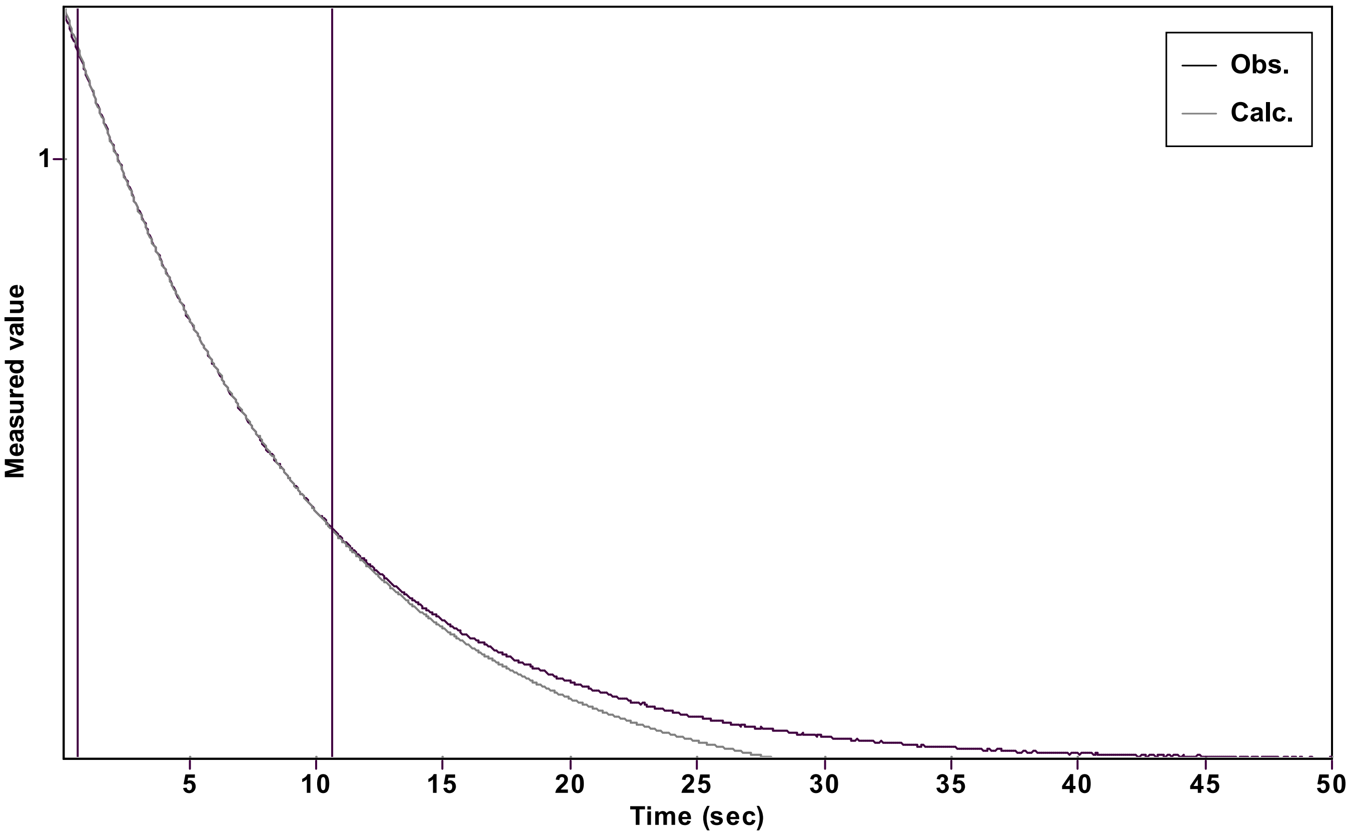

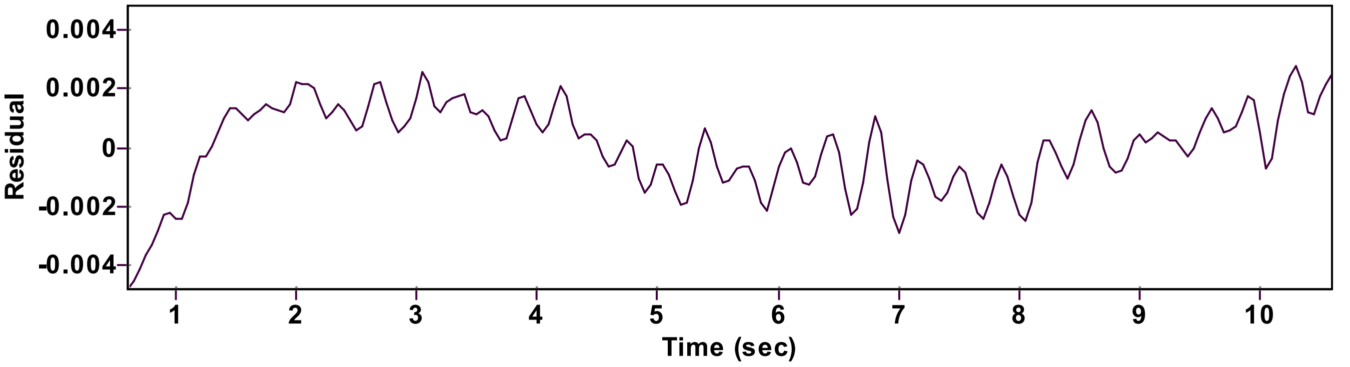

Function: y = A exp (-kx) + C (Exponential decrease)	Reference point: C (of function)
Amp A = 1.178008029830896 卤 0.002462183705657	Quality r^2 = 0.9999507494866
Rate k = 0.100736827210340 卤 0.000405511571887	Data points = 201 of 1000
Final C = 0.054373404758686 卤 0.002792765567852	Conversion = 59.6 %

Start at position: 0.6 / 1.15844 (5.5 %)

End at position: 10.6 / 0.461805 (65.1 %)

ExpoFit file: File not saved

Date of file: Not available

Source file: dma-tBu_40equicarbanion.txt

Date of file: 13/02/2023 14:52:12

Type of source file: Universal ASCII - file data

2007 by Dr. Kempf

Date of print: 13/02/2023 15:10:59
