# Supplementary material for: Lactone Enolates of Isochroman-3-ones and 2-Coumaranones: Quantification of Their Nucleophilicity in DMSO and Conjugate Additions to Chalcones
Source: J Org Chem. 2024 Apr 30;89(10):6915–28. doi: 10.1021/acs.joc.4c00277 (PMC11110064; doi:10.1021/acs.joc.4c00277)
Supplement: Supplementary file 2 — jo4c00277_si_002.zip [file jo4c00277_si_002.zip › 5+6e coumaranone_OMe-tBu/OMe-tBu_10equicarbanion.pdf]

Evaluation of kinetic data with ExpoFit V 1.3

Graph

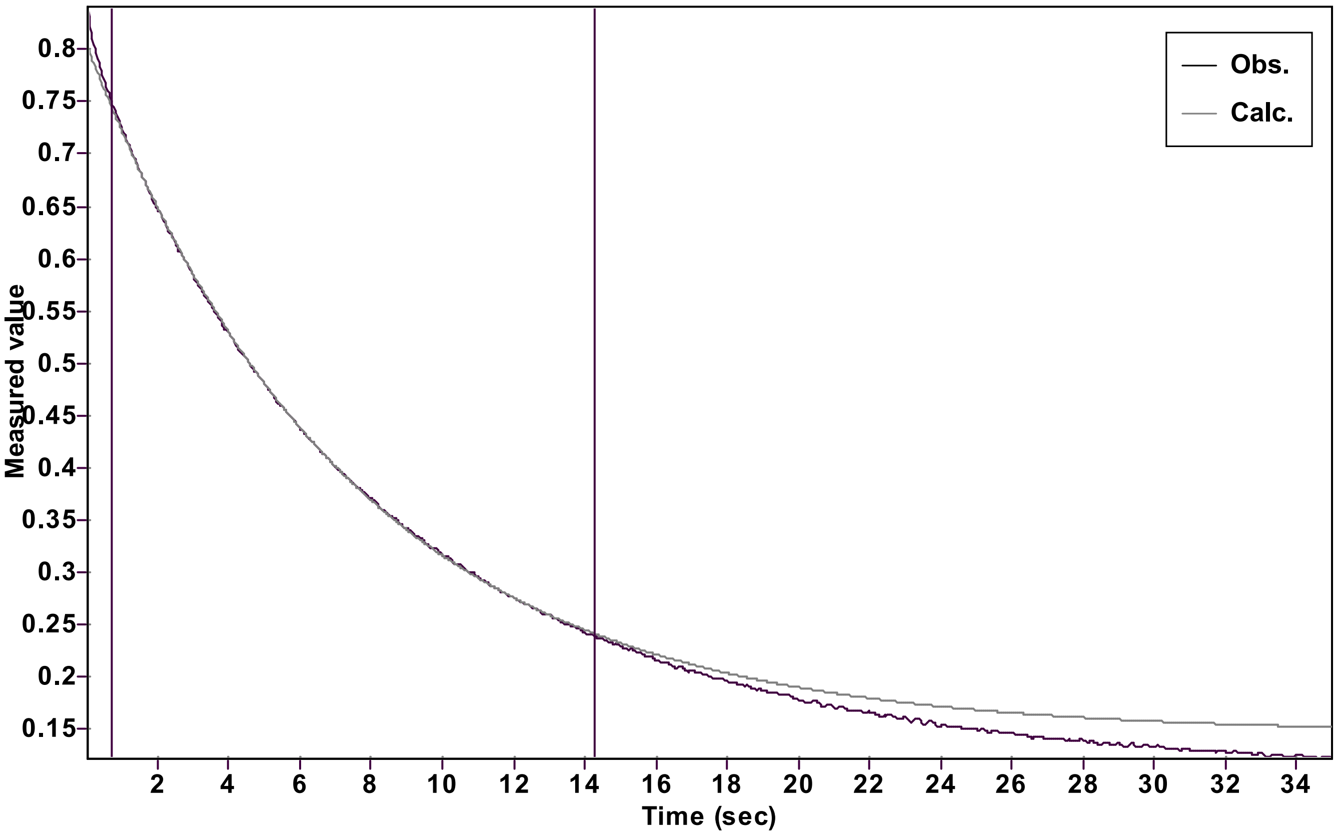

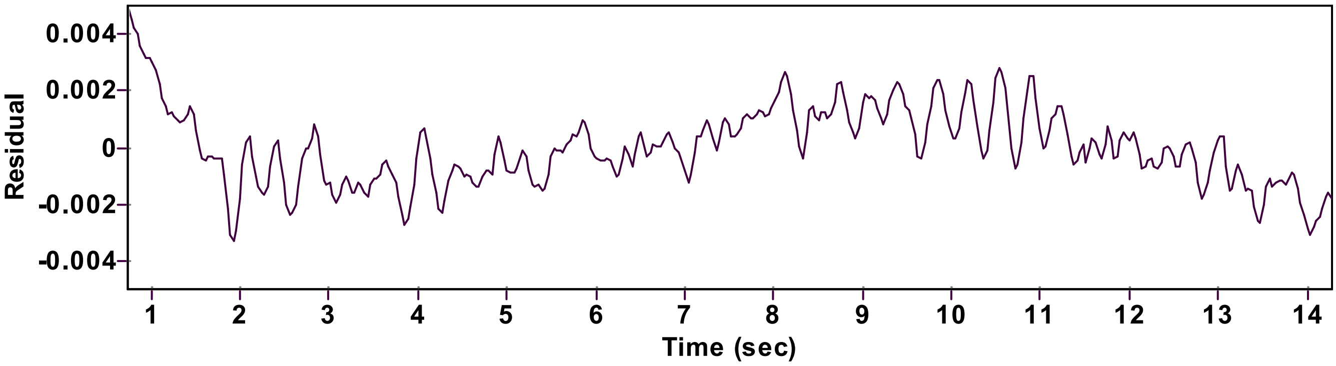

Function: y = A exp (-kx) + C (Exponential decrease)	Reference point: C (of function)
Amp A = 0.659702079994888 卤 0.000435783051859	Quality r^2 = 0.9999070690012
Rate k = 0.135664808817261 卤 0.000302237126306	Data points = 388 of 1000
Final C = 0.145818773113396 卤 0.000597447115663	Conversion = 73.2 %
Start at position: 0.735 / 0.747883 (13.4 %)	End at position: 14.28 / 0.239063 (86.6 %)
ExpoFit file: OMe-tBu_10equicarbanion.exp	Date of file: 10/02/2023 17:54:50
Source file: OMe-tBu_10equicarbanion.txt	Date of file: 10/02/2023 16:09:12
Type of source file: Universal ASCII - file data	
2007 by Dr. Kempf	Date of print: 10/02/2023 17:56:45
