# Supplementary material for: Lactone Enolates of Isochroman-3-ones and 2-Coumaranones: Quantification of Their Nucleophilicity in DMSO and Conjugate Additions to Chalcones
Source: J Org Chem. 2024 Apr 30;89(10):6915–28. doi: 10.1021/acs.joc.4c00277 (PMC11110064; doi:10.1021/acs.joc.4c00277)
Supplement: Supplementary file 2 — jo4c00277_si_002.zip [file jo4c00277_si_002.zip › 5+6e coumaranone_OMe-tBu/OMe-tBu_20equicarbanion.pdf]

Evaluation of kinetic data with ExpoFit V 1.3

Graph

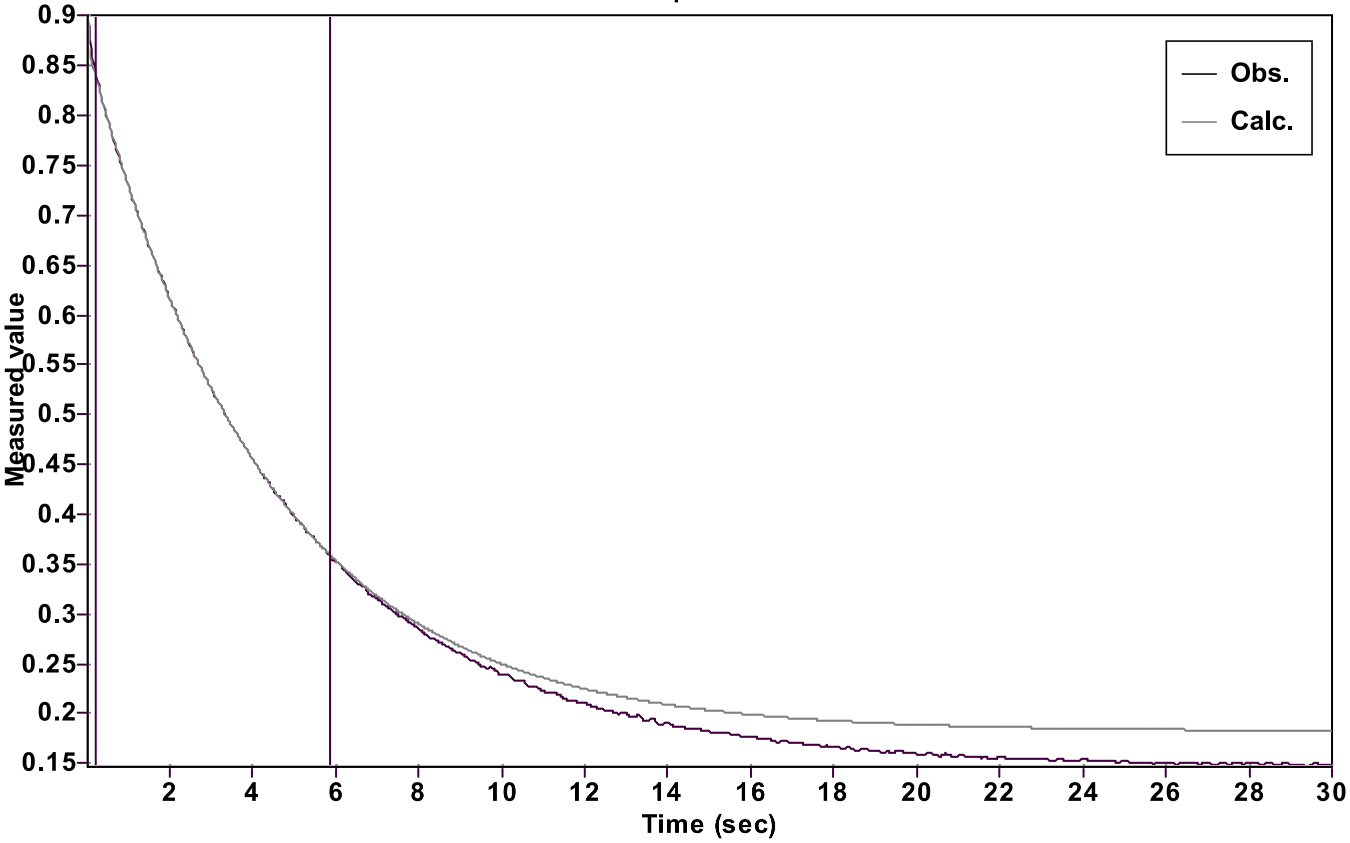

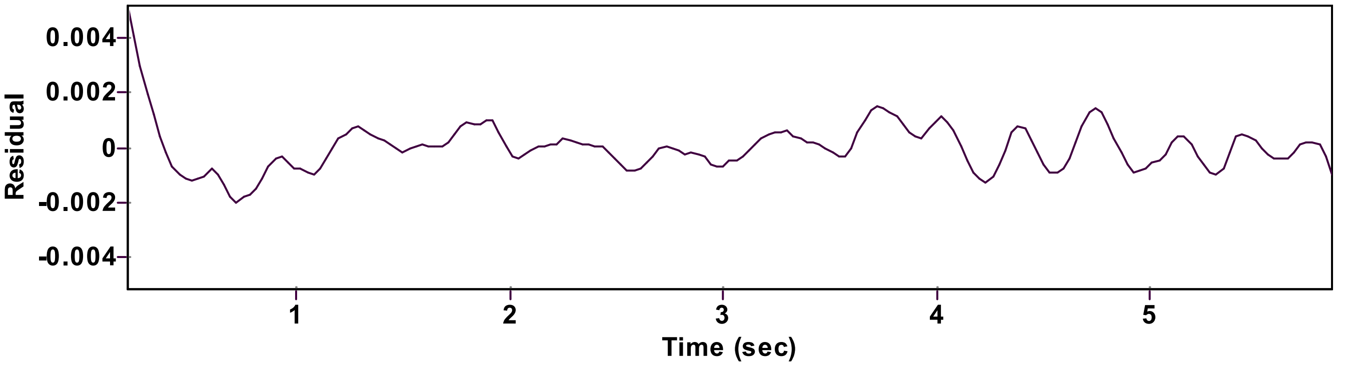

Function: y = A exp (-kx) + C (Exponential decrease)	Reference point: C (of function)
Amp A = 0.691813198837688 卤 0.000881127865310	Quality r^2 = 0.9999594366884
Rate k = 0.232578816306725 卤 0.000672919222232	Data points = 189 of 1000
Final C = 0.182442874049817 卤 0.001049955136494	Conversion = 67.9 %
Start at position: 0.21 / 0.846497 (7.5 %)	End at position: 5.85 / 0.358926 (75.4 %)
ExpoFit file: File not saved	Date of file: Not available
Source file: OMe-tBu_20equicarbanion.txt	Date of file: 10/02/2023 16:22:36
Type of source file: Universal ASCII - file data	
2007 by Dr. Kempf	Date of print: 10/02/2023 17:58:28
