# Supplementary material for: Lactone Enolates of Isochroman-3-ones and 2-Coumaranones: Quantification of Their Nucleophilicity in DMSO and Conjugate Additions to Chalcones
Source: J Org Chem. 2024 Apr 30;89(10):6915–28. doi: 10.1021/acs.joc.4c00277 (PMC11110064; doi:10.1021/acs.joc.4c00277)
Supplement: Supplementary file 2 — jo4c00277_si_002.zip [file jo4c00277_si_002.zip › 5+6e coumaranone_OMe-tBu/OMe-tBu_30equicarbanion.pdf]

Evaluation of kinetic data with ExpoFit V 1.3

Graph

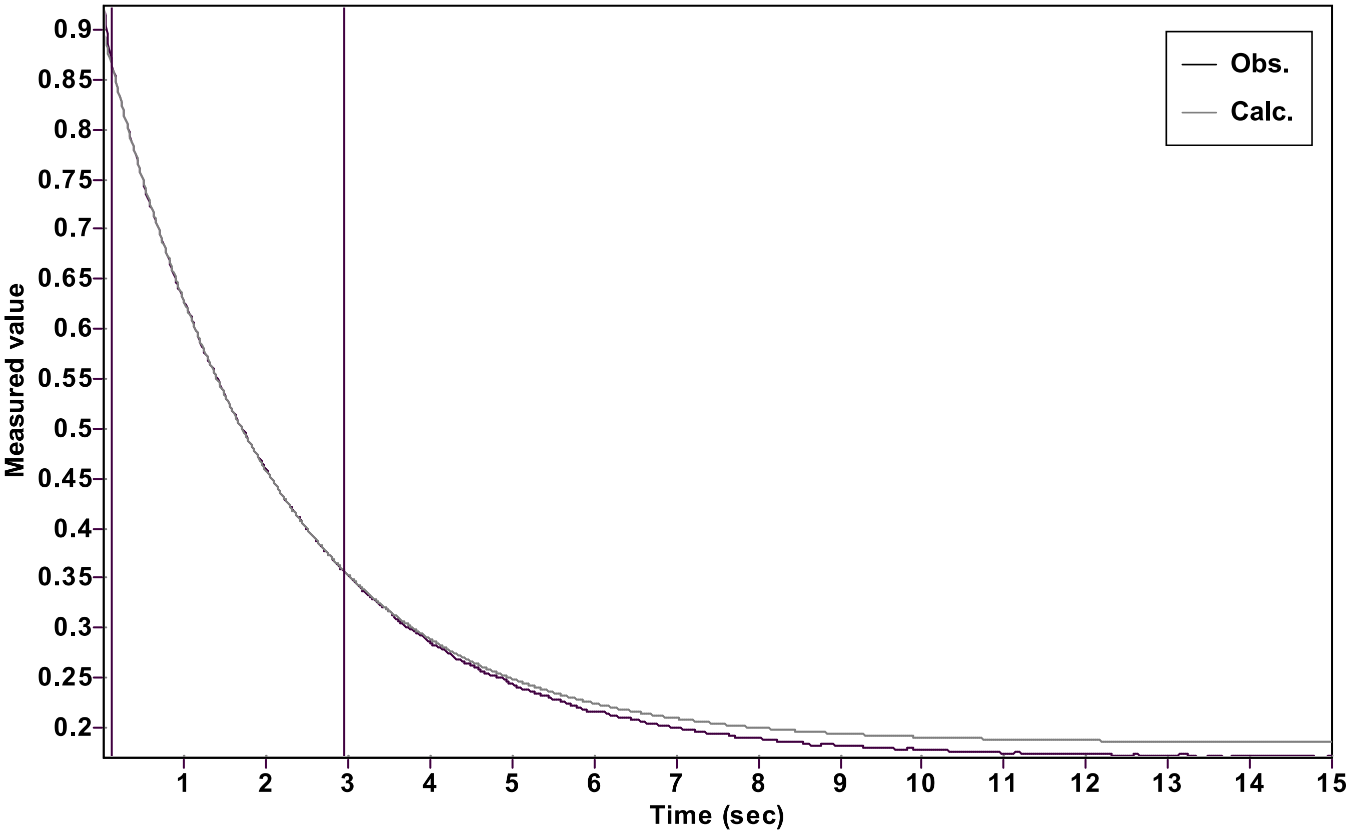

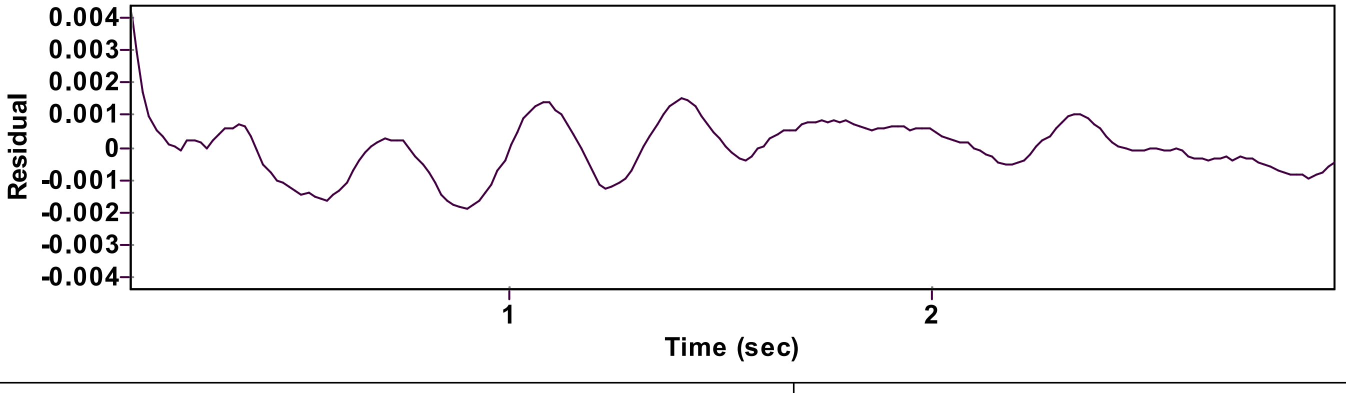

Function: y = A exp (-kx) + C (Exponential decrease)	Reference point: C (of function)
Amp A = 0.717183166731703 卤 0.000770414736082<br>Rate k = 0.483197193232202 卤 0.001231173846075<br>Final C = 0.184834739179832 卤 0.000931650505328	Quality r^2 = 0.9999647882200<br>Data points = 191 of 1000<br>Conversion = 69.6 %
Start at position: 0.105 / 0.870923 (7.2 %)	End at position: 2.955 / 0.356369 (76.8 %)
ExpoFit file: OMe-tBu_30equicarbanion.exp	Date of file: 10/02/2023 18:00:18
Source file: OMe-tBu_30equicarbanion.txt	Date of file: 10/02/2023 16:30:32
Type of source file: Universal ASCII - file data	
2007 by Dr. Kempf	Date of print: 10/02/2023 18:04:33
