# Supplementary material for: Lactone Enolates of Isochroman-3-ones and 2-Coumaranones: Quantification of Their Nucleophilicity in DMSO and Conjugate Additions to Chalcones
Source: J Org Chem. 2024 Apr 30;89(10):6915–28. doi: 10.1021/acs.joc.4c00277 (PMC11110064; doi:10.1021/acs.joc.4c00277)
Supplement: Supplementary file 2 — jo4c00277_si_002.zip [file jo4c00277_si_002.zip › 5+6e coumaranone_OMe-tBu/OMe-tBu_40equicarbanion.pdf]

Evaluation of kinetic data with ExpoFit V 1.3

Graph

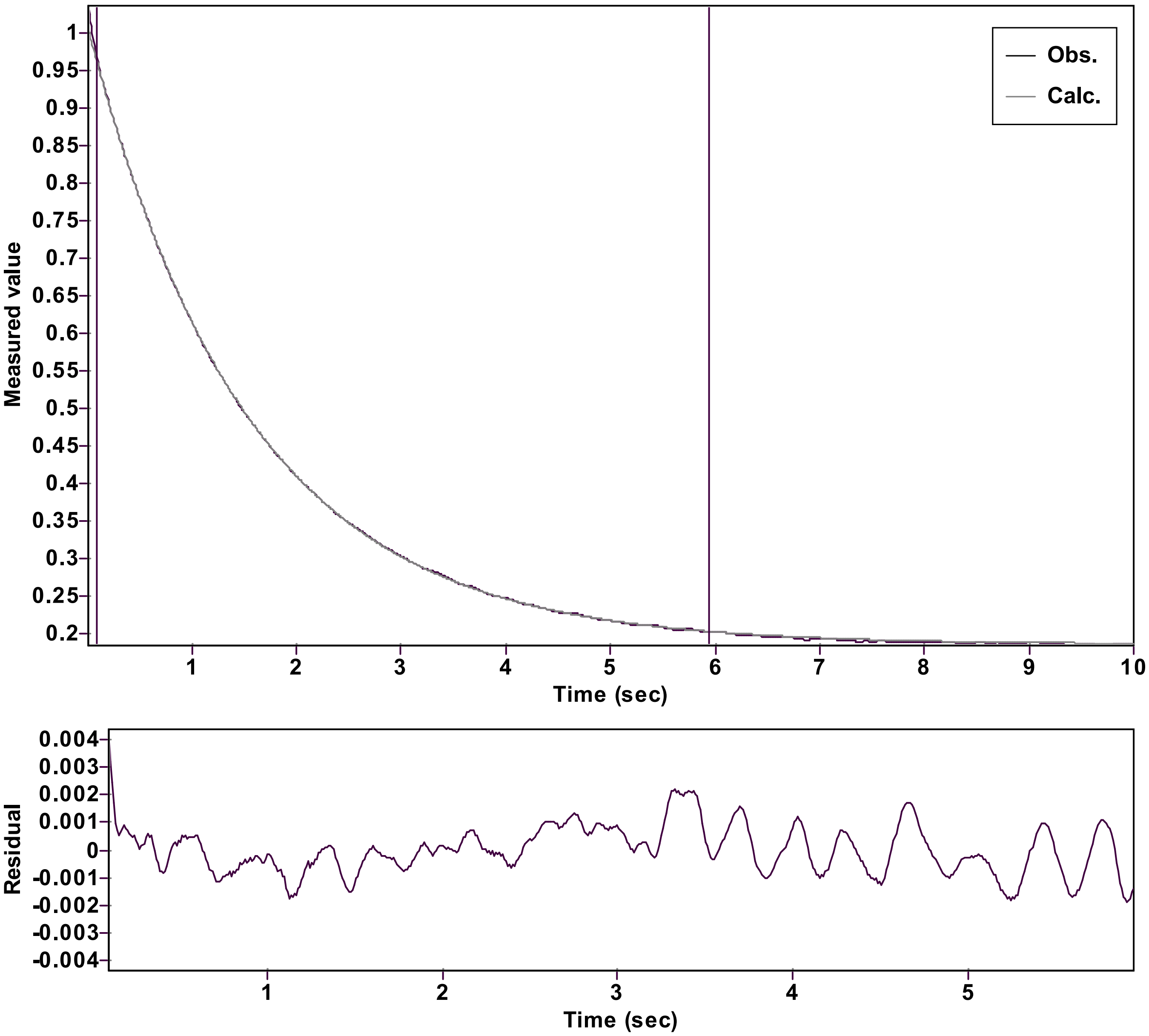

Function: y = A exp (-kx) + C (Exponential decrease)	Reference point: C (of function)
Amp A = 0.825830313746491 卤 0.000161254202471	Quality r^2 = 0.9999803616322
Rate k = 0.651672696535683 卤 0.000321086886034	Data points = 587 of 1000
Final C = 0.184635574715559 卤 0.000097044003190	Conversion = 90.1 %

Start at position: 0.09 / 0.967789 (8.1 %) | End at position: 5.95 / 0.200358 (98.2 %)

ExpoFit file: OMe-tBu_40equicarbanion1.exp — Date of file: 10/02/2023 18:02:14

Source file: OMe-tBu_40equicarbanion1.txt — Date of file: 10/02/2023 16:38:04

Type of source file: Universal ASCII - file data

2007 by Dr. Kempf Date of print: 10/02/2023 18:05:46
