# Supplementary material for: Lactone Enolates of Isochroman-3-ones and 2-Coumaranones: Quantification of Their Nucleophilicity in DMSO and Conjugate Additions to Chalcones
Source: J Org Chem. 2024 Apr 30;89(10):6915–28. doi: 10.1021/acs.joc.4c00277 (PMC11110064; doi:10.1021/acs.joc.4c00277)
Supplement: Supplementary file 2 — jo4c00277_si_002.zip [file jo4c00277_si_002.zip › 5+6f coumaranone_mF-tBu/10equiv-CH-Acid-mF-tBu-370nm.pdf]

Evaluation of kinetic data with ExpoFit V 1.3

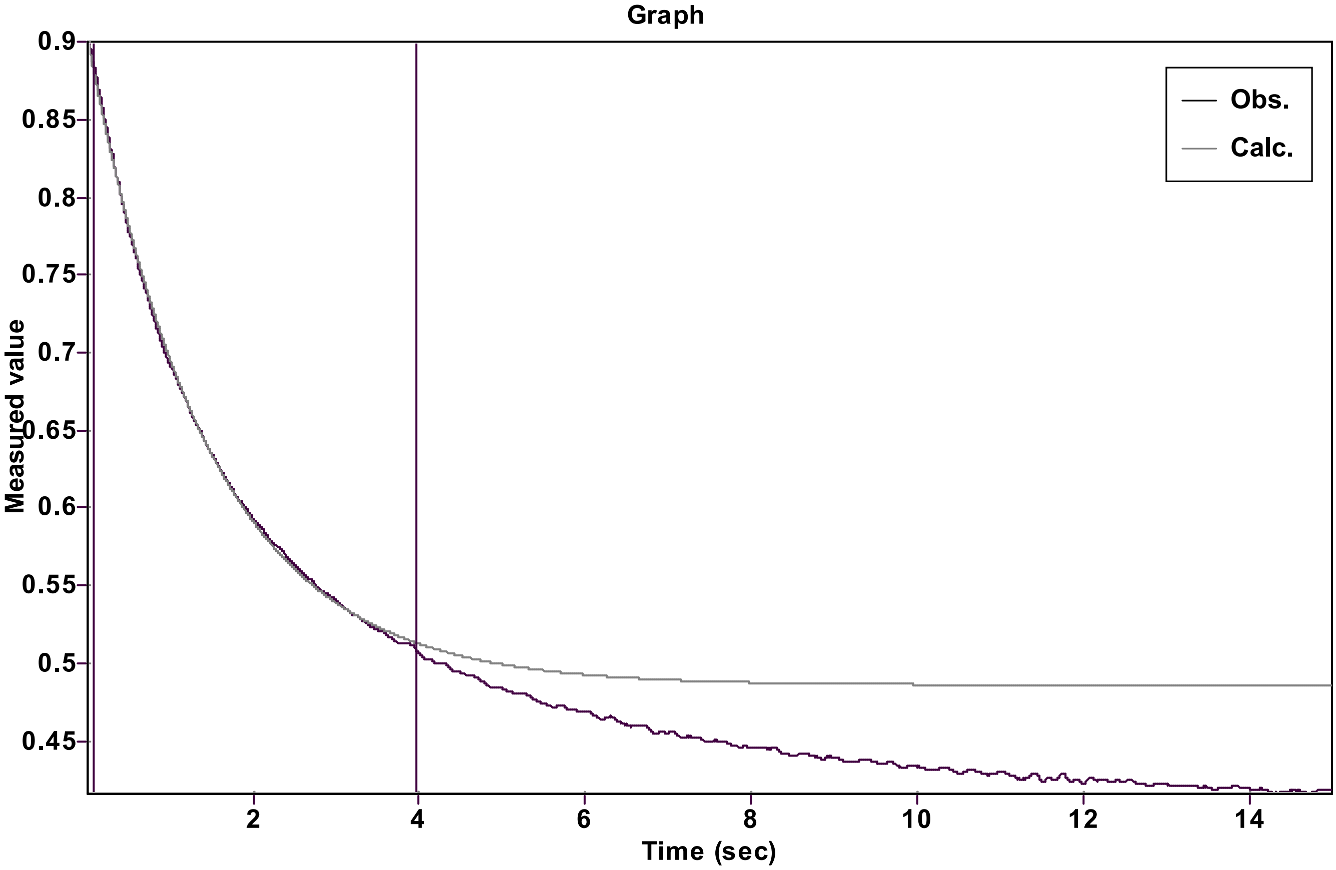

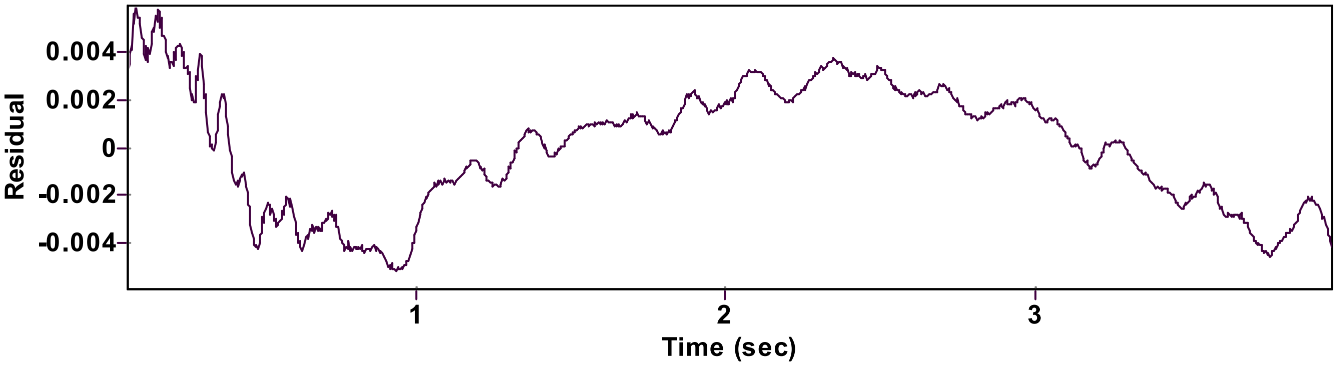

Function: y = A exp (-kx) + C (Exponential decrease)	Reference point: C (of function)
Amp A = 0.417193391552241 卤 0.000334844379580	Quality r^2 = 0.9993546425030
Rate k = 0.688361308187028 卤 0.001834556981077	Data points = 1037 of 4000
Final C = 0.485806493615878 卤 0.000359369702531	Conversion = 91.0 %
Start at position: 0.07125 / 0.886193 (3.4 %)	End at position: 3.95625 / 0.509018 (94.4 %)
ExpoFit file: 10equiv-CH-Acid-mF-tBu-370nm.exp	Date of file: 08/02/2023 16:13:44
Source file: 10equiv-CH-Acid-mF-tBu-370nm.txt	Date of file: 08/02/2023 15:25:32
Type of source file: Universal ASCII - file data	
2007 by Dr. Kempf	Date of print: 10/02/2023 17:29:00
