# Supplementary material for: Lactone Enolates of Isochroman-3-ones and 2-Coumaranones: Quantification of Their Nucleophilicity in DMSO and Conjugate Additions to Chalcones
Source: J Org Chem. 2024 Apr 30;89(10):6915–28. doi: 10.1021/acs.joc.4c00277 (PMC11110064; doi:10.1021/acs.joc.4c00277)
Supplement: Supplementary file 2 — jo4c00277_si_002.zip [file jo4c00277_si_002.zip › 5+6f coumaranone_mF-tBu/20equiv-CH-Acid-mF-tBu-370nm.pdf]

Evaluation of kinetic data with ExpoFit V 1.3

Graph

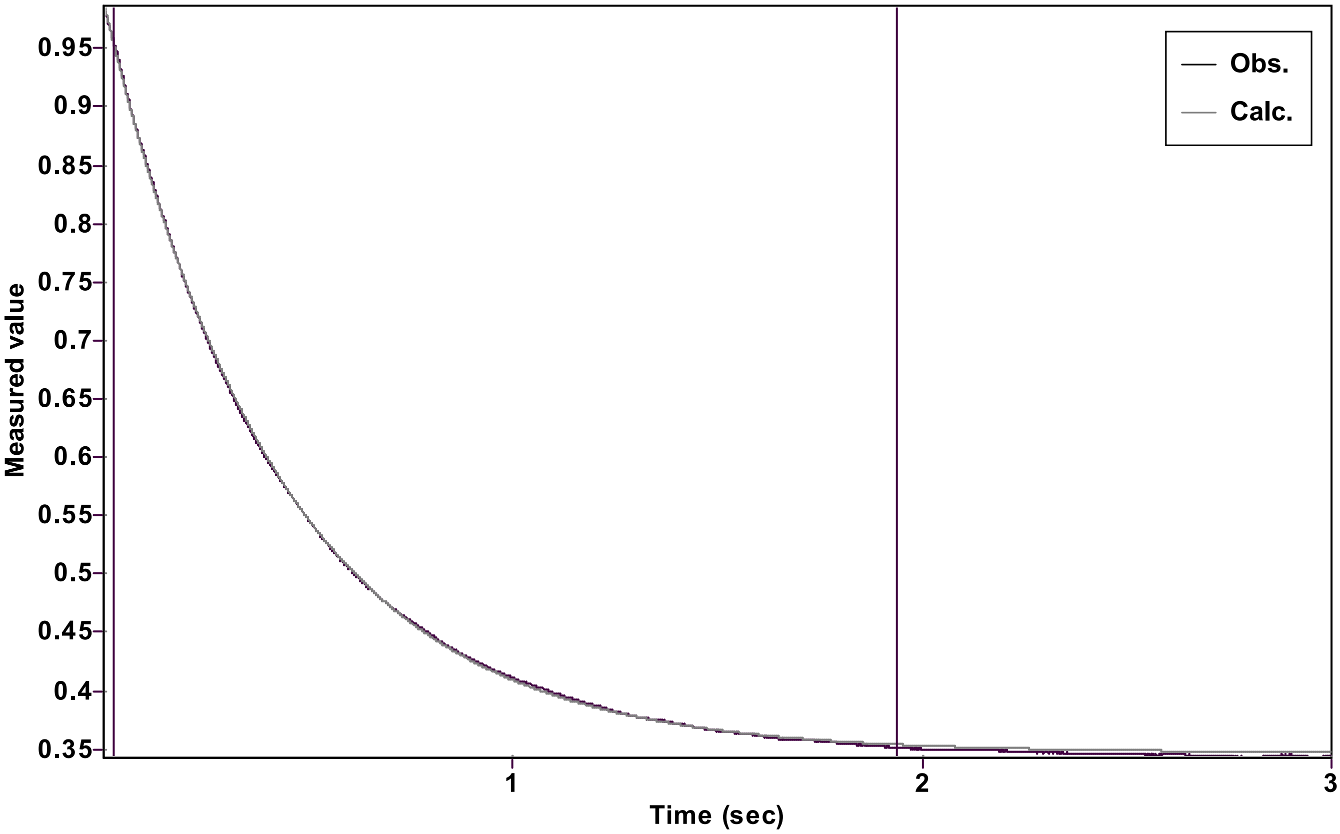

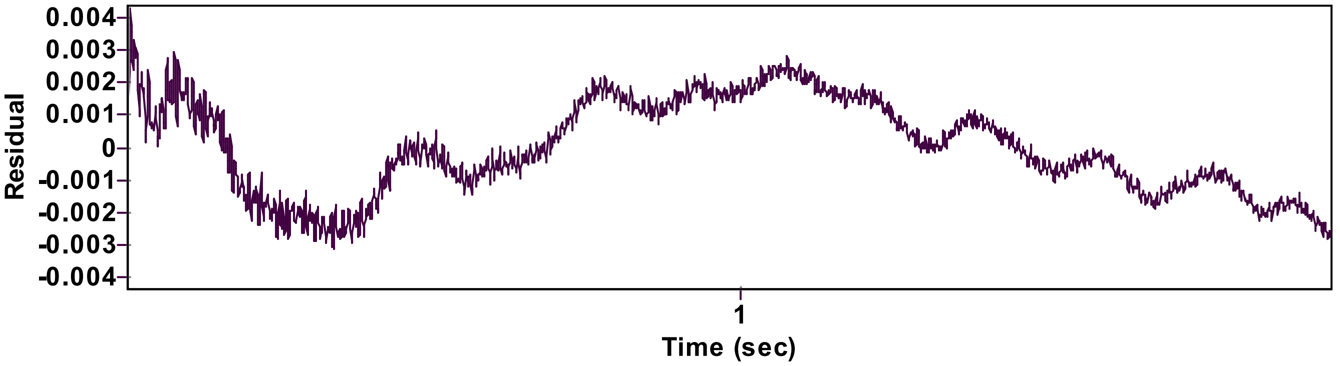

Function: y = A exp (-kx) + C (Exponential decrease)	Reference point: C (of function)
Amp A = 0.644156344636661 囟 0.000136932370546	Quality r^2 = 0.9999101513858
Rate k = 2.339165789398785 囟 0.001093910660130	Data points = 2547 of 4000
Final C = 0.347099746214764 囟 0.000062782845459	Conversion = 94.1 %
Start at position: 0.027 / 0.953155 (5.2 %)	End at position: 1.9365 / 0.351594 (99.3 %)
ExpoFit file: 20equiv-CH-Acid-mF-tBu-370nm.exp	Date of file: 08/02/2023 16:17:08
Source file: 20equiv-CH-Acid-mF-tBu-370nm.txt	Date of file: 08/02/2023 15:33:24
Type of source file: Universal ASCII - file data	
2007 by Dr. Kempf	Date of print: 10/02/2023 17:30:16
