# Supplementary material for: Lactone Enolates of Isochroman-3-ones and 2-Coumaranones: Quantification of Their Nucleophilicity in DMSO and Conjugate Additions to Chalcones
Source: J Org Chem. 2024 Apr 30;89(10):6915–28. doi: 10.1021/acs.joc.4c00277 (PMC11110064; doi:10.1021/acs.joc.4c00277)
Supplement: Supplementary file 2 — jo4c00277_si_002.zip [file jo4c00277_si_002.zip › 5+6f coumaranone_mF-tBu/30equiv-CH-Acid-mF-tBu-370nm.pdf]

Evaluation of kinetic data with ExpoFit V 1.3

Graph

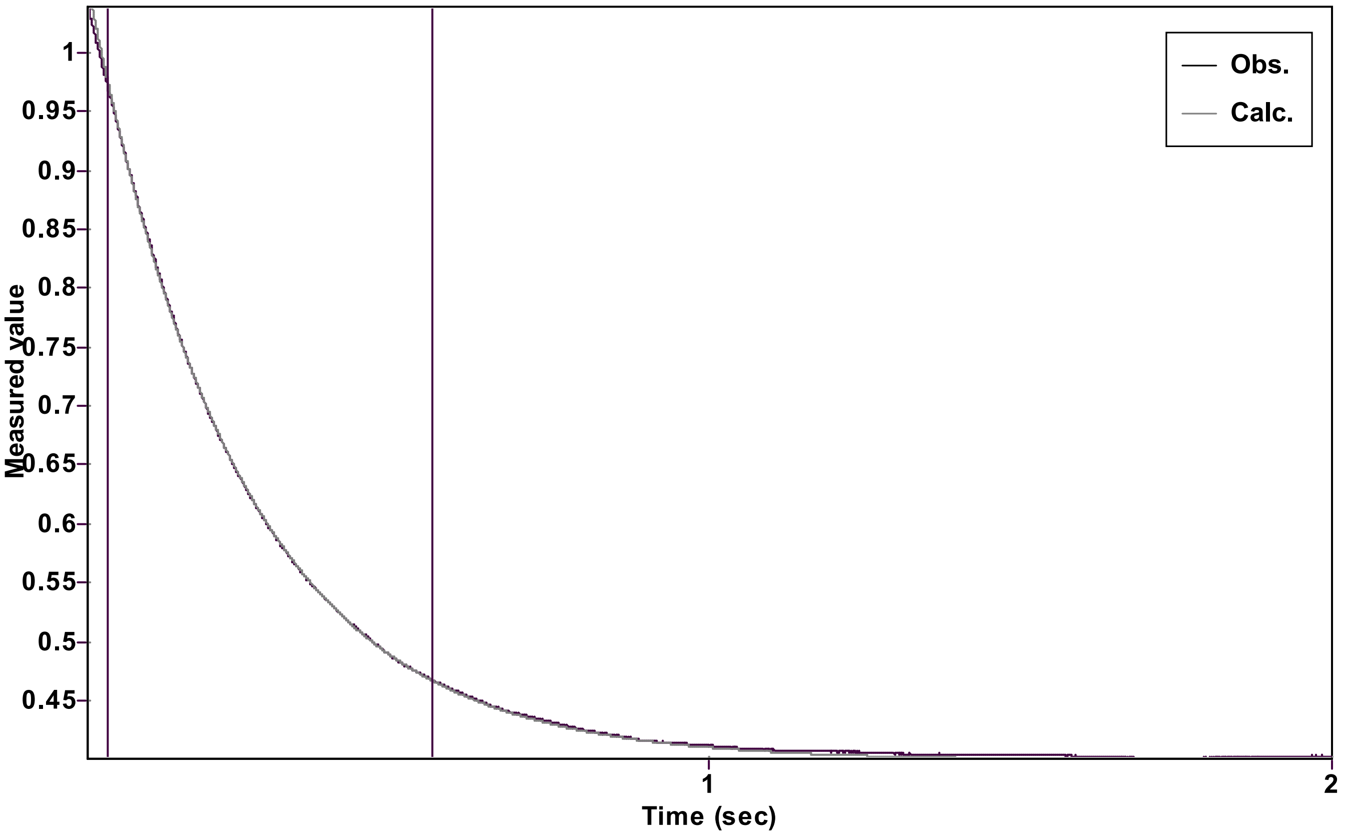

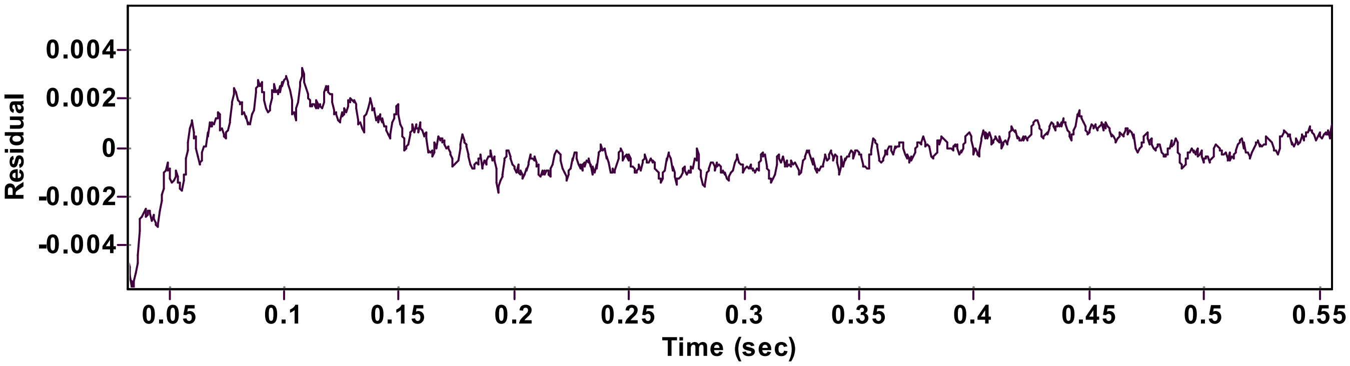

Function: y = A exp (-kx) + C (Exponential decrease)	Reference point: C (of function)
Amp A = 0.659582155577106 卤 0.000170694722545<br>Rate k = 4.112110750863613 卤 0.004018695386302<br>Final C = 0.399344801911559 卤 0.000226831475146	Quality r^2 = 0.9999371525835<br>Data points = 1048 of 4000<br>Conversion = 79.1 %
Start at position: 0.032 / 0.973019 (10.3 %)	End at position: 0.5555 / 0.467436 (89.4 %)
ExpoFit file: 30equiv-CH-Acid-mF-tBu-370nm.exp<br>Source file: 30equiv-CH-Acid-mF-tBu-370nm.txt<br>Type of source file: Universal ASCII - file data	Date of file: 08/02/2023 16:18:40<br>Date of file: 08/02/2023 15:39:20
2007 by Dr. Kempf	Date of print: 10/02/2023 17:30:56
