# Supplementary material for: Lactone Enolates of Isochroman-3-ones and 2-Coumaranones: Quantification of Their Nucleophilicity in DMSO and Conjugate Additions to Chalcones
Source: J Org Chem. 2024 Apr 30;89(10):6915–28. doi: 10.1021/acs.joc.4c00277 (PMC11110064; doi:10.1021/acs.joc.4c00277)
Supplement: Supplementary file 2 — jo4c00277_si_002.zip [file jo4c00277_si_002.zip › 5+6f coumaranone_mF-tBu/40equiv-CH-Acid-mF-tBu-370nm.pdf]

Evaluation of kinetic data with ExpoFit V 1.3

Graph

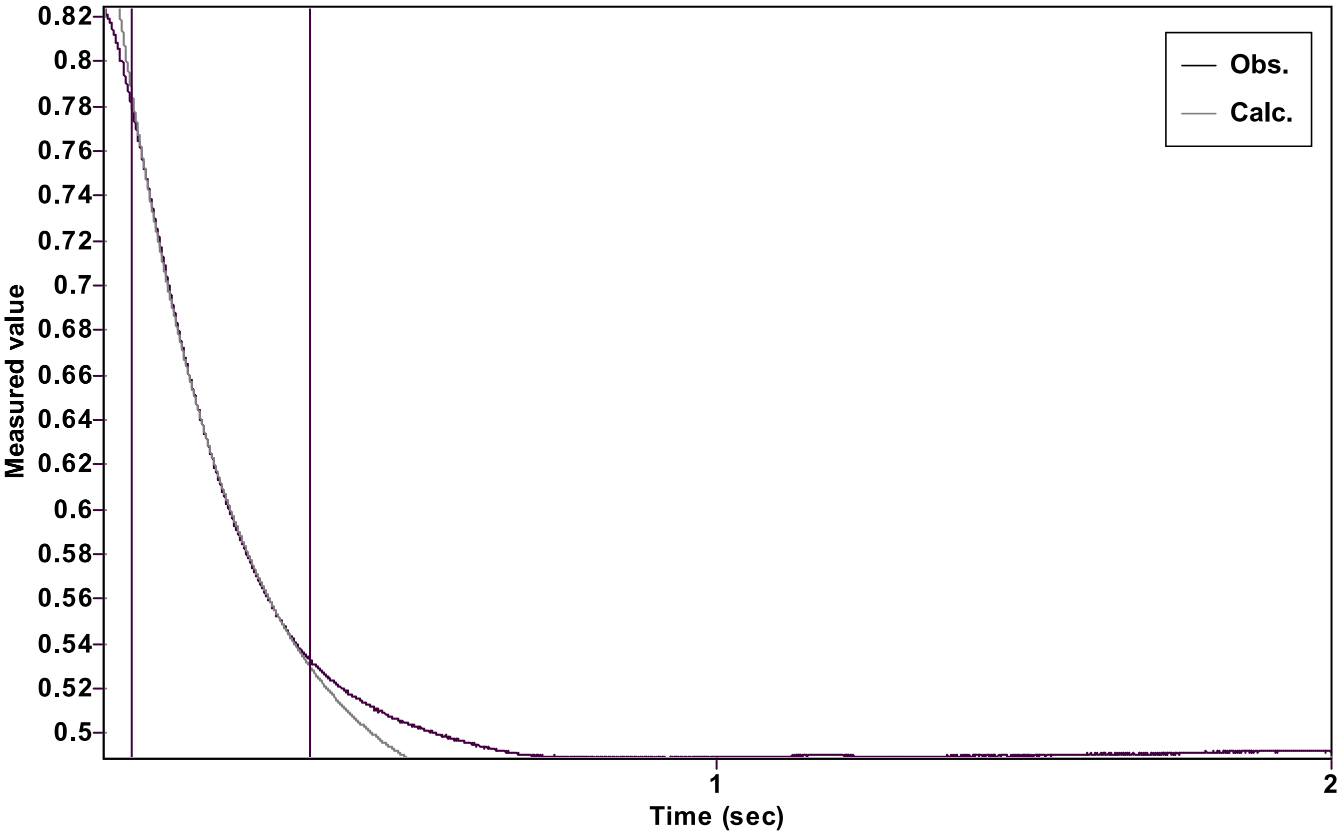

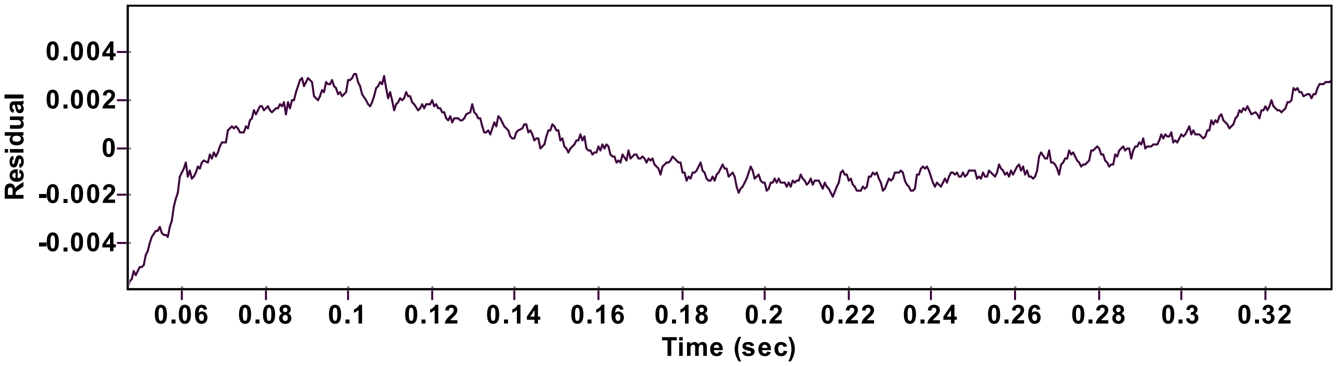

Function: y = A exp (-kx) + C (Exponential decrease)	Reference point: 0 (Zero)
Amp A = 0.419133491478025 囟 0.000501797118977<br>Rate k = 5.198766532933726 囟 0.026098168610176<br>Final C = 0.456858443862591 囟 0.000840500730468	Quality r^2 = 0.9995064737051<br>Data points = 579 of 4000<br>Conversion = 29.9 %
Start at position: 0.047 / 0.779143 (5.5 %)	End at position: 0.336 / 0.532739 (35.4 %)
ExpoFit file: 40equiv-CH-Acid-mF-tBu-370nm.exp<br>Source file: 40equiv-CH-Acid-mF-tBu-370nm.txt<br>Type of source file: Universal ASCII - file data	Date of file: 08/02/2023 16:20:22<br>Date of file: 08/02/2023 15:50:42
2007 by Dr. Kempf	Date of print: 10/02/2023 17:31:42
