# Supplementary material for: Lactone Enolates of Isochroman-3-ones and 2-Coumaranones: Quantification of Their Nucleophilicity in DMSO and Conjugate Additions to Chalcones
Source: J Org Chem. 2024 Apr 30;89(10):6915–28. doi: 10.1021/acs.joc.4c00277 (PMC11110064; doi:10.1021/acs.joc.4c00277)
Supplement: Supplementary file 2 — jo4c00277_si_002.zip [file jo4c00277_si_002.zip › 5+6g coumaranone_NO2-tBu/NO2-tBu_10eqcarbanion.pdf]

Evaluation of kinetic data with ExpoFit V 1.3

Graph

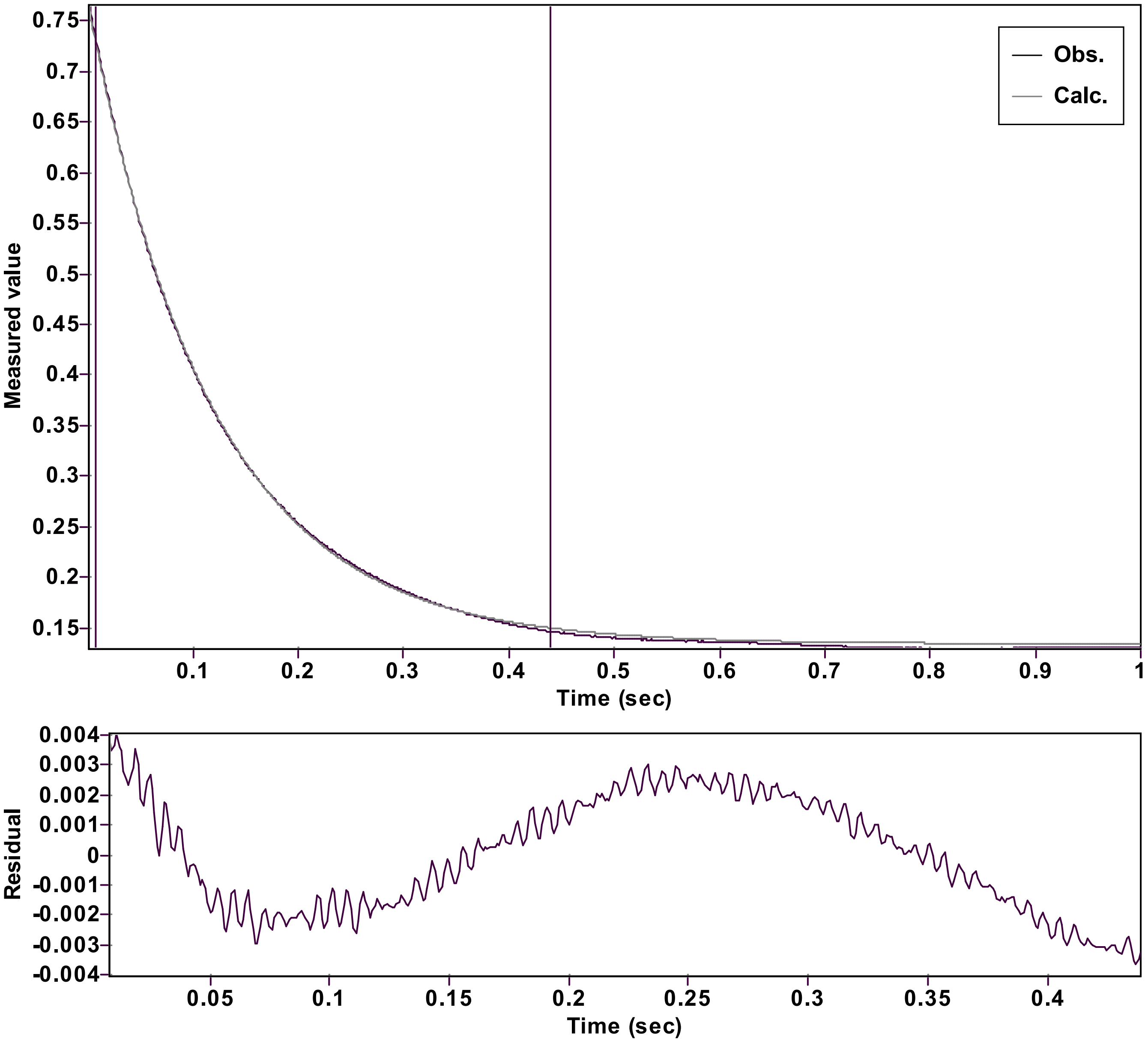

Function: y = A exp (-kx) + C (Exponential decrease)			Reference point: C (of function)
Amp A =	0.633819668040552	卤 0.000396641855642	Quality r^2 = 0.9998485341609
Rate k =	8.418457424833329	卤 0.013819895107902	Data points = 432 of 1000
Final C =	0.133956891010802	卤 0.000256884862769	Conversion = 92.4 %

Start at position: 0.008 / 0.730435 (5.6 %)

End at position: 0.439 / 0.146475 (98.0 %)

ExpoFit file: NO2-tBu_10eqcarbanion.exp — Date of file: 10/02/2023 17:35:30

Source file: NO2-tBu_10eqcarbanion.txt — Date of file: 10/02/2023 15:22:18

Type of source file: Universal ASCII - file data

2007 by Dr. Kempf — Date of print: 10/02/2023 17:36:50
