# Supplementary material for: Lactone Enolates of Isochroman-3-ones and 2-Coumaranones: Quantification of Their Nucleophilicity in DMSO and Conjugate Additions to Chalcones
Source: J Org Chem. 2024 Apr 30;89(10):6915–28. doi: 10.1021/acs.joc.4c00277 (PMC11110064; doi:10.1021/acs.joc.4c00277)
Supplement: Supplementary file 2 — jo4c00277_si_002.zip [file jo4c00277_si_002.zip › 5+6g coumaranone_NO2-tBu/NO2-tBu_20eqcarbanion.pdf]

Evaluation of kinetic data with ExpoFit V 1.3

Graph

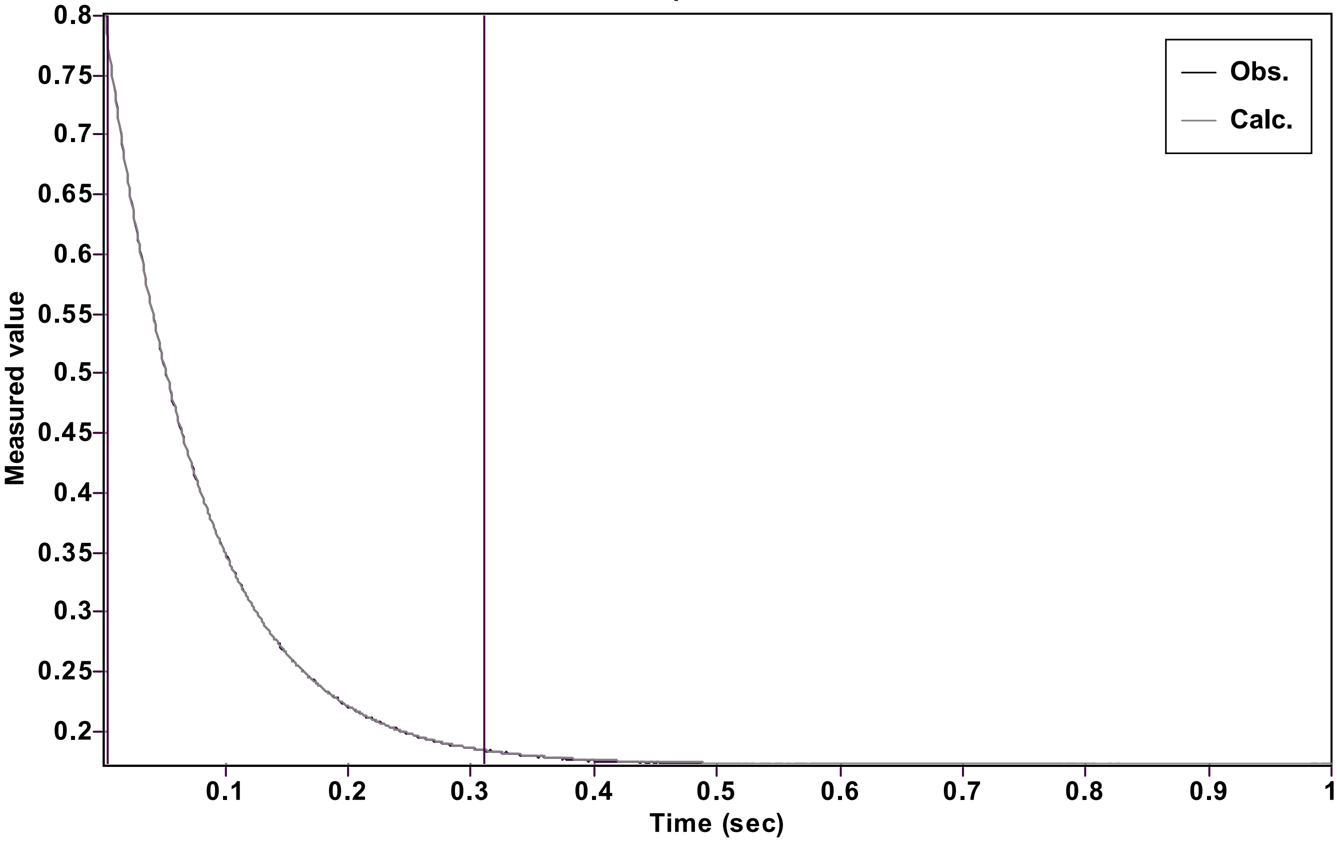

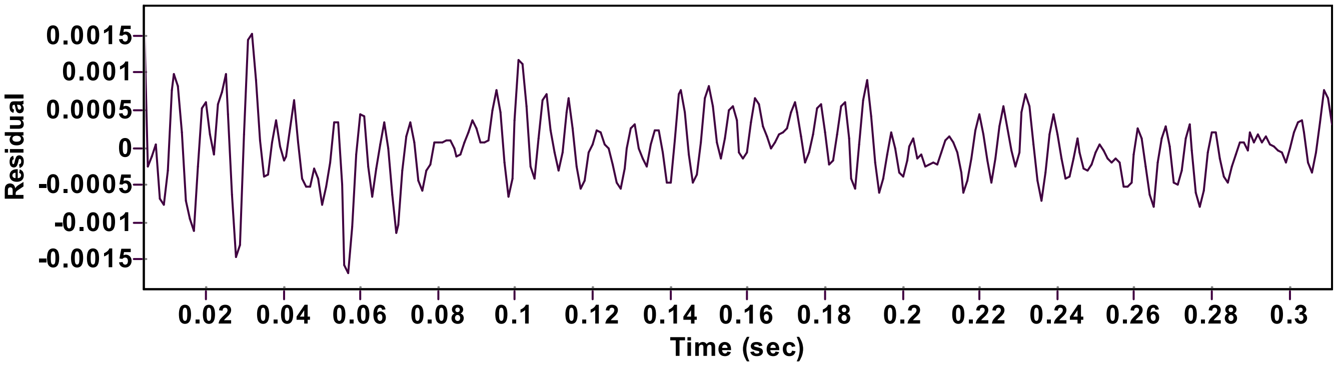

Function: y = A exp (-kx) + C (Exponential decrease)	Reference point: C (of function)
Amp A = 0.640282905250930 卤 0.000118098039046	Quality r^2 = 0.9999908315969
Rate k = 12.89755916599970 卤 0.005861228786124	Data points = 308 of 1000
Final C = 0.172491019104793 卤 0.000067951660722	Conversion = 95.1 %
Start at position: 0.004 / 0.782483 (3.0 %)	End at position: 0.311 / 0.184406 (98.1 %)
ExpoFit file: File not saved	Date of file: Not available
Source file: NO2-tBu_20eqcarbanion.txt	Date of file: 10/02/2023 15:29:58
Type of source file: Universal ASCII - file data	
2007 by Dr. Kempf	Date of print: 10/02/2023 17:46:21
