# Supplementary material for: Lactone Enolates of Isochroman-3-ones and 2-Coumaranones: Quantification of Their Nucleophilicity in DMSO and Conjugate Additions to Chalcones
Source: J Org Chem. 2024 Apr 30;89(10):6915–28. doi: 10.1021/acs.joc.4c00277 (PMC11110064; doi:10.1021/acs.joc.4c00277)
Supplement: Supplementary file 2 — jo4c00277_si_002.zip [file jo4c00277_si_002.zip › 5+6g coumaranone_NO2-tBu/NO2-tBu_30eqcarbanion.pdf]

Evaluation of kinetic data with ExpoFit V 1.3

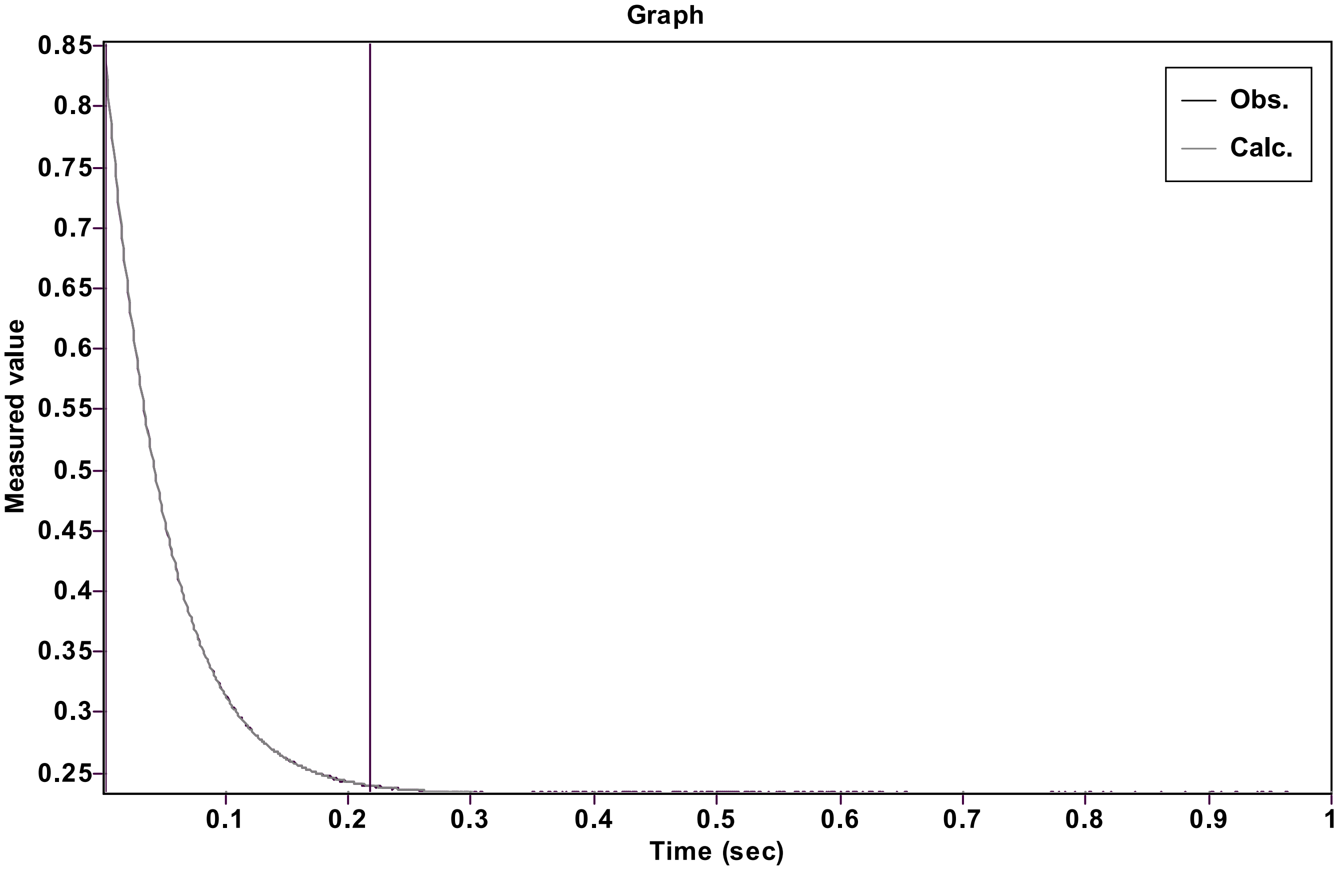

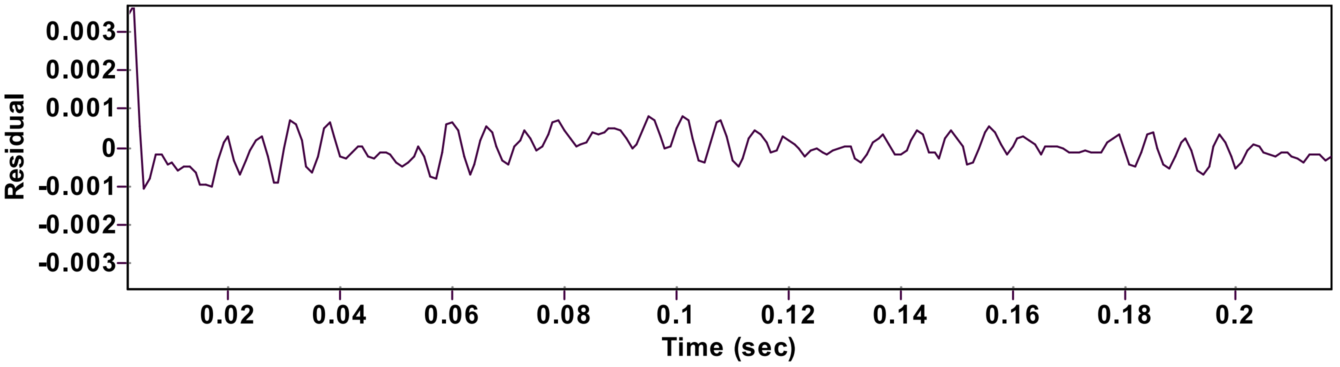

Function: y = A exp (-kx) + C (Exponential decrease)

Reference point: C (of function)

Amp A = 0.640155238718650 卤 0.000159061807361

Rate k = 20.58194015971414 卤 0.011649003395594

Final C = 0.232179327784630 卤 0.000078239434065

Quality r^2 = 0.9999894296568

Data points = 216 of 1000

Conversion = 98.3 %

Start at position: 0.002 / 0.849977 (0.6 %)

End at position: 0.217 / 0.239275 (98.9 %)

ExpoFit file: File not saved

Date of file: Not available

Source file: NO2-tBu_30eqcarbanion.txt

Date of file: 10/02/2023 15:41:06

Type of source file: Universal ASCII - file data

2007 by Dr. Kempf

Date of print: 10/02/2023 17:48:04
