# Supplementary material for: Lactone Enolates of Isochroman-3-ones and 2-Coumaranones: Quantification of Their Nucleophilicity in DMSO and Conjugate Additions to Chalcones
Source: J Org Chem. 2024 Apr 30;89(10):6915–28. doi: 10.1021/acs.joc.4c00277 (PMC11110064; doi:10.1021/acs.joc.4c00277)
Supplement: Supplementary file 2 — jo4c00277_si_002.zip [file jo4c00277_si_002.zip › 5+6g coumaranone_NO2-tBu/NO2-tBu_40eqcarbanion.pdf]

Evaluation of kinetic data with ExpoFit V 1.3

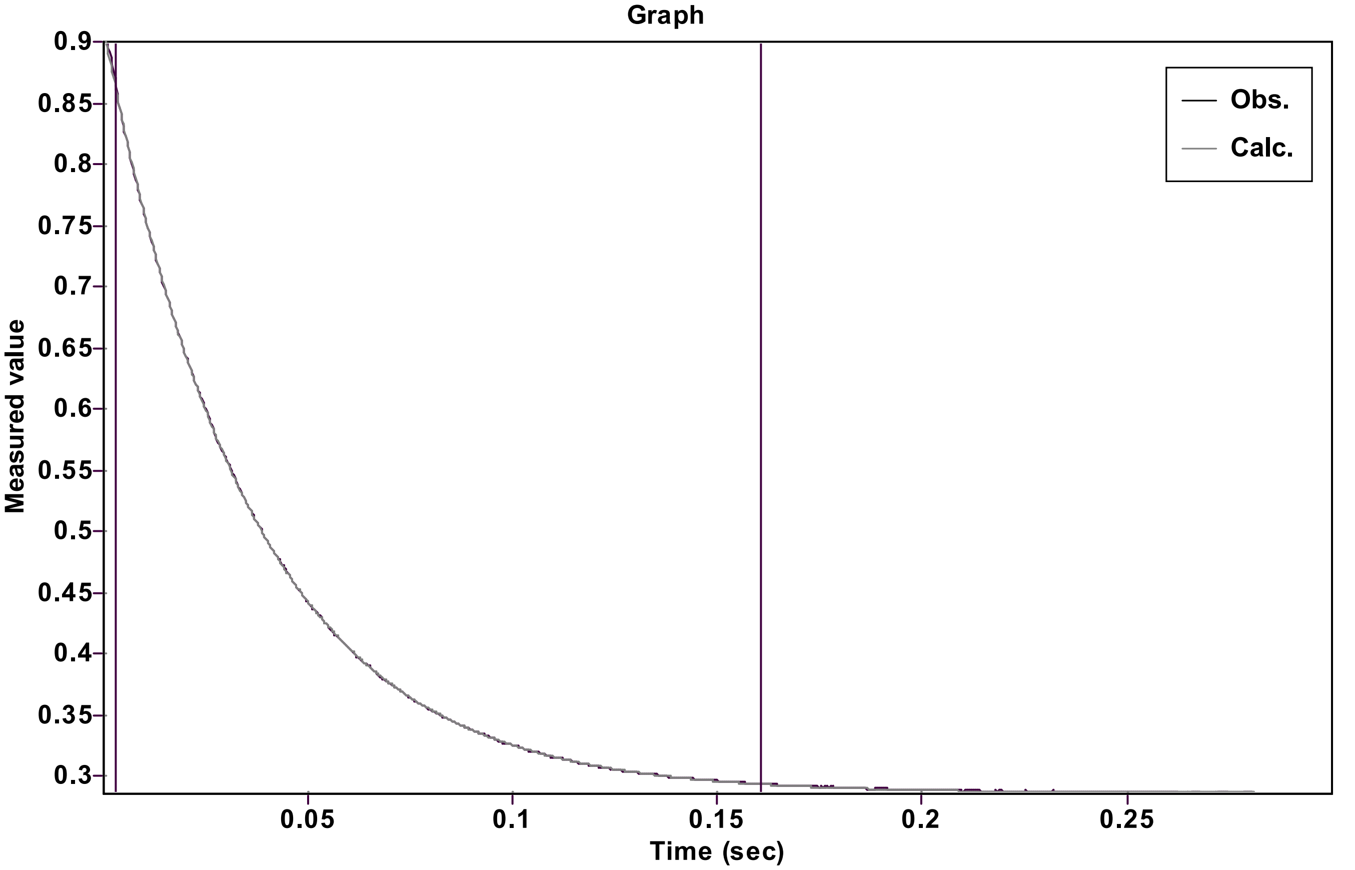

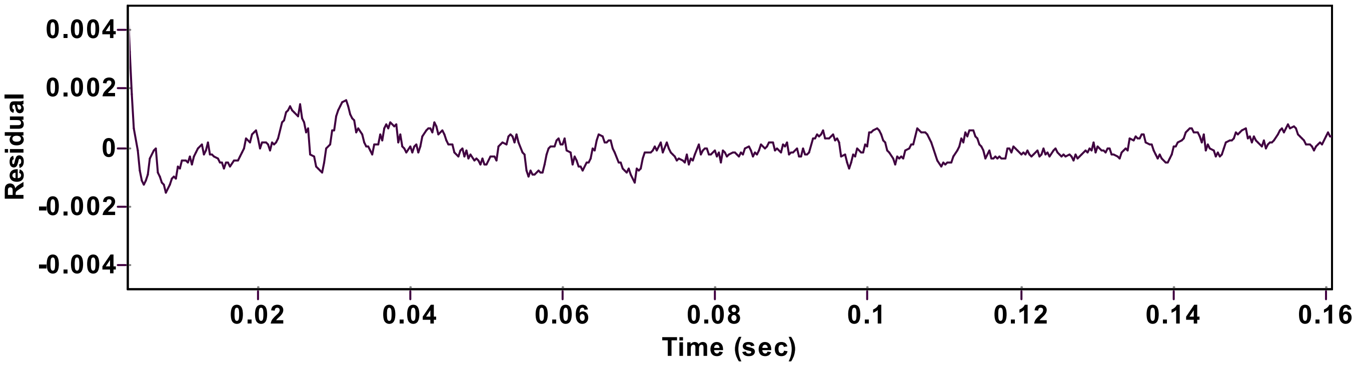

Function: y = A exp (-kx) + C (Exponential decrease)	Reference point: C (of function)
Amp A = 0.629518144884460 卤 0.000117300061655<br>Rate k = 27.76342855779963 卤 0.011340766496706<br>Final C = 0.286183897627521 卤 0.000053458302830	Quality r^2 = 0.9999847447190<br>Data points = 527 of 1000<br>Conversion = 93.8 %
Start at position: 0.003 / 0.870238 (4.9 %)	End at position: 0.1608 / 0.293818 (98.8 %)

ExpoFit file: File not saved — Date of file: Not available

Source file: NO2-tBu_40eqcarbanion.txt — Date of file: 10/02/2023 15:48:38

Type of source file: Universal ASCII - file data

2007 by Dr. Kempf — Date of print: 10/02/2023 17:50:11
