# Supplementary material for: Lactone Enolates of Isochroman-3-ones and 2-Coumaranones: Quantification of Their Nucleophilicity in DMSO and Conjugate Additions to Chalcones
Source: J Org Chem. 2024 Apr 30;89(10):6915–28. doi: 10.1021/acs.joc.4c00277 (PMC11110064; doi:10.1021/acs.joc.4c00277)
Supplement: Supplementary file 2 — jo4c00277_si_002.zip [file jo4c00277_si_002.zip › 5+6h coumaranone_dma-Ph/dma-Ph_10equicarbanion.pdf]

Evaluation of kinetic data with ExpoFit V 1.3

Graph

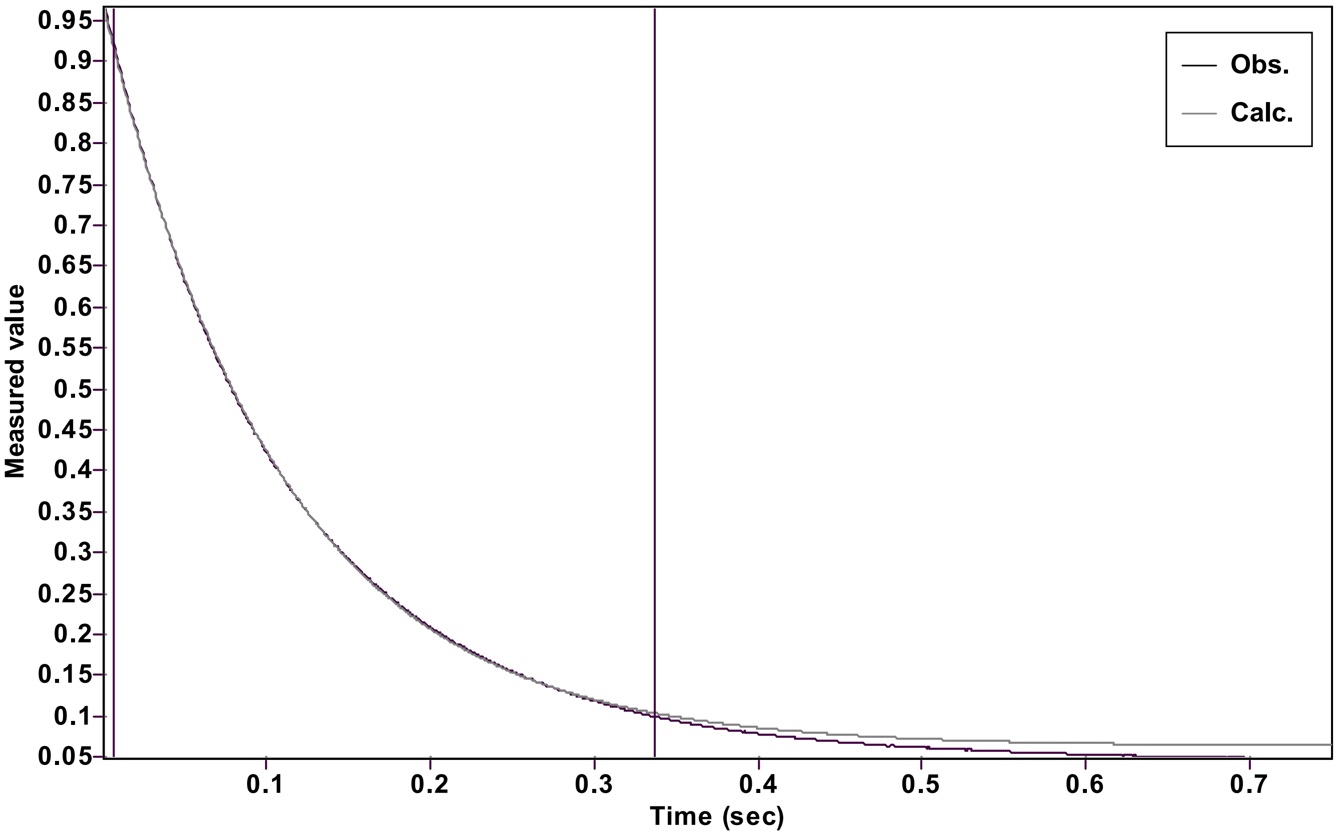

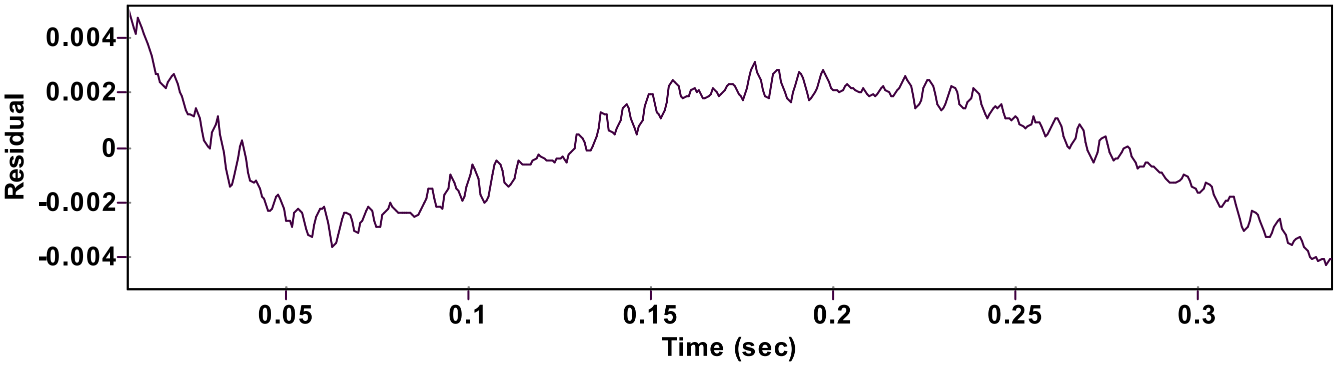

Function: y = A exp (-kx) + C (Exponential decrease)

Reference point: C (of function)

Amp A = 0.909205925184082 卤 0.000401586698945

Rate k = 9.237394506048670 卤 0.012505656967674

Final C = 0.063422108778114 卤 0.000353105051088

Quality r^2 = 0.9999151819631

Data points = 441 of 1000

Conversion = 91.0 %

Start at position: 0.00675 / 0.922868 (4.9 %)

End at position: 0.33675 / 0.0998442 (96.0 %)

ExpoFit file: File not saved

Date of file: Not available

Source file: dma-Ph_10equicarbanion.txt

Date of file: 16/02/2023 14:38:16

Type of source file: Universal ASCII - file data

2007 by Dr. Kempf

Date of print: 16/02/2023 15:16:54
