# Supplementary material for: Lactone Enolates of Isochroman-3-ones and 2-Coumaranones: Quantification of Their Nucleophilicity in DMSO and Conjugate Additions to Chalcones
Source: J Org Chem. 2024 Apr 30;89(10):6915–28. doi: 10.1021/acs.joc.4c00277 (PMC11110064; doi:10.1021/acs.joc.4c00277)
Supplement: Supplementary file 2 — jo4c00277_si_002.zip [file jo4c00277_si_002.zip › 5+6h coumaranone_dma-Ph/dma-Ph_20equicarbanion.pdf]

Evaluation of kinetic data with ExpoFit V 1.3

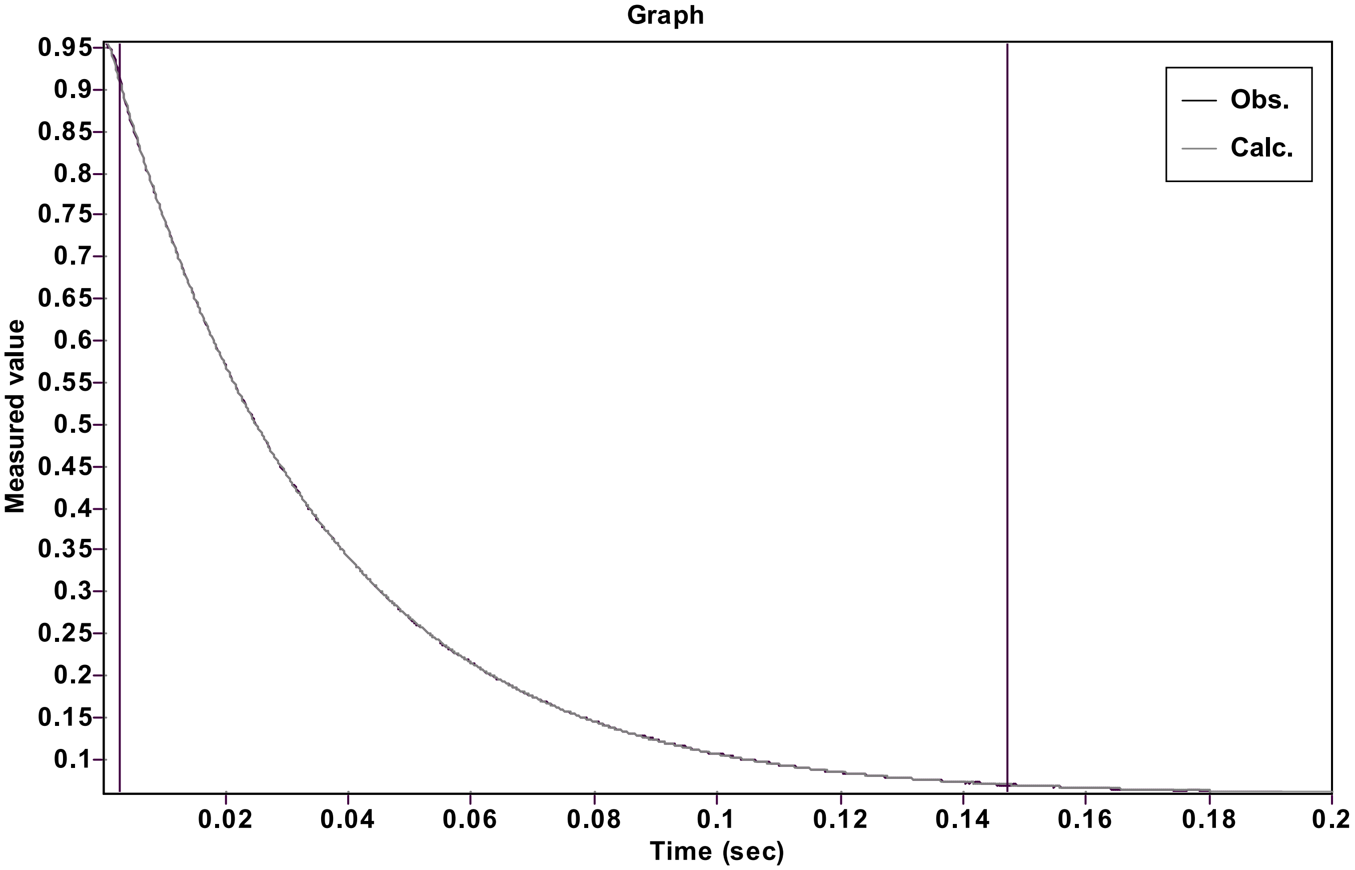

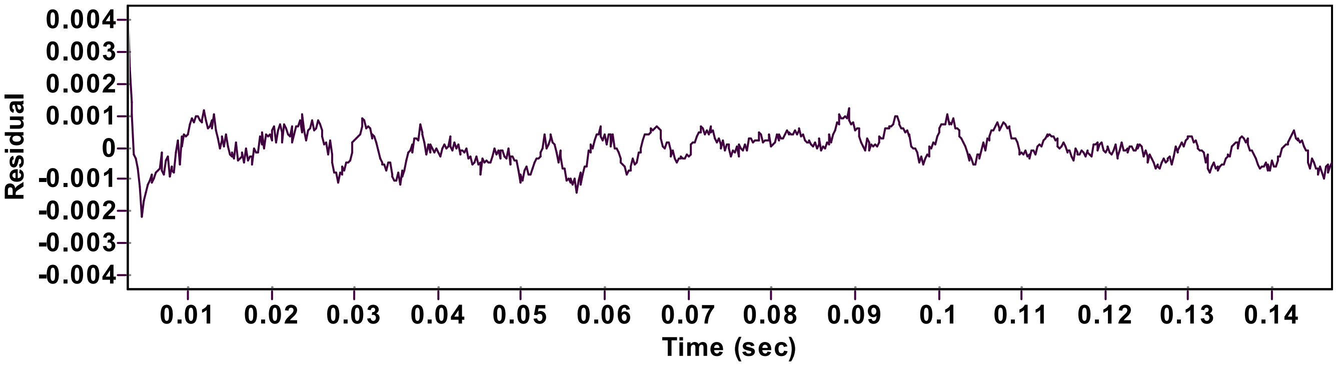

Function: y = A exp (-kx) + C (Exponential decrease)	Reference point: C (of function)
Amp A = 0.923914333605859 鹵 0.000096831171988	Quality r^2 = 0.9999937801768
Rate k = 29.53259440209760 鹵 0.006935053347701	Data points = 723 of 1000
Final C = 0.058191297507942 鹵 0.000046100416307	Conversion = 93.9 %

Start at position: 0.0028 / 0.91322 (4.9 %)	End at position: 0.1472 / 0.0696473 (98.7 %)

ExpoFit file: File not saved	Date of file: Not available
Source file: dma-Ph_20equicarbanion.txt	Date of file: 16/02/2023 14:43:22
Type of source file: Universal ASCII - file data	

2007 by Dr. Kempf — Date of print: 16/02/2023 15:18:44
