# Supplementary material for: Lactone Enolates of Isochroman-3-ones and 2-Coumaranones: Quantification of Their Nucleophilicity in DMSO and Conjugate Additions to Chalcones
Source: J Org Chem. 2024 Apr 30;89(10):6915–28. doi: 10.1021/acs.joc.4c00277 (PMC11110064; doi:10.1021/acs.joc.4c00277)
Supplement: Supplementary file 2 — jo4c00277_si_002.zip [file jo4c00277_si_002.zip › 5+6h coumaranone_dma-Ph/dma-Ph_30equicarbanion.pdf]

Evaluation of kinetic data with ExpoFit V 1.3

Graph

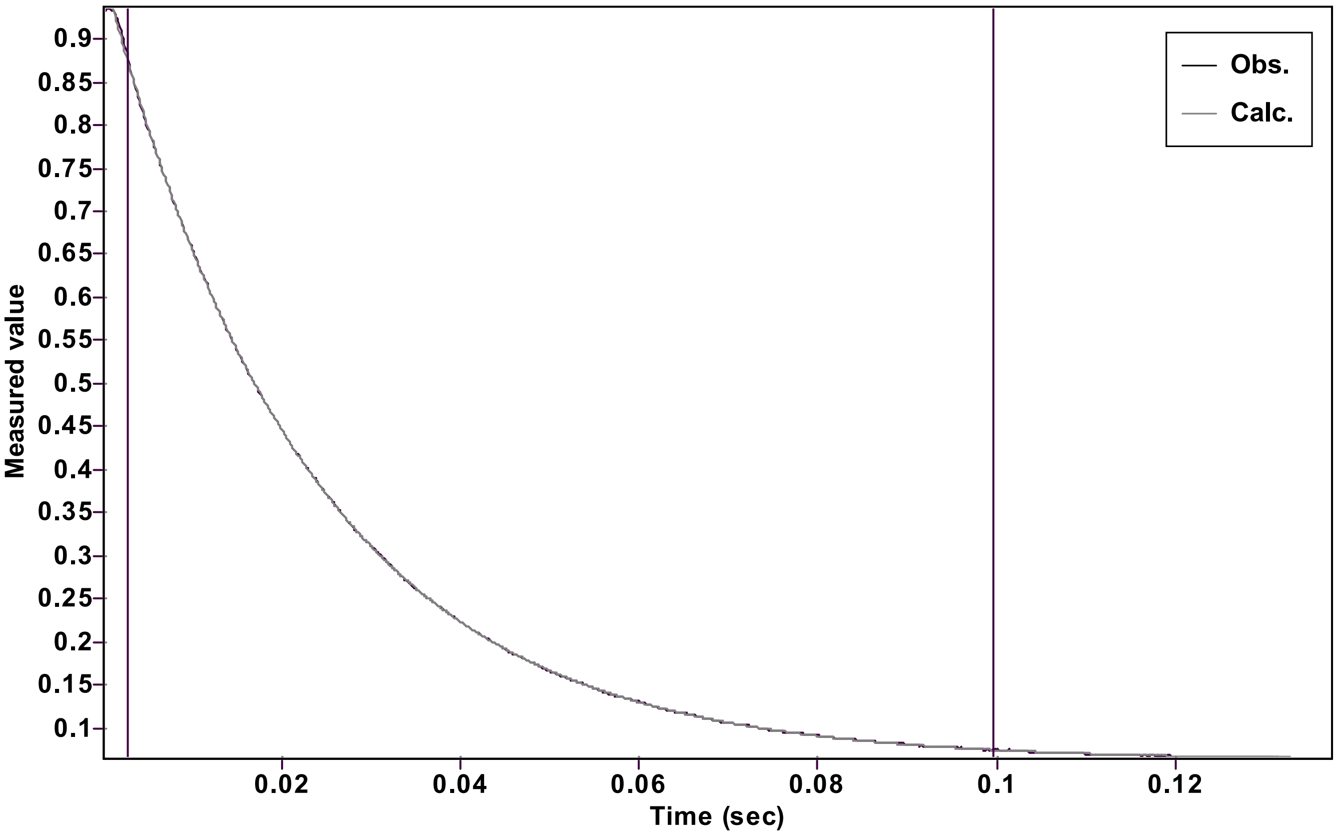

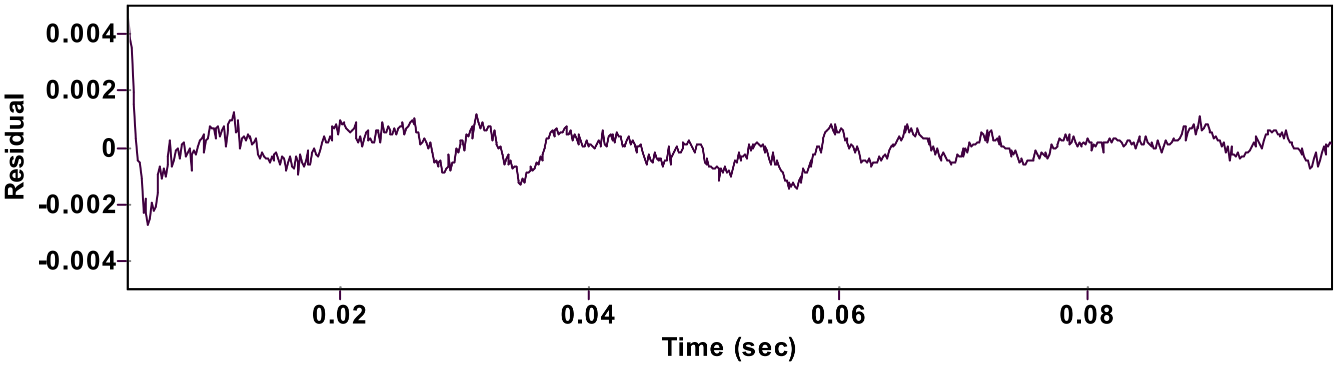

Function: y = A exp (-kx) + C (Exponential decrease)

Reference point: C (of function)

Amp A = 0.918065995842589 卣 0.000123747887062
Rate k = 43.50924876895968 卣 0.012661566625353
Final C = 0.062302820329565 卣 0.000054951315619

Quality r^2 = 0.9999909938449
Data points = 705 of 1000
Conversion = 91.7 %

Start at position: 0.0028875 / 0.877003 (6.9 %)

End at position: 0.0996875 / 0.0743914 (98.6 %)

ExpoFit file: File not saved
Date of file: Not available
Source file: dma-Ph_30equicarbanion.txt
Date of file: 16/02/2023 14:47:12
Type of source file: Universal ASCII - file data

2007 by Dr. Kempf
Date of print: 16/02/2023 15:20:42
