# Supplementary material for: Lactone Enolates of Isochroman-3-ones and 2-Coumaranones: Quantification of Their Nucleophilicity in DMSO and Conjugate Additions to Chalcones
Source: J Org Chem. 2024 Apr 30;89(10):6915–28. doi: 10.1021/acs.joc.4c00277 (PMC11110064; doi:10.1021/acs.joc.4c00277)
Supplement: Supplementary file 2 — jo4c00277_si_002.zip [file jo4c00277_si_002.zip › 5+6h coumaranone_dma-Ph/dma-Ph_40equicarbanion.pdf]

Evaluation of kinetic data with ExpoFit V 1.3

Graph

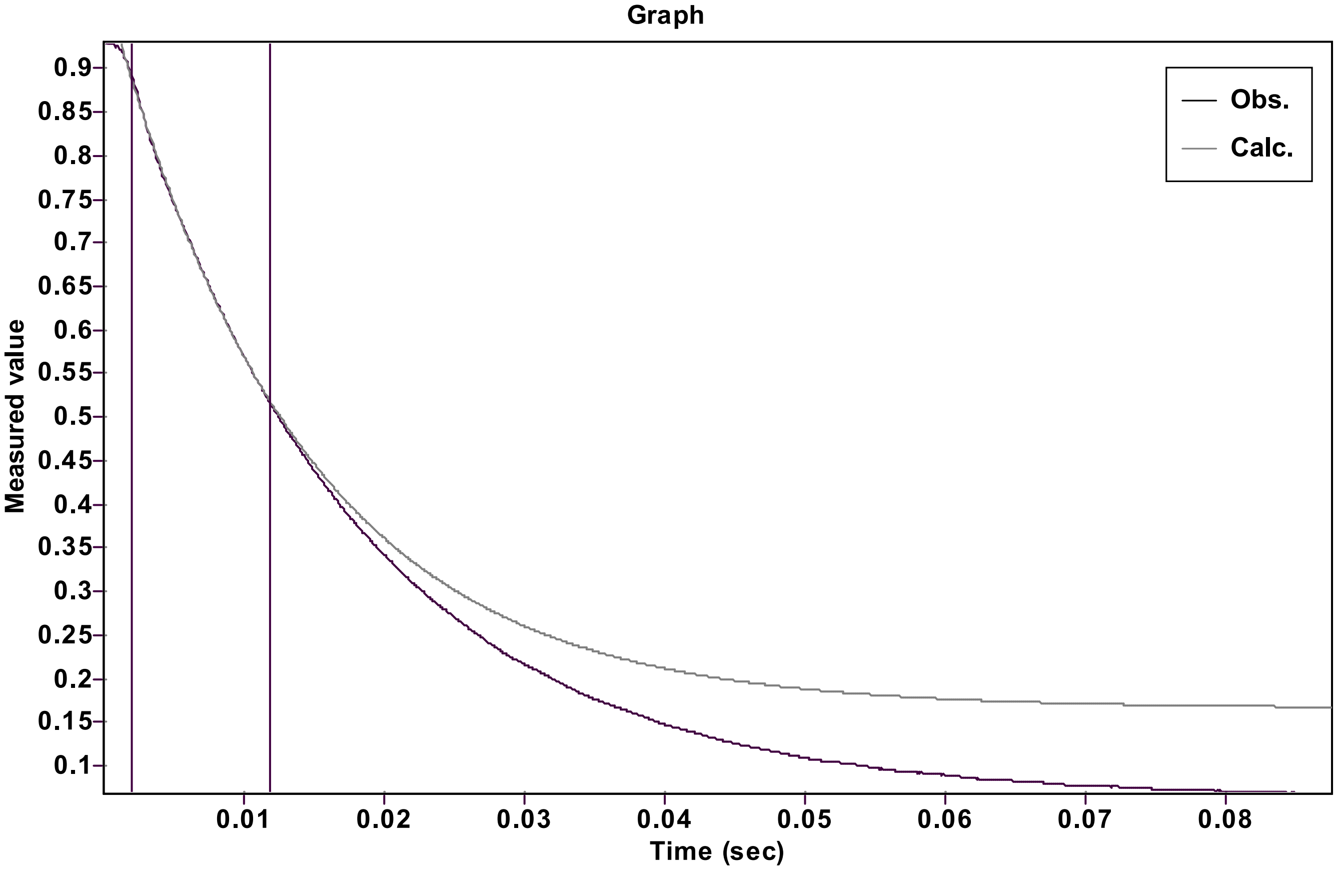

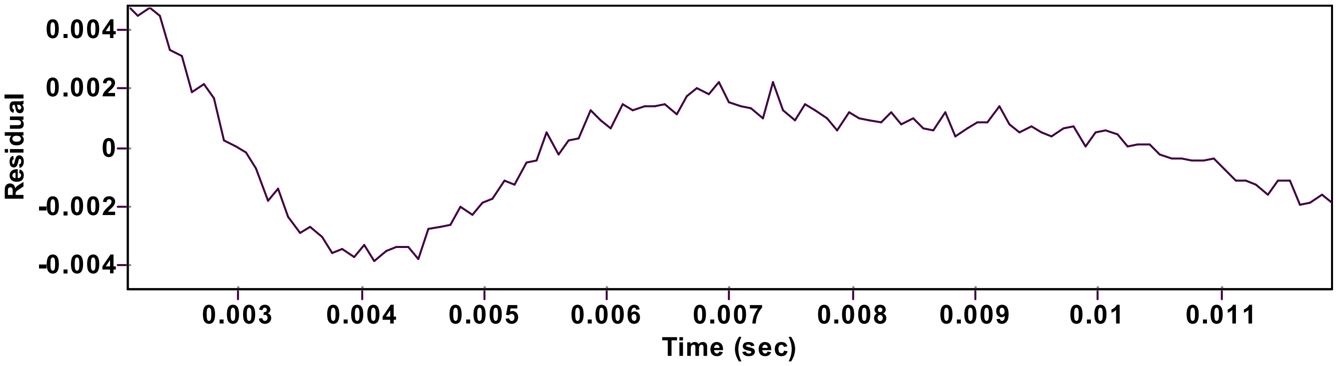

Function: y = A exp (-kx) + C (Exponential decrease)	Reference point: C (of function)
Amp A = 0.840208243643766 卤 0.008246180100782	Quality r^2 = 0.9996849464802
Rate k = 72.83618827210172 卤 1.342560030528507	Data points = 113 of 1000
Final C = 0.166152775543786 卤 0.009464668425407	Conversion = 49.0 %
Start at position: 0.0021 / 0.89205 (5.0 %)	End at position: 0.0119 / 0.517419 (54.1 %)
ExpoFit file: File not saved	Date of file: Not available
Source file: dma-Ph_40equicarbanion.txt	Date of file: 16/02/2023 14:53:42
Type of source file: Universal ASCII - file data	
2007 by Dr. Kempf	Date of print: 16/02/2023 15:23:46
